# Isothiourea-Mediated One-Pot Synthesis of Functionalized Pyridines**

**DOI:** 10.1002/anie.201306786

**Published:** 2013-09-17

**Authors:** Daniel G Stark, Louis C Morrill, Pei-Pei Yeh, Alexandra M Z Slawin, Timothy J C O'Riordan, Andrew D Smith

**Affiliations:** EaStCHEM, School of Chemistry, University of St AndrewsNorth Haugh, St Andrews, Fife, KY16 9ST (UK); Syngenta, Jealott's Hill International Research CentreBracknell, RG42 6EY (UK)

**Keywords:** cyclization, heterocycles, isothiourea, Lewis base, organocatalysis

Pyridines are an extremely privileged heterocyclic class commonly found in natural products and functional materials. They are also important building blocks in both the agrochemical and pharmaceutical industries.^1^ Consequently, a vast array of synthetic methods has been successfully developed to access these useful molecules.^2^ Despite many recent advances, novel methods for the synthesis of highly functionalized pyridines in a selective and high yielding manner from accessible starting materials remains an important goal within the synthetic community.^3^

Following the demonstration by Romo and co-workers of generating ammonium enolates^4^ from carboxylic acids,^5^ we have shown that isothioureas^6^, ^7^ catalyze the intermolecular Michael addition/lactonization/lactamization of arylacetic acids and electron-deficient Michael acceptors.^8^ To expand this mode of activation, we questioned whether this methodology could be used to access functionalized pyridines. Conceptually, an isothiourea-catalyzed reaction of an acetic acid bearing an α-leaving group with a suitably electron-deficient α,β-unsaturated ketimine **1**^9^ would result in Michael addition/lactamization with subsequent elimination to form the pyridones **2** (Scheme 1).^10^ Subsequent N- to O-sulfonyl migration^11^ would allow the pyridines **3** to be accessed directly in one pot. Importantly, in this process the activating sulfonyl group on the ketimine would be transformed into a synthetically useful functional handle (the 2-sulfonate group) in the resultant pyridines, thus allowing subsequent derivatization into a variety of products.

**Scheme 1:**
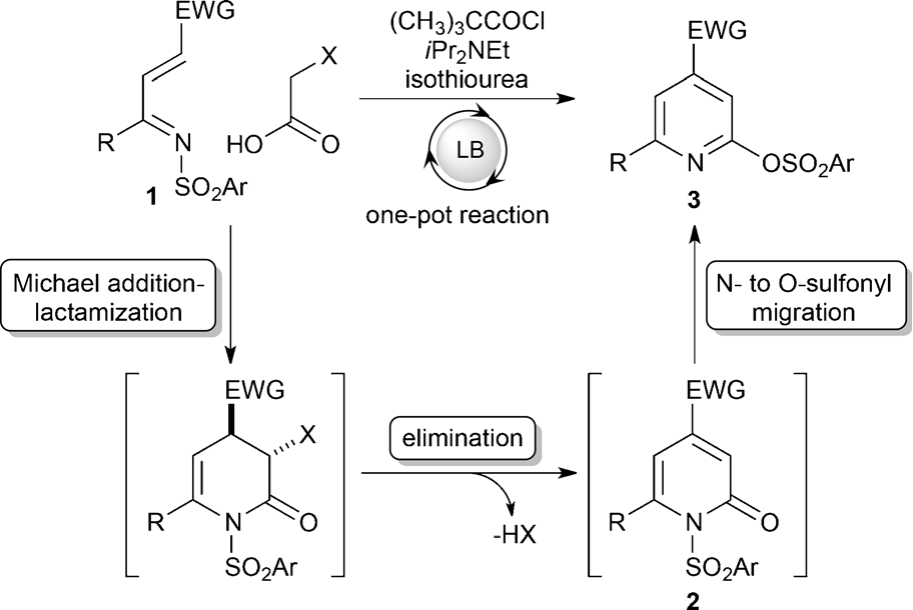
Proposed strategy for functionalized pyridines. LB=Lewis base.

After initial screening,^12^ commercially available (phenylthio)acetic acid **4** was identified as a suitable acid in this process. Treatment of **4** with pivaloyl chloride and *i*Pr_2_NEt gave the corresponding mixed anhydride in situ. Subsequent addition of the α,β-unsaturated ketimine **5**^13^ in the presence of the isothiourea DHPB (**6**; 20 mol %) in CH_2_Cl_2_ at 0 °C for 4 hours afforded the pyridine **7** in only 7 % yield after chromatographic purification,^14^ despite complete consumption of **5** (Table 1, entry 1). Optimization of this process showed that a combination of increased temperature and changing the solvent gave a higher yield of the isolated pyridine (entries 2–4). The increased temperatures are necessary to promote effective N- to O-sulfonyl migration in this process, with lower temperatures leading to mixtures of the intermediate pyridone and desired pyridine. The use of a microwave reactor led to good product yields (entry 5), and alternative reaction concentrations or catalysts (**8**–**10**) had a negative effect upon the yield of the isolated product (entries 6–10). Using **6** and extending the reaction time to 16 h in THF at 80 °C was determined to be the optimal reaction conditions, thus giving **7** in 67 % yield (entry 10).^15^

**Table 1:** Reaction optimization
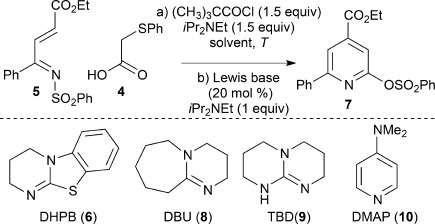

Entry	Cat.	Solvent	*T* [°C]	*t* [h]	Yield [%]^[a]^
1	6	CH_2_Cl_2_	0	4	7
2	6	CH_2_Cl_2_	RT	4	30
3	6	1,4-dioxane	80	16	52
4	6	THF	80	4	49
5	6	THF	80^[b]^	2	52
6^[c]^	6	THF	80	4	10
7	8	THF	80	4	13
8	9	THF	80	4	–
9	10	THF	80	4	36
10	6	THF	80	16	67

[a] Yield of isolated **7** following chromatography. [b] Biotage Initiator with a program of heating to 80 °C at maximum power of 150 W. [c] 0.0067 m in the ketimine **5** (typically 0.067 m). DBU=1,8-diazabicyclo[5.4.0]undec-7-ene, DHPB=(3,4-dihydro-2*H*-pyrimido[2,1-*b*]benzothiazole), DMAP=4-(*N*,*N*-dimethylamino)pyridine, TBD=1,5,7-triazabicyclo[4.4.0]dec-5-ene.

The generality of this process was next investigated (Table 2). Under optimized reaction conditions, this process tolerates a variety of α,β-unsaturated ketimines. The N-sulfonyl group (benzenesulfonyl and tosyl) can be altered, whilst a variety of different esters (methyl, ethyl, and benzyl) are also tolerated at the β position. Additionally, a range of aryl groups bearing electron-withdrawing (-NO_2_, -CN,-CF_3_), electron-donating (-OMe, -Me), and halogen (-F, -Cl) substituents are efficient substrates in this process, along with alkyl substitution. The functionalized trisubstituted pyridines **7** and **11**–**21** are formed in moderate to good yields following the three consecutive synthetic transformations in one pot.

**Table 2:** Reaction scope.^[a]^

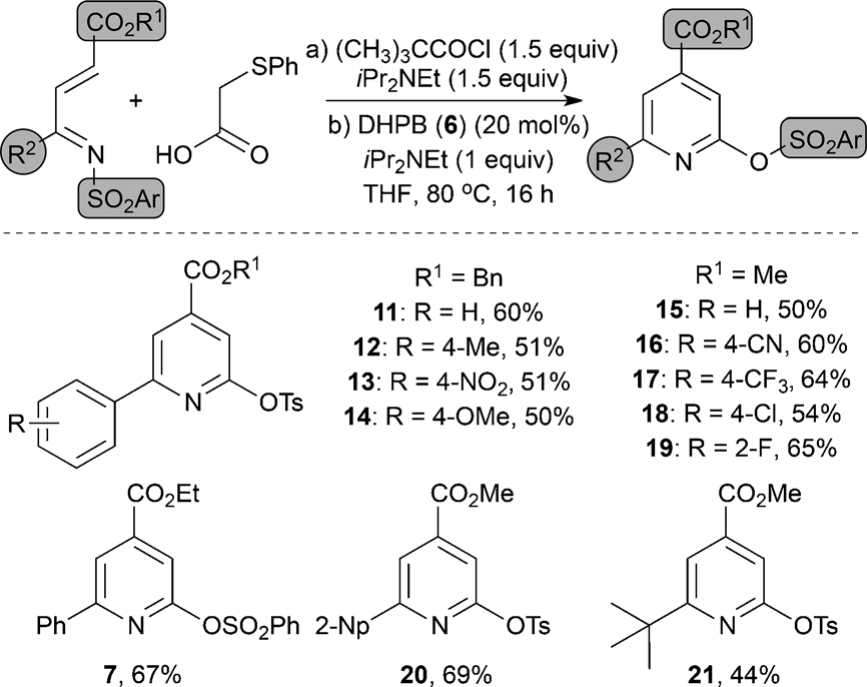

[a] Yield is that of the product isolated after chromatography. Np=Naphthyl, Ts=*p*-toluenesulfonyl.

Given the wide interest in the preparation of functional heterocycles containing a trifluoromethyl unit, this protocol was extended to the synthesis of 4- and 6-trifluoromethyl-containing pyridines. Trifluoromethyl-containing α,β-unsaturated ketimines were readily prepared from the corresponding enones and used in this protocol, thus giving pyridines in acceptable yields (40–66 %; Table 3). Variation of the sulfonyl unit, as well as incorporation of heteroaryl (2-furyl and 2-thiophenyl) and aryl substituents was explored, with the incorporation of 3- and 4-bromosubstituted aromatics targeted to allow the possibility of derivatization by cross-coupling.^16^, ^17^ This pyridine-forming protocol is readily applicable to large-scale synthesis, thus forming the pyridine **22** on a 26 mmol scale using **6** (20 mol %) to generate 5.2 g of product (51 % yield). The pyridine **22** can also be accessed from the corresponding (*Z*)-ketimine under standard reaction conditions in 54 % yield. In addition, the isomeric pyridine **33** could be isolated in 40 % yield from the corresponding ketimine after heating for 48 h.^18^

**Table 3:** Reaction scope using trifluoromethyl α,β-unsaturated ketimines.^[a]^

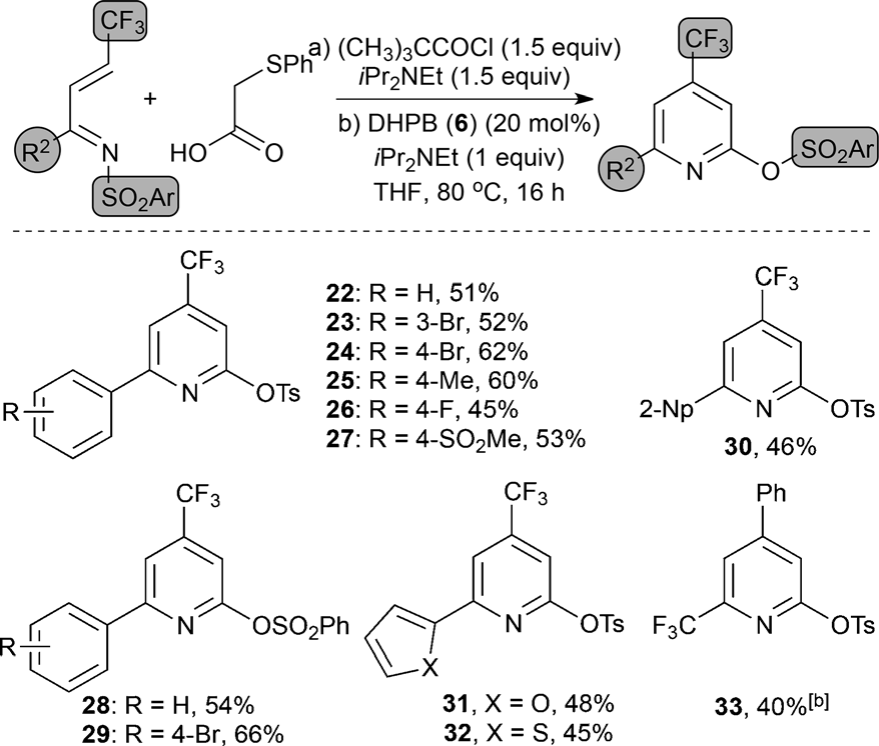

[a] Yield is that of the product isolated after chromatography. [b] 48 h. Np=Naphthyl, Ts=*p*-toluenesulfonyl.

The proposed reaction mechanism proceeds by initial formation of the mixed anhydride **34** from **4** and pivaloyl chloride with subsequent *N*-acylation of DHPB to generate the corresponding acyl isothiouronium ion **35** (Figure 1). Deprotonation generates the (*Z*)-enolate **36**, which undergoes Michael addition with the α,β-unsaturated ketimine **37** and subsequent intramolecular lactamization to generate the corresponding dihydropyridinone **38** and regenerate DHPB. Subsequent elimination of thiophenol gives the pyridone **39**, which undergoes thermally promoted N- to O-sulfonyl migration to afford the pyridine **40**.^19^

**Figure 1:**
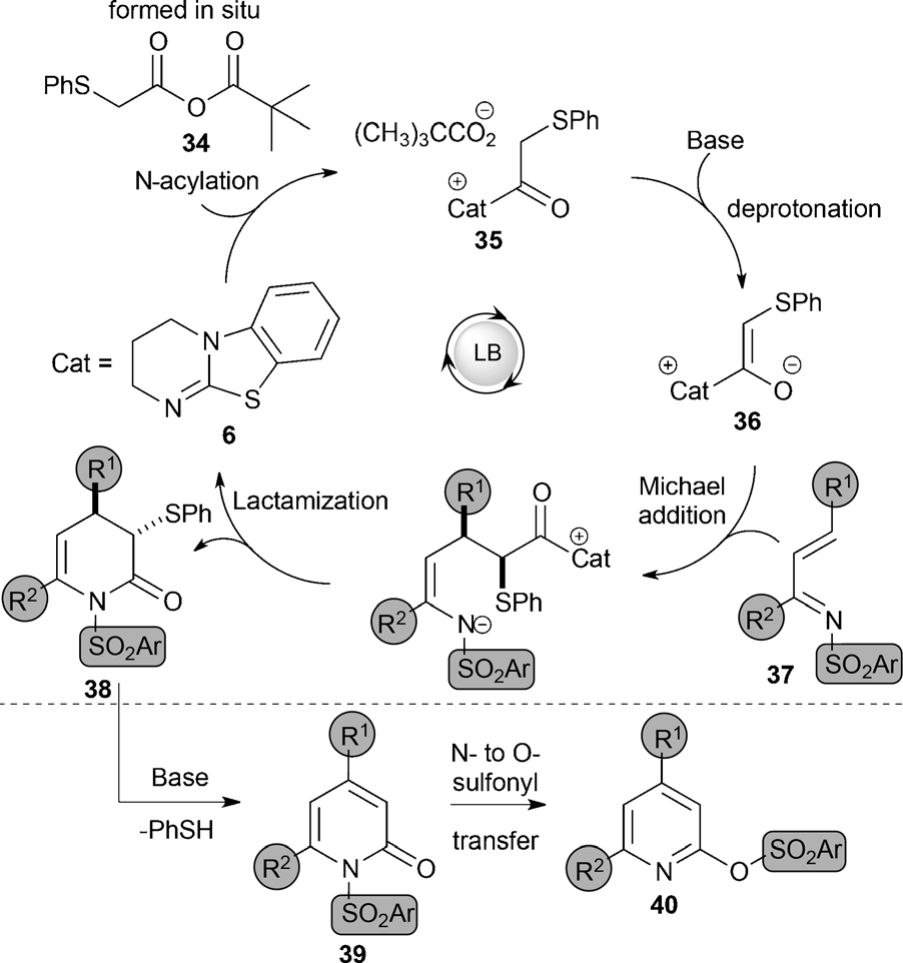
Proposed reaction mechanism.

Within the literature, sporadic examples of the key N- to O-sulfonyl rearrangement utilized in this process have been observed, usually as an undesired nonproductive pathway in thermally promoted Diels–Alder reactions of sulfonyl pyridones.^11^ To understand this process further, a crossover experiment was performed to determine if the N- to O-sulfonyl migration process involves an intra- or intermolecular transfer (Scheme 2). Reaction of **4** (1 equiv) with a 50:50 mixture of the ketimines **41** and **42** under the optimized reaction conditions led exclusively to the pyridines **24** and **28**, respectively, in a 50:50 mixture, and is consistent with intramolecular N- to O-sulfonyl transfer from an intermediate pyridone being involved within this process.

**Scheme 2:**
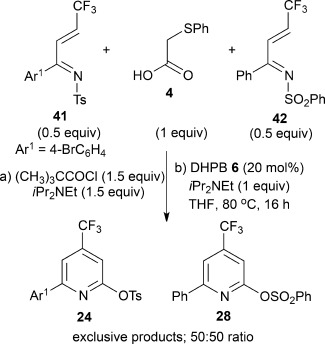
Crossover experiment to determine intra- or intermolecular N- to O-sulfonyl transfer.

The synthetic utility of the newly installed 2-sulfonate functional handle was next demonstrated through a series of product derivatizations to install H, aryl, heteroaryl, alkyl, and amino substituents (Scheme 3). For example, **22** can be reduced to give the 2,4-substituted pyridine **43** in 89 % yield,^20^ and it also readily undergoes S_N_Ar with morpholine to give **44** in 85 % yield.^21^, ^22^ The pyridine substrates are compatible with traditional cross-coupling methodologies, thus leading to diverse 2,4,6-substituted pyridines. For example, **22** undergoes a Mizoroki–Heck reaction with *N*-vinylacetamide to afford the pyridine **45** in 76 % yield,^23^ Suzuki coupling to give **46** in 81 % yield,^24^, ^25^ and Kumada cross-coupling to give **47** in 66 % yield.^26^ In addition to sp^2^–sp^2^ coupling reactions, iron-catalyzed sp^2^–sp^3^ coupling of **22** with *n*-hexylmagnesium bromide gives **48** in 74 % yield.^27^, ^28^

**Scheme 3:**
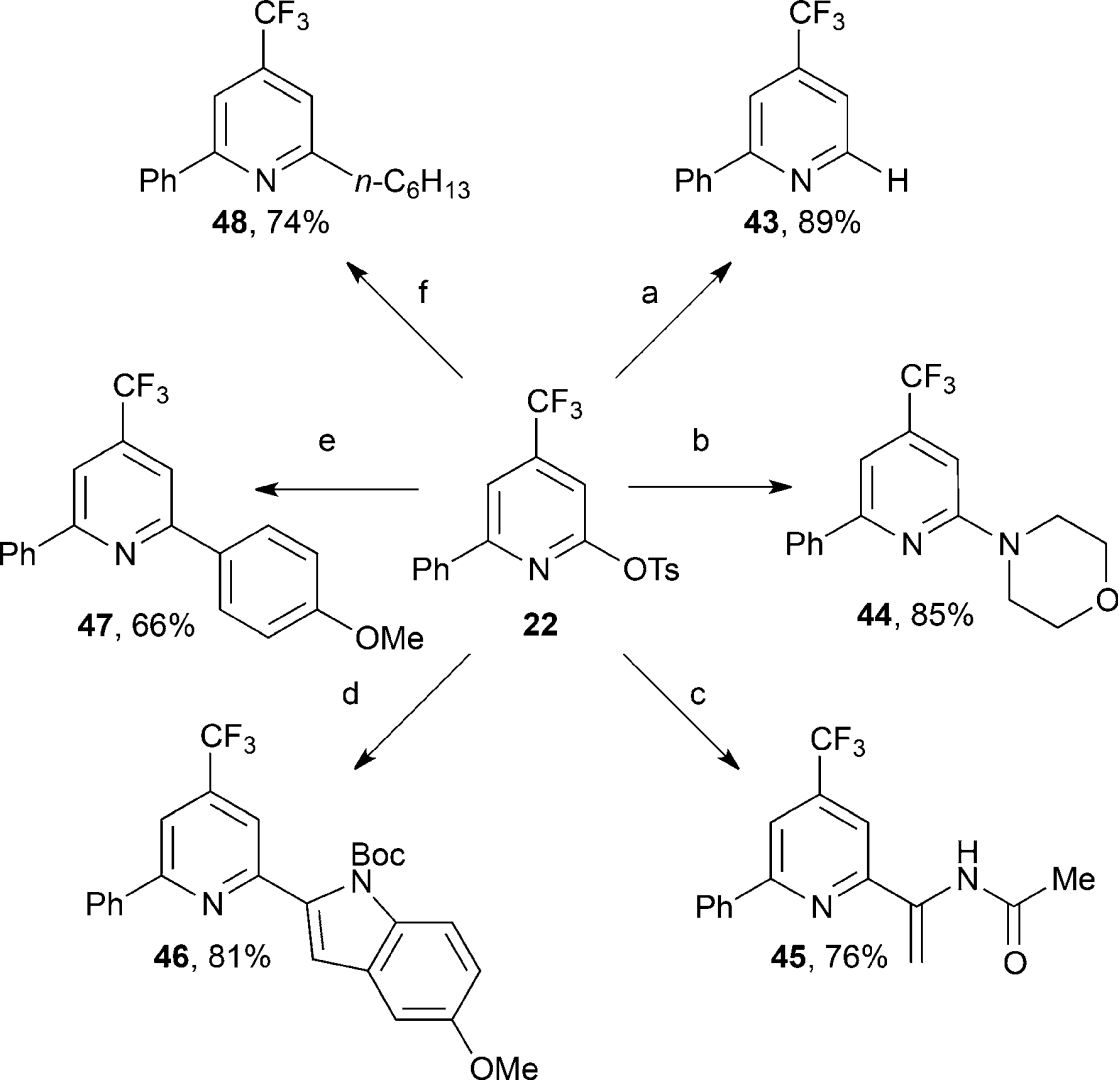
Product derivatizations. a) HCO_2_H (3 equiv), Pd(OAc)_2_ (5 mol %), dppp (5 mol %), Et_3_N (5 equiv) DMF, 60 °C, 1 h. b) morpholine (10 eq), Et_3_N (2 equiv), toluene, 110 °C, 16 h. c) *N*-vinylacetamide (4 equiv), [Pd(dba)_2_] (5 mol %), dppf (5 mol %), Cy_2_NMe (3 equiv), 1,4-dioxane, 100 °C, 16 h. d) ArB(OH)_2_ (2 equiv), Pd(OAc)_2_ (2 mol %), BrettPhos (2 mol %), K_3_PO_4_⋅H_2_O (3 equiv), toluene, 110 °C, 2 h. e) 4-MeOC_6_H_4_MgBr, (1.5 equiv), [Pd(dba)_2_] (2.5 mol %), PinP(O)H (5 mol %), 1,4-dioxane, 80 °C, 24 h. f) *n*-C_6_H_13_MgBr (1.5 equiv), FeCl_3_ (5 mol %), NMP (9 equiv), THF, −10 °C, 10 min. Boc=*tert*-butoxycarbonyl, Cy=cyclohexyl, dba=dibenzylideneacetone, dppp=1,3-bis(diphenylphosphino)propane, NMP=*N*-methyl-2-pyrrolidone, Pin=pinacol, THF=tetrahydrofuran.

Finally, the utility of this approach for the synthesis of medicinally relevant pyridine-containing molecules was illustrated. For example, the pyridine **50**, which possesses activity as a COX-2 inhibitor (<0.5 mm activity, >100 fold selectivity for COX-2 versus COX-1)^29^ for the treatment of depressive disorders, can be made in 49 % yield over two synthetic steps from the α,β-unsaturated ketimine **49** (14 % overall yield from commercially available starting materials, Scheme 4).

**Scheme 4:**
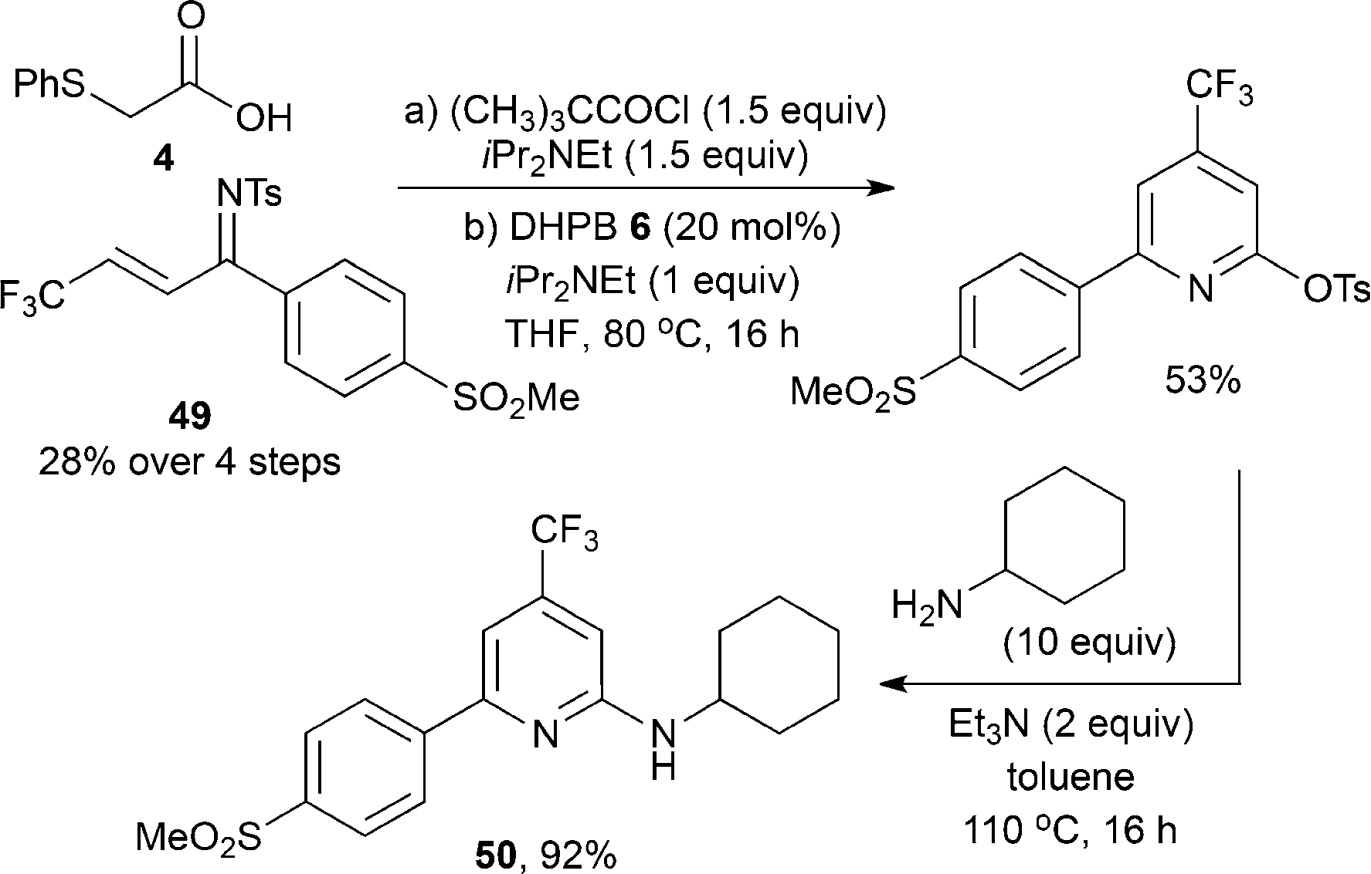
Rapid assembly of the biologically active pyridine **50**.

In conclusion, we have developed an isothiourea-catalyzed, one-pot synthesis of 2,4,6-substituted pyridines bearing a readily derivatized 2-sulfonate functionality from (phenylthio)acetic acid and range of α,β-unsaturated ketimines. This process proceeds by intermolecular Michael addition/lactamization, thiophenyl elimination, and N- to O-sulfonyl migration, wherein the N-sulfonyl activating group within the α,β-unsaturated ketimine is transformed into a valuable 2-sulfonate functional handle in the resulting pyridine. Functionalization of this group allows the rapid assembly of both novel and biologically relevant pyridines. Current research from this laboratory is directed towards developing new applications of isothioureas in catalysis.

## Supplementary material

As a service to our authors and readers, this journal provides supporting information supplied by the authors. Such materials are peer reviewed and may be re-organized for online delivery, but are not copy-edited or typeset. Technical support issues arising from supporting information (other than missing files) should be addressed to the authors.
