# Supplementary material for: Isothiourea-Mediated One-Pot Synthesis of Functionalized Pyridines
Source: Angew Chem Int Ed Engl. 2013 Sep 17;52(44):11642–6. doi: 10.1002/anie.201306786 (PMC4065352; doi:10.1002/anie.201306786)
Supplement: Supplementary file 1 [file anie0052-11642-SD1.pdf]

Angewandte Chemie
Eine Zeitschrift der Gesellschaft Deutscher Chemiker

Supporting Information
© Wiley-VCH 2013
69451 Weinheim, Germany

Isothiourea-Mediated One-Pot Synthesis of Functionalized Pyridines**

Daniel G. Stark, Louis C. Morrill, Pei-Pei Yeh, Alexandra M. Z. Slawin,
*Timothy J. C. O'Riordan, and Andrew D. Smith**

anie_201306786_sm_miscellaneous_information.pdf

SUPPORTING INFORMATION

Contents

1.1 General Information S2

1.2 General Experimental Procedures S3

1.3 Reaction Optimisation S5

1.4 Synthesis of Starting Materials S6

1.5 Examples – Ketimine Variation S28

1.6 Product Derivatisations S43

1.7 Sulfonyl *N*- to *O*-migration Studies S49

1.8 Other Michael-Acceptors Investigated S49

1.9 References and Notes S53

1.10 Appendix I: ^{1}H and ^{13}C NMR Spectra for Novel Compounds S54

1.1 General information

Reactions involving moisture sensitive reagents were carried out under a nitrogen atmosphere using standard vacuum line techniques in addition to freshly distilled solvents. All glassware used was flame dried and cooled under vacuum.

Anhydrous solvents (THF, CH_2Cl_2, toluene, and Et_2O) were dried and purified by an alumina column (Mbraun SPS-800). Petrol is defined as petroleum ether 40-60 °C. All other solvents and commercial reagents were used as supplied without further purification unless stated otherwise.

Room temperature (rt) refers to 20-25 °C. Temperatures of 0 °C and −78 °C were obtained using ice/water and CO_2(s)/acetone baths respectively. Temperatures of 0 °C to −50 °C for overnight reactions were obtained using an immersion cooler (HAAKE EK 90). Reflux conditions were obtained using an oil bath equipped with a contact thermometer. *In vacuo* refers to the use of a Büchi Rotavapor R-2000 rotary evaporator with a Vacubrand CVC_2 vacuum controller or a Heidolph Laborota 4001 rotary evaporator with a vacuum controller.

Analytical thin layer chromatography was performed on pre-coated aluminium plates (Kieselgel 60 F_{254} silica). TLC visualisation was carried out with ultraviolet light (254 nm), followed by staining with a 1% aqueous $KMnO_4$ solution. Flash column chromatography was performed on Kieselgel 60 silica in the solvent system stated.

^{1}H and ^{13}C nuclear magnetic resonance (NMR) spectra were acquired on either a Bruker Avance 300 (300 MHz, ^{1}H, 75 MHz ^{13}C), Bruker Avance II 400 (400 MHz, ^{1}H, 100 MHz ^{13}C) or a Bruker Avance II 500 (500 MHz, ^{1}H, 125 MHz ^{13}C) spectrometer at ambient temperature in the deuterated solvent stated. All chemical shifts are quoted in parts per million (ppm) relative to the residual solvent as the internal standard. All coupling constants, *J*, are quoted in Hz. Multiplicities are indicated by: s (singlet), d (doublet), t (triplet), q (quartet), ABq (AB quartet), sept (septet), oct (octet), m (multiplet), dd (doublet of doublets), ddd (doublet of doublet of doublets, dt (doublet of triplets) and td (triplet of doublets). The abbreviation Ar is used to denote aromatic, py to denote pyridine ring, Np to denote naphthalene substituent, br. to denote broad and app. to denote apparent.

Infrared spectra (ν_{max}) Infrared spectra were recorded on a Shimadzu Fourier transform IR Affinity-1 Infrared spectrophotometer using the MIRacle^{TM} single reflection horizontal ATR accessory from Pike (ZnSe single crystal). Only the characteristic peaks are quoted. Samples were directly placed on the crystal (ATR). Melting points were recorded on an Electrothermal apparatus.

Mass spectrometry (*m/z*) data were acquired by electrospray ionisation (ES), electron impact (EI) or nanospray ionisation (NSI) either at the University of St Andrews or the EPSRC National Mass Spectrometry Service Centre, Swansea. At the University of St Andrews, low

and high resolution ESI MS were carried out on a Micromass LCT spectrometer. At the EPSRC National Mass Spectrometry Service Centre, low resolution NSI MS was carried out on a Micromass Quattro II spectrometer and high resolution NSI MS on a Thermofisher LTQ Orbitrap XL spectrometer.

1.2 General Experimental Procedures

General Procedure A*:* *Preparation of phosphoranes*
To a solution of requisite bromide (1 equiv.) in CH_2Cl_2 (0.5 M) at rt was added triphenylphosphine (1 equiv.) and the solution was allowed to stir for 4 h before being concentrated *in vacuo*. The residue was dissolved in CH_2Cl_2:H_2O (40:60) and 2 M NaOH (2 equiv.) was added. The reaction mixture was stirred at rt for 1 h before being extracted with CH_2Cl_2 (× 3). The combined organic fraction were washed with brine, dried ($MgSO_4$), filtered and concentrated *in vacuo* to give the crude reaction mixture. Products were purified by recrystallisation with specified solvent or by column chromatography.

General Procedure B*:* *Preparation of keto esters*
Keto esters prepared as per literature procedure.[1] To a solution of requisite tartrate (1 equiv.) in Et_2O (0.45 M) at rt was added periodic acid (1 equiv.) and the solution was allowed to stir for 1 h. The reaction mixture was filtered and the solids washed with THF. The organic fraction was dried ($MgSO_4$) and filtered. The requisite phosphorane (1.5 equiv.) was added and the reaction mixture was stirred at rt for 2 h before being concentrated *in vacuo* to give the crude reaction mixture. Products purified by column chromatography.

General Procedure C*:* *Preparation of trifluoromethyl enones*
Trifluoromethyl enones prepared as per literature procedure.[2] To a solution of *i*-Pr_2NH (2.2 equiv.) in THF at −78 °C was added *n*-BuLi (2.2 equiv.) and the solution was allowed to stir for 20 mins. A pre-cooled (−78 °C) solution of 2-bromo-3,3,3-trifluoroprop-1-ene (1 equiv.) in THF was added dropwise at −78 °C followed by a further 5 mins of stirring. The desired aldehyde (1.2 equiv.) was added dropwise followed by stirring at −78 °C for 30 minutes. The reaction mixture was quenched by addition of HCl (1 M in H_2O) and allowed to warm to rt. The reaction mixture was extracted with EtOAc (× 3) and the combined organics were dried ($MgSO_4$), filtered and concentrated *in vacuo* to give the crude intermediate propargyl alcohol. The resulting oil was dissolved in THF before Et_3N (4 equiv.) was added before the reaction mixture was heated at reflux for 16 h. Once cool the reaction mixture was quenched by addition of HCl (1 M in H_2O) and the reaction mixture was extracted with EtOAc (× 3). The

combined organics were dried ($MgSO_4$), filtered and concentrated *in vacuo* to give the crude reaction mixture. Products purified by column chromatography.

General Procedure D: *Preparation of α,β-unsaturated ketimines*

To a solution of the requisite enone (1 equiv.) and requisite sulfonamide (1 equiv.) in CH_2Cl_2 (0.2 M) at 0 °C was added Et_3N (2 equiv.) followed by $TiCl_4$ (1 equiv.) and the reaction mixture was allowed to stir at rt for 16 h. The reaction mixture was quenched by addition of H_2O and extracted with EtOAc (× 3). The combined organic extracts were dried ($MgSO_4$), filtered and concentrated *in vacuo* to give the crude reaction mixture.

Generally ketimines within the ester series proved troublesome to purify. These were judged to be >90% pure by ^{1}H NMR and suitable for use in this protocol. Full characterisation data for ketimine **89** has been provided as a representative example for the ketimines used as crude. It should be noted that these issues were not encountered with ketimines from the trifluoromethyl series.

General Procedure E: *Pyridine synthesis*

To a solution of (phenylthio)acetic acid (1 equiv.) in THF (0.06 M of acid) were added *i*-Pr_2NEt (1.5 equiv.) and pivaloyl chloride (1.5 equiv.) at 0 °C. The reaction mixture was allowed to stir at 0 °C for 10 minutes. The requisite Michael acceptor (1 equiv.), DHPB (0.2 equiv.), and *i*-Pr_2NEt (1 equiv.) were then added at rt. The reaction mixture was stirred at rt until total consumption of the Michael acceptor as judged by TLC analysis. The reaction mixture was then heated at reflux for the required time for complete conversion to the pyridine. The reaction mixture was subsequently quenched by addition of H_2O and extracted with CH_2Cl_2 (× 3). The combined organics were dried ($MgSO_4$), filtered and concentrated *in vacuo* to give the crude reaction mixture. Products were purified by column chromatography.

1.3 Reaction Optimisation

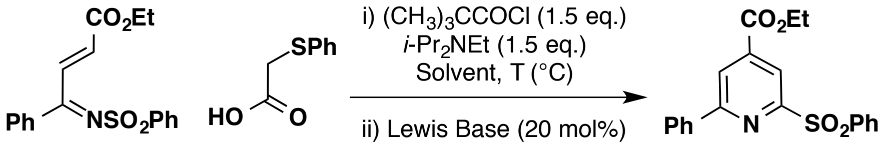

5 **4** **7**

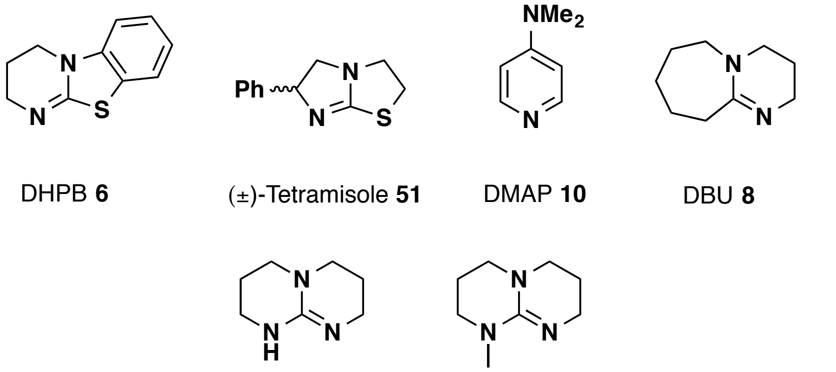

DHPB **6** (±)-Tetramisole **51** DMAP **10** DBU **8**

TBD **9** MTBD **52**

Entry	Lewis Base	Solvent	T (°C)	Time (h)	Yield (%)[a]
**1**	DHPB	CH_2Cl_2	0	4	7
**2**	DHPB	CH_2Cl_2	rt	4	30
**3**	(±)-Tetramisole	CH_2Cl_2	rt	4	-
**4**	DBU	CH_2Cl_2	rt	4	13
**5**	TBD	CH_2Cl_2	rt	4	-
**6**	MTBD	CH_2Cl_2	rt	4	-
**7**	DMAP	CH_2Cl_2	rt	4	4
**8**	DHPB	THF	80	4	49
**9**	(±)-Tetramisole	THF	80	4	26
**10**	DBU	THF	80	4	-
**11**	TBD	THF	80	4	-
**12**	MTBD	THF	80	4	-
**13**	DMAP	THF	80	4	36
**14**	DHPB	THF	80	16	67
**15**	DHPB	THF[b]	80	16	10
**16**	DHPB	THF	80[c]	2	52
**17**	DHPB	1,4-dioxane	80	16	52
**18**	DHPB	EtOAc	80	16	50
**19**	DHPB	MeCN	80	16	Nd
**20**	DHPB	PhMe	80	16	Nd
**21**	DHPB	DCE	80	16	Nd

**22**	DHPB	THF	80	48	59
**23**	DHPB	THF	120	16	35

[a] Isolated yield of **7** following chromatography; [b] 0.006 M in ketimine (typically 0.06 M); [c] Biotage Initiator with a program of heating to 80 °C at maximum power of 150 W; Nd = Not determined.

1.4 Preparation of Starting Materials

1-Phenyl-2-(triphenylphosphoranylidene)ethanone[3]

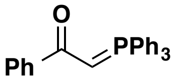

53

Following general procedure **A**, 2-bromo-1-phenylethanone (10.0 g, 50.2 mmol) and triphenylphosphine (13.2 g, 50.2 mmol) in CH_2Cl_2 (100 mL) followed by NaOH (2 M in H_2O, 50 mL, 100 mmol) in CH_2Cl_2 (60 mL) and H_2O (90 mL) gave, after recrystallisation (EtOH:Et_2O), phosphorane **53** as a white solid (13.0 g, 68%); mp 174-176 °C; {lit.[3] mp 173-175 °C}; δ_H (500 MHz, $CDCl_3$) 4.46 (1 H, d, *J* 24.5, C(2)*H*), 7.38-7.39 (3 H, m, C(1)Ar(3,5)*H* and C(1)Ar(4)*H*), 7.48-7.52 (6 H, m, 3 PAr(3,5)*H*), 7.57-7.60 (3 H, m, 3 PAr(4)*H*), 7.73-7.77 (6H, m, 3 PAr(2,6)*H*), 7.99-8.01 (2 H, m, C(1)Ar(2,6)*H*). All data in accordance with literature.[3]

1-(*p*-Tolyl)-2-(triphenylphosphoranylidene)ethanone[3]

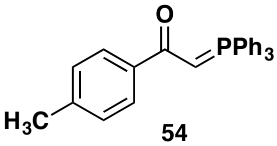

54

Following general procedure **A**, 2-bromo-1-(*p*-tolyl)ethanone (5.33 g, 25.0 mmol) and triphenylphosphine (6.57 g, 25.0 mmol) in CH_2Cl_2 (50 mL) followed by NaOH (2 M in H_2O, 25 mL, 50 mmol) in CH_2Cl_2 (30 mL) and H_2O (45 mL) gave, after recrystallisation (EtOH:petrol), phosphorane **54** as a white solid (4.16 g, 42%); mp 174-176 °C; {lit.[3] mp 168-169 °C}; δ_H (400 MHz, $CDCl_3$) 2.39 (3 H, s, CH_3), 4.42 (1 H, d, *J* 24.8, C(2)*H*), 7.18 (2 H, d, *J* 8.0, C(1)Ar(3,5)*H*), 7.47-7.51 (6 H, m, 3 PAr(3,5)*H*), 7.55-7.60 (3 H, m, 3 PAr(4)*H*), 7.72-7.77 (6 H, m, 3 PAr(2,6)*H*), 7.90 (2 H, d, *J* 8.1, C(1)Ar(2,6)*H*). All data in accordance with literature.[3]

1-(4-Nitrophenyl)-2-(triphenylphosphoranylidene)ethanone[4]

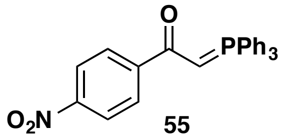

55

Following general procedure **A**, 2-bromo-1-(4-nitrophenyl)ethanone (6.10 g, 25.0 mmol) and triphenylphosphine (6.57 g, 25.0 mmol) in CH_2Cl_2 (50 mL) followed by NaOH (2 M in H_2O, 25 mL, 50 mmol) in CH_2Cl_2 (30 mL) and H_2O (45 mL) gave, after recrystallisation (EtOH:Et_2O), phosphorane **55** as a yellow solid (5.98 g, 56%); mp 158-160 °C; {lit.[4] mp 159-160 °C}; δ_H (400 MHz, $CDCl_3$) 4.52 (1 H, d, *J* 23.0, C(2)*H*), 7.50-7.55 (6 H, m, 3 PAr(3,5)*H*), 7.60-7.64 (3 H, m, 3 PAr(4)*H*), 7.70-7.76 (6 H, m, 3 PAr(2,6)*H*), 8.09-8.11 (2 H, m, C(1)Ar(2,6)*H*), 8.21-8.23 (2 H, m, C(1)Ar(3,5)*H*). All data in accordance with literature.[4]

1-(4-Methoxyphenyl)-2-(triphenylphosphoranylidene)ethanone[5]

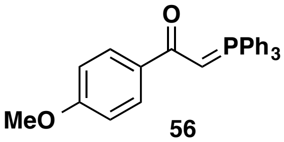

56

Following general procedure **A**, 2-bromo-1-(4-methoxyphenyl)ethanone (5.73 g, 25.0 mmol) and triphenylphosphine (6.57 g, 25.0 mmol) in CH_2Cl_2 (50 mL) followed by NaOH (2 M in H_2O, 25 mL, 50 mmol) in CH_2Cl_2 (30 mL) and H_2O (45 mL) gave, after recrystallisation (EtOH:Et_2O), phosphorane **56** as a white solid (7.00 g, 68%); mp 156-158 °C; {lit.[5] mp 159-161 °C}; δ_H (400 MHz, $CDCl_3$) 3.85 (3 H, s, OCH_3), 4.37 (1 H, d, *J* 24.6, C(2)*H*), 6.87-6.91 (2 H, m, C(1)Ar(3,5)*H*), 7.47-7.51 (6 H, m, 3 PAr(3,5)*H*), 7.55-7.60 (3 H, m, 3 PAr(4)*H*), 7.71-7.77 (6 H, m, 3 PAr(2,6)*H*), 7.95-7.97 (2 H, m, C(1)Ar(2,6)*H*). All data in accordance with literature.[5]

4-[2-(Triphenylphosphanylidene)acetyl]benzonitrile[5]

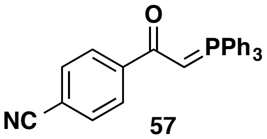

57

Following general procedure **A**, 2-(2-bromoacetyl)benzonitrile (5.00 g, 22.3 mmol) and triphenylphosphine (5.86 g, 22.3 mmol) in CH_2Cl_2 (50 mL) followed by NaOH (2 M in H_2O, 22 mL, 100 mmol) in CH_2Cl_2 (30 mL) and H_2O (45 mL) gave, after recrystallisation (EtOH:Et_2O), phosphorane **57** as a white solid (7.30 g, 81%); mp 199-201 °C;[6] δ_H (300 MHz, $CDCl_3$) 4.46 (1 H, d, *J* 23.1, C(2)*H*), 7.46-7.52 (8 H, m, Ar*H*), 7.59-7.73 (9 H, m, Ar*H*), 8.02 (2 H, d, *J* 8.42, Ar(2,6)*H*). All spectrascopic data in accordance with literature.[5]

1-(4-Chlorophenyl)-2-(triphenylphosphoranylidene)ethanone[3]

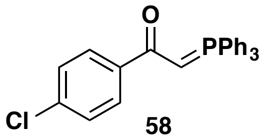

58

Following general procedure **A**, 2-bromo-1-(4-chlorophenyl)ethanone (5.84 g, 25.0 mmol) and triphenylphosphine (6.57 g, 25.0 mmol) in CH_2Cl_2 (50 mL) followed by NaOH (2 M in H_2O, 25 mL, 50 mmol) in CH_2Cl_2 (30 mL) and H_2O (45 mL) gave, after recrystallisation (EtOH:petrol), phosphorane **58** as a white solid (6.83 g, 66%); mp 186-188 °C; {lit.[3] mp 194-196 °C}; δ_H (400 MHz, $CDCl_3$) 4.41 (1 H, d, *J* 24.0, C(2)*H*), 7.31-7.35 (2 H, m, C(1)Ar(3,5)*H*), 7.48-7.52 (6 H, m, 3 PAr(3,5)*H*), 7.57-7.62 (3 H, m, 3 PAr(4)*H*), 7.70-7.76 (6 H, m, 3 PAr(2,6)*H*), 7.90-7.94 (2 H, m, C(1)Ar(2,6)*H*). All data in accordance with literature.[3]

1-(2-Fluorophenyl)-2-(triphenylphosphoranylidene)ethanone

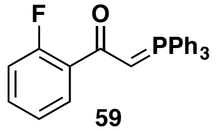

59

Following general procedure **A**, 2-bromo-1-(2-fluorophenyl)ethanone (5.00 g, 23.0 mmol) and triphenylphosphine (6.06 g, 23.0 mmol) in CH_2Cl_2 (50 mL) followed by NaOH (2 M in H_2O, 25 mL, 50 mmol) in CH_2Cl_2 (30 mL) and H_2O (45 mL) gave, after chromatographic purification (eluent CH_2Cl_2 100% to EtOAc 100%), phosphorane **59** as a yellow oil (6.96 g, 76%); δ_H (500 MHz, $CDCl_3$) 4.59 (1 H, d, *J* 25.9, C(2)*H*), 7.06 (1 H, ddd, *J* 11.2, 8.2, 1.1, C(1)Ar(3)*H*), 7.16 (1 H, td, *J* 7.5, 1.1, C(1)Ar(5)*H*), 7.28-7.33 (1 H, m, C(1)Ar(6)*H*), 7.47-7.52 (6 H, m, 3 PAr(3,5)*H*), 7.55-7.60 (3 H, m, 3 PAr(4)*H*), 7.73-7.78 (6 H, m, 3 PAr(2,6)*H*), 7.98 (1 H, td, *J* 7.8, 1.9, C(1)Ar(4)*H*). This compound was approximately 83% pure by ^{1}H NMR (17% triphenylphosphine) and taken on without full characterisation.

1-(Naphthalene-2-yl)-2-(triphenylphosphanylidene)ethan-1-one[4]

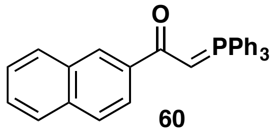

60

Following general procedure **A**, 2-(bromoacetyl)naphthalane (6.23 g, 25.0 mmol) and triphenylphosphine (6.57 g, 25.0 mmol) in CH_2Cl_2 (50 mL) followed by NaOH (2 M in H_2O, 25 mL, 50.0 mmol) in CH_2Cl_2 (30 mL) and H_2O (45 mL) gave, after recrystallisation (EtOAc:Petrol), phosphorane **60** as a white solid (8.71 g, 66%); mp 192-194 °C; {lit.[4] mp 190-192 °C}; δ_H (300 MHz, $CDCl_3$) 4.59 (1 H, d, *J* 24.4, C(2)*H*), 7.42-7.58 (11 H, m, Ar*H*),

7.68-7.82 (9 H, m, Ar*H*), 8.07 (1 H, dd, *J* 8.6, 1.7, Np(9)*H*), 8.49 (1 H, s, Np(1)*H*). All data in accordance with literature.[4]

(*E*)-Benzyl 4-oxo-4-phenylbut-2-enoate[1]

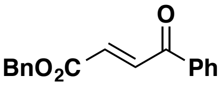

61

Following general procedure **B**, dibenzyl tartrate (3.00 g, 9.07 mmol) and periodic acid (2.07 g, 9.07 mmol) in Et_2O (20 mL), phosphorane **53** (5.17 g, 13.6 mmol) in THF (25 mL) gave, after chromatographic purification (eluent Et_2O:petrol 15:85), keto ester **61** as a light yellow oil (2.90 g, 80%); δ_H (500 MHz, $CDCl_3$) 5.31 (2H, s, CH_2), 6.96 (1 H, d, *J* 15.6, C(2)*H*), 7.37-7.45 (5 H, m, Ar*H*), 7.54 (2 H, t, *J* 7.7, C(4)Ar(3,5)*H*), 7.65 (1 H, t, *J* 7.4, C(4)Ar(4)*H*), 7.97 (1 H, d, *J* 15.6, C(3)*H*), 8.01-8.03 (2 H, m, C(4)Ar(2,6)*H*). All data in accordance with literature.[1]

(*E*)-Benzyl 4-oxo-4-(*p*-tolyl)but-2-enoate

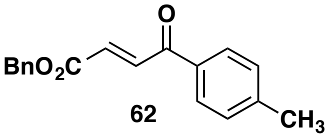

62

Following general procedure **B**, dibenzyl tartrate (3.00 g, 9.07 mmol) and periodic acid (2.07 g, 9.07 mmol) in Et_2O (20 mL), phosphorane **54** (5.36 g, 13.6 mmol) in THF (25 mL) gave, after chromatographic purification (eluent Et_2O:petrol 15:85), keto ester **62** as a yellow solid (2.65 g, 70%); mp 40-42 °C; ν_{max} (ATR)/cm^{-1} 1601, 1668 (Enone C=O), 1713 (Ester C=O), 2856, 3070 (C-H); δ_H (300 MHz, $CDCl_3$) 2.47 (3 H, s, CH_3), 5.32 (2 H, s, CH_2), 6.97 (1 H, d, *J* 15.5, C(2)*H*), 7.34 (2 H, d, *J* 8.4, C(4)Ar(3,5)*H*), 7.39-7.47 (5 H, m, Ar*H*), 7.93-8.01 (3 H, m, C(4)Ar(2,6)*H* and C(3)*H*); δ_C (75 MHz, $CDCl_3$) 21.8 (CH_3), 67.1 (CH_2), 128.4 (*ArC*), 128.5 (*ArC*), 128.7 (*ArC*), 129.1 (C(4)*ArC*(2,6)), 129.6 (C(4)*ArC*(3,5)), 131.8 (*C*(2)), 134.2 (C(4)*ArC*(1)), 135.4 (CH_2*ArC*(1)), 137.1 (*C*(3)), 145.0 (C(4)*ArC*(4)), 165.5 (*C*(1)), 188.9 (*C*(4)); *m/z* (NSI$^+$) 281 ([M+H]$^+$, 100%); HRMS (NSI$^+$) $C_{18}H_{17}O_3^+$ ([M+H]$^+$) requires 281.1172; found 281.1171 (−0.4 ppm).

(*E*)-Benzyl 4-(4-nitrophenyl)-4-oxobut-2-enoate

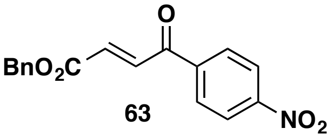

63

Following general procedure **B**, dibenzyl tartrate (3.00 g, 9.07 mmol) and periodic acid (2.07 g, 9.07 mmol) in Et_2O (20 mL), phosphorane **55** (5.78 g, 13.6 mmol) in THF (25 mL) gave,

after chromatographic purification (eluent Et_2O:petrol 50:50), keto ester **63** as a yellow solid (3.27 g, 77%); mp 92-94 °C; ν_{max} (ATR)/cm^{-1} 1601, 1668 (Enone C=O), 1709 (Ester C=O), 2859, 3076 (C-H); δ_H (300 MHz, $CDCl_3$) 5.33 (2 H, s, C*H*$_2$), 7.01 (1 H, d, *J* 15.6, C(2)*H*), 7.38-7.46 (5 H, m, Ar*H*), 7.93 (1 H, d, *J* 15.6, C(3)*H*), 8.15-8.18 (2 H, m, C(4)Ar(2,6)*H*), 8.37-8.40 (2 H, m, C(4)Ar(3,5)*H*); δ_C (75 MHz, $CDCl_3$) 67.5 (*C*H_2), 124.1 (C(4)*ArC*(3,5), 128.5 (*ArC*), 128.7 (*ArC*), 128.8 (*ArC*), 129.9 (C(4)*ArC*(2,6)), 133.9 (*C*(2)), 135.1 (CH_2*ArC*(1)), 135.7 (*C*(3)), 141.0 (C(4)*ArC*(4)), 150.7 (C(4)*ArC*(1)), 164.9 (*C*(1)), 188.1 (*C*(4)); *m/z* (NSI^+) 312 ($[M+H]^+$, 19%); HRMS (NSI^+) $C_{17}H_{14}NO_5^+$ ($[M+H]^+$) requires 312.0866; found 312.0871 (+1.4 ppm).

(*E*)-Benzyl 4-(4-methoxyphenyl)-4-oxobut-2-enoate

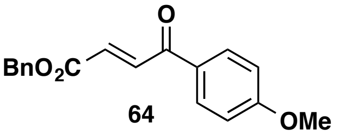

64

Following general procedure **B**, dibenzyl tartrate (3.00 g, 9.07 mmol) and periodic acid (2.07 g, 9.07 mmol) in Et_2O (20 mL), phosphorane **56** (5.58 g, 13.6 mmol) in THF (25 mL) gave, after chromatographic purification (eluent Et_2O:petrol 30:70), keto ester **64** as a yellow solid (3.67 g, 91%); mp 74-76 °C; ν_{max} (ATR)/cm^{-1} 1591, 1663 (Enone C=O), 1707 (Ester C=O), 2847, 3073 (C-H); δ_H (400 MHz, $CDCl_3$) 3.90 (3 H, s, OC*H*$_3$), 5.30 (2 H, s, *C*H_2), 6.94 (1 H, d, *J* 15.5, C(2)*H*), 6.97-7.01 (2 H, m, C(4)Ar(3,5)*H*), 7.36-7.45 (5 H, m, Ar*H*), 7.97 (1 H, d, *J* 15.5, C(3)*H*), 7.99-8.03 (2 H, m, C(4)Ar(2,6)*H*); δ_C (100 MHz, $CDCl_3$) 55.6 (O*C*H_3), 67.1 (*C*H_2), 114.2 (C(4)*ArC*(3,5), 128.4 (*ArC*), 128.5 (*ArC*), 128.7 (*ArC*), 129.7 (C(4)*ArC*(1)), 131.4 (C(4)*ArC*(2,6)), 131.5 (*C*(2)H), 135.4 (CH_2*ArC*(1)), 137.1 (*C*(3)H), 164.3 (O*C*H_3), 165.6 (*C*(1)), 187.5 (*C*(4)); *m/z* (NSI^+) 297 ($[M+H]^+$, 100%); HRMS (NSI^+) $C_{18}H_{17}O_4^+$ ($[M+H]^+$) requires 297.1121; found 297.1121 (−0.1 ppm).

Methyl (2*E*)-4-oxo-4-phenylbut-2-enoate[1]

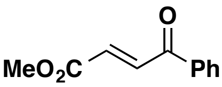

65

Following general procedure **B**, dimethyl tartrate (4.69 g, 26.3 mmol) and periodic acid (5.99 g, 26.3 mmol) in Et_2O (50 mL), phosphorane **53** (15.0 g, 39.4 mmol) in THF (60 mL) gave, after chromatographic purification (eluent Et_2O:petrol 5:95), keto ester **65** as a light yellow oil (4.8 g, 64%); δ_H (500 MHz, $CDCl_3$) 3.82 (3 H, s, OC*H*$_3$), 6.87 (1 H, d, *J* 15.6, C(2)*H*), 7.47-7.50 (2 H, app. m, Ar(3,5)*H*), 7.58-7.61 (1 H, app. m, Ar(4)*H*), 7.90 (1 H, d, *J* 15.6, C(3)*H*), 7.98 (2 H, d, *J* 7.60, Ar(2,6)*H*).All data in accordance with literature.[1]

Methyl (2*E*)-4-(4-cyanophenyl)-4-oxobut-2-enoate

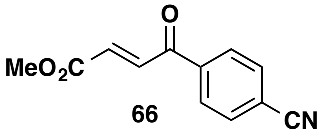

66

Following general procedure **B**, dimethyl tartrate (2.05 g, 11.5 mmol) and periodic acid (2.62 g, 11.5 mmol) in Et_2O (30 mL), phosphorane **57** (7.0 g, 17.3 mmol) in THF (35 mL) gave, after chromatographic purification (eluent EtOAc:Petrol 30:70), keto ester **66** as a light green solid (2.2 g, 59%); mp 168-170 °C; ν_{max} (ATR)/cm^{-1} 1629, 1668 (enone C=O), 1720 (ester C=O), 2231 (CN), 3086 (C-H); δ_H (500 MHz, $CDCl_3$) 3.86 (3 H, s, C*H*$_3$), 6.94 (1 H, d, *J* 15.5, C(2)*H*), 7.83 (2 H, *J* 8.5, Ar(3,5)*H*), 7.87 (1 H, d, *J* 15.5, C(3)*H*), 8.08 (2 H, d, *J* 8.5, Ar(2,6)*H*); δ_C (125 MHz, $CDCl_3$) 52.6 (*C*H$_3$), 117.1 (*C*N), 117.7 (*ArC*(4)), 129.2 (*ArC*(2,6)H), 132.8 (*ArC*(3,5)), 133.6 (*C*(2)H), 135.3 (*C*(3)H), 139.5 (*ArC*(1)), 165.6 (*C*(4)), 188.2 (*C*(1)); *m/z* (NSI$^+$) 233 ([M+NH$_4$], 24%), HRMS (NSI$^+$) $C_{12}H_{13}N_2O_3^+$ ([M+NH]$^+$) requires 233.0921 found 233.0922 (+0.6 ppm)

Methyl (2*E*)-4-oxo-4-[4-(trifluoromethyl)phenyl]but-2-enoate[7]

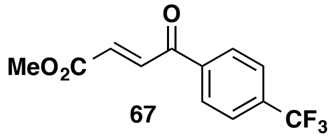

67

Following general procedure **B**, dimethyl tartrate (1.06 g, 5.95 mmol) and periodic acid (1.36 g, 5.95 mmol) in Et_2O (20 mL), 1-[4-(trifluoromethyl)phenyl]-2-(triphenylphosphanylidene)ethan-1-one[3] (4.0 g, 8.92 mmol) in THF (25 mL) gave, after chromatographic purification (eluent EtOAc:Petrol 15:85), keto ester **67** as a light yellow solid (2.12 g, 90%); mp 74-75 °C {lit.[7] 73.5-74.0 °C}; δ_H (300 MHz, $CDCl_3$) 3.86 (3 H, s, C*H*$_3$), 6.92 (1 H, d, *J* 15.6, C(3)*H*), 7.78 (2 H, Ar(3)*H*), 7.89 (1 H, d, *J* 15.6, C(2)*H*), 8.10 (2 H, d, *J* 8.8, Ar(2)*H*). All data in accordance with literature.[7]

(*E*)-Methyl 4-oxo-4-(4-chlorophenyl)but-2-enoate[8]

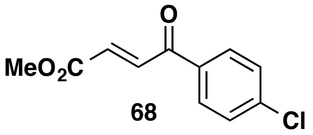

68

Following general procedure **b**, dimethyl tartrate (1.62 g, 9.07 mmol) and periodic acid (2.07 g, 9.07 mmol) in Et_2O (20 mL), phosphorane **58** (5.64 g, 13.6 mmol) in THF (25 mL) gave, after chromatographic purification (eluent Et_2O:petrol 15:85), keto ester **68** as a light yellow solid (2.34 g, 77%); mp 82-84 °C; {lit.[9] mp 81-82 °C}; δ_H (500 MHz, $CDCl_3$) 3.89 (3 H, s, OC*H*$_3$), 6.94 (1 H, d, *J* 15.5, C(2)*H*), 7.51-7.56 (2 H, m, Ar(3,5)*H*), 7.92 (1 H, d, *J* 15.5, C(3)*H*), 7.97-8.01 (2 H, m, Ar(2,6)*H*). All data in accordance with literature.[8]

(E)-Methyl 4-oxo-4-(2-fluorophenyl)but-2-enoate[7]

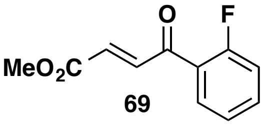

69

Following general procedure **B**, dimethyl tartrate (2.07 g, 11.7 mmol) and periodic acid (2.65 g, 11.7 mmol) in Et_2O (40 mL), phosphorane **59** (6.96 g, 17.5 mmol) in THF (50 mL) gave, after chromatographic purification (eluent Et_2O:petrol 20:80), keto ester **69** as a light yellow oil (2.96 g, 81%); δ_H (500 MHz, $CDCl_3$) 3.87 (3H, s, OCH_3), 6.87 (1 H, dd, J 15.6, 1.2, C(2)H), 7.20 (1 H, dd, J 10.9, 8.4, Ar(3)H), 7.28-7.31 (1 H, m, Ar(5)H), 7.58-7.63 (1 H, m, Ar(6)H), 7.79 (1 H, dd, J 15.6, 3.4 C(3)H), 7.85 (1 H, td, J 7.6, 1.8, Ar(4)H). All data in accordance with literature.[7]

Methyl (2E)-4-(naphthalene-2-yl)-4-oxobut-2-enoate

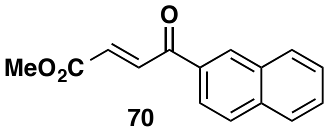

70

Following general procedure **B**, dimethyl tartrate (2.21 g, 12.4 mmol) and periodic acid (2.83 g, 12.4 mmol) in Et_2O (30 mL), phosphorane **60** (8.0 g, 18.6 mmol) in THF (35 mL) gave, after chromatographic purification (eluent EtOAc:Petrol 15:85), keto ester **70** as a light yellow solid (3.6 g, 81%); mp 113-115 °C; ν_{max} (ATR)/cm^{-1} 1624, 1666 (Enone C=O), 1724 (Ester C=O), 2956, 3067 (C-H); δ_H (300 MHz, $CDCl_3$) 3.88 (3 H, s, OCH_3), 6.97 (1 H, d, J 15.5, C(2)H), 7.62 (2H, ddd, J 10.1, 7.8, 1.31, NpH), 7.89-8.06 (4 H, m, NpH), 8.11 (1 H, d, J 15.5, C(3)H), 8.53 (1 H, s, Np(1)H); δ_C (125 MHz, $CDCl_3$) 52.4 (CH$_3$), 124.0 (*NpC*H), 127.1 (*NpC*H), 127.9 (*NpC*H), 129.0 (*NpC*H), 129.1 (*NpC*H), 129.8 (*NpC*H), 131.2 (*NpC*H), 132.0 (C(2)H), 132.4 (*NpC*), 134.0 (*NpC*), 135.9 (*NpC*), 136.6 (C(3)H), 166.2 (C(1)), 189.1 (C(4)); *m/z* (NSI$^+$) 241 ([M+H], 100%), HRMS (NSI$^+$) $C_{15}H_{13}O_3^+$ ([M+H]$^+$) requires 241.0859 found 241.0860 (+0.3 ppm).

Methyl (2E)-5,5-dimethyl-4-oxohex-2-enoate[10]

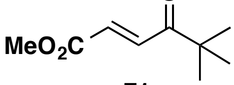

71

Following general procedure **B**, dimethyl tartrate (825 mg, 4.63 mmol) and periodic acid (1.06 g, 4.63 mmol) in Et_2O (20 mL), 3,3-dimethyl-1-(triphenylphosphanylidene)butan-2-one[3] (2.5 g, 6.94 mmol) in THF (25 mL) gave, after chromatographic purification (eluent EtOAc:Petrol, 5:95), keto ester **71** as a light yellow oil (1.0 g, 85%); δ_H (300 MHz, $CDCl_3$)

1.13 (9 H, s, (CH_3)$_3$), 3.74 (3 H, s, CH_3), 6.71 (1 H, d, *J* 15.5, C(3)*H*), 7.46 (1 H, d, *J* 15.5, C(2)*H*). All data in accordance with literature.[10]

(*E*)-4,4,4-Trifluoro-1-phenylbut-2-en-1-one[11] and (*Z*)-4,4,4-Trifluoro-1-phenylbut-2-en-1-one

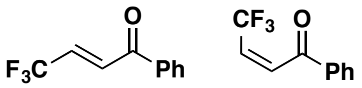

72 **73**

Following general procedure **C**, diisopropylamine (15.42 mL, 110 mmol) and *n*-BuLi (2.5 M in hexanes, 44.0 mL, 110 mmol) in THF (130 mL), 2-bromo-3,3,3-trifluoroprop-1-ene (5.30 mL, 50.0 mmol) in THF (90 mL) and benzaldehyde (6.10 mL, 60.0 mmol) gave the intermediate alcohol. Susequent treatement with Et_3N (27.9 mL, 200 mmol) in THF (280 mL) gave, a mixture of enones (*E:Z* 80:20).[12] Chromatographic purification (eluent Et_2O:petrol 5:95) gave:

(*E*)-enone **72** as a light yellow solid (7.22 g, 72% over 2 steps); mp 26-28 °C; {lit.[11] mp 28 °C}; δ_H (500 MHz, $CDCl_3$) 6.85 (1 H, dq, *J* 15.6, 6.6, C(3)*H*), 7.55-7.59 (3 H, m, C(2)*H* and Ar(3,5)*H*), 7.66-7.69 (1 H, m, Ar(4)*H*), 8.00-8.02 (2 H, m, Ar(2,6)*H*); δ_F (282 MHz, $CDCl_3$); −65.6 (CF_3); All data in accordance with literature.[11]

(*Z*)-enone **73** as a light yellow oil (1.72 g, 17% over 2 steps); δ_H (500 MHz, $CDCl_3$) 6.12 (1 H, dq, *J* 12.8, 7.9, C(3)*H*), 6.90 (1 H, d, *J* 12.8, C(2)*H*), 7.54 (2 H, t, *J* 7.8, Ar(3,5)*H*), 7.65-7.68 (1 H, m, Ar(4)*H*), 7.95-7.97 (2 H, m, Ar(2,6)*H*); δ_F (282 MHz, $CDCl_3$); −61.3 (CF_3).

(2*E*)-1-(3-Bromophenyl)-4,4,4-trifluorobut-2-en-1-one

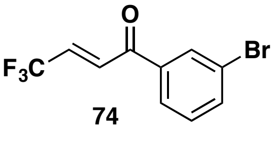

74

Following general procedure **C**, diisopropylamine (5.54 mL. 39.6 mmol) and *n*-BuLi (2.5 M in hexane, 15.8 mL, 39.6 mmol) in THF (50 mL), 2-bromo-3,3,3-trifluoroprop-1-ene (1.95 mL, 18.0 mmol) and 3-bromobenzaldehyde (2.52 mL, 21.6 mmol) gave intermediate alcohol. Subsequent treatment with Et_3N (10.0 mL, 71.7 mmol) in THF (100 mL) gave, after chromatographic purification (eluent; EtOAc:Petrol; 5:95), (*E*)-enone **74** as yellow solid (2.25 g, 45% over 2 steps); mp 30-32 °C; ν_{max} (ATR)/cm^{-1} 1125, 1651, 1686 (C=O), 3063 (ArH); δ_H (500 MHz, $CDCl_3$) 6.83 (1 H, dq, *J* 15.5, 6.67 C(3)*H*), 7.41 (1 H, t, Ar(3)*H*), 7.48 (1 H, dq, *J* 15.5, 1.71 C(2)*H*), 7.75 (1 H, ddd, *J* 8.11, 2.05, 1.06, Ar(4)*H*), 7.88 (1 H, d, *J* 8.4, Ar(2)*H*), 8.09 (1 H, s, Ar(6)*H*); δ_C (125 MHz, $CDCl_3$); 122.3 (q, *J* 271.0, *C*F_3), 123.4 (*ArC*(5)Br), 127.3 (*ArC*(4)H), 130.4 (*C*(2)H), 130.5 (*ArC*(3)H), 131 (q, *J* 35.9, *C*(3)H), 131.7 (*ArC*(6)H), 136.9 (*ArC*(2)H), 137.1 (*ArC*(1)), 186.6 (*C*(1)). *m/z* (NSI$^+$) 296 ([M+NH_4]$^+$,

100%); HRMS (NSI$^+$) $C_{10}H_{10}{}^{79}BrF_3NO^+$ ([M+NH$_4$]$^+$) requires 295.9892 found 295.9897 (+1.6 ppm).

(*E*)-1-(4-Bromophenyl)-4,4,4-trifluorobut-2-en-1-one[2]

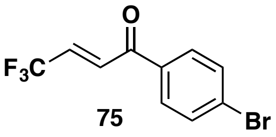

75

Following general procedure **C**, diisopropylamine (7.71 mL, 55.0 mmol) and *n*-BuLi (2.5 M in hexanes, 22.0 mL, 55.0 mmol) in THF (65 mL), 2-bromo-3,3,3-trifluoroprop-1-ene (2.65 mL, 25.0 mmol) in THF (45 mL) and 4-bromobenzaldehyde (5.55 g, 30.0 mmol) gave the intermediate alcohol. Subseqent treatment with Et_3N (13.9 mL, 100 mmol) in THF (140 mL) gave, after chromatographic purification (eluent Et_2O:petrol 3:97), (*E*)-enone **75** as a light yellow solid (4.62 g, 66% over 2 steps); mp 52-54 °C; {lit.[2] mp 51-52 °C}; δ_H (500 MHz, $CDCl_3$) 6.86 (1 H, dq, *J* 15.5, 6.6, C(3)*H*), 7.51 (1 H, dq, *J* 15.5, 2.0, C(2)*H*), 7.69-7.72 (2 H, m, Ar*H*), 7.86-7.88 (2 H, m, Ar*H*). All data in accordance with literature.[2]

(2*E*)-4,4,4-Trifluoro-1-(4-methylphenyl)but-2-en-1-one[13]

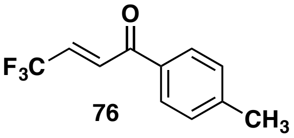

76

Following general procedure **C**, diisopropylamine (8.56 mL. 61.1 mmol) and *n*-BuLi (2.5 M in hexane, 24.4 mL, 61.1 mmol) in anhydrous THF (75 mL), 2-bromo-3,3,3-trifluoroprop-1-ene (3.0 mL, 27.8 mmol) and 4-tolualdehyde (3.93 mL, 33.3 mmol) gave intermediate alcohol. Subsequent treatment with Et_3N (13.3 mL, 96 mmol) in THF (100 mL) gave, after chromatographic purification (eluent; EtOAc:Petrol; 5:95) gave (*E*)-enone **76** as yellow solid (2.85 g, 42% over 2 steps); mp 58-60 °C {lit.[13] 56-59 °C}; δ_H (500 MHz, $CDCl_3$) 2.45 (3 H, s, Ar(4)C*H$_3$*), 6.81 (1 H, dq, *J* 15.6, 6.7 C(3)*H*), 7.32 (2 H, d, Ar(3)*H*), 7.53 (1 H, dq, *J* 15.6, 2.1 C(2)*H*), 7.88 (2 H, d, *J* 8.30, Ar(2)*H*). All data in accordance with literature.[13]

(2*E*)-4,4,4-Trifluoro-1-(4-fluorophenyl)but-2-en-1-one

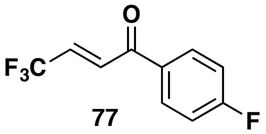

77

Following general procedure **C**, diisopropylamine (12.17 mL. 88.7 mmol) and *n*-BuLi (2.5 M in hexane, 35.5 mL, 88.7 mmol) in THF (90 mL), 2-bromo-3,3,3-trifluoroprop-1-ene (4.37 mL, 40.3 mmol) in THF (50 mL) and 4-fluorobenzaldehyde (5.19 mL, 48.3 mmol) gave

intermediate alcohol. Subsequent treatment with Et_3N (26 mL, 187 mmol) in THF (250 mL) gave, after chromatographic purification (eluent; EtOAc:Petrol; 5:95), (*E*)-enone **77** as yellow solid (3.12 g, 35% over 2 steps); mp 78-80 °C; ν_{max} (ATR)/cm^{-1} 1151, 1564, 1597 (C=O), 3086 (C-H) δ_H (400 MHz, $CDCl_3$) 6.82 (1 H, dq, *J* 15.5, 6.6 C(3)*H*), 7.20 (2 H, t, Ar(3)*H*), 7.51 (1 H, dq, *J* 15.5, C(2)*H*), 8.02 (2 H, m, Ar(2)*H*); δ_C (100 MHz, $CDCl_3$); 116.2 (d, *J* 23.8, *ArC*(3,5)H), 122.5 (q, *J* 272, *C*F_3) 130 (*C*(3)H), 130.3 (q, 35.6, *C*(2)H), 131.5 (d, *J* 9.92, *ArC*(2,6)H), 132 (*ArC*(1)), 166.4 (d, *J* 255.8, *ArC*(4)F), 186.3 (*C*(1)); *m/z* (APCI$^+$) 219 ([M+H], 100%); HRMS (APCI$^+$) $C_{10}H_7F_4O^+$ ([M+H]$^+$) requires 219.0428 found 219.0427 (−0.2 ppm).

(*E*)-4,4,4-Trifluoro-1-(4-(methylthio)henyl)but-2-en-1-one

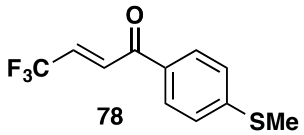

78

Following general procedure **C**, diisopropylamine (7.71 mL, 55.0 mmol) and *n*-BuLi (2.5M in hexanes, 22.0 mL, 55.0 mmol) in THF (65 mL), 2-bromo-3,3,3-trifluoroprop-1-ene (2.65 mL, 25.0 mmol) in THF (45 mL) and 4-(methylthio)benzaldehyde (3.99 mL, 30.0 mmol) gave the intermediate alcohol. Subsequent treatement with Et_3N (13.9 mL, 100 mmol) in THF (140 mL) gave, after chromatographic purification (eluent Et_2O:petrol 10:90), (*E*)-enone **78** as a light yellow solid (3.48 g, 57% over 2 steps); mp 102-104 °C; ν_{max} (ATR)/cm^{-1} 1585, 1636, 1682, 2967 (C=O), 3123 (C-H); δ_H (500 MHz, $CDCl_3$) 2.57 (3 H, s, C*H*$_3$), 6.84 (1 H, dq, *J* 15.5, 6.7, C(3)*H*), 7.33-7.35 (2 H, m, C(1)Ar(3,5)*H*), 7.54 (1 H, dq, *J* 15.5, 2.0, C(2)*H*), 7.91-7.93 (2 H, m, C(1)Ar(2,6)*H*); δ_C (125 MHz, $CDCl_3$) 14.6 (*C*H_3), 122.6 (q, *J* 268, *C*F_3), 125.1 (C(1)*ArC*(3,5)), 129.2 (C(1)*ArC*(2,6)), 130.0, (q, *J* 35.0, *C*(3)), 130.9 (q, *J* 5.5, *C*(2)), 132.4 (C(1)*ArC*(4)), 147.9 (C(1)*ArC*(1)), 168.7 (*C*(1)); *m/z* (APCI$^+$) 247 ([M+H]$^+$, 10%); HRMS (APCI$^+$) $C_{11}H_{10}F_3OS^+$ ([M+H]$^+$) requires 247.0399; found 247.0396 (−1.2 ppm).

(2*E*)-4,4,4-Trifluoro-1-(naphthalene-2-yl)but-2-en-1-one[13]

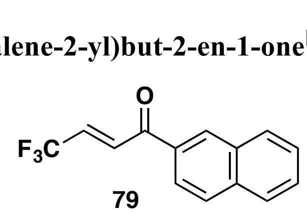

79

Following general procedure **C**, diisopropylamine (6.58 mL. 46.9 mmol) and *n*-BuLi (2.5 M in hexane, 18.8 mL, 46.9 mmol) in anhydrous THF (100 mL), 2-bromo-3,3,3-trifluoroprop-1-ene (2.3 mL, 21.3 mmol) and 2-naphthaldehyde (4.0 g, 25.6 mmol) gave intermediate alcohol. Subsequent treatment with Et_3N (11.2 mL, 80.0 mmol) in THF (75 mL) gave, after chromatographic purification (eluent; EtOAc:Petrol; 5:95) gave (*E*)-enone **79** as yellow solid (2.94 g, 59% over 2 steps); mp 70-72 °C;[6] δ_H (500 MHz, $CDCl_3$) 6.85 (1 H, dq, *J* 15.5, 6.7

C(3)*H*), 7.55-7.69 (3 H,m, Np*H*), 7.85-8.02 (4 H, m, Np*H,* and C(2)*H*), 8.40 (1 H, s, Np(2)*H*). All spectrascopic data in accordance with literature.[13]

(*E*)-4,4,4-Trifluoro-1-(furan-2-yl)but-2-en-1-one[14]

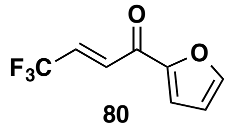

80

Following general procedure **C**, diisopropylamine (7.71 mL, 55.0 mmol) and *n*-BuLi (2.5M in hexanes, 22.0 mL, 55.0 mmol) in THF (65 mL), 2-bromo-3,3,3-trifluoroprop-1-ene (2.65 mL, 25.0 mmol) in THF (45 mL) and furfural (2.50 mL, 30.0 mmol) gave the intermediate alcohol. Subsequent treatement with Et_3N (13.9 mL, 100 mmol) in THF (140 mL) gave, after chromatographic purification (eluent Et_2O:petrol 10:90), (*E*)-enone **80** as an off-white solid (2.48 g, 52% over 2 steps); mp 66-68 °C; {lit.[14] mp 64-66 °C}; δ_H (500 MHz, $CDCl_3$) 6.67 (1 H, dd, *J* 3.7, 1.7, C(1)Ar(4)*H*), 6.92 (1 H, dq, *J* 15.6, 6.7, C(3)*H*), 7.41-7.46 (2 H, m, C(2)*H* and Ar*H*), 7.73 (1 H, m, Ar*H*). All data in accordance with literature.[14]

(2*E*)-4,4,4-Trifluoro-1-(thiophen-2-yl)but-2-en-1-one[13]

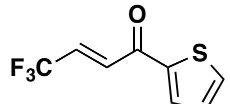

81

Following general procedure **C**, diisopropylamine (11.0 mL. 78.5 mmol) and *n*-BuLi (2.5 M in hexane, 31.4 mL, 78.5 mmol) in anhydrous THF (100 mL), 2-bromo-3,3,3-trifluoroprop-1-ene (3.2 mL, 29.8 mmol) and 2-thiophene carboxaldehyde (3.27 mL, 35.7 mmol) gave intermediate alcohol. Subsequent treatment or crude with Et_3N (24.4 mL, 174.8 mmol) in THF (100 mL) gave, after chromatographic purification (eluent; EtOAc:Petrol; 10:90) gave (*E*)-enone **81** as yellow solid (2.0 g, 27% over 2 steps); mp 47-49 {lit.[13] 50-52 °C}; δ_H (500 MHz, $CDCl_3$) 6.85 (1 H, dq, *J* 15.4, 6.66 C(3)*H*), 7.21 (1 H, dd, *J* 4.9, 3.9, C(1)Ar(3)*H*), 7.40 (1 H, dq, *J* 15.5, 2.0, C(2)*H*), 7.79 (1 H, dd, *J* 5.0, 1.1, C(1)Ar(2)*H*) 7.84 (1 H, dd. *J* 3.9, 1.1, C(1)Ar(4)*H*). All data in accordance with literature.[13]

Ethyl (2*E*)-4-[(benzenesulfonyl)imino]-4-phenylbut-2-enoate[15]

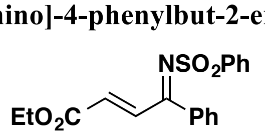

5

Following general procedure **D**, ethyl 3-benzoylacrylate (5.5mL, 30 mmol) and benzenesulfonamide (4.72 g, 30 mmol) were stirred in CH_2Cl_2 (50mL), Et_3N (8.3 mL, 60 mmol) and $TiCl_4$ (3.3 mL, 30 mmol) gave, after recrystallisation (Et_2O:Petrol), ketimine **5** as

orange solid (4.59 g, 44.6%): mp 54-56°C; {lit.[15] 51-53 °C}; δ_H (300 MHz, $CDCl_3$) 1.37 (3 H, t, *J* 7.1, CH_2CH_3), 4.33 (2 H, q, *J* 7.0, CH_2CH_3), 6.27 (1 H, d, *J* 16.2, C(3)*H*), 7.48 (1 H, t, *J* 7.7, Ar*H*), 7.53 – 7.82 (8 H, m, Ar*H*)), 8.06 (1 H, d, *J* 7.1, C(2)*H*). All data in accordance with literature.[15]

(2*E*)-Benzyl 4-phenyl-4-(tosylimino)but-2-enoate

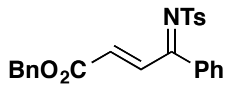

82

Following general procedure **D**, keto ester **61** (2.90 g, 10.9 mmol) and 4-toluenesulfonamide (1.86 g, 10.9 mmol) in CH_2Cl_2 (25 mL), Et_3N (3.04 mL, 21.8 mmol) and $TiCl_4$ (1.20 mL, 10.9 mmol) gave, after recrystallisation (Et_2O:Petrol), ketimine **82** as a light brown solid (3.04 g, 67%); mp 96-98 °C; ν_{max} (ATR)/cm^{-1} 1143 (S=O), 1304 (S=O), 1548, 1721 (C=O), 3067 (C-H); δ_H (300 MHz, $CDCl_3$) 2.47 (3 H, s, CH_3), 5.32 (2 H, s, CH_2), 6.30 (1 H, d, *J* 16.2, C(2)*H*), 7.34-7.48 (9 H, m, Ar*H*), 7.60 (1 H, t, *J* 7.4, C(4)Ar(4)*H*), 7.65-7.80 (2 H, m, SO_2Ar(2,6)*H*), 7.94 (2 H, m, C(4)Ar(2,6)*H*), 8.31-8.36 (1 H, m, C(3)*H*); δ_C (100 MHz, $CDCl_3$) 21.7 (CH_3), 67.3 (CH_2), 127.5 (SO_2*ArC*(2,6)), 128.6 (*ArC*), 128.6 (*ArC*), 128.7 (*ArC*), 128.7 (*ArC*), 129.6 (SO_2*ArC*(3,5)), 130.2 (C(4)*ArC*(2,6)), 132.4 (*C*(2)), 133.5 (C(4)*ArC*(4)), 135.3 (CH_2*ArC*(1)), 135.8 (C(4)*ArC*(1)), 137.3 (*C*(3)), 137.6 (SO_2*ArC*(4)), 144.1 (SO_2*ArC*(1)), 164.5 (*C*(1)), 174.5 (*C*(4)); *m/z* ($APCI^+$) 420 ($[M+H]^+$, 100%); HRMS ($APCI^+$) $C_{24}H_{22}NO_4S^+$ ($[M+H]^+$) requires 420.1264; found 420.1260 (−1.0 ppm).

(2*E*)-benzyl 4-(*p*-tolyl)-4-(tosylimino)but-2-enoate

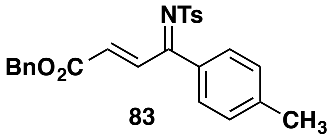

83

Following general procedure **D**, keto ester **62** (3.64 g, 13.0 mmol) and 4-toluenesulfonamide (2.22 g, 13.0 mmol) in CH_2Cl_2 (35 mL), Et_3N (3.62 mL, 26.0 mmol) and $TiCl_4$ (1.43 mL, 13.0 mmol) gave, after concentration *in vacuo*, crude ketimine **83** as a brown oil that was used crude without further purification; δ_H (500 MHz, $CDCl_3$) 2.43 (3H, s, CH_3), 2.45 (3H, s, CH_3), 5.30 (2H, s, CH_2), 6.26 (1H, d, *J* 16.2, C(2)*H*), 7.24 (2H, d, *J* 8.0, C(1)Ar(3,5)*H*), 7.32-7.45 (7H, m, Ar*H*), 7.65-7.66 (2H, m, SO_2Ar(2,6)*H*), 7.91 (2H, d, *J* 7.3, C(4)Ar(2,6)*H*), 8.20-8.35 (1H, m, C(3)*H*).

(2*E*)-Benzyl 4-(4-nitrophenyl)-4-(tosylimino)but-2-enoate

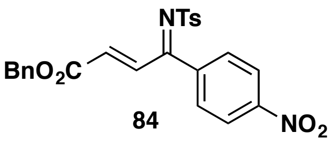

84

Following general procedure **D**, keto ester **63** (0.96 g, 3.09 mmol) and 4-toluenesulfonamide (0.53 g, 3.09 mmol) in CH_2Cl_2 (20 mL), Et_3N (0.86 mL, 6.188 mmol) and $TiCl_4$ (0.34 mL, 3.09 mmol) gave, after concentration *in vacuo*, crude ketimine **84** as a brown oil which was used crude without further purification; δ_H (300 MHz, $CDCl_3$) 2.459 (3H, s, C*H*$_3$), 5.32 (2H, s, C*H*$_2$), 6.27 (1H, d, *J* 16.2, C(2)*H*), 7.37-7.44 (7H, m, Ar*H*), 7.84-7.96 (4H, m, Ar*H*), 8.29-8.42 (3H, m, Ar*H* and C(3)*H*).

(2*E*)-Benzyl 4-(4-methoxyphenyl)-4-(tosylimino)but-2-enoate

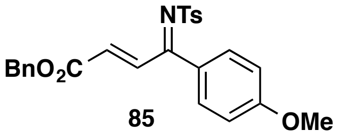

85

Following general procedure **D**, keto ester **64** (3.49 g, 11.8 mmol) and 4-toluenesulfonamide (2.02 g, 11.8 mmol) in CH_2Cl_2 (30 mL), Et_3N (3.29 mL, 23.6 mmol) and $TiCl_4$ (1.30 mL, 11.8 mmol) gave, after concentration *in vacuo*, crude ketimine **85** as a brown oil that was used crude without further purification; δ_H (500 MHz, $CDCl_3$) 2.45 (3H, s, ArC*H*$_3$), 3.88 (3H, s, OC*H*$_3$), 5.30 (2H, s, C*H*$_2$), 6.24 (1H, d, *J* 16.3, C(2)*H*), 6.92 (2H, d, *J* 8.9, C(1)Ar(3,5)*H*), 7.31-7.46 (7H, m, Ar*H*), 7.78 (2H, d, *J* 8.3, SO_2Ar(2,6)*H*), 7.91 (2H, d, *J* 8.2, C(4)Ar(2,6)*H*), 8.10-8.26 (1H, m, C(3)*H*).

Methyl (2*E*)-4-{[(4-methylbenzene)sulfonyl]imino}-4-phenylbut-2-enoate

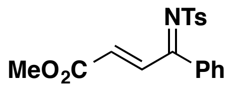

86

Following general procedure **B**, keto ester **65** (4.70 g, 24.7 mmol) and 4-toluenesulfonamide (4.23 g, 24.7 mmol) in CH_2Cl_2 (100 mL), Et_3N (6.79 mL, 49.4 mmol) and $TiCl_4$ (2.71 mL, 24.7 mmol) gave, after concentration *in vacuo*, crude ketimine **86** as a brown oil that was used crude without further purification; δ_H (300 MHz, $CDCl_3$) 2.42 (3 H, s, SO_2ArC*H*3), 3.82 (3 H, s, C*H*$_3$), 6.22 (1 H, d, *J* 16.2, C(2)*H*), 7.24-7.57 (7 H, m, Ar*H*), 7.89 (2 H, d, *J* 7.46, SO-$_2$Ar(2,6) *H*), 8.23 (1 H, d, *J* 14.2, C(3)*H*).

Methyl (2*E*)-4-(4-cyanophenyl)-4-{[(4-methylbenzene)sulfonyl]imino}but-2-enoate

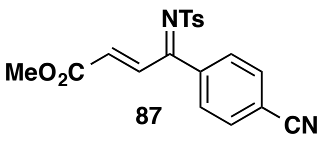

87

Following general procedure **D**, keto ester **66** (2.00 g, 9.30 mmol) and 4-toluenesulfonamide (1.59 g, 9.30 mmol) in CH_2Cl_2 (25 mL), Et_3N (2.56 mL, 18.6 mmol) and $TiCl_4$ (1.02 mL, 9.30 mmol) gave, after concentration *in vacuo*, crude ketimine **87** as a brown oil that was used crude without further purification; δ_H (300 MHz, $CDCl_3$) 2.48 (3 H, s, SO_2ArCH_3), 3.89 (3 H, s, CH_3), 6.23 (1H, d, *J* 15.9, C(2)*H*), 7.38 (2 H, d, *J* 7.92 SO_2Ar(3,5)*H*), 7.71-7.93 (4 H, m, Ar*H*), 8.11 (2 H, d, *J* 8.70, SO_2Ar(2,6)*H*), 8.29 (2 H, d, *J* 15.6, C(3)*H*).

Methyl (2*E*)-4-{[(4-methylbenzene)sulfonyl]imino}-4-[4-(trifluoromethyl)phenyl]but-2-enoate

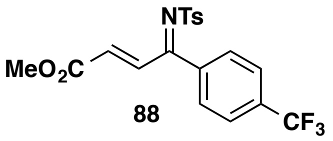

88

Following general procedure **D**, keto ester **67** (1.57 g, 6.08 mmol) and 4-toluenesulfonamide (1.04 g, 6.08 mmol) in CH_2Cl_2 (25 mL), Et_3N (1.68 mL, 12.2 mmol) and $TiCl_4$ (0.67 mL, 6.08 mmol) gave, after concentration *in vacuo*, crude ketimine **88** as a brown oil that was used crude without further purification; δ_H (300 MHz, $CDCl_3$) 2.45 (3 H, s, CH_3), 3.85 (3 H, s, CH_3), 6.21 (1H, d, *J* 16.1, C(2)*H*), 7.35 (2 H, d, *J* 7.77, SO_2Ar(3,5)*H*), 7.61-7.91 (6 H, m, Ar*H*), 8.29 (1 H, d, *J* 16.2, C(3)*H*).

(2*E*)-Methyl 4-(4-chlorophenyl)-4-(tosylimino)but-2-enoate

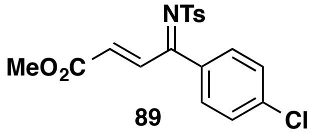

89

Following general procedure **B**, keto ester **68** (2.34 g, 10.4 mmol) and 4-toluenesulfonamide (1.78 g, 10.4 mmol) in CH_2Cl_2 (25 mL), Et_3N (2.90 mL, 20.8 mmol) and $TiCl_4$ (1.15 mL, 10.4 mmol) gave, after concentration *in vacuo*, crude ketimine **89** as a brown oil that was used crude without further purification; δ_H (400 MHz, $CDCl_3$) 2.45 (3 H, s, $ArCH_3$), 3.86 (3 H, s, OCH_3), 6.23 (1 H, d, *J* 16.3, C(2)*H*), 7.36 (2 H, d, *J* 8.0, C(4)Ar(3,5)*H*), 7.41 (2 H, d, *J* 8.4, SO_2Ar(3,5)*H*), 7.62-7.75 (2 H, m, SO_2Ar(2,6)*H*), 7.91 (2 H, d, *J* 7.3, C(4)Ar(2,6)*H*), 8.22 (1 H, d, *J* 16.3, C(3)*H*); δ_C (100 MHz, $CDCl_3$) 21.7 (Ar*C*H_3), 52.6 (O*C*H_3), 127.5 (*C*(4)*ArC*(2,6)), 129.1 (SO_2*ArC*(3,5)), 129.7 (C(4)*ArC*(2,6)), 131.5 (SO_2*ArC*(2,6)), 132.2

(*C*(2)), 134.1 (*C*(4)*ArC*(1)), 136.8 (*C*(3)), 137.4 (SO_2*ArC*(1)), 140.1 (*C*(4)*ArC*(4)), 144.3 (SO_2*ArC*(4)), 164.9 (*C*O_2CH_3), 173.1 (*C*(4)).

(2*E*)-Benzyl 4-(2-fluorophenyl)-4-(tosylimino)but-2-enoate

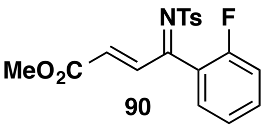

90

Following general procedure **D**, keto ester **69** (2.96 g, 14.2 mmol) and 4-toluenesulfonamide (2.45 g, 14.2 mmol) in CH_2Cl_2 (50 mL), Et_3N (3.97 mL, 28.5 mmol) and $TiCl_4$ (1.56 mL, 14.2 mmol) gave, after concentration *in vacuo*, crude ketimine **90** as a brown oil which was used crude without further purification; δ_H (500 MHz, $CDCl_3$) 2.45 (3H, s, ArCH_3), 3.78-3.86 (3H, m, OCH_3), 6.18 (1H, d, *J* 15.9, C(2)*H*), 7.14-7.15 (2H, m, Ar*H*), 7.30-7.54 (5H, m, Ar*H*), 7.78-7.94 (2H, m, Ar*H* and C(3)*H*).

Methyl (2*E*)-4-{[(4-methylbenzene)sulfonyl]imino}-4-(naphthalene-2-yl)but-enoate

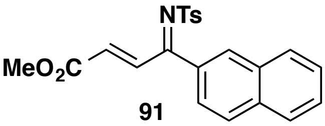

91

Following general procedure **D**, keto ester **70** (3.00 g, 12.5 mmol) and 4-toluenesulfonamide (2.15 g, 12.5 mmol) in CH_2Cl_2 (30 mL), Et_3N (3.44 mL, 25.0 mmol) and $TiCl_4$ (1.37 mL, 12.5 mmol) gave, after concentration *in vacuo*, crude ketimine **91** as a brown oil that was used crude without further purification; δ_H (300 MHz, $CDCl_3$) 2.48 (3 H, s, SO_2ArC*H*3), 3.87 (3 H, s, CH_3), 6.29 (1 H, d, *J* 16.3, C(2)*H*), 7.30-7.36 (3 H, m, Ar*H*), 7.55-7.58 (4 H, m, Ar*H*), 7.79-7.95 (5 H, m, Ar*H* and C(3)*H*).

Methyl (2*E*)-5,5-dimethyl-4-{[(4-methylbenzene)sulfonyl]imino}hex-2-enoate

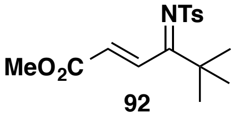

92

Following general procedure **D**, keto ester **71** (560 mg, 3.29 mmol) and 4-toluenesulfonamide (563 mg, 3.29 mmol) in CH_2Cl_2 (40 mL), Et_3N (0.91 mL, 6.58 mmol) and $TiCl_4$ (0.36 mL, 3.29 mmol) gave, after chromatographic purification (eluent; EtOAc:Petrol, 30:70), ketimine **92** as brown solid (360 mg, 34%); mp 70-72 ^oC; ν_{max} (ATR)/cm^{-1} 1310 (S=O), 1597 (C=N), 2974 (C-H); δ_H (500 MHz, $CDCl_3$) 1.17 (9 H, s, (CH_3)$_3$), 2.43 (3 H, s, SO_2ArCH_3), 6.28 (1 H, d, *J* 16.5, C(3)*H*), 7.31 (2 H, d, *J* 8.15, SO_2Ar(3,5)*H*), 7.55 (1 H, d, *J* 16.5, C(2)*H*), 7.82 (2 H, d, *J* 8.2, SO_2Ar(2,6)*H*); δ_C (125 MHz, $CDCl_3$); 21.6 (SO_2Ar*C*H_3), 27.5 ((*C*H_3)$_3$), 42.5 (*C*(CH-$_3$)$_3$, 52.3 (*C*H_3O), 126.7 (*C*(3)H), 127.3 (SO_2*ArC*(2,6)H), 129.4 (SO_2*ArC*(3,5)H), 137.9 (SO-

${}_2$*ArC*(4)), 138.7 (*C*(2)H), 143.8 (SO_2*ArC*(1)), 165.0 (C(1)), 188.5 (*C*(1)); *m*/*z* ($APCI^+$) 324 ($[M+H]^+$, 100%); HRMS ($APCI^+$) $C_{16}H_{22}NO_4S^+$ ($[M+H]^+$) requires 324.1264; found 324.1267 (+0.9 ppm).

4-Methyl-*N*-((*E*)-4,4,4-trifluoro-1-phenylbut-2-en-1-ylidine)benzenesulfonamide

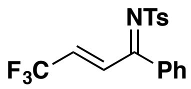

93

Following general procedure **D**, trifluoromethyl enone **72** (7.22 g, 36.1 mmol) and 4-toluenesulfonamide (6.18 g, 36.1 mmol) in CH_2Cl_2 (200 mL), Et_3N (10.1 mL, 72.2 mmol) and $TiCl_4$ (3.98 mL, 36.1 mmol) gave, after recrystallisation (Et_2O:Petrol), ketimine **93** as a white solid (10.2 g, 80%); mp 102-104 °C; ν_{max} (ATR)/cm^{-1} 1306, 1549, 1582, 1597, 1582 (S=O), 1146 (S=O), 3053 (C-H),; δ_H (500 MHz, $CDCl_3$) 2.47 (3 H, s, C*H*$_3$), 6.15 (1 H, dq, *J* 16.3, 6.1, C(3)*H*), 7.38 (2 H, d, *J* 8.0, SO_2Ar(3,5)*H*), 7.48 (2 H, t, *J* 7.7, C(1)Ar(3,5)*H*), 7.62 (1 H, t, *J* 7.4, C(1)Ar(4)*H*), 7.67-7.80 (2 H, m, SO_2Ar(2,6)*H*), 7.82-8.00 (3 H, m, C(2)*H* and C(1)Ar(2,6)*H*); δ_C (125 MHz, $CDCl_3$) 21.7 (*C*H$_3$), 121.9 (q, *J* 270, *C*F$_3$), 126.5 (*C*(3)), 127.5 (C(1)*ArC*(3,5)), 128.9 (C(1)*ArC*(4)), 129.7 (C(1)*ArC*(2,6)), 130.2 (SO_2*ArC*), 131.7 (SO_2*ArC*), 133.7 (*C*(2)), 135.5 (SO_2*ArC*(4)), 137.4 (C(1)*ArC*(1)), 144.3 (SO_2*ArC*(1)), 172.9 (*C*(1)); δ_F (470 MHz, $CDCl_3$) −65.1 (C*F*$_3$); *m*/*z* ($APCI^+$) 354 ($[M+H]^+$, 100%); HRMS ($APCI^+$) $C_{17}H_{15}F_3NO_2S^+$ ($[M+H]^+$) requires 354.0770; found 354.0768 (−0.6 ppm).

4-Methyl-*N*-((*Z*)-4,4,4-trifluoro-1-phenylbut-2-en-1-ylidine)benzenesulfonamide

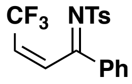

94

Following general procedure **D**, trifluoromethyl enone **73** (1.72 g, 8.60 mmol) and 4-toluenesulfonamide (1.47 g, 8.60 mmol) in CH_2Cl_2 (50 mL), Et_3N (2.40 mL, 17.2 mmol) and $TiCl_4$ (0.95 mL, 8.60 mmol) gave, after recrystallisation (Et_2O:Petrol), ketimine **94** (90% pure with 10% $TsNH_2$ impurity) as a white solid (0.64 g, 21%); mp 98-100 °C; ν_{max} (ATR) 1143 (S=O), 1310 (S=O), 1557, 1587, 2968 (C-H); δ_H (500 MHz, $CDCl_3$) 2.48 (3H, s, C*H*$_3$), 6.20 (1H, dq, *J* 12.8, 7.7, C(3)*H*), 7.18 (1H, d, *J* 12.8, C(2)*H*), 7.39 (2H, d, *J* 8.0, SO_2Ar(3,5)*H*), 7.47 (2H, t, *J* 7.9, C(1)Ar(3,5)*H*), 7.62 (1H, t, *J* 7.4, C(1)Ar(4)*H*), 7.92-7.95 (4H, m, C(1)Ar(2,6)*H* and SO_2Ar(2,6)*H*); δ_C (100 MHz, $CDCl_3$) 21.7 (*C*H$_3$), 121.7 (q, *J* 270, *C*F$_3$), 122.1 (q, *J* 34.8, *C*(3)), 127.6 (*ArC*), 128.9 (*ArC*), 129.5 (*ArC*), 129.6 (*ArC*), 132.7 (q, *J* 5.4, *C*(2)), 134.2 (C(1)*ArC*(4)), 134.5 (SO_2*ArC*(4)), 137.2 (C(1)*ArC*(1)), 144.2 (SO_2*ArC*(1)), 172.8 (*C*(1)); δ_F (282 MHz, $CDCl_3$) −65.4 (C*F*$_3$); *m*/*z* (NSI^+) 354 ($[M+H]^+$, 12%); HRMS (NSI^+) $C_{17}H_{15}F_3NO_2S^+$ ($[M+H]^+$) requires 354.0770; found 354.0762 (-2.3 ppm).

***N*-((*E*)-4,4,4-Trifluoro-1-phenylbut-2-en-1-ylidine)benzenesulfonamide**

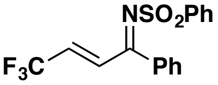

42

Following general procedure **D**, trifluoromethyl enone **72** (7.22 g, 36.1 mmol) and benzenesulfonamide (5.67 g, 36.1 mmol) in CH_2Cl_2 (200 mL), Et_3N (10.1 mL, 72.2 mmol) and $TiCl_4$ (3.98 mL, 36.1 mmol) gave, after recrystallisation (Et_2O:Petrol), ketimine **42** as a white solid (6.98 g, 57%); mp 72-74 °C; ν_{max} (ATR)/cm^{-1} 1304, 1551, (S=O), 1151 (S=O), 3083 (C-H); δ_H (500 MHz, $CDCl_3$) 6.18 (1 H, dq, *J* 16.3, 6.1, C(3)*H*), 7.48-7.68 (7 H, m, Ar*H*), 7.70-7.80 (2 H, m, Ar*H*), 7.85-7.95 (1 H, m, C(2)*H*), 8.05 (2 H, d, *J* 4.1, C(1)Ar(2,6)*H*); δ_C (125 MHz, $CDCl_3$) 121.9 (q, *J* 270, *C*F_3), 126.4 (*C*(3)), 127.4 (C(1)*ArC*(3,5)), 128.9 (C(1)*ArC*(4)), 129.1 (C(1)*ArC*(2,6)), 130.2 (SO_2*ArC*), 131.7 (SO_2*ArC*), 133.4 (*C*(2)), 133.9 (SO_2*ArC*(4)), 135.3 (C(1)*ArC*(1)), 140.3 (SO_2*ArC*(1)), 173.3 (*C*(1)); *m/z* (NSI$^+$) 340 ([M+H]$^+$, 7%); HRMS (NSI$^+$) $C_{16}H_{13}F_3NO_2S^+$ ([M+H]$^+$) requires 340.0614; found 340.0615 (+0.4 ppm).

***N*-[(2*E*)-1-(3-Bromophenyl)-4,4,4-trifluoro-1-(4-methylphenyl)but-2-en-1-ylidene]benzene-1-sulfonamide**

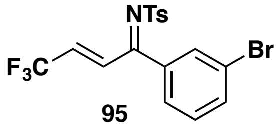

95

Following general procedure **D**, and trifluoromethyl enone **74** (1.9 g, 6.8 mmol) and 4-toluenesulfonamide (1.16 g, 6.80 mmol) in CH_2Cl_2 (50 mL), Et_3N (1.88 mL, 13.6 mmol) and $TiCl_4$ (0.75 mL, 6.8 mmol) gave, after recrystallisation (Et_2O/Petrol), ketimine **95** as orange solid (2.0 g, 80%): mp 64-66 ^oC; ν_{max} (ATR)/cm^{-1} 1100 (ArBr), 1287, 1314 (SO_2), 1558 (C=N), 297.2, 3072.6 (C-H); δ_H (500 MHz, $CDCl_3$); 2.46 (3 H, s, SO_2ArC*H_3*), 6.13 (1 H, dq, *J* 16.3, 6.1, C(3)*H*), 7.30-7.37 (3 H, m, Ar(5)*H* and SO_2Ar(3,5)*H*), 7.60 (1 H, s, Ar(4)*H*), 7.70 (1 H, d, *J* 7.8, C(2)*H*), 7.80-7.89 (4 H, m, Ar(2)*H*, Ar(6)*H* and SO_2Ar(2,6)*H*); δ_C (125 MHz, $CDCl_3$); 21.7 (SO_2Ar*C*H_3), 122.8 (*ArC*(3)Br), 126.5 (SO_2*ArC*(2,6)H), 127.6 (*ArC*(2)H), 128.8 (*ArC*(4)H), 129.8 (*ArC*(5)H and SO_2*ArC*(3,5)H), 130.3 (*C*(3)H), 131.2 (*ArC*(4)H), 136.4 (*C*(2)H), 136.9 (SO_2*ArC*(4)), 144.6 (SO_2*ArC*(1) and *ArC*(1)), 171.2 (*C*(1)); *m/z* (NSI$^+$) 432 ([M+H], 65%), HRMS (NSI$^+$) $C_{17}H_{14}{}^{79}BrF_3NO_2S^+$ ([M+H]$^+$) requires 431.9875 found 431.9871 (−0.1 ppm).

***N*-((*E*)-1-(4-Bromophenyl)-4,4,4-trifluorobut-2-en-1-ylidine)-4 methylbenzenesulfonamide**

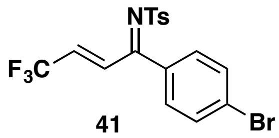

41

Following general procedure **D**, trifluoromethyl enone **75** (4.62 g, 16.6 mmol) and 4-toluenesulfonamide (2.83 g, 16.6 mmol) in CH_2Cl_2 (70 mL), Et_3N (4.63 mL, 33.1 mmol) and $TiCl_4$ (1.82 mL, 16.6 mmol) gave, after recrystallisation (Et_2O:Petrol), ketimine **41** as a white solid (4.62 g, 65%); mp 78-80 °C; ν_{max} (ATR)/cm^{-1} 1304, 1549, 1574 (S=O), 1140 (S=O), 3103 (C-H); δ_H (500 MHz, $CDCl_3$) 2.47 (3 H, s, C*H*$_3$), 6.15 (1 H, dq, *J* 16.3, 6.1, C(3)*H*), 7.38 (2 H, d, *J* 8.0, C(1)Ar(3,5)*H*), 7.62 (4 H, app. s, SO_2Ar*H*), 7.85-7.92 (3 H, m, C(2)*H* and C(1)Ar(2,6)*H*); δ_C (125 MHz, $CDCl_3$) 21.7 (*C*H$_3$), 121.8 (q, *J* 282, *C*F$_3$), 126.4 (*C*(3)), 127.5 (C(1)*ArC*(3,5)), 129.2 (C(1)*ArC*(4)), 129.7 (C(1)*ArC*(2,6)), 131.5 (SO_2*ArC*), 131.5 (SO_2*ArC*), 132.2 (*C*(2)), 134.3 (SO_2*ArC*(4)), 137.1 (C(1)*ArC*(1)), 144.5 (SO_2*ArC*(1)), 171.7 (*C*(1)); *m/z* (APCI$^+$) 432 ([M+H]$^+$, 95%); HRMS (APCI$^+$) $C_{17}H_{14}{}^{79}BrF_3NO_2S^+$ ([M+H]$^+$) requires 431.9875; found 431.9875 (+0.0 ppm).

***N*-((*E*)-1-(4-Bromophenyl)-4,4,4-trifluorobut-2-en-1-ylidine)-benzenesulfonamide**

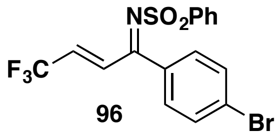

96

Following general procedure **D**, trifluoromethyl enone **75** (4.62 g, 16.6 mmol) and benzenesulfonamide (2.61 g, 16.6 mmol) in CH_2Cl_2 (70 mL), Et_3N (4.63 mL, 33.1 mmol) and $TiCl_4$ (1.82 mL, 16.6 mmol) gave, chromatographic purification (eluent Et_2O:petrol 15:85), ketimine **96** as an orange oil (3.46 g, 50%); ν_{max} (ATR)/cm^{-1} 1574, 1549, 1310 (S=O), 1147 (S=O) 3110 (C-H); δ_H (500 MHz, $CDCl_3$) 6.17 (1 H, dq, *J* 16.3, 6.1, C(3)*H*), 7.57-7.70 (7 H, m, Ar*H*), 7.82-7.93 (1 H, m, C(2)*H*), 8.04 (2 H, d, *J* 5.8, C(1)Ar(2,6)*H*); δ_C (125 MHz, $CDCl_3$) 121.8 (q, *J* 270, *C*F$_3$), 127.5 (C(1)*ArC*(3,5)), 129.2 (C(1)*ArC*(2,6)), 129.3 (C(1)*ArC*(4)), 130.1 (*C*(3)), 131.5 (SO_2*ArC*), 131.5 (SO_2*ArC*), 132.3 (*C*(2)), 134.5 (SO_2*ArC*), 134.3 (SO_2*ArC*(1)), 140.1 (C(1)*ArC*(1)), 172.1 (*C*(1)); *m/z* (NSI$^+$) 419 ([M+H]$^+$, 65%); HRMS (NSI$^+$) $C_{16}H_{11}{}^{81}BrF_3NO_2S^+$ ([M+H]$^+$) requires 419.9704; found 419.9694 (−1.2 ppm).

4-Methyl-*N*-[(2*E*)-4,4,4-trifluoro-1-(4-methylphenyl)but-2-en-1-ylidene]benzene-1-sulfonamide

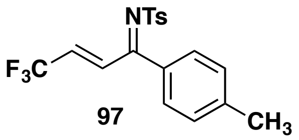

Following general procedure **D**, trifluoromethyl enone **76** (2.0 g, 9.89 mmol) and 4-toluenesulfonamide (1.69 g, 9.89 mmol) in CH_2Cl_2 (50 mL), Et_3N (2.72 mL, 19.8 mmol) and $TiCl_4$ (1.10 mL, 9.89 mmol) gave, after recrystallisation (Et_2O/Petrol), ketimine **97** as orange solid (2.8 g, 70%): mp 106-109 °C; ν_{max} (ATR)/cm^{-1} 1285 (SO_2), 1557 (C=N), 3261 (C-H); δ_H (400 MHz, $CDCl_3$); 2.42 (3 H, s,ArC*H₃*), 2.45 (3 H, s, SO_2ArC*H₃*), 6.11 (1 H, dq, *J* 16.3, 6.12, C(3)*H*); 7.25 (2 H, d, *J* 8.0, Ar(3)*H*), 7.34 (2 H, d, *J* 8.1, SO_2Ar (3,5)*H*), 7.62 (3 H, br s, Ar(2,6)*H*), 7.75-7.95 (3 H, m, C(2)*H* and SO_2Ar (2,6)*H*); δ_C (100 MHz, $CDCl_3$) 21.6 (*C*H_3), 21.7 (SO_2Ar*C*H_3), 121.9 (q, *J* 265, *C*F_3), 127.4 (SO_2*ArC*(2,6)H)), 129.6 (*C*(3)H, *ArC*(3,5)H and SO_2*ArC*(3,5)H), 130.3 (*C*(2)H), 131.9 (*ArC*(2,6)H), 137.6 (*ArC*(4) and SO_2*ArC*(4)), 144.2 (*ArC*(1) and SO_2*ArC*(1)), 172.2 (*C*(1)); *m/z* (NSI^+) 368 ([M+H], 100%), HRMS (NSI^+) $C_{18}H_{17}F_3NO_2S^+$ ($[M+H]^+$) requires 368.0927 found 368.0928 (+0.4 ppm).

4-Methyl-*N*-[(2*E*)-4,4,4-trifluoro-1-(4-fluorophenyl)but-2-en-1-ylidene] benzene-1-sulfonamide

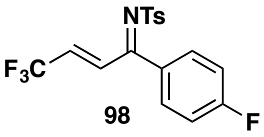

Following general procedure **D**, trifluoromethyl enone **77** (2.6 g, 11.9 mmol) and 4-toluenesulfonamide (2.03 g, 11.9 mmol) in CH_2Cl_2 (40 mL), Et_3N (3.28 mL, 23.8 mmol), $TiCl_4$ (1.31 mL, 11.9 mmol) gave, after recrystallisation (Et_2O/Petrol), ketimine **98** as off-white solid (2.73 g, 62%): mp 78-80 °C; ν_{max} (ATR)/cm^{-1} 1152 (ArCF), 1303 (SO_2), 1564 (C=N), 3100 (C-H); δ_H (400 MHz, $CDCl_3$); 2.45 (3 H, s, SO_2ArC*H₃*), 6.12 (1 H, dq, *J* 16.3, 6.1, C(3)*H*), 7.15 (2 H, t, *J* 8.6, Ar(3,5)*H*), 7.36 (2 H, d, *J* 8.2, SO_2Ar(3,5)*H*), 7.77-7.84 (3 H, m, Ar(2)*H* and C(2)*H*), 7.89 (2 H, d, *J* 8.6, SO_2Ar(2,5)*H*); δ_C (125 MHz, $CDCl_3$); 21.7 (SO_2Ar*C*H_3), 116.2 (d, *J* 22.2, *ArC*(3,5)H), 121.7 (q, *J* 270.9, *C*F_3), 126.4 (*ArC*(2,6)H), 127.5 (SO_2*ArC*(2,6)H), 129.7 (SO_2*ArC*(3,5)H and *C*(3)H), 132.8 (*C*(2)H), 137.2 (SO_2*ArC*(4)), 139.3 (*ArC*(1)), 144.4 (SO_2*ArC*(1)), 165.2 (*C*(1)), 169.2 (d, *J* 519, *ArC*(4)F); *m/z* ($APCI^+$) 372 ([M+H], 100%), HRMS ($APCI^+$) $C_{17}H_{13}F_4NO_2S^+$ ($[M+H]^+$) requires 372.0676 found 372.0677 (+0.3 ppm).

4-Methyl-*N*-((*E*)-4,4,4-trifluoro-1-(4-(methylthio)phenyl)but-2-en-1-ylidine)benzenesulfonamide

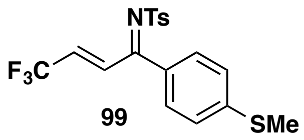

99

Following general procedure **D**, trifluoromethyl enone **78** (3.27 g, 13.3 mmol) and 4-toluenesulfonamide (2.28 g, 13.3 mmol) in CH_2Cl_2 (60 mL), Et_3N (3.73 mL, 26.6 mmol) and $TiCl_4$ (1.47 mL, 13.3 mmol) gave, chromatographic purification (eluent Et_2O:petrol 40:60), ketimine **99** as a yellow solid (4.60 g, 87%); mp 80-82 °C; ν_{max} (ATR)/cm^{-1} 1306, 1535, 1560, 2924 (S=O), 1144 (S=O), 3106 (C-H); δ_H (500 MHz, $CDCl_3$) 2.47 (3 H, s, C*H*$_3$), 2.54 (3 H, s, C*H*$_3$), 6.13 (1 H, dq, *J* 16.5, 5.8, C(3)*H*), 7.27 (2 H, d, *J* 8.5, SO_2Ar(3,5)*H*), 7.37 (2 H, d, *J* 8.1, C(1)Ar(3,5)*H*), 7.67 (2 H, d, *J* 7.2, SO_2Ar(2,6)*H*)), 7.81 (1 H, br s, C(2)*H*), 7.92 (2 H, d, *J* 8.1, C(1)Ar(2,6)*H*); δ_C (125 MHz, $CDCl_3$) 14.7 (S*C*H$_3$), 21.7 (Ar*C*H$_3$), 121.9 (q, *J* 270, *C*F$_3$), 125.1 (*ArC*), 127.4 (*ArC*), 128.9 (*C*), 129.6 (*ArC*), 130.5 (*C*), 131.2 (C(1)*ArC*(1)), 131.9 (*C*), 137.6 (4ry *C*), 144.2 (4ry *C*), 148.0 (4ry *C*), 172.0 (*C*(1)); *m/z* (NSI$^+$) 400 ([M+H]$^+$, 45%); HRMS (NSI$^+$) $C_{18}H_{17}F_3NO_2S_2^+$ ([M+H]$^+$) requires 400.0647; found 400.0646 (-0.3 ppm).

4-Methyl-*N*-((*E*)-4,4,4-trifluoro-1-(4-(methylsulfonyl)phenyl)but-2-en-1-ylidine)benzenesulfonamide

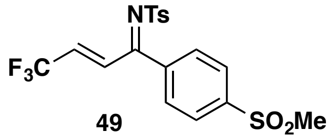

49

To a solution of ketimine **99** (4.60 g, 11.5 mmol) in CH_2Cl_2 (200 mL) at 0 °C was added *m*-CPBA (70% w/w, 5.70 g, 23.0 mmol) portionwise and the reaction mixture was allowed to stir at rt for 30 minutes. The reaction mixture was quenched by addition of sat. aq. $NaHCO_3$. The organic layer was dried ($MgSO_4$), filtered and concentrated *in vacuo*. Recrystallisation (Et_2O:Petrol) gave ketimine **49** as a white solid (2.85 g, 57%); mp 126-130 °C; ν_{max} (ATR)/cm^{-1} 1145 (S=O), 1309, 1558, 1584, 2920 (S=O), 3098 (C-H),; δ_H (500 MHz, $CDCl_3$) 2.48 (3 H, s, ArC*H*$_3$), 3.09 (3 H, s, SO_2C*H*$_3$), 6.09-6.17 (1 H, m, C(3)*H*), 7.38-7.40 (2 H, m, NSO_2Ar(3,5)*H*), 7.66-8.05 (7 H, m, Ar*H* and C(2)*H*); δ_C (125 MHz, $CDCl_3$) 21.7 (Ar*C*H$_3$), 44.3 (SO_2*C*H$_3$), 121.7 (q, *J* 270, *C*F$_3$), 124.0 (*C*(3)), 127.6 (*ArC*), 127.9 (*ArC*), 129.9 (*ArC*), 130.9, (*ArC*), 136.6 (4ry *C*), 138.2 (*C*(2)), 140.3 (4ry *C*), 144.4 (4ry *C*), 144.9 (4ry *C*), 171.0 (*C*(1)); *m/z* (NSI$^+$) 432 ([M+H]$^+$, 5%); HRMS (NSI$^+$) $C_{18}H_{17}F_3NO_4S_2^+$ ([M+H]$^+$) requires 432.0546; found 432.0543 (−0.6 ppm).

4-Methyl-*N*-[(1*E*)-4,4,4-trifluoro-1-(naphthalene-2-yl)but-2-en-1-ylidene]benzene-1-sulfonamide

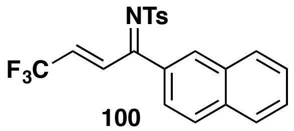

100

Following general procedure **D**, trifluoromethyl enone **79** (2.00 g, 8.00 mmol) amd 4-toluenesulfonamide (1.37 g, 8.00 mmol) in CH_2Cl_2 (50 mL), Et_3N (2.20 mL, 16.0 mmol) and $TiCl_4$ (0.88 mL, 8.00 mmol) gave, after recrystallisation (Et_2O/Petrol), ketimine **100** as yellow solid (1.97 g, 61%): mp 120-122 °C; ν_{max} (ATR)/cm^{-1} 1287, 1288 (SO_2), 1550 (C=N), 3067 (C-H); δ_H (500 MHz, $CDCl_3$) 2.46 (3 H, s, SO_2ArC*H₃*), 6.18 (1 H, dq, *J* 16.3, 6.1, C(3)*H*), 7.37 (2 H, d, *J* 7.1, SO_2Ar(3,5)*H*), 7.58-7.65 (2 H, m, Np*H*), 7.80-8.00 (7 H, m, Np*H* and SO_2Ar(2,6)*H*), 8.20 (C(2)*H*); δ_C (125 MHz, $CDCl_3$) 21.7 (SO_2Ar*C*H_3), 121.9 (q, *J* 271, *C*F_3), 124.9 (*NpC*H), 127.3 (*NpC*H), 127.5 (*NpC*H and *NpC*H), 127.9 (*NpC*H), 128.9 (*C*(3)H), 129.2 (*NpC*H), 129.6 (SO_2*ArC*(2,6)H), 129.7 (SO_2*ArC*(3,5)H and *NpC*H), 132.3 (*NpC* and *NpC*), 137.5 (SO_2*ArC*(4)), 144.3 (SO_2*ArC*(1) and *NpC*(2)) 161.2 (*C*(1)); *m/z* (NSI^+) 404 ([M+H], 100%), HRMS (NSI^+) $C_{21}H_{17}F_3NO_2S^+$ ($[M+H]^+$) requires 404.0927 found 404.0924 (−0.6 ppm).

***N*-((*E*)-1-(Furan-2-yl)-4,4,4-trifluorobut-2-en-1-ylidine)-4-methylbenzenesulfonamide**

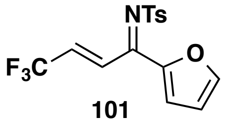

101

Following general procedure **D** trifluoromethyl enone **80** (2.48 g, 13.1 mmol) and 4-toluenesulfonamide (2.23 g, 13.1 mmol) in CH_2Cl_2 (60 mL), Et_3N (3.65 mL, 26.1 mmol) and $TiCl_4$ (1.44 mL, 13.1 mmol) gave, after recrystallisation (Et_2O:Petrol), ketimine **101** as a light brown solid (2.79 g, 62%); mp 80-82 °C; ν_{max} (ATR)/cm^{-1} 1305, 1539, 1566, 1595, 2989 (S=O), 1146 (S=O), 3110 (C-H); δ_H (300 MHz, $(CD_3)_2S$=O, 80 °C) 2.32 (3 H, s, C*H₃*), 6.61-6.73 (2 H, m, C(3)*H* and C(1)Ar(4)*H*), 7.35 (2 H, d, *J* 8.0, SO_2Ar(3,5)*H*), 7.40 (1 H, d, *J* 3.7, C(1)Ar(3)*H*), 7.52 (1 H, dq, *J* 16.3, 2.1, C(2)*H*), 7.73 (2 H, d, *J* 8.3, SO_2Ar(2,6)*H*), 8.04 (1 H, d, *J* 1.1, C(1)Ar(5)*H*); δ_C (75 MHz, $(CD_3)_2S$=O, 80 °C) 21.4 (*C*H_3), 114.6 (C(1)*ArC*(4)), 122.8 (q, *J* 269, *C*F_3), 125.5 (C(1)*ArC*(3)), 126.7 (q, *J* 34.0, *C*(3)), 127.3 (SO_2*ArC*(2,6)), 130.2 (SO_2*ArC*(3,5)), 132.1 (q, *J* 7.1, *C*(2)), 138.3 (SO_2*ArC*(1)), 144.4 (SO_2*ArC*(4)), 149.4 (C(1)*ArC*(2)), 151.6 (C(1)*ArC*(5)) 159.7 (*C*(1)); *m/z* ($APCI^+$) 344 ($[M+H]^+$, 100%); HRMS (NSI^+) $C_{15}H_{13}F_3NO_3S^+$ ($[M+H]^+$) requires 344.0563; found 344.0561 (−0.5 ppm).

4-Methyl-*N*-[(2*E*)-4,4,4-trifluoro-1-(thiophen-2-yl)but-2-en-1-ylidene]benzene-1-sulfonamide

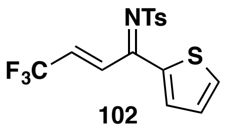

102

Following general procedure **D**, trifluoromethyl enone **81** (1.9 g, 6.8 mmol) and 4-toluenesulfonamide (1.16 g, 6.80 mmol) in CH_2Cl_2 (50 mL), Et_3N (1.88 mL, 13.6 mmol) and $TiCl_4$ (0.75 mL, 6.8 mmol) gave, after recrystallisation (Et_2O/Petrol), ketimine **102** as orange solid (2.0 g, 80%): mp 102-104 °C; ν_{max} (ATR)/cm^{-1} 1288 (S=O), 1533, 3086 (C-H); δ_H (500 MHz, $CDCl_3$); 2.45 (3 H, s, SO_2ArC*H₃*), 6.12 (1 H, dq, *J* 16.2, 6.0, C(3)*H*), 7.16 (1 H, dd, *J* 5.02, 3.89, C(1)Ar(4)*H*), 7.35 (2 H, d, *J* 8.1, SO_2ArC(3,5)*H*), 7.63 (1 H, d, *J* 3.9, C(1)Ar(3)*H*), 7.70-7.75 (3 H, m, C(1)Ar(5)*H* and C(2)*H*), 7.88 (2 H, d, *J* 8.30, SO_2ArC(2)*H*); δ_C (125 MHz, $CDCl_3$); 21.7 (SO_2Ar*C*H_3), 121.7 (q, *J* 270, *C*F_3), 127.4 (SO_2*ArC*(2,6)H), 127.8 (*C*(3)H), 128.9 (C(1)*ArC*(4)H), 129.6 (SO_2*ArC*(3,5)H), 131.6 (C(1)*ArC*(5)H), 136.6 (C(1)*ArC*(3)H), 136.9 (*C*(2)H), 137.3 (SO_2*ArC*(4)), 144.22 (C(1)*ArC*(2) and SO_2*ArC*(1)), 165.6 (*C*(1)); *m/z* (NSI^+) 360 ([M+H], 100%); HRMS (NSI^+) $C_{15}H_{13}F_3NO_2S_2^+$ ($[M+H]^+$) requires 360.0334 found 360.0336 (0.5 ppm).

4-Methyl-*N*-((*E*)-1,1,1-trifluoro-4-phenylbut-3-en-2-ylidene)benzenesulfonamide

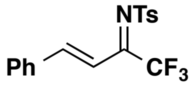

103

Following general procedure **D**, (3*E*)-1,1,1-trifluoro-4-phenylbut-3-en-2-one (1.50 g, 7.50 mmol) and 4-toluenesulfonamide (1.29 g, 7.50 mmol) in CH_2Cl_2 (20 mL), Et_3N (2.10 mL, 15.0 mmol) and $TiCl_4$ (0.83 mL, 7.50 mmol) gave, after recrystallisation (EtOAc:Petrol), ketimine **103** as a light yellow solid (404 mg, 19%); mp 140-142 °C; ν_{max} (ATR)/cm^{-1} 1146 (S=O), 1306, 1574, 1585, 1608 (S=O), 3053 (C-H); δ_H (500 MHz, $CDCl_3$) 2.49 (3H, s, C*H*$_3$), 7.40 (2H, d, *J* 8.1, SO_2Ar(3,5)*H*), 7.48-7.55 (3H, m, C(4)Ar(3,4,5)*H*), 7.66-7.70 (3 H, m, C(4)Ar(2,6)*H* and =C*H*), 7.87 (1 H, dd, *J* 16.7, 1.6, =C*H*), 7.94 (2 H, d, *J* 8.3, SO_2Ar(2,6)*H*); δ_C (100 MHz, $CDCl_3$) 21.7 (*C*H_3), 116.0 (*C*(4)), 119.1 (q, *J* 280, *C*F_3), 127.5 (*ArC*), 129.3 (*ArC*), 129.4 (*ArC*), 129.8 (*ArC*), 132.5 (C(4)*ArC*(4)), 133.9 (C(4)*ArC*(1)), 137.0 (SO_2*ArC*(4)), 144.7 (SO_2*ArC*(1)), 149.2 (q, *J* 2.5, *C*(3)), 161.2 (q, *J* 33.8, *C*(2)); *m/z* ($APCI^+$) 354 ($[M+H]^+$, 100%); HRMS ($APCI^+$) $C_{17}H_{14}F_3NO_2S^+$ ($[M+H]^+$) requires 354.0770; found 354.0766 (-1.2 ppm).

1.5 Pyridine Examples – Ketimine Variation

Ethyl 2-phenyl-6-((phenylsulfonyl)oxy)isonicotinate

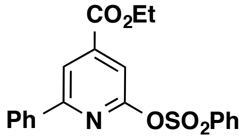

7

Following general procedure **E**, (phenylthio)acetic acid (101 mg, 0.6 mmol) in THF (6 mL), *i*-Pr_2NEt (157 μL, 0.9 mmol), pivaloyl chloride (110 μL, 0.9 mmol), ketimine **5** (206 mg, 0.6 mmol), DHPB **6** (22 mg, 0.12 mmol), and *i*-Pr_2NEt (101 μL, 0.6 mmol) for 4 h at rt followed by heating at reflux for 16 h gave, after chromatographic purification (eluent; EtOAc:Petrol, 10:90), pyridine **7** as a yellow solid (155 mg, 67%), sample was recrystallised (Et_2O:Petrol) for analysis; mp 119-120 °C; ν_{max} (ATR)/cm^{-1} 1362, 1558 (S=O); 1724 (C=O); 2938, 2980 (C-H); δ_H (400 MHz, $CDCl_3$) 1.43 (3 H, t, *J* 7.1, CH_2CH_3), 4.45 (2 H, q, *J* 7.1 CH_2CH_3), 7.39-7.44 (3 H, m, Ar(3,5)*H* and Ar(4)*H*), 7.55-7.61 (3 H, m, SO_2Ar(3,5)*H* and SO_2Ar(4)*H*), 7.68-7.77 (3 H, m, py(3)*H*, Ar(2,6)*H*), 8.06-8.10 (2 H, m, SO_2Ar(2,6)*H*), 8.21 (1 H, d, *J* 1.06, py(5)*H*); δ_C (100 MHz, $CDCl_3$) 14.2 (CH_3), 62.3 (CH_2), 113.4 (*pyC*(3)), 118.2 (*pyC*(5)), 127.0 (py(6)*ArC*(4)H), 128.8 (py(6)*ArC*(2,6)H and py(6)*ArC*(3,5)H), 129.1 (SO_2*ArC*(2,6)H), 130.0 (SO_2*ArC*(3,5)H), 134.1 (SO_2*ArC*(4)H), 136.6 (SO_2*ArC*(1)), 137.3 (py(6)*ArC*(1)), 143.0 (*pyC*(6)), 157.2 (*pyC*(4)), 157.4 (*pyC*(2)), 163.9 (*C*(1)); *m/z* (NSI$^+$) 384 ([M+H], 100%); HRMS (NSI$^+$) $C_{20}H_{18}NO_5S^+$ ([M+H]$^+$) requires 384.0900 found 384.0899 (−0.3 ppm).

Benzyl 2-([(4-methylbenzene)sulfonyl]oxy)-6-phenylpyridine-4-carboxylate

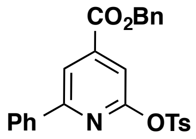

11

Following general procedure **E**, (phenylthio)acetic acid (101 mg, 0.6 mmol) and *i*-Pr_2NEt (157 μL, 0.9 mmol) in THF (9 mL) were added pivaloyl chloride (110 μL, 0.9 mmol) DHPB **6** (22 mg, 0.12 mmol, 20 mol%), ketimine **82** (252 mg, 0.6 mmol) and *i*-Pr_2NEt (110 μL, 0.6 mmol) for 4 hours at rt followed by heating at reflux for 16 h gave, after chromatographic purification (eluent Et_2O:petrol 10:90), pyridine **11** as a white solid (166 mg, 60%), sample was recrystallised (Et_2O:Petrol) for analysis; mp 64-66 °C; ν_{max} (ATR)/cm^{-1} 1168, 1362 (S=O), 1734 (C=O), 3068 (Ar-H); δ_H (500 MHz, $CDCl_3$) 2.49 (3 H, s, SO_2ArCH_3), 5.55 (2 H, s, CO_2CH_2Ph), 7.35-7.48 (10 H, m, Ar*H*), 7.58 (1 H, s, pyC(3)*H*), 7.78 (2 H, m, SO_2ArC(3,5)*H*), 7.96 (2 H, d, *J* 8.40, py(6)Ar(2,6)*H*), 8.24 (1 H, m, py(5)*H*), δ_C (125 MHz,

$CDCl_3$); 21.8 (SO_2ArCH_3), 67.9 (CO_2CH_2Ph), 113.5 (*pyC*(5)H), 118.2 (*pyC*(3)H), 127.0 (SO_2*ArC*(2,6)H), 128.6 (C(1)*Ar*(2,6)H), 128.7-128.8 py(6)*ArC*(4)H, py(6)*ArC*(2,6)H and C(1)*Ar*(4)H), 129.7 (C(1)*ArC*(3,5)H), 130.1 (SO_2*ArC*(3,5)H), 134.2 (SO_2*ArC*(1)), 135.0 (C(1)*ArC*(1)), 136.6 (SO_2*ArC*(4)), 142.6 (py(6)*ArC*(1)), 145.3 (*pyC*(6)), 157.2 (*pyC*(4)), 157.6 (*pyC*(2)), 163.9 (*C*(1)); *m/z* (NSI^+) 460 ([M+H], 100%); HRMS (NSI^+) $C_{26}H_{22}NO_5S^+$ ($[M+H]^+$) requires 460.1213 found 460.1200 (−2.9 ppm).

Benzyl 2-(4-methylphenyl)-6-([(4-methylbenzene)sulfonyl]oxy)pyridine-4-carboxylate

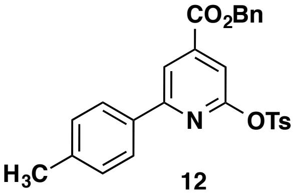

12

Following general procedure **E**, (phenylthio)acetic acid (101 mg, 0.6 mmol) and *i*-Pr_2NEt (157 μL, 0.9 mmol) in THF (9 mL) were added pivaloyl chloride (110 μL, 0.9 mmol) DHPB **6** (22 mg, 0.12 mmol, 20 mol%), ketimine **83** (270 mg, 0.6 mmol) and *i*-Pr_2NEt (110 μL, 0.6 mmol) for 4 hours at rt followed by heating at reflux for 16 h gave, after chromatographic purification (eluent Et_2O:petrol 10:90), pyridine **12** as a white solid (166 mg, 60%), sample was recrystallised (Et_2O:Petrol) for analysis; mp 127-130 °C; ν_{max} (ATR)/cm^{-1} 1171 (C-O), 1367 (SO_2), 1722 (C=O), 3088 (Ar-H); δ_H (500 MHz, $CDCl_3$) 2.40 (3 H, s, py(6)ArC*H₃*), 2.47 (3 H, s, SO_2ArCH_3), 5.41 (2 H, s, CO_2CH_2Ph), 7.21 (2 H, d , *J* 8.72, py(6)Ar(3,5)*H*), 7.35-7.47 (7 H, m, Ar*H*), 7.55 (1 H, s, py(5)*H*), 7.66 (2 H, d, *J* 7.6, SO_2ArC(2,6)*H*), 7.94 (2 H, d, *J* 7.6, py(6)Ar(2,6)*H*) 8.19 (1 H, s, py(3)*H*); δ_C (125 MHz, $CDCl_3$) 21.4 ($ArCH_3$), 21.7 (SO_2ArCH_3), 67.9 (CO_2CH_2Ph), 113.0 (*pyC*(5)H), 117.9 (*pyC*(3)H), 126.9 (SO_2*ArC*(2,6)H), 128.5 (Ar*C*H), 128.7 (Ar*C*H), 128.8 (py(6)*Ar*(2,6)H), 128.9 (Ar*C*H) 129.5 (py(6)*Ar*(3,5)H), 129.7 (Ar*C*H), 133.9 (SO_2*ArC*(1)), 134.2 (py(6)*Ar*(4)), 135.0 (C(1)*Ar*(1)), 140.4 (SO_2*ArC*(4)), 142.4 (py(6)*Ar*(1)), 145.2 (*pyC*(6)), 157.3 (*pyC*(4)), 157.4 (*pyC*(2)), 163.9 (*C*(1)); *m/z* ($APCI^+$) 474 ([M+H], 100%); HRMS ($APCI^+$) $C_{27}H_{24}NO_5S^+$ ($[M+H]^+$) requires 474.1370 found 474.1369 (−0.1 ppm).

Benzyl 2-{[(4-methylbenzene)sulfonyl]oxy}-6-(4-nitrophenyl)pyridine-4-carboxylate

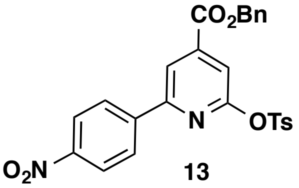

13

Following general procedure **E**, (phenylthio)acetic acid (101 mg, 0.6 mmol) and *i*-Pr_2NEt (157 μL, 0.9 mmol) in THF (9 mL) were added pivaloyl chloride (110 μL, 0.9 mmol) DHPB **6** (22 mg, 0.12 mmol, 20 mol%), crude ketimine **84** (464 mg, 0.6 mmol) and *i*-Pr_2NEt (110 μL, 0.6 mmol) for 4 hours at rt followed by heating at reflux for 16 h gave, after chromatographic purification (eluent Et_2O:petrol 15:85), pyridine **13** as orange solid (303 mg, 51 %), sample was recrystallised (Et_2O:Petrol) for analysis; mp 124-127 °C; ν_{max} (ATR)/cm^{-1} 1165 (C-O), 1327 (SO_2), 1365, 1526 ($ArNO_2$), 1728 (C=O), 3062 (Ar-H); δ_H (500 MHz, $CDCl_3$) 2.49 (3 H, s, SO_2ArCH_3), 5.43 (2 H, s, CO_2CH_2Ph), 7.36-7.47 (7 H, m , Ar*H*), 7.67 (1 H, s, py(5)*H*), 7.92-7.97 (4 H, m, Ar*H*), 8.25-8.27 (3 H, m, Ar*H*); δ_C (125 MHz, $CDCl_3$) 21.8 (SO_2ArCH_3), 68.2 (CO_2CH_2Ph), 115.1 (*pyC*(5)H), 118.9 (*pyC*(3)H), 124.0 (*ArC*H), 127.9 (*ArC*H), 128.8-128.9 (*ArC*H), *ArC*H), *ArC*H, *ArC*H)), 129.8 (*ArC*H), 134.1 (SO_2*ArC*(1)), 134.7 (C(1)*Ar*(1)), 142.4 (SO_2*ArC*(4)), 143.1 (py(6)*Ar*(1)), 145.6 (py(6)*Ar*(4)), 148.7 (*pyC*(6)), 154.5 (*pyC*(4)), 157.9 (*pyC*(2)), 163.4 (C(1)); *m/z* (NSI^+) 505 ([M+H], 100%); HRMS (NSI^+) $C_{26}H_{21}N_2O_7S^+$ ($[M+H]^+$) requires 505.1064 found 505.1056 (−1.6 ppm).

Benzyl 2-(4-methoxyphenyl)-6-([(4-methylbenzene)sulfonyl]oxy)pyridine-4-carboxylate

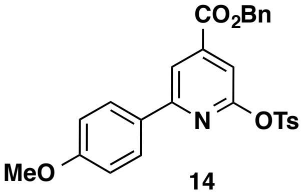

14

Following general procedure **E**, (phenylthio)acetic acid (101 mg, 0.6 mmol) and *i*-Pr_2NEt (157 μL, 0.9 mmol) in THF (9 mL) were added pivaloyl chloride (110 μL, 0.9 mmol) DHPB **6** (22 mg, 0.12 mmol, 20 mol%), ketimine **85** (270 mg, 0.6 mmol) and *i*-Pr_2NEt (110 μL, 0.6 mmol) for 4 hours at rt followed by heating at reflux for 16 h gave, after chromatographic purification (eluent Et_2O:petrol 10:90), pyridine **14** as an orange solid (150 mg, 51%), sample was recrystallised (Et_2O:Petrol) for analysis; mp 129-131 ^oC; ν_{max} (ATR)/cm^{-1} 1165 (C-O), 1084, 1251 (ArOMe), 1552 (SO_2), 1726 (C=O), 2936, 2995 (Ar-H); δ_H (500 MHz, $CDCl_3$) 2.46 (3 H, s, OCH_3), 3.86 (3 H, s, SO_2ArCH_3), 5.40 (2 H, s, CO_2CH_2Ph), 6.90 (2 H, d , *J* 8.72, py(6)Ar(3,5)*H*), 7.34-7.50 (8 H, m, Ar*H*), 7.70 (2 H, d, *J* 8.6, SO_2ArC(2,6)*H*), 7.93 (2 H, d, *J* 8.4, py(6)Ar(2,6)*H*), 8.14 (1 H, s, pyC(3)*H*); δ_C (125 MHz, $CDCl_3$) 21.8 (SO_2ArCH_3), 55.4

(OCH_3), 67.9 (CH_2), 112.4 (*pyC*(5)H), 114.1 (*pyC*(3)H), 117.3 (py(6)*Ar*(3)H), 128.5 (SO_2*ArC*(2)H), 128.7-128.8 (C(1)*ArC*(2,6)H, C(1)*ArC*(3,5)H, C(1)*Ar*(4)H, py(6)*Ar*(2)H), 129.3 (SO_2*ArC*(1)), 129.7 (SO_2*ArC*(3,5)H), 134.2 (py(6)*Ar*(1)), 135.0 (C(1)*Ar*(1)), 142.4 (SO_2*ArC*(4)), 145.2 (*pyC*(6)), 157.0 (*pyC*(4)), 157.5 (py(6)*Ar*(4)), 161.3 (*pyC*(2)), 164.0 (*C*(1)); *m/z* ($APCI^+$) 490 ([M+H], 100%); HRMS ($APCI^+$) $C_{27}H_{24}NO_6S^+$ ($[M+H]^+$) requires 490.1319 found 490.1311 (−1.6 ppm).

Methyl 2-{[(4-methylbenzene)sulfonyl]oxy}-6-phenylpyridine-4-carboxylate

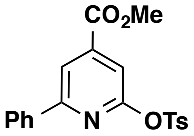

15

Following general procedure **E**, (phenylthio)acetic acid (4.41 g, 26.2 mmol) in THF (400 mL), *i*-Pr_2NEt (6.85 mL, 39.3 mmol), pivaloyl chloride (4.73 mL, 39.3 mmol), ketimine **86** (9.0 mg, 26.2 mmol), DHPB **6** (1.00 g, 5.24 mmol), and *i*-Pr_2NEt (4.57 mL, 26.2 mmol) for 4 h at rt followed by heating at reflux for 16 h gave, after chromatographic purification (eluent; EtOAc:Petrol, 10:90), pyridine **15** as a red solid (5.00 g, 50%), sample was recrystallised (Et-$_2$O:Petrol) for analysis; mp 118-120 ^oC; ν_{max} (ATR)/cm^{-1} 1169, 1364 (S=O), 1557, 1730 (C=O), 2953 (Ar-H); ; δ_H (400 MHz, $CDCl_3$) 2.47 (3 H, s, SO_2ArC*H₃*), 3.99 (3 H, s, OC*H₃*), 7.36 (2 H, d, *J* 8.04, SO_2Ar(3,5)*H*), 7.40-7.43 (3 H, m, Ar(3,5)*H* and Ar(4)*H*), 7.58 (1 H, d, *J* 1.0, py(3)*H*), 7.76-7.78 (2 H, m, Ar(2,6)*H*), 7.94 (2 H, d, *J* 8.3, SO_2Ar(2,6)*H*), 8.21 (1 H, d, *J* 1.0, py(5)*H*); δ_C (100 MHz, $CDCl_3$) 21.8 (SO_2ArCH_3), 53.1 (OCH_2), 113.5 (*pyC*(3)), 118.1 (*pyC*(5)), 127.0 (py(6)*ArC*(2,6)H), 128.8 (SO_2*ArC*(2,6)H), 129.7 (SO_2*ArC*(3,5)H), (py(6)*ArC*(3,5)H), 130.1 (py(6)*ArC*(4)H), 134.1 (SO_2*ArC*(4)), 136.6 (SO_2*ArC*(1)), 142.5 (py(6)*ArC*(1)), 145.3 (*pyC*(6)), 157.2 (*pyC*(4)), 157.6 (*pyC*(2)), 164.5 (*C*(1)); *m/z* (NSI^+) 384 ([M+H], 100%), HRMS (NSI^+) $C_{20}H_{18}NO_5S^+$ ($[M+H]^+$) requires 384.0900 found 384.0901 (+0.2 ppm).

Methyl 2-(4-cyanophenyl)-6-{[(4-methylbenzene)sulfonyl]oxy}pyridine-4-carboxylate

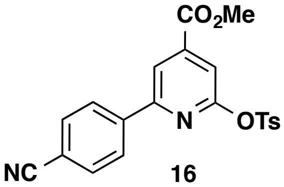

16

Following general procedure **E**, (phenylthio)acetic acid (456 mg, 2.71 mmol) and *i*-Pr_2NEt (0.71 mL, 4.10 mmol) in THF (40 mL) were added pivaloyl chloride (0.5 mL, 4.10 mmol) DHPB **6** (103 mg, 0.54 mmol, 20 mol%), crude ketimine **87** (1.00 g, 2.71 mmol) and *i*-Pr_2NEt (0.47 mL, 2.71 mmol) for 4 hours at rt followed by heating at reflux for 16 h gave, after

chromatographic purification (eluent Et_2O:petrol 20:80), pyridine **16** as a brown solid (664 mg, 60 %), sample was recrystallised (Et_2O:Petrol) for analysis; mp 154-156 °C; ν_{max} (ATR)/cm^{-1} 1165 (C-O), 1370 (SO_2), 1734 (C=O), 2224 (ArCN) δ_H (400 MHz, $CDCl_3$) 2.49 (3 H, s, SO_2ArCH_3), 4.00 (3 H, s, CO_2CH_3), 7.38 (2 H, d, *J* 8.56, SO_2Ar(3)*H*), 7.65 (1 H, d, *J* 1.0, py(3,5)*H*), 7.71 (Ar(2,6)*H*), 7.92 (4 H, m, SO_2Ar(2,6)*H,* Ar(3,5)*H*), 8.24 (1 H, d, *J* 1.0, py(5)*H*); δ_C (100 MHz, $CDCl_3$) 21.8 (SO_2ArCH_3), 53.3 (O*C*H_3), 113.6 (*ArC*(4)), 114.9 (*pyC*(3)H), 118.4 (Ar(4)*C*N), 118.6 (*pyC*(5)H), 127.5 (*ArC*(2,6)H), 128.7 (SO_2*ArC*(2,6)H), 129.8 (SO_2*ArC*(3,5)H), 132.6 (*ArC*(3,5)H), 134.0 (SO_2*ArC*(1)), 140.7 (SO_2*ArC*(4)), 143.0 (Ar*C*(1)), 145.6 (*pyC*(6)), 154.9 (*pyC*(4)), 157.8 (*pyC*(2)), 164.0 (*C*(1)); *m/z* (NSI^+) 409 ([M+H], 100%); HRMS (NSI^+) $C_{21}H_{17}N_2O_5S^+$ ($[M+H]^+$) requires 409.0853 found 409.848 (−1.1 ppm).

Methyl 2-{[(4-methylbenzene)sulfonyl]oxy}-6-[4-(trifluoromethyl)phenyl] pyridine-4-carboxylate

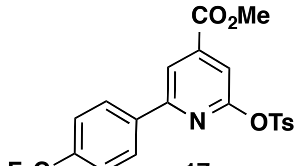

17

Following general procedure **E**, (phenylthio)acetic acid (168 mg, 1.00 mmol) and *i*-Pr_2NEt (260 μL, 1.50 mmol) in THF (15 mL) were added pivaloyl chloride (180 μL, 1.50 mmol) DHPB **6** (38 mg, 0.2 mmol, 20 mol%), crude ketimine **88** (411 mg, 1.00 mmol) and *i*-Pr_2NEt (170 μL, 1.00 mmol) for 4 hours at rt followed by heating at reflux for 16 h gave, after chromatographic purification (eluent Et_2O:petrol 15:85), pyridine **17** as a white solid (288 mg, 64%), sample was recrystallised (Et_2O:Petrol) for analysis; mp 108-110 °C; ν_{max} (ATR)/cm^{-1} 1377 (SO_2), 1732 (C=O), 3094 (C-H); δ_H (500 MHz, $CDCl_3$) 2.48 (3 H, s, SO_2ArCH_3), 4.00 (3 H, s, OCH_3), 7.37 (2 H, d, *J* 8.13, SO_2Ar(3,5)*H*), 7.63 (1 H, s, py(3)*H*), 7.67 (2 H, d, *J* 8.2, Ar(2,6)*H*), 7.89 (2 H, d, *J* 8.2, Ar(3,5)*H*), 7.93 (2 H, d, *J* 8.3, SO_2Ar (2,6)*H*), 8.24 (1 H, s, py(5)*H*); δ_C (125 MHz, $CDCl_3$) 21.8 (SO_2ArCH_3), 53.2 (*C*H_3), 114.5 (*pyC*(3)H), 118.5 (*pyC*(5)H), 123.8 (q, *J* 272, *C*F_3), 125.7 (*ArC*(3,5)H), 127.7 (*ArC*(2,6)H), 128.7 (SO_2*ArC*(2,6)H), 129.7 (SO_2*ArC*(3,5)H), 131.8 (*ArC*(4)), 134.0 (SO_2*ArC*(4)), 139.9 (SO_2*ArC*(1)), 142.8 (*ArC*(1)), 145.5 (*pyC*(6)), 155.5 (*pyC*(4)), 157.7 (*pyC*(2)), 164.2 (*C*(1)); δ_F (470 MHz, $CDCl_3$); −62.8 (CF_3); *m/z* (NSI^+) 452 ([M+H], 100%); HRMS (NSI^+) $C_{21}H_{17}F_3NO_5S^+$ ($[M+H]^+$) requires 452.0774 found 452.0766 (−1.8 ppm).

Methyl 2-(4-chlorophenyl)-6-([(4-methylbenzene)sulfonyl]oxy)pyridine-4-carboxylate

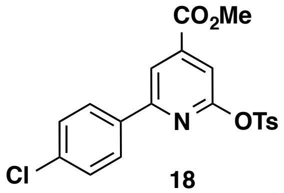

18

Following general procedure **E**, (phenylthio)acetic acid (101 mg, 0.6 mmol) and *i*-Pr_2NEt (157 μL, 0.9 mmol) in THF (9 mL) were added pivaloyl chloride (110 μL, 0.9 mmol) DHPB **6** (22 mg, 0.12 mmol, 20 mol%), ketimine **89** (0.227 g, 0.6 mmol) and *i*-Pr_2NEt (110 μL, 0.6 mmol) for 4 hours at rt followed by heating at reflux for 16 h gave, after chromatographic purification (eluent Et_2O:petrol 10:90), pyridine **18** as a white solid (130 mg, 54%), sample was recrystallised (Et_2O:Petrol) for analysis; mp 120-122 °C; ν_{max} (ATR)/cm^{-1} 1086 (ArC(4)) 1171, 1181 (SO_2), 1734 (C=O), 2956, 3096 (Ar-H); δ_H (500 MHz, $CDCl_3$) 2.47 (3 H, s, C*H₃*), 3.98 (3 H, s, SO_2ArC*H₃*), 7.36-7.37 (4 H, m, Ar(3,5)*H*, SO_2Ar(3,5)*H*), 7.57 (1 H, s, py(3)*H*), 7.70 (2 H, d, *J* 8.0, SO_2Ar(2,6)*H*), 7.92 (2 H, *J* 7.7, Ar(2,6)*H*), 8.16 (1 H, s, py(5)*H*); δ_C (125 MHz, $CDCl_3$) 21.8 (*C*H_3), 53.1 (SO_2Ar*C*H_3), 113.7 (*pyC*(5)H), 117.9 (*pyC*(3)H), 128.3 (SO_2*ArC*(2,6)H), 128.8 (py(6)*ArC*(2,6)H), 129.0 (py(6)*Ar*(3,5)H), 129.7 (SO_2*ArC*(3,5)H), 134.1 (SO_2*ArC*(1)), 135.1 (py(6)*ArC*(4)Cl), 136.3 (py(6)*ArC*(1)), 142.7 (SO_2*ArC*(4)), 145.4 (*pyC*(6)), 155.9 (*pyC*(4)), 157.6 (*pyC*(2)), 164.3 (*C*(1)); *m/z* ($APCI^+$) 418 ([M+H], 100%); HRMS ($APCI^+$) $C_{20}H_{17}ClNO_5S^+$ ($[M+H]^+$) requires 418.0510 found 418.0510 (−0.1 ppm).

Methyl 2-(2-fluorophenyl)-6-{[(4-methylbenzene)sulfonyl]oxy}pyridine-4-carboxylate

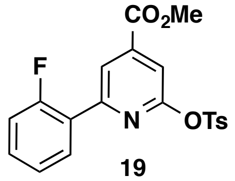

19

Following general procedure **E**, (phenylthio)acetic acid (234 mg, 1.39 mmol) and *i*-Pr_2NEt (360 μL, 2.09 mmol) in THF (21 mL) were added pivaloyl chloride (260 μL, 2.09 mmol) DHPB **6** (53 mg, 0.28 mmol, 20 mol%), crude ketimine **90** (500 mg, 1.39 mmol) and *i*-Pr_2NEt (240 μL, 1.39 mmol for 4 hours at rt followed by heating at reflux for 16 h gave, after chromatographic purification (eluent Et_2O:petrol 10:90), pyridine **19** as yellow solid (375 mg, 65%), sample was recrystallised (Et_2O:Petrol) for analysis; mp 86-88 °C; ν_{max} (ATR)/cm^{-1}1368 (SO_2), 1735 (C=O), 2974 (C-H); δ_H (500 MHz, $CDCl_3$) 2.46 (3 H, s, SO_2ArC*H₃*), 3.98 (3 H, s, OC*H₃*), 7.13-7.18 (2 H, m, Ar(3)*H* and Ar(5)*H*), 7.35 (2 H, d, *J* 8.2, SO_2Ar(3,5)*H*), 7.37-7.42 (1 H, m, Ar(4)*H*), 7.57-7.61 (2 H, m, py(3)*H* and Ar(6)*H*), 7.93 (2 H, d, *J* 8.3, SO_2Ar(2,6)*H*), 8.31 (1 H, s, py(5)*H*); δ_C (125 MHz, $CDCl_3$) 21.8 (SO_2Ar*C*H_3), 53.1 (O*C*H_3),

113.9 (*pyC*(3)H), 116.4 (d, *J* 23.0, *ArC*(3)H), 122.4 (d, *J* 12.3, *pyC*(5)H), 124.4 (d, *J* 3.42, *ArC*(5)H), 124.8 (d, *J* 10.6, *ArC*(1)), 128.8 (SO_2*ArC*(2,6)H), 129.7 (SO_2*ArC*(3,5)H), 131.0 (d, *J* 1.96 (*ArC*(6)H), 131.6 (d, *J* 8.74, *ArC*(4)H), 134.0 (SO_2*ArC*(4)), 142.4 (SO_2*ArC*(1)), 145.4 (*pyC*(6)), 153.0 (*pyC*(4)), 157.4 (*pyC*(2)), 160.7 (d, *J* 254, *ArC*(2)), 164.4 (*C*(1)); δ_F (470 MHz, $CDCl_3$); −115.4 (Ar*F*); *m/z* (NSI^+) 402 ($[M+H]^+$, 100%); HRMS (NSI^+) $C_{20}H_{17}FNO_5S^+$ ($[M+H]^+$) requires 402.0806 found 402.0803 (−0.7 ppm).

Methyl 2-{[(4-methylbenzene)sulfonyl]oxy}-6-(naphthalene-2-yl)pyridine-4-carboxylate

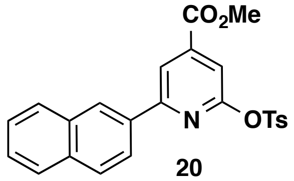

20

Following general procedure **E**, (phenylthio)acetic acid (427 mg, 2.54 mmol) and *i*-Pr_2NEt (0.66 mL, 3.81 mmol) in THF (40 mL) were added pivaloyl chloride (0.74 mL, 3.81 mmol) DHPB **6** (97 mg, 0.51 mmol, 20 mol%), crude ketimine **91** (1.00 g, 2.54 mmol) and *i*-Pr_2NEt (0.44 mL, 2.54 mmol) for 4 hours at rt followed by heating at reflux for 16 h gave, after chromatographic purification (eluent Et_2O:petrol 10:90), pyridine **20** as a green solid (760 mg, 69 %), sample was recrystallised (Et_2O:Petrol) for analysis; mp 142-144 °C; ν_{max} (ATR)/cm^{-1} 1171 (C-O), 1369 (SO_2), 1732 (C=O), 2951 (C-H) δ_H (400 MHz, $CDCl_3$) 2.49 (3 H, s, SO_2ArC*H₃*), 4.02 (3 H, s, CO_2C*H₃*), 7.39 (2 H, d, *J* 8.0, SO_2Ar(3,5)*H*), 7.52-7.56 (2 H, m, *NpH*), 7.61 (1 H, d, *J* 1.0, py(5)*H*), 7.85-7.87 (4 H, m, *NpH*), 7.98 (2 H, d, *J* 8.4, SO_2Ar (2)*H*), 8.26 (1 H, s, py(3)*H*), 8.35 (1 H, d, *J* 1.05, *Np*(1)*H*); δ_C (100 MHz, $CDCl_3$) 21.8 (SO_2Ar*C*H_3), 53.1 (O*C*H_3), 113.5 (*pyC*(5)H), 118.4 (*pyC*(3)H), 124.1 (*NpC*H), 126.6 (Np*C*H), 127.0 (*NpC*H), 127.2 (*NpC*H), 127.7 (*NpC*H), 128.5 (*NpC*H), 128.8 (*NpC*H), 128.9 (SO_2*ArC*(2,6)H), 129.8 (SO_2*ArC*(3,5)H), 133.2 (*NpC*(10)), 133.9 (*NpC*(5)), 134.1 (SO_2*ArC*(1)), 134.3 (*NpC*(2)), 142.5 (SO_2*ArC*(4)), 145.3 (*pyC*(6)), 157.2 (*pyC*(4)), 157.7 (*pyC*(2)), 164.5 (*C*(1)); *m/z* (NSI^+) 434 ([M+H], 100%); HRMS (NSI^+) $C_{24}H_{20}NO_5S^+$ ($[M+H]^+$) requires 434.1057 found 434.1051 (−1.3 ppm).

Methyl 2-tert-butyl-6-{[(4-methylbenzene)sulfonyl]oxy}pyridine-4-carboxylate

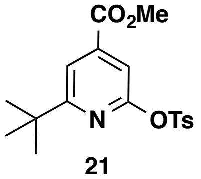

21

Following general procedure **E**, (phenylthio)acetic acid (101 mg, 0.6 mmol) and *i*-Pr_2NEt (150 μL, 0.9 mmol) in THF (9 mL) were added pivaloyl chloride (110 μL, 0.9 mmol) DHPB

6 (22.8 mg, 0.12 mmol, 20 mol%), ketimine **92** (194 mg, 0.6 mmol) and *i*-Pr_2NEt (100 μL, 0.6 mmol for 4 hours at rt followed by heating at reflux for 16 h gave, after chromatographic purification (eluent Et_2O:petrol 15:85), pyridine **21** as a brown solid (97 mg, 44%), sample was recrystallised (Petrol) for analysis; mp 72-74 °C; ν_{max} (ATR)/cm^{-1} 1172, 1323 (S=O), 1371, 1730 (C=O), 2962 (C-H); δ_H (500 MHz, $CDCl_3$) 1.17 (9 H, s, $(CH_3)_3$), 2.44 (3 H, s, SO_2ArCH_3), 3.95 (3 H, s, OCH_3), 7.33 (2 H, d, *J* 7.45, $SO_2Ar(3,5)H$), 7.48 (1 H, s, py(3)*H*), 7.75 (1 H, s, py(5)*H*), 7.87 (2 H, d, *J* 7.50, $SO_2Ar(2,6)H$); δ_C (125 MHz, $CDCl_3$) 21.7 (SO_2ArCH_3), 29.6 ($(CH_3)_3$), 37.7 (*C*(CH_3)), 52.9 (CH_3), 112.4 (*pyC*(3)H), 117.4 (*pyC*(5)H), 128.6 (SO_2*ArC*(2,6)H), 129.1 (SO_2*ArC*(3,5,)H), 134.1 (SO_2*ArC*(4)), 141.8 (SO_2*ArC*(1)), 145.1 (*pyC*(6)), 156.7 (*pyC*(4)), 164.8 (*pyC*(2)), 170.2 (*C*(1)); *m/z* (NSI^+) 364 ($[M+H]^+$, 100%); HRMS (NSI^+) $C_{18}H_{22}NO_5S^+$ ($[M+H]^+$) requires 364.1213 found 364.1214 (+0.2 ppm).

6-Phenyl-4-(trifluoromethyl)pyridin-2-yl 4-methylbenzene-1-sulfonate

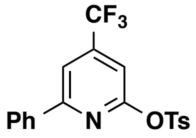

22

Following general procedure **E**, (phenylthio)acetic acid (84.1 mg, 0.50 mmol) in THF (5 mL), *i*-Pr_2NEt (130 μL, 0.75 mmol), pivaloyl chloride (92.5 μL, 0.75 mmol), ketimine **93** (177 mg, 0.5 mmol), DHPB **6** (19.0 mg, 0.1 mmol, 20 mol%) and *i*-Pr_2NEt (87.5 μL, 0.50 mmol) for 4 h at rt followed by heating at reflux for 16 h gave, after chromatographic purification (eluent Et_2O:petrol 10:90), pyridine **22** as a light yellow solid (113 mg, 58%) which was recrystallised (Et_2O:petrol) for an analytical sample; mp 100-102 °C; ν_{max} (ATR)/cm^{-1} 1616, 1595, 1564, 1306 (S=O), 1142 (S=O), 3109, 2940 (C-H); δ_H (500 MHz, $CDCl_3$) 2.51 (3H, s, CH_3), 7.26 (1H, s, C(3)*H*), 7.40 (2H, d, *J* 8.1, $SO_2Ar(3,5)H$), 7.45-7.49 (3H, m, C(6)Ar(3,4,5)*H*), 7.78-7.79 (2H, m, C(6)Ar(2,6)*H*), 7.85 (1H, s, *C*(5)*H*), 7.99 (2H, d, *J* 8.2, $SO_2Ar(2,6)H$); δ_C (100 MHz, $CDCl_3$) 21.8 (CH_3), 109.8 (q, *J* 3.4, (*C*(3)), 114.3 (q, *J* 3.3, *C*(5)), 122.2 (q, *J* 272, CF_3), 127.1 (C(6)*ArC*(2,6)), 128.9 (*ArC*), 128.9 (*ArC*), 129.8 (*ArC*), 130.5 (C(6)*ArC*(4)), 134.0 (OSO_2*ArC*(1)), 136.2 (C(6)*ArC*(1)), 143.1 (q, *J* 34.2, C(4)), 145.6 (OSO_2*ArC*(4)), 157.5 (*C*(2) or *C*(6)), 158.0 (*C*(2) or *C*(6)); δ_F (470 MHz, $CDCl_3$); −65.1 (CF_3); *m/z* ($APCI^+$) 394 ($[M+H]^+$, 100%); HRMS ($APCI^+$) $C_{19}H_{15}F_3NO_3S^+$ ($[M+H]^+$) requires 394.0719; found 394.0721 (+0.4 ppm).

Scale-up: Following general procedure **E**, (phenylthio)acetic acid (4.35 g, 25.9 mmol) in THF (250 mL), *i*-Pr_2NEt (6.73 mL, 38.9 mmol), pivaloyl chloride (4.80 mL, 38.9 mmol), ketimine **93** (9.13 g, 25.9 mmol), DHPB **6** (0.98 g, 5.18 mmol, 20 mol%) and *i*-Pr_2NEt (4.48 mL, 25.9

mmol) for 4 h at rt followed by heating at reflux for 16 h gave, after chromatographic purification (eluent Et_2O:petrol 10:90), pyridine **22** as a light yellow solid (5.18 g, 51%) with identical spectroscopic properties to above.

From (*Z*)-ketimine **94**: Following general procedure **E**, (phenylthio)acetic acid (84.1 mg, 0.50 mmol) in THF (5 mL), *i*-Pr_2NEt (130 μL, 0.75 mmol), pivaloyl chloride (92.5 μL, 0.75 mmol), ketimine **94** (177 mg, 0.5 mmol), DHPB **6** (19.0 mg, 0.1 mmol, 20 mol%) and *i*-Pr_2NEt (87.5 μL, 0.50 mmol) for 4 h at rt followed by heating at reflux for 16 h gave, after chromatographic purification (eluent Et_2O:petrol 10:90), pyridine **22** as a light yellow solid (107 mg, 54%) with identical spectroscopic properties to above.

6-(3-Bromophenyl)-4-(trifluoromethyl)pyridine-2-yl-4-methylbenzne-1-sulfonate

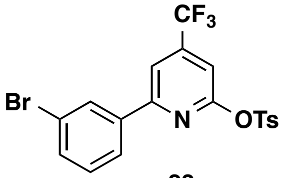

23

Following general procedure **E**, (phenylthio)acetic acid (101 mg, 0.6 mmol) and *i*-Pr_2NEt (210 μL, 1.2 mmol) in THF (9 mL) were added pivaloyl chloride (110 μL, 0.9 mmol) DHPB **6** (22 mg, 0.12 mmol, 20 mol%), ketimine **95** (259 mg, 0.6 mmol) and *i*-Pr_2NEt (110 μL, 0.6 mmol) for 4 hours at rt followed by heating at reflux for 16 h gave, after chromatographic purification (eluent Et_2O:petrol 5:95), pyridine **23** as a white solid (73 mg, 30%) which was recrystallised (Et_2O:petrol) for an analytical sample; mp 106-108 °C; ν_{max} (ATR)/cm^{-1} 1093 (Ar-Br), 1350 (SO_2), 3091 (Ar-H); δ_H (500 MHz, $CDCl_3$) 2.50 (3 H, s, SO_2ArC*H*$_3$), 7.28 (1 H, s, pyC(3)*H*), 7.32 (1 H, t, *J* 7.9, ArC(3)*H*), 7.43 (2 H, d, *J* 8.0, SO_2Ar(3,5)*H*), 7.58 (1 H, d, *J* 8.0, ArC(2)*H*), 7.71 (1 H, d, *J* 7.8, ArC(4)*H*), 7.77 - 7.83 (2 H, m), 7.95 (2 H, d, *J* 8.0, SO_2Ar(2)*H*); δ_C (125 MHz, $CDCl_3$); 21.9 (SO_2Ar*C*H_3), 110.7 (*pyC*(3)H), 114.4 (*pyC*(5)H), 122.0 (q, *J* 275, *C*F_3), 123.3 (*ArC*Br), 125.4 (*ArC*(6)H), 128.8 (SO_2*ArC*(2,6)H), 130.0 (SO_2*ArC*(3,5)H), 130.2 (*ArC*(4)H), 130.4 (*ArC*(5)H), 133.4 (*ArC*(2)H), 134.0 (SO_2*ArC*(4)), 138.2 (*ArC*(1)), 143.4 (*pyC*(4)), 145.7 (SO_2*ArC*(1)), 156.3 (*pyC*(6)), 157.6 (*pyC*(2)); δ_F (470 MHz, $CDCl_3$); −64.6 (C*F*$_3$); *m/z* (NSI$^+$) 472 ([M+H], 100%), HRMS (NSI$^+$) $C_{19}H_{14}{}^{79}BrF_3NO_3S^+$ ([M+H]$^+$) requires 471.9824 found 471.9819 (−1.1 ppm).

6-(4-Bromophenyl)-4-(trifluoromethyl)pyridin-2-yl 4-methylbenzene-1-sulfonate

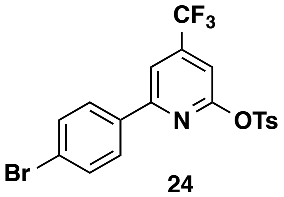

24

Following general procedure **E**, (phenylthio)acetic acid (84.1 mg, 0.50 mmol) in THF (5 mL), *i*-Pr_2NEt (130 μL, 0.75 mmol), pivaloyl chloride (92.5 μL, 0.75 mmol), ketimine **41** (216 mg, 0.5 mmol), DHPB **6** (19.0 mg, 0.1 mmol, 20 mol%) and *i*-Pr_2NEt (87.5 μL, 0.50 mmol) for 4 h at rt followed by heating at reflux for 16 h gave, chromatographic purification (eluent Et_2O:petrol 10:90), pyridine **24** as a white solid (146.8 mg, 62%) which was recrystallised (Et_2O:petrol) for an analytical sample; mp 134-136 °C; ν_{max} (ATR)/cm^{-1} 1146 (S=O), 1306 (S=O), 1564, 1591, 1614, 3110 (C-H); δ_H (400 MHz, $CDCl_3$) 2.51 (3H, s, C*H*$_3$), 7.26 (1H, s, C(3)*H*), 7.40 (2H, d, *J* 8.6, SO_2Ar(3,5)*H*), 7.56-7.60 (2H, m, C(6)Ar*H*), 7.65-7.68 (2H, m, C(6)Ar*H*), 7.81 (1H, s, *C*(5)*H*), 7.95-7.98 (2H, m, SO_2Ar(2,6)*H*); δ_C (100 MHz, $CDCl_3$) 21.8 (*C*H_3), 110.2 (q, *J* 3.5, (*C*(3)), 114.1 (q, *J* 3.2, *C*(5)), 122.0 (q, *J* 272, *C*F_3), 125.3 (C(6)*ArC*(4)), 128.6 (*ArC*), 128.8 (*ArC*), 129.8 (*ArC*), 132.1 (*ArC*), 134.0 (SO_2*ArC*(1)), 135.1 (C(6)*ArC*(1)), 143.3 (q, *J* 34.4, C(4)), 145.7 (SO_2*ArC*(4)), 156.7 (*C*(6)), 157.5 (*C*(2)); δ_F (470 MHz, $CDCl_3$); −64.5 (C*F*$_3$); *m/z* (APCI$^+$) 472 ([M+H]$^+$, 100%); HRMS (APCI$^+$) $C_{19}H_{14}{}^{79}BrF_3NO_3S^+$ ([M+H]$^+$) requires 471.9824; found 471.9825 (+0.1 ppm).

6-(4-Methylphenyl)-4-(trifluoromethyl)pyridine-2-yl-4-methylbenzne-1-sulfonate

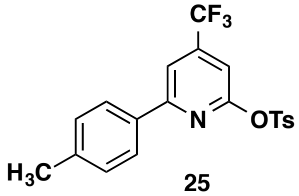

25

Following general procedure **E**, (phenylthio)acetic acid (67 mg, 0.4 mmol) and *i*-Pr_2NEt (100 μL, 0.6 mmol) in THF (6 mL) were added pivaloyl chloride (74 μL, 0.9 mmol) DHPB **6** (15.2 mg, 0.08 mmol, 20 mol%), ketimine **97** (147 mg, 0.4 mmol) and *i*-Pr_2NEt (79 μL, 0.4 mmol) for 4 hours at rt followed by heating at reflux for 16 h gave, after chromatographic purification (eluent Et_2O:petrol 5:95), pyridine **25** as a white solid (97 mg, 60%) which was recrystallised (Et_2O:petrol) for an analytical sample; mp 124-126 °C; ν_{max} (ATR)/cm^{-1} 1346, 1368 (S=O), 3086 (Ar-H); δ_H (500 MHz, $CDCl_3$) 2.44 (3 H, s, ArC*H*$_3$) 2.51 (3 H, s, SO_2ArC*H*$_3$), 7.22 (1 H, s, pyC(3)*H*), 7.25 (2 H, d, *J* 8.0, ArC(3)*H*), 7.40 (2 H, d, *J* 8.6, SO_2ArC(3)*H*), 7.68 (2 H, d, *J* 8.3, SO_2Ar(2)*H*), 7.81 (1 H, s, py(5)*H*), 7.98 (2 H, d, *J* 8.3,

ArC(2)*H*); δ_C (125 MHz, $CDCl_3$); 21.4 (Ar*C*H_3), 21.8 (SO_2Ar*C*H_3), 109.3 (*pyC*(3)H), 113.9 (*pyC*(5)H), 122.0 (q, *J* 274.8, 633.0, *C*F_3), 127.0 (SO_2*ArC*(2,6)H) 128.8 (*ArC*(2,6)H), 129.6 (*ArC*(3,5)H), 129.7 (SO_2*ArC*(3,5)H), 133.5 (*ArC*(4)), 134.1 (SO_2*ArC*(4)), 140.9(*ArC*(1)), 143.0 (SO_2*ArC*(1)), 145.5 (*pyC*(6)), 157.4 (*pyC*(4)), 158.0 (*pyC*(2)); δ_F (470 MHz, $CDCl_3$); −64.6 (C*F_3*); *m/z* (NSI^+) 408 ([M+H], 100%), HRMS (NSI^+) $C_{20}H_{17}F_3NO_3S^+$ ($[M+H]^+$) requires 408.0876 found 408.0874 (−0.4 ppm).

6-(4-Fluorophenyl)-4-(trifluoromethyl)pyridine-2-yl-4-methylbenzene-1-sulfonate

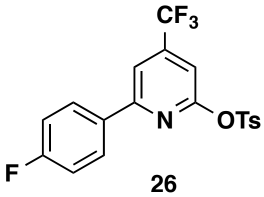

26

Following general procedure **E**, (phenylthio)acetic acid (101 mg, 0.6 mmol) and *i*-Pr_2NEt (210 μL, 1.2 mmol) in THF (9 mL) were added pivaloyl chloride (110 μL, 0.9 mmol) DHPB **6** (22 mg, 0.12 mmol, 20 mol%), ketimine **98** (223 mg, 0.6 mmol) and *i*-Pr_2NEt (110 mL, 0.6 mmol) for 4 hours at rt followed by heating at reflux for 16 h gave, after chromatographic purification (eluent Et_2O:petrol 5:95), pyridine **26** as a yellow solid (114 mg, 45%) which was recrystallised (Et_2O:petrol) for an analytical sample; mp 99-102 °C; ν_{max} (ATR)/cm^{-1} 1170 (Ar-F), 1350 (S=O), 3094 (Ar-H); δ_H (400 MHz, $CDCl_3$) 2.48 (3 H, s, SO_2ArC*H_3*), 7.11 (2 H, t, *J* 8.57, ArC(3,5)*H*), 7.21 (1 H, s, pyC(3)*H*), 7.38 (2 H, d, *J* 7.24, SO_2Ar(3,5)*H*), 7.74-7.79 (3 H, m, py(5)*H* and Ar(2,6)*H*), 7.95 (SO_2ArC(2,6)*H*); δ_C (100 MHz, $CDCl_3$); 21.8 (SO_2Ar*C*H_3), 109.7 (*pyC*(3)H), 113.9 (*pyC*(5)H), 115.9 (d, *J* 21.9, *ArC*(3,5)H), 122.0 (q, *J* 271, *C*F_3), 128.8 (SO_2*ArC*(2,6)H), 129.1 (d, *J* 8.7, *ArC*(2,6)H), 129.8 (SO_2*ArC*(3,5)H), 132.4 (SO_2*ArC*(4)), 134.0 (*ArC*(1)), 143.2 (q, *J* 34.9, *pyC*(4)), 145.6 (SO_2*ArC*(1)), 156.8 (*pyC*(6)), 157.4 (*pyC*(4)), 164.3 (d, *J* 252, *ArC*(4)); δ_F (376 MHz, $CDCl_3$); −65.2 (C*F_3*), −110.5 (Ar*F*); *m/z* (NSI^+) 412 ([M+H], 100%); HRMS (NSI^+) $C_{19}H_{14}F_4NO_3S^+$ ($[M+H]^+$) requires 412.0625 found 412.0623 (−0.5 ppm).

6-(4-(Methylsulfonyl)phenyl)-4-(trifluoromethyl)pyridin-2-yl 4-methylbenzenesulfonate

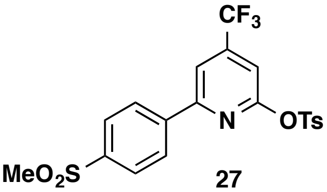

27

Following general procedure **E**, (phenylthio)acetic acid (390 mg, 2.32 mmol) in THF (25 mL), *i*-Pr_2NEt (0.60 mL, 3.48 mmol), pivaloyl chloride (0.44 mL, 3.48 mmol), ketimine **49** (1.00 g, 2.32 mmol), DHPB **6** (88.3 mg, 0.46 mmol, 20 mol%) and *i*-Pr_2NEt (0.41 mL, 2.32

mmol) for 4 h at rt followed by heating at reflux for 16 h gave, after chromatographic purification (eluent Et_2O:petrol 65:35), pyridine **27** as a white solid (580 mg, 53%) which was recrystallised (Et_2O:petrol) for an analytical sample; mp 146-148 °C; ν_{max} (ATR)/cm^{-1} 1144 (S=O), 1306 (S=O), 1562, 1599, 2912, 2934, 3118 (C-H); δ_H (500 MHz, $CDCl_3$) 2.52 (3H, s, ArC*H*$_3$), 3.13 (3H, s, SO_2C*H*$_3$), 7.35 (1H, s, C(3)*H*), 7.42 (2H, d, *J* 8.0, SO_2Ar(3,5)*H*), 7.91 (1H, s, *C*(5)*H*), 7.96-8.06 (6H, m, Ar*H*); δ_C (125 MHz, $CDCl_3$) 21.9 (Ar*C*H_3), 44.5 (SO_2*C*H_3), 111.3 (q, *J* 3.5, (*C*(3)), 115.0 (q, *J* 3.1, *C*(5)), 121.9 (q, *J* 272, *C*F_3), 128.1 (*ArC*), 128.1 (*ArC*), 128.7 (*ArC*), 129.9 (*ArC*), 133.8 (OSO_2*ArC*(1)), 141.2 (4ry *ArC*), 142.0 (4ry *ArC*), 143.7 (q, *J* 34.5, C(4)), 145.9 (OSO_2*ArC*(4)), 155.7 (*C*(6)), 157.7 (*C*(2)); *m*/*z* (NSI^+) 472 ($[M+H]^+$, 100%); HRMS (NSI^+) $C_{20}H_{17}F_3NO_5S_2^+$ ($[M+H]^+$) requires 472.0495; found 472.0485 (−2.1 ppm).

6-Phenyl-4-(trifluoromethyl)pyridin-2-yl benzene-1-sulfonate

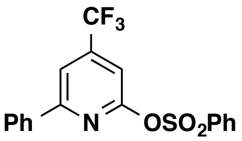

28

Following general procedure **E**, (phenylthio)acetic acid (84.1 mg, 0.50 mmol) in THF (5 mL), *i*-Pr_2NEt (130 μL, 0.75 mmol), pivaloyl chloride (92.5 μL, 0.75 mmol), ketimine **42** (170 mg, 0.5 mmol), DHPB **6** (19.0 mg, 0.1 mmol, 20 mol%) and *i*-Pr_2NEt (87.5 mL, 0.50 mmol) for 4 h at rt followed by heating at reflux for 16 h gave, after chromatographic purification (eluent Et_2O:petrol 10:90), pyridine **28** as a white solid (103 mg, 54%) which was recrystallised (Et_2O:petrol) for an analytical sample; mp 88-90 °C; ν_{max} (ATR)/cm^{-1} 1570, 1371 (S=O), 1177 (S=O), 3118 (C-H); δ_H (300 MHz, $CDCl_3$) 7.26 (1H, s, C(3)*H*), 7.43-7.50 (3H, m, SO_2Ar(3,5)*H* and SO_2Ar(4)*H*), 7.62 (2H, t, *J* 7.8, C(6)Ar(3,5)*H*), 7.72-7.78 (3H, m, C(6)Ar(2,6)*H* and C(6)Ar(4)*H*), 7.85 (1H, s, *C*(5)*H*), 8.11 (2H, d, *J* 8.3, SO_2Ar(2,6)*H*); δ_C (75 MHz, $CDCl_3$) 109.8 (q, *J* 3.5, (*C*(3)), 114.4 (q, *J* 3.2, *C*(5)), 122.2 (q, *J* 272, *C*F_3), 127.1 (C(6)*ArC*(2,6)), 128.8 (*ArC*), 128.9 (*ArC*), 129.2 (*ArC*), 130.6 (C(6)*ArC*(4)), 134.3 (SO_2*ArC*(4)), 136.2 (C(6)*ArC*(1)), 137.2 (SO_2*ArC*(1)), 143.2 (q, *J* 34.1, C(4)), 157.4 (*C*(2) or *C*(6)), 158.0 (*C*(2) or *C*(6)); δ_F (282 MHz, $CDCl_3$); −65.1 (C*F*$_3$); *m*/*z* (NSI^+) 380 ($[M+H]^+$, 100%); HRMS (NSI^+) $C_{18}H_{13}F_3NO_3S^+$ ($[M+H]^+$) requires 380.0563; found 380.0563 (+0.1 ppm).

6-(4-Bromophenyl)-4-(trifluoromethyl)pyridin-2-yl benzene-1-sulfonate

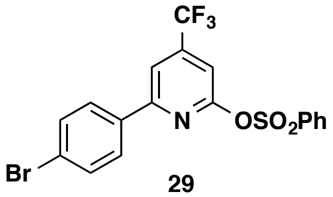

29

Following general procedure **E**, (phenylthio)acetic acid (84.1 mg, 0.50 mmol) in THF (5 mL), *i*-Pr_2NEt (130 mL, 0.75 mmol), pivaloyl chloride (92.5 mL, 0.75 mmol), ketimine **96** (209 mg, 0.5 mmol), DHPB **6** (19.0 mg, 0.1 mmol, 20 mol%) and *i*-Pr_2NEt (87.5 mL, 0.50 mmol) for 4 h at rt followed by heating at reflux for 16 h gave, chromatographic purification (eluent Et_2O:petrol 5:10), pyridine **29** as a white solid (151 mg, 66%) which was recrystallised (Et_2O:petrol) for an analytical sample; mp 150-152 °C; ν_{max} (ATR)/cm^{-1} 1172 (S=O), 1373 (S=O), 1564, 1616, 3115 (C-H); δ_H (500 MHz, $CDCl_3$) 7.28 (1H, s, C(3)*H*), 7.54-7.65 (6H, m, Ar*H*), 7.75 (1H, t, *J* 7.2, SO_2Ar(4)*H*), 7.82 (1H, s, *C*(5)*H*), 8.09 (2H, d, *J* 7.6, SO_2Ar(2,6)*H*); δ_C (75 MHz, $CDCl_3$) 110.2 (q, *J* 3.6, (*C*(3)), 114.2 (q, *J* 3.2, *C*(5)), 122.1 (q, *J* 272, *C*F_3), 125.3 (C(6)*ArC*(4)), 128.6 (*ArC*), 128.7 (*ArC*), 129.2 (*ArC*), 132.1 (*ArC*), 134.3 (SO_2*ArC*(4)), 135.0 (C(6)*ArC*(1)), 137.2 (SO_2*ArC*(1)), 143.4 (q, *J* 34.3, C(4)), 156.8 (*C*(6)), 157.5 (*C*(2)); δ_F (470 MHz, $CDCl_3$); −64.6 (C*F_3*); *m/z* (NSI^+) 457 ($[M+H]^+$, 97%); HRMS (NSI^+) $C_{18}H_{12}{}^{79}BrF_3N_2O_2S^+$ ($[M+H]^+$) requires 457.9668; found 457.9667 (−0.2 ppm).

6-(Naphthalene-2-yl)-4-(trifluoromethyl)pyridine-2-yl 4-methylbenzene-1-sulfonate

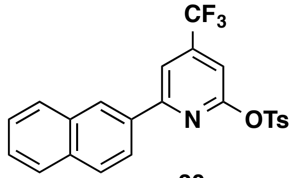

30

Following general procedure **E**, (phenylthio)acetic acid (67 mg, 0.4 mmol) and *i*-Pr_2NEt (100 μL, 0.6 mmol) in THF (6 mL) were added pivaloyl chloride (74 μL, 0.6 mmol) DHPB **6** (15.2 mg, 0.08 mmol, 20 mol%), ketimine **100** (161 mg, 0.4 mmol) and *i*-Pr_2NEt (79 μL, 0.4 mmol) for 4 hours at rt followed by heating at reflux for 16 h gave, after chromatographic purification (eluent Et_2O:petrol 10:90), pyridine **30** as a yellow solid (82 mg, 46%) which was recrystallised (Et_2O:petrol) for an analytical sample mp 112-114 °C; ν_{max} (ATR)/cm^{-1} 1344, 1371 (S=O), 3090 (Ar-H); δ_H (500 MHz, $CDCl_3$) 2.49 (3 H, s, SO_2ArC*H_3*), 7.26 (1 H, s, py(3)*H*), 7.40 (2 H, d, *J* 8.1, SO_2Ar(3,5)*H*), 7.54-7.58 (2 H, m, Np*H*), 7.83-7.90 (4 H, m, N*pH*), 7.96 (1 H, s, py(5)*H*), 7.99 (2 H, d, *J* 8.3, SO_2Ar(2,6)*H*), 8.24 (1 H, s, Np(1)*H*); δ_C (125 MHz, $CDCl_3$) 21.8 (SO_2Ar*C*H_3), 109.9 (*pyC*(3)H), 114.5 (*pyC*(5)H), 122.1 (*C*F_3), 123.9 (*NpC*H), 126.8 (*NpC*H), 127.3(*NpC*H), 127.5 (*NpC*H), 127.8 (*NpC*H), 128.7 (*NpC*H), 128.8 (*NpC*H), 128.9 (SO_2*ArC*(2,5)H), 129.8 (SO_2*Ar*C(3,5)H), 133.2 (SO_2*ArC*(4)), 133.5

(SO_2*ArC*(1)), 134.1 (*NpC*), 134.2 (*NpC*), 143.2 (q, *J* 34.1, *pyC*(4)), 145.5 (*NpC*(2)), 157.5 (*pyC*(6)), 157.9 (*pyC*(2)); δ_F (470 MHz, $CDCl_3$) −64.6 (C*F₃*); *m/z* ($APCI^+$) 444 ([M+H], 100%), HRMS (NSI^+) $C_{23}H_{17}F_3NO_3S^+$ ($[M+H]^+$) requires 444.0876 found 444.0872 (−0.8 ppm).

6-(Furan-2-yl)-4-(trifluoromethyl)pyridin-2-yl 4-methylbenzene-1-sulfonate

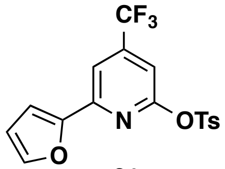

31

Following general procedure **E**, (phenylthio)acetic acid (84.1 mg, 0.50 mmol) in THF (5 mL), *i*-Pr_2NEt (130 μL, 0.75 mmol), pivaloyl chloride (92.5 μL, 0.75 mmol), ketimine **101** (172 mg, 0.5 mmol), DHPB **6** (19.0 mg, 0.1 mmol, 20 mol%) and *i*-Pr_2NEt (87.5 μL, 0.50 mmol) for 4 hours at rt followed by heating at reflux for 16 h gave, after chromatographic purification (eluent Et_2O:petrol 10:90), pyridine **31** as a white solid (89.9 mg, 47%) which was recrystallised (Et_2O:petrol) for an analytical sample; mp 78-80 °C; ν_{max} (ATR)/cm^{-1} 1146 (S=O), 1305 (S=O), 1570, 1624, 3090 (C-H); δ_H (500 MHz, $CDCl_3$) 2.50 (3H, s, CH_3), 6.55 (1H, s, C(6)Ar*H*), 6.88 (1H, d, *J* 2.8, C(6)Ar*H*), 7.14 (1H, s, C(3)*H*), 7.42 (2H, d, *J* 7.9, SO_2Ar(3,5)*H*), 7.56 (1H, s, C(6)Ar(5)*H*), 7.77 (1H, s, *C*(5)*H*), 7.99 (2H, d, *J* 8.0 SO_2Ar(2,6)*H*); δ_C (125 MHz, $CDCl_3$) 21.8 (CH_3), 109.2 (q, *J* 3.6, (*C*(3)), 111.6 (C(6)*ArC*), 112.4 (q, *J* 3.4, *C*(5)), 112.6 (C(6)*ArC*), 122.0 (q, *J* 272, CF_3), 128.9 (SO_2*ArC*), 129.7 (SO_2*ArC*), 133.9 (SO_2*ArC*(1)), 143.1 (q, *J* 34.3, C(4)), 144.6 (C(6)*ArC*(5), 145.6 (SO_2*ArC*(4)), 149.4 (C(6)*ArC*(2)), 151.4 (*C*(6)), 157.4 (*C*(2)); δ_F (282 MHz, $CDCl_3$); −66.8 (C*F₃*); *m/z* ($APCI^+$) 384 ($[M+H]^+$, 100%); HRMS ($APCI^+$) $C_{17}H_{13}F_3NO_4S^+$ ($[M+H]^+$) requires 384.0512; found 384.0509 (−0.8 ppm).

6-(Thiophen-2-yl)-4-(trifluoromethyl)pyridine-2-yl-4-methylbenzne-1-sulfonate

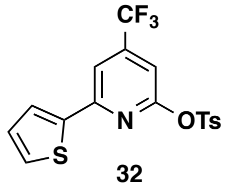

32

Following general procedure **E**, (phenylthio)acetic acid (67 mg, 0.4 mmol) and *i*-Pr_2NEt (100 μL, 0.4 mmol) in THF (6 mL) were added pivaloyl chloride (74 μL, 0.6 mmol) DHPB (15.2 mg, 0.08 mmol, 20 mol%), ketimine **102** (143.8 mg, 0.4 mmol) and *i*-Pr_2NEt (79 μL, 0.4 mmol for 4 hours at rt followed by heating at reflux for 16 h gave, after chromatographic purification (eluent Et_2O:petrol 5:95), pyridine **32** as a white solid (72 mg, 45%) which was

recrystallised (Et_2O:petrol) for an analytical sample; mp 124-125 °C; ν_{max} (ATR)/cm^{-1} 1369 (S=O), 3098 (Ar-H); δ_H (500 MHz, $CDCl_3$) 2.47 (3 H, s, SO_2ArC*H₃*), 7.08 - 7.15 (2 H, m, py(3)*H* and C(1)Ar(4)*H*), 7.39 (2 H, d, *J* 8.0, SO_2Ar(3,5)*H*), 7.46 (1 H, d, *J* 5.1, C(1)Ar(3)*H*), 7.57 (1 H, d, *J* 3.8, C(1)Ar(5)*H*), 7.67 (1 H, s, pyC(5)*H*), 8.00 (2 H, d, *J* 8.2, SO_2Ar(2,6)*H*). δ_C (125 MHz, $CDCl_3$); 21.8 (SO_2Ar*C*H_3), 109.2 (*pyC*(3)H), 112.8 (*pyC*(5)H), 127.0 (C(1)*ArC*(5)H) 127.9 (q, *J* 1345, 593, *C*F_3), 128.4 (C(1)*ArC*(4)H) 128.9 (SO_2*ArC*(2,6)H), 129.7 (C(1)*ArC*(3)H), 129.8 (SO_2*ArC*(3,5)H), 134.2 (SO_2*ArC*(4)), 141.7 (C(1)*ArC*(1)), 143.1 (*pyC*(4)), 145.6 (SO_2*ArC*(1)), 150.5 (*pyC*(6)), 153.1 (*pyC*(4)), 157.3 (*pyC*(2)); δ_F (470 MHz, $CDCl_3$); −64.8 (CF_3); *m/z* (NSI$^+$) 400 ([M+H], 100%), HRMS (NSI$^+$) $C_{17}H_{13}F_3NO_3S_2^+$ ([M+H]$^+$) requires 400.0283 found 400.0283 (−0.1 ppm).

4-Phenyl-6-(trifluoromethyl)pyridin-2-yl 4-methylbenzenesulfonate

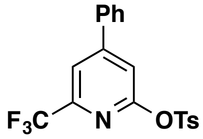

33

Following general procedure **E** (phenylthio)acetic acid (84.1 mg, 0.50 mmol) in THF (5 mL), *i*-Pr_2NEt (130 μL, 0.75 mmol), pivaloyl chloride (92.5 μL, 0.75 mmol), ketimine **103** (177 mg, 0.5 mmol), DHPB **6** (19.0 mg, 0.1 mmol, 20 mol%) and *i*-Pr_2NEt (87.5 μL, 0.50 mmol) for 30 minutes at rt followed by heating at reflux for 48 h gave, after chromatographic purification (eluent Et_2O:petrol 15:85), pyridine **33** as a white solid (79.0 mg, 40%) which was recrystallised (Et_2O:petrol) for an analytical sample; mp 130-132 °C; ν_{max} (ATR)/cm^{-1} 1147 (S=O), 1303 (S=O), 1553, 1597, 1614, 3119 (C-H); δ_H (300 MHz, $CDCl_3$) 2.51 (3H, s, C*H*₃), 7.41 (2H, d, *J* 8.1, SO_2Ar(3,5)*H*), 7.52-7.68 (6H, , m, Ar*H* and C(3)*H*), 7.80 (1H, s, C(5)*H*), 8.03 (2H, d, *J* 8.0, SO_2Ar(2,6)*H*); δ_C (75 MHz, $CDCl_3$) 21.8 (*C*H_3), 115.8 (*C*(3)), 117.1 (q, *J* 2.9, *C*(5)), 120.8 (q, *J* 273, *C*F_3), 127.2 (*ArC*), 129.3 (*ArC*), 129.5 (*ArC*), 129.6 (*ArC*), 130.5 (C(4)*ArC*(4)), 133.4 (4ry *ArC*), 135.9 (4ry *ArC*), 145.8 (SO_2*ArC*(4)), 146.8 (q, *J* 35.7, C(6)), 154.9 (*C*(4)), 157.9 (*C*(2)); *m/z* (NSI$^+$) 394 ([M+H]$^+$, 100%); HRMS (NSI$^+$) $C_{19}H_{15}F_3NO_3S^+$ ([M+H]$^+$) requires 394.0719; found 394.0716 (−0.8 ppm).

1.6 Product Derivatisations

2-Phenyl-4-(trifluoromethyl)pyridine[16]

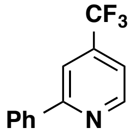

43

To a screw cap glass test tube was charged $Pd(OAc)_2$ (3.37 mg, 15.0 mmol), 1,3-Bis(diphenylphosphino)propane (6.19 mg, 15.0 mmol), Et_3N (0.21 mL, 1.50 mmol), pyridine **22** (118 mg, 0.30 mmol), DMF (2 mL) and formic acid (34.0 mL, 0.90 mmol) and the reaction mixture was heated at 60 °C for 1 h. Once cooled, the reaction mixture was quenched with brine and extracted with EtOAc (x 3). The combined organic extracts were dried ($MgSO_4$), filtered and concentrated *in vacuo*. Chromatographic purification (eluent Et_2O:petrol 10:90) gave pyridine **43** as a colourless oil (59.8 mg, 89%); δ_H (300 MHz, $CDCl_3$) 7.48-7.59 (4H, m, C(2)Ar(3,5)*H*, C(2)Ar(4)*H* and C(5)*H*), 7.97 (1H, s, C(3)*H*), 8.05-8.09 (2H, m, C(2)Ar(2,6)*H*), 8.91 (1H, d, *J* 5.1, C(6)*H*); δ_F (470 MHz, $CDCl_3$); −64.8 (C*F₃*); All data in accordance with literature.[16]

4-(6-Phenyl-4-(trifluoromethyl)pyridin-2-yl)morpholine

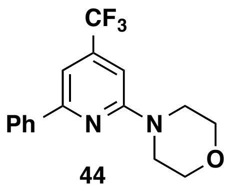

44

To a solution of pyridine **22** (78.6 mg, 0.20 mmol) in toluene (2 mL) was added Et_3N (55.8 μL, 0.40 mmol) and morpholine (172 μL, 2.00 mmol) and the reaction mixture was heated at 120 °C for 16 h. Once cooled, the reaction mixture was concentrated *in vacuo*. Chromatographic purification (eluent Et_2O:petrol 15:85) gave pyridine **44** as a white solid (52.6 mg, 85%); mp 98-100 °C; ν_{max} (ATR)/cm^{-1} 1568, 1611, 2860, 2968, 2988 (C-H); δ_H (500 MHz, $CDCl_3$) 3.71 (4H, t, *J* 4.9, 2 NCH_2), 3.90 (4H, t, *J* 4.9, 2 OCH_2), 6.78 (1H, s, C(3)*H*), 7.32 (1H, s, C(5)*H*), 7.44-7.52 (3H, m, C(6)Ar(3,5)*H* and C(6)Ar(4)*H*), 8.04-8.06 (2H, m, C(6)Ar(2,6)*H*); δ_C (100 MHz, $CDCl_3$) 45.3 (2 N*C*H_2), 66.7 (2 O*C*H_2), 100.8 (q, *J* 3.9, *C*(3)), 105.3 (q, *J* 3.2, *C*(5)), 123.4 (q, *J* 272, *C*F_3), 126.9 (C(6)*ArC*(2,6)), 128.7 (C(6)*ArC*(3,5)), 129.5 (C(6)*ArC*(4)), 138.6 (C(6)*ArC*(1)), 140.7 (q, *J* 32.7, C(4)), 156.8 (*C*(6)), 159.2 (*C*(2)); δ_F (282 MHz, $CDCl_3$); −65.4 (C*F₃*); *m*/*z* (NSI^+) 309 ($[M+H]^+$, 100%); HRMS (NSI^+) $C_{16}H_{16}F_3N_2O^+$ ($[M+H]^+$) requires 309.1209; found 309.1210 (+0.2 ppm).

***N*-(1-(6-Phenyl-4-(trifluoromethyl)pyridine-2-yl)vinyl)acetamide**

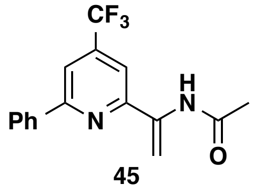

45

To a screw cap glass test tube was charged Pd(dba)$_2$ (8.60 mg, 15.0 mmol), 1,1'-bis(diphenylphosphino)ferrocene (8.30 mg, 15.0 mmol), dicyclohexylamine (0.19 mL, 0.90 mmol), *N*-vinylacetamide (102 mg, 1.2 mmol), pyridine **22** (118 mg, 0.30 mmol) and 1,4-dioxane (3 mL) and the reaction mixture was heated 100 °C for 16 h. Once cooled, the reaction mixture filtered through Celite using CH_2Cl_2 as eluent and concentrated *in vacuo*. Chromatographic purification (eluent Et_2O:petrol 30:70) gave pyridine **45** as a white solid (69.7 mg, 76%); mp 130-132 °C; ν_{max} (ATR)/cm^{-1} 1506, 1566, 1692 (C=O), 3082 (C-H), 3310 (N-H),; δ_H (300 MHz, $CDCl_3$) 2.30 (3H, s, C*H*$_3$), 5.72 (1H, s, =CH*H*), 6.70 (1H, s, =C*H*H), 7.56-7.63 (3H, m, C(6)Ar(3,5)*H* and C(6)Ar(4)*H*), 7.91 (1H, s, C(3)*H* or C(5)*H*), 7.92 (1H, s, C(3)*H* or C(5)*H*), 8.02-8.05 (2H, m, C(6)Ar(2,6)*H*), 9.30 (1H, s, N*H*); δ_C (75 MHz, $CDCl_3$) 25.2 (*C*H$_3$), 101.1 (=*C*HH), 113.2 (q, *J* 3.5, *C*(3) or *C*(5)), 115.5 (q, *J* 3.3, *C*(3) or *C*(5)), 122.8 (q, *J* 272, *C*F$_3$), 127.0 (C(6)*ArC*(2,6)), 129.2 (C(6)*ArC*(3,5)), 130.3 (C(6)*ArC*(4)), 136.8 (C(2)*C*=CHH), 137.5 (C(6)*ArC*(1)), 140.4 (q, *J* 33.7, C(4)), 153.3 (*C*(2)), 157.2 (*C*(6)), 169.1 (*C*=O); δ_F (282 MHz, $CDCl_3$); −65.2 (C*F$_3$*); *m*/*z* (NSI$^+$) 307 ([M+H]$^+$, 57%); HRMS (NSI$^+$) $C_{16}H_{14}F_3N_2O^+$ ([M+H]$^+$) requires 307.1053; found 307.1052 (−0.2 ppm).

***tert*-Butyl 5-methoxy-2-(6-phenyl-4-(trifluoromethyl)pyridine-2-yl)-1*H*-indole-1-carboxylate**

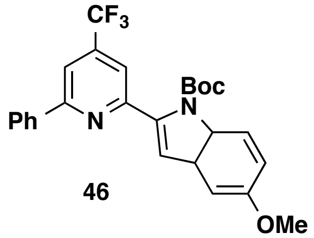

46

To a screw cap glass test tube was charged Pd(OAc)$_2$ (2.25 mg, 0.01 mmol), 2-(dicyclohexylphospino)-3,6-dimethoxy-2',4',6'-triisopropyl-1,1'-biphenyl (10.7 mg, 0.02 mmol), pyridine **22** (197 mg, 0.50 mmol), (1-(*tert*-butoxycarbonyl)5-methoxy-1*H*-indol-2-yl)boronic acid (291 mg, 1.00 mmol), $K_3PO_4.H_2O$ (345 mg, 1.50 mmol) and toluene (1 mL) and the reaction mixture was heated at 110 °C for 2 h. Once cooled, the reaction mixture was quenched with H_2O and extracted with EtOAc (× 3). The combined organic extracts were dried ($MgSO_4$), filtered and concentrated *in vacuo*. Chromatographic purification (eluent Et_2O:petrol 5:95) gave pyridine **46** as a colourless oil (190 mg, 81%); ν_{max} (ATR)/cm^{-1} 1572,

1732, (C=O), 2978 (C-H),; δ_H (300 MHz, $CDCl_3$) 1.37 (9H, s, C(CH_3)$_3$), 3.92 (3H, s, OCH_3), 6.91 (1H, s, C(2)Ar(3)*H*), 7.06-7.12 (2H, m, C(2)Ar(4)*H* and C(2)Ar(6)*H*), 7.47-7.60 (3H, m, C(6)Ar(3,5)*H* and C(6)Ar(4)*H*), 7.70 (1H, s, C(3)*H*), 7.96 (1H, s, C(5)*H*), 8.16-8.19 (3H, m, C(6)Ar(2,6)*H* and C(2)Ar(7)*H*); δ_C (75 MHz, $CDCl_3$) 27.6 (C(*C*H_3)$_3$), 55.7 (O*C*H_3), 83.7 (*C*(CH_3)$_3$), 103.4 (C(2)*ArC*(4)), 112.2 (C(2)*ArC*(3)), 114.0 (q, *J* 3.2, *C*(5)), 114.6 (C(2)*ArC*(6)), 116.1 (C(2)*ArC*(7)), 117.1 (q, *J* 3.3, *C*(3)), 123.1 (q, *J* 271, *C*F_3), 127.2 (C(6)*ArC*(2,6)), 129.0 (C(6)*ArC*(3,5)), 129.5 (C(2)*ArC*(7a)), 130.0 (C(6)*ArC*(4)), 132.8 (C(2)*ArC*(3a)), 137.7 (C(6)*ArC*(1)), 138.9 (C(2)*ArC*(2)), 139.3 (q, *J* 33.5, C(4)), 149.9 (*C*=O), 154.3 (*C*(2)), 156.2 (C(2)*ArC*(5)), 157.8 (*C*(6)); δ_F (470 MHz, $CDCl_3$); −64.6 (C*F₃*); *m*/*z* (NSI^+) 469 ($[M+H]^+$, 70%); HRMS (NSI^+) $C_{26}H_{24}F_3N_2O_3^+$ ($[M+H]^+$) requires 469.1734; found 469.1727 (−1.4 ppm).

2-(4-Methoxyphenyl)-6-phenyl-4-(trifluoromethyl)pyridine

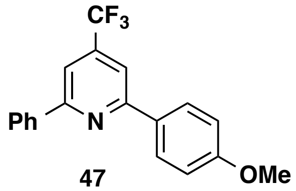

47

To a screw cap glass test tube was charged $Pd(dba)_2$ (7.20 mg, 12.5 mmol), 4,4,5,5-tetramethyl-1,3,2-dioxaphospholane 2-oxide (4.10 mg, 25.0 mmol) and 1,4-dioxane (2 mL) and the reaction mixture was stirred at rt for 5 minutes. 4-Methoxylphenylmagnesium bromide solution (0.5 M in THF, 1.5 mL, 0.75 mmol) was added and the reaction mixture was stirred at rt for 5 minutes. Pyridine **22** (197 mg, 0.50 mmol) was added and the reaction was heated at 80 °C for 16 h. Once cooled, the reaction mixture was quenched with HCl (1 M in H_2O) and extracted with Et_2O (× 3). The combined organic extracts were dried ($MgSO_4$), filtered and concentrated *in vacuo*. Chromatographic purification (eluent Et_2O:petrol 3:97) gave pyridine **47** as a colourless oil (109 mg, 66%); ν_{max} (ATR)/cm^{-1} 1516, 1566, 1609, 2961, 3042; δ_H (300 MHz, $CDCl_3$) 3.78 (3H, s, OCH_3), 6.91-6.96 (2H, m, C(2)Ar(3,5)*H*), 7.34-7.46 (3H, m, C(6)Ar(3,5)*H* and C(6)Ar(4)*H*), 7.70 (2H, app. s, C(3)*H* and C(5)*H*), 8.01-8.08 (4H, m, C(2)Ar(2,6)*H* and C(6)Ar(2,6)*H*); δ_C (75 MHz, $CDCl_3$) 55.4 (O*C*H_3), 113.3 (app. t, *J* 3.6, *C*(3) and *C*(5)), 114.3 (C(2)*ArC*(3,5)), 123.3 (q, *J* 272, *C*F_3), 127.1 (C(6)*ArC*(2,6)), 128.5 (C(2)*ArC*(2,6)), 128.9 (C(6)*ArC*(3,5)), 129.8 (C(6)*ArC*(4)), 132.8 (C(2)*ArC*(1)), 138.4 (C(6)*ArC*(1)), 139.9 (q, *J* 33.1, C(4)), 157.8 (*C*(2) or *C*(6)), 158.0 (C(2) or *C*(6)), 161.2 (*C*(2)*ArC*(4)); δ_F (470 MHz, $CDCl_3$); −64.7 (C*F₃*); *m*/*z* (NSI^+) 330 ($[M+H]^+$, 100%); HRMS (NSI^+) $C_{19}H_{15}F_3NO^+$ ($[M+H]^+$) requires 330.1100; found 330.1102 (+0.5 ppm).

2-Hexyl-6-phenyl-4-(trifluoromethyl)pyridine

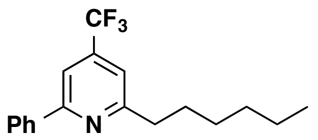

48

To a flask containing Mg turnings (486 mg, 20.0 mmol) in Et_2O (10 ml) under an argon atmosphere was added 2 drops of bromohexane and the reaction mixture was heated to intiate Grigard formation. Bromohexane (2.81 mL, 20.0 mmol) was added dropwise to maintain autoreflux. Once all bromide was added the reaction mixture was stirred at rt for 30 minutes until all Mg was consumed.

To a screw cap glass test tube was charged pyridine **22** (197 mg, 0.50 mmol), $FeCl_3$ (4.06 mg, 0.025 mmol) and THF (2 mL). *N*-Methyl-2-pyrrolidone (0.43 mL, 4.50 mmol) was added and the reaction mixture was cooled to –10 °C before the freshly prepared hexylmagnesium bromide (2.0 M in Et_2O, 0.38 mL, 0.75 mmol) was added dropwise. The reaction mixture was stirred at – 10 °C for 15 minutes before being quenched with MeOH and diluted with H_2O. The reaction mixture was extracted with Et_2O (× 3). The combined organic extracts were dried ($MgSO_4$), filtered and concentrated *in vacuo*. Chromatographic purification (eluent Et_2O:petrol 3:97) gave pyridine **48** as a colourless oil (114 mg, 74%); ν_{max} (ATR)/cm^{-1} 1574, 2928, 2957; δ_H (400 MHz, $CDCl_3$) 0.82 (3H, t, *J* 7.0, CH_3), 1.22-1.35 (6H, m, 3 CH_2), 1.70-1.78 (2H, m, C(2)$CH_2CH_2C_4H_9$), 2.84 (2H, t, *J* 7.8, C(2)$CH_2C_5H_{11}$), 7.20 (1H, s, C(3)), 7.34-7.43 (3H, m, C(6)Ar(3,5)*H* and C(6)Ar(4)*H*), 7.64 (1H, s, C(5)*H*), 7.94-7.97 (2H, m, C(6)Ar(2,6)*H*); δ_C (100 MHz, $CDCl_3$) 14.1 (CH_3) 22.6 (CH_3CH_2), 29.1 (CH_2), 29.6 (CH_2), 31.7 (CH_2), 38.6 (C(2)$CH_2C_5H_{11}$), 113.3 (q, *J* 3.5, *C*(5)), 116.4 (q, *J* 3.4, *C*(3)), 123.2 (q, *J* 272, CF_3), 127.1 (C(6)*ArC*(2,6)), 128.9 (C(6)*ArC*(3,5)), 129.6 (C(6)*ArC*(4)), 138.5 (C(6)*ArC*(1)), 139.1 (q, *J* 33.2, C(4)), 158.1 (*C*(6)), 164.0 (C(2)); δ_F (282 MHz, $CDCl_3$); −65.1 (CF_3); *m/z* (NSI^+) 308 ($[M+H]^+$, 100%); HRMS (NSI^+) $C_{18}H_{21}F_3N^+$ ($[M+H]^+$) requires 308.1621; found 308.1621 (+0.1 ppm).

***N*-Cyclohexyl-6-(4-(methylsulfonyl)phenyl)-4-(trifluoromethyl)pyridin-2-amine[17]**

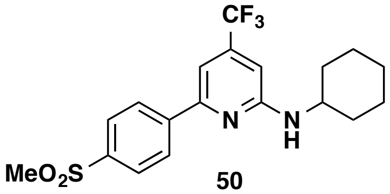

50

To a solution of pyridine **22** (335 mg, 0.71 mmol) in toluene (5 mL) was added Et_3N (0.20 mL, 1.42 mmol) and cyclohexylamine (0.40 mL, 3.56 mmol) and the reaction mixture was heated at 120 °C for 16 h. A further portion of cyclohexylamine (0.40 mL, 3.56 mmol) was added and the reaction mixture was heated at 120 °C for a further 24 h. Once cooled, the

reaction mixture was concentrated *in vacuo*. Chromatographic purification (eluent eluent Et_2O 100%) gave pyridine **50** as a white solid (260 mg, 92%); mp 130-132 °C;[6] δ_H (400 MHz, $CDCl_3$) 1.27-1.55 (5H, m, cyclohexyl C*H*), 1.69-1.74 (1H, m, cyclohexyl C*H*), 1.80-1.86 (2 H, m, cyclohexyl C*H*), 2.10-2.14 (2H, m, cyclohexyl C*H*), 3.11 (3 H, s, SO_2CH_3), 3.72-3.76 (1 H, m, NHC*H*), 4.86 (1 H, d, *J* 7.1, N*H*), 6.58 (1 H, s, C(3)*H*), 7.21 (1 H, s, *C*(5)*H*), 8.03-8.06 (2 H, m, Ar*H*), 8.17-8.20 (2 H, m, Ar*H*); δ_F (282 MHz, $CDCl_3$); −65.6 (C*F₃*); All data in accordance with literature.[17]

N-Cyclohexyl-6-phenyl-4-(trifluoromethyl)pyridin-2-amine

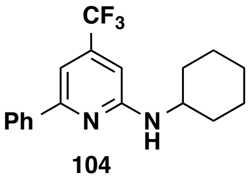

104

To a solution of pyridine **22** (78.6 mg, 0.20 mmol) in toluene (2 mL) was added Et_3N (55.8 μL, 0.40 mmol) and cyclohexylamine (115 μL, 1.00 mmol) and the reaction mixture was heated at 120 °C for 16 h. A further portion of cyclohexylamine (115 μL, 1.00 mmol) was added and the reaction mixture was heated at 120 °C for a further 24 h. Once cooled, the reaction mixture was concentrated *in vacuo*. Chromatographic purification (eluent Et_2O:petrol 10:90) gave pyridine **104** as a colourless oil (50.7 mg, 79%); ν_{max} (ATR)/cm^{-1} 1510, 1572, 1616, 2855; δ_H (500 MHz, $CDCl_3$) 1.27-1.33 (3H, m, cyclohexyl C*H*), 1.43-1.52 (2H, m, cyclohexyl C*H*), 1.69-1.85 (3H, m, cyclohexyl C*H*), 2.11-2.14 (2H, m, cyclohexyl C*H*), 3.71-3.72 (1H, m, NHC*H*), 4.82 (1H, d, *J* 7.6, N*H*), 6.50 (1H, s, C(3)*H*), 7.19 (1H, s, C(5)*H*), 7.43-7.51 (3H, m, C(6)Ar(3,5)*H* and C(6)Ar(4)*H*), 7.99-8.01 (2H, m, C(6)Ar(2,6)*H*); δ_C (100 MHz, $CDCl_3$) 24.9 (cyclohexyl *C*H_2), 25.8 (cyclohexyl *C*H_2), 33.2 (cyclohexyl *C*H_2), 50.4 (NH*C*H), 100.8 (*C*(3)), 104.4 (q, *J* 3.3, *C*(5)), 123.4 (q, *J* 271, *C*F_3), 126.9 (C(6)*ArC*(2,6)), 128.7 (C(6)*ArC*(3,5)), 129.2 (C(6)*ArC*(4)), 138.9 (C(6)*ArC*(1)), 140.4 (q, *J* 32.5, C(4)), 157.5 (*C*(2) or *C*(6)), 158.0 (*C*(2) or *C*(6)); δ_F (282 MHz, $CDCl_3$); −65.6 (C*F₃*); *m/z* (NSI$^+$) 321 ([M+H]$^+$, 97%); HRMS (NSI$^+$) $C_{18}H_{20}F_3N_2^+$ ([M+H]$^+$) requires 321.1573; found 321.1573 (+0.0 ppm).

2-(Furan-3-yl)-6-phenyl-4-(trifluoromethyl)pyridine

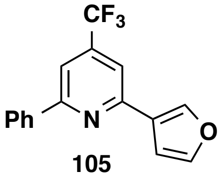

105

To a screw cap glass test tube was charged $Pd(OAc)_2$ (2.25 mg, 0.01 mmol), 2-(dicyclohexylphospino)-3,6-dimethoxy-2’,4’,6’-triisopropyl-1,1’-biphenyl (10.7 mg, 0.02

mmol), pyridine **22** (197 mg, 0.50 mmol), 3-furanylboronic acid (112 mg, 1.00 mmol), $K_3PO_4.H_2O$ (345 mg, 1.50 mmol) and toluene (1 mL) and the reaction mixture was heated at 110 °C for 2 h. Once cooled, the reaction mixture was quenched with H_2O and extracted with EtOAc (x 3). The combined organic extracts were dried ($MgSO_4$), filtered and concentrated *in vacuo*. Chromatographic purification (eluent Et_2O:petrol 5:95) gave pyridine **105** as a colourless oil (101 mg, 70%); ν_{max} (ATR)/cm^{-1} 1508, 1572, 1616, 2959 2988, 3012; δ_H (500 MHz, $CDCl_3$) 7.05 (1H, s, C(2)Ar(4)*H*), 7.50-7.59 (5H, m, Ar*H*), 7.82 (1H, s, C(3)*H*), 8.15 (2H, d, *J* 7.3, C(6)Ar(2,6)*H*), 8.22 (1H, s, C(5)*H*); δ_C (125 MHz, $CDCl_3$) 108.7 (C(2)*ArC*(4)), 113.6 (q, *J* 3.4, *C*(3), 113.6 (q, *J* 3.4, *C*(5)), 123.1 (q, *J* 272, *C*F_3), 126.6 (C(2)*ArC*(3)), 127.1 (C(6)*ArC*(2,6)), 128.9 (C(6)*ArC*(3,5)), 129.9 (C(6)*ArC*(4)), 138.1 (C(6)*ArC*(1)), 139.7 (q, *J* 33.3, C(4)), 142.3 (C(2)*ArC*(2)), 144.2 (C(2)*ArC*(5)), 152.9 (*C*(2)), 158.3 (*C*(6)); δ_F (470 MHz, $CDCl_3$); −64.8 (C*F₃*); *m*/*z* (NSI$^+$) 290 ([M+H]$^+$, 100%); HRMS (NSI$^+$) $C_{16}H_{11}F_3NO^+$ ([M+H]$^+$) requires 290.0787; found 290.0789 (+0.6 ppm).

6-Phenyl-4-(trifluoromethyl)pyridin-2(1*H*)-one

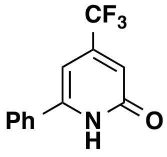

106

To a solution of pyridine **22** (118 mg, 0.30 mmol) in EtOH (10 mL) was added *n*-Bu_4NOH (1.5 M in H_2O, 2 mL, 3.00 mmol) and the reaction mixture was stirred at rt for 15 minutes. The reaction mixture was extracted with CH_2Cl_2 (x 3) and the combined organic extracts were dried ($MgSO_4$), filtered and concentrated *in vacuo*. Chromatographic purification (eluent Et_2O 100%) gave pyridone **106** as a white solid (63.9 mg, 89%); mp 174-176 °C; ν_{max} (ATR)/cm^{-1} 1506, 1612, 1667 (C=O), 3063 (C-H); δ_H (300 MHz, $CDCl_3$) 6.66 (1H, d, *J* 1.5, C(5)*H*), 6.84 (1H, s, C(3)*H*), 7.55-7.60 (3H, m, C(6)Ar(3,5)*H* and C(6)Ar(4)*H*), 7.78-7.82 (2H, m, C(6)Ar(2,6)*H*); δ_C (125 MHz, $CDCl_3$) 100.4 (q, *J* 2.4, *C*(5)), 115.8 (q, *J* 3.0, *C*(3)), 122.3 (q, *J* 273, *C*F_3), 127.0 (C(6)*ArC*(2,6)), 129.4 (C(6)*ArC*(3,5)), 131.0 (C(6)*ArC*(4)), 132.5 (C(6)*ArC*(1)), 143.2 (q, *J* 33.3, C(4)), 149.1 (*C*(6)), 164.8 (*C*(2)=O); δ_F (470 MHz, $CDCl_3$); −64.6 (C*F₃*); *m*/*z* (NSI$^+$) 240 ([M+H]$^+$, 100%); HRMS (NSI$^+$) $C_{12}H_9F_3NO^+$ ([M+H]$^+$) requires 240.0631; found 240.0631 (+0.1 ppm).

1.7 *N*- to *O*-Sulfonyl Migration Studies

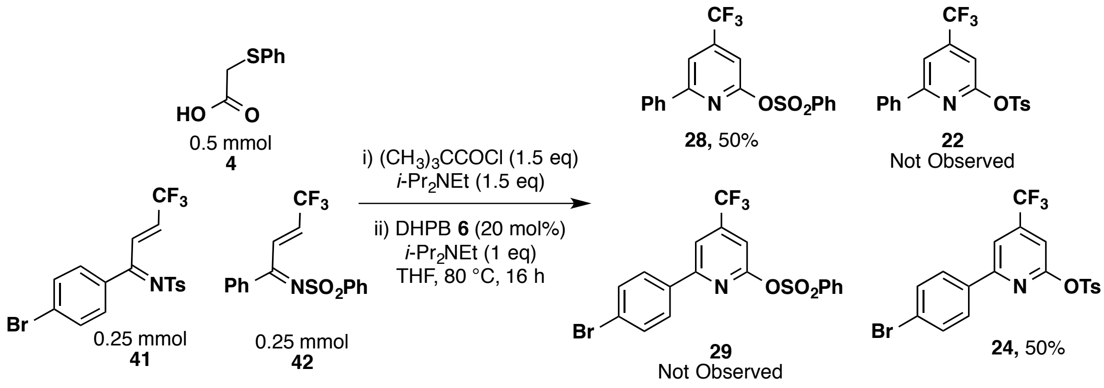

Following general procedure **E**, (phenylthio)acetic acid (84.1 mg, 0.50 mmol) in THF (5 mL), *i*-Pr_2NEt (130 mL, 0.75 mmol), pivaloyl chloride (92.5 mL, 0.75 mmol), ketimine **42** (85.0 mg, 0.25 mmol), ketimine **41** (108 mg, 0.25 mmol), DHPB **6** (19.0 mg, 0.1 mmol, 20 mol%) and *i*-Pr_2NEt (87.5 mL, 0.50 mmol) for 30 minutes at rt followed by heating at reflux for 16 h gave, by analysis of the crude ^{1}H NMR, a crude reaction mixture containing 50% of both pyridines **28** and **24** (arising from either intra- or inter-molecular sulfonyl transfer) with no observable presence of pyridines **22** or **29** (arising from intermolecular transfer only). This result suggests that the sulfonyl transfer process is intramolecular in nature due to the absence of pyrdines **22** and **29**.

1.8 Other Michael-Acceptors Investigated

Dimethyl 2-(phenyl(tosylimino)methyl)fumarate[18]

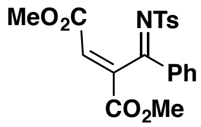

107

To a solution of *N*-benzylidene-4-methylbenzenesulfonamide (1.00 g, 3.86 mmol) in THF (25 mL) was added dimethyl acetylenedicarboxylate (0.56 mL, 4.63 mmol) and the reaction mixture was stirred at rt for 5 minutes. DMAP (94.0 mg, 0.77 mmol) was added and the reaction mixture was stirred at 60 °C for 16 h before being concentrated *in vacuo*. Chromatographic purification (eluent Et_2O:petrol 40:60) gave ketimine **107** as a white solid (0.53 g, 34%); mp 124-126 °C; {lit.[18] mp 140-142 °C}; δ_H(300 MHz, $CDCl_3$) 2.46 (3 H, s, ArCH_3), 3.61 (3 H, s, CO_2CH_3), 3.87 (3 H, s, CO_2CH_3), 7.23 (1 H, s, C*H*), 7.35 (2 H, d, *J* 8.0, SO_2Ar(3,5)*H*), 7.40-7.46 (2 H, m, Ph(3,5)*H*), 7.54-7.60 (1 H, m, Ph(4)*H*), 7.85-7.91 (4 H, m, Ph(2,6)*H* and SO_2Ar(2,6)*H*). All data in accordance with literature.[18]

Methyl 2-(phenyl(tosylimino)methyl)acrylate[19]

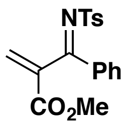

108

To a solution of *N*-benzylidene-4-methylbenzenesulfonamide (1.00 g, 3.86 mmol) and PPh_3 (202 mg, 0.77 mmol) in toluene (60 mL) at 80 °C was added a solution of methyl propiolate (0.41 mL, 4.63 mmol) in toluene (20 mL) dropwise over 3 h. After complete addition the reaction mixture was cooled and concentrated *in vacuo*. Chromatographic purification (eluent Et_2O:petrol 25:75) gave ketimine **108** as a colourless oil (0.35 g, 27%); δ_H (500 MHz, $CDCl_3$) 2.47 (3 H, s, ArC*H*$_3$), 3.81 (3 H, s, CO_2C*H*$_3$), 5.96 (1 H, s, =C*H*H), 6.86 (1 H, s, =CH*H*), 7.37 (2 H, d, *J* 8.1, SO_2Ar(3,5)*H*), 7.44 (2 H, t, *J* 7.9, Ph(3,5)*H*), 7.59 (1 H, t, *J* 7.4, Ph(4)*H*), 7.88-7.93 (4 H, m, Ph(2,6)*H* and SO_2Ar(2,6)*H*). All data in accordance with literature.[19]

(3*E*)-Methyl 4-phenyl-2-(tosylimino)but-3-enoate

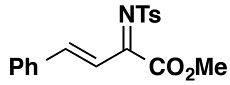

109

Following general procedure **E**, methyl (3*E*)-2-oxo-4-phenylbut-3-enoate[3] (1.60 g, 8.42 mmol) and 4-toluenesulfonamide (1.44 g, 8.42 mmol) in CH_2Cl_2 (20 mL), Et_3N (2.35 mL, 16.8 mmol) and $TiCl_4$ (0.93 mL, 8.42 mmol) gave, after chromatographic purification (eluent Et_2O:petrol 50:50), ketimine **109** as a yellow oil (1.59 g, 55%); ν_{max} (ATR)/cm^{-1} 1145 (S=O), 1310, 1560, 1612, (S=O), 1736 (C=O), 3061 (C-H); δ_H (500 MHz, $CDCl_3$) 2.46 (3 H, s, ArC*H*$_3$), 4.10 (3 H, s, CO_2C*H*$_3$), 6.86 (1 H, d, *J* 16.4, C(3)*H*), 7.36-7.54 (8 H, m, Ar*H* and C(4)*H*), 7.92 (2 H, d, *J* 8.3, SO_2Ar(2,6)*H*); δ_C (125 MHz, $CDCl_3$) 21.7 (Ar*C*H_3), 53.6 (CO_2*C*H_3), 123.4 (*C*(3)), 128.1 (*ArC*), 128.8 (*ArC*), 129.2 (*ArC*), 129.2 (*ArC*), 129.8 (*ArC*), 131.9 (C(4)*ArC*(4)), 133.9 (C(4)*ArC*(1)), 135.7 (SO_2*ArC*(4)), 149.7 (*C*(4)), 165.0 (*C*(2)), 167.5 (*C*(1)); *m*/*z* (NSI$^+$) 344 ([M+H]$^+$, 18%); HRMS (NSI$^+$) $C_{18}H_{18}NO_4S^+$ ([M+H]$^+$) requires 344.0951; found 344.0955 (+1.1 ppm).

Methyl 6-oxo-4-phenyl-1-tosyl-1,6-dihydropyridine-2-carboxylate

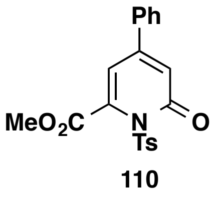

110

Following general procedure **E**, (phenylthio)acetic acid (84.1 mg, 0.50 mmol) in THF (5 mL), *i*-Pr_2NEt (130 μL, 0.75 mmol), pivaloyl chloride (92.5 μL, 0.75 mmol), ketimine **109** (172

mg, 0.5 mmol), DHPB **6** (19.0 mg, 0.1 mmol, 20 mol%) and *i*-Pr_2NEt (87.5 μL, 0.50 mmol) for 30 minutes at rt followed by heating at reflux for 16 h gave, after chromatographic purification (eluent Et_2O:petrol 40:60), pyridone **110** as an off-white solid (86.0 mg, 45%); mp 150-152 °C; ν_{max} (ATR)/cm^{-1} 1595, 1612, 1668 (C=O Lactam), 1728, 2951, (C=O Ester), 3071 (C-H); δ_H (500 MHz, $CDCl_3$) 2.48 (3H, s, ArC*H*$_3$), 4.03 (3H, s, CO_2C*H*$_3$), 6.65 (1H, d, *J* 1.8, C(5)*H*), 6.81 (1H, d, *J* 1.8, C(3)*H*), 7.39 (2H, d, *J* 8.2, SO_2Ar(3,5)*H*), 7.48-7.50 (3H, m, Ar*H*), 7.55-7.57 (2H, m, Ar*H*), 8.16 (2H, d, *J* 8.4, SO_2Ar(2,6)*H*); δ_C (75 MHz, $CDCl_3$) 21.9 (Ar*C*H_3), 53.6 (CO_2*C*H_3), 110.7 (*C*(3)), 121.1 (*C*(5)), 126.7 (*ArC*), 129.3 (*ArC*), 129.4 (*ArC*), 130.2 (*ArC*), 130.7 (C(4)*ArC*(4)), 134.3 (4ry *ArC*), 135.2 (4ry *ArC*), 137.3 (4ry *ArC*), 146.2 (SO_2*ArC*(4)), 151.0 (*C*(4)), 161.1 (*C*(6)), 163.4 (*C*O_2CH_3); *m/z* (NSI$^+$) 384 ([M+H]$^+$, 100%); HRMS (NSI$^+$) $C_{20}H_{18}NO_5S^+$ ([M+H]$^+$) requires 384.0900; found 384.0900 (−0.1 ppm).

Methyl 6-oxo-2-phenyl-5-(phenylthio)-1-tosyl-1,4,5,6-tetrahydropyridine-3-carboxylate

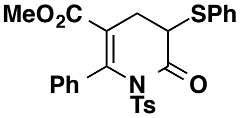

(±)-**111**

Following general procedure **E**, (phenylthio)acetic acid (84.1 mg, 0.50 mmol) in THF (5 mL), *i*-Pr_2NEt (130 μL, 0.75 mmol), pivaloyl chloride (92.5 μL, 0.75 mmol), ketimine **108** (172 mg, 0.5 mmol), DHPB **6** (19.0 mg, 0.1 mmol, 20 mol%) and *i*-Pr_2NEt (87.5 μL, 0.50 mmol) for 30 minutes at rt followed by heating at reflux for 16 h gave, after chromatographic purification (eluent Et_2O:petrol 40:60), lactam **111** as a white solid (130 mg, 53%); mp 128-130 °C; ν_{max} (ATR)/cm^{-1} 1717 (C=O Ester), 1597, 1636 (C=O Lactam), 2930 (C-H); δ_H (500 MHz, $CDCl_3$) 2.42 (3H, s, ArC*H*$_3$), 2.99 (1H, dd, *J* 15.8, 5.6, C(4)*H*H), 3.14 (1H, dd, *J* 15.8, 4.2, C(4)H*H*), 3.55 (3H, s, CO_2C*H*$_3$), 4.00 (1H, dd, *J* 5.5, 4.3, C(5)*H*), 7.17 (2H, d, *J* 8.2, SO_2Ar(3,5)*H*), 7.25-7.34 (7H, m, Ar*H*), 7.40 (1H, m, Ar*H*), 7.47 (2H, d, *J* 8.4, SO_2Ar(2,6)*H*), 7.51-7.53 (2H, m, C(2)Ar(2,6)*H*); δ_C (125 MHz, $CDCl_3$) 21.8 (Ar*C*H_3), 29.0 (*C*(4)), 50.9 (CO_2*C*H_3 or *C*(5)), 52.1 (CO_2*C*H_3 or *C*(5)), 119.2 (*C*(3)), 127.5 (*ArC*), 128.5 (*ArC*), 128.8 (*ArC*), 129.0 (*ArC*), 129.2 (*ArC*), 129.3 (*ArC*), 129.4 (*ArC*), 132.2 (4ry *ArC*), 133.1 (C(2)*ArC*(2,6)), 133.8 (4ry *ArC*), 136.0 (4ry *ArC*), 145.2 (4ry *ArC*), 145.2 (4ry *ArC*), 166.4 (*C*O_2CH_3), 169.6 (*C*(6)); *m/z* (NSI$^+$) 494 ([M+H]$^+$, 100%); HRMS (NSI$^+$) $C_{26}H_{24}NO_5S_2^+$ ([M+H]$^+$) requires 494.1090; found 494.1080 (−2.1 ppm).

The following reactions did not produce the desired pyridine products, although other major products were isolated in the first two cases. Under standard reaction conditions, ketimine **108** affords dihydropyridone **111** in 53% yield, which does not undergo thiophenol

elimination or subsequent sulfonyl transfer. Reaction with ketimine **109** affords pyridone **110** in 45% yield, which does not readily undergo sulfonyl transfer. Ketimine **107** gave less than 5% conversion.

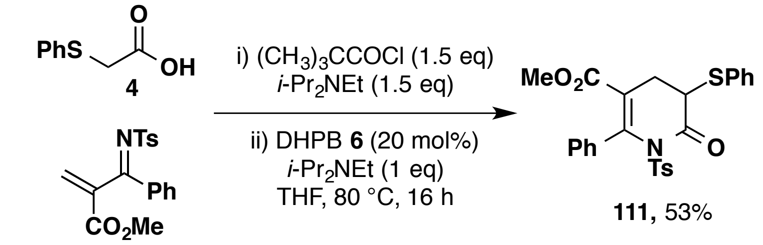

108

111, 53%

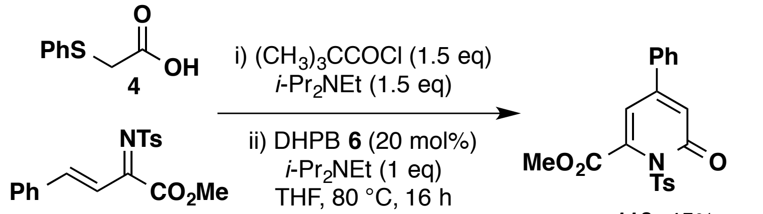

109

110, 45%

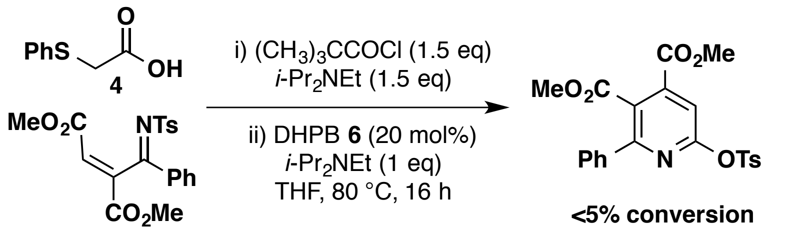

107

<5% conversion

1.9 References and Notes

[1] H. H. Lu, X. F. Wang, C. J. Yao, J. M. Zhang, H. Wu, W. J. Xiao, *Chem. Commun.* **2009**, 4251-4253.

[2] T. Yamazaki, T. Kawasaki-Takasuka, A. Furuta, S. Sakamoto, *Tetrahedron* **2009**, *65*, 5945-5948.

[3] D. Belmessieri, L. C. Morrill, C. Simal, A. M. Slawin, A. D. Smith, *J. Am. Chem. Soc.* **2011**, *133*, 2714-2720.

[4] E. Marqués-López, R. P. Herrera, T. Marks, W. C. Jacobs, D. Könning, R. M. de Figueiredo, M. Christmann, *Org. Lett.* **2009**, *11*, 4116-4119.

[5] E. Venkateswararao, M.-S. Kim, V. K. Sharma, K.-C. Lee, S. Subramanian, E. Roh, Y. Kim, S.-H. Jung, *Eur. J. Med. Chem.* **2013**, *59*, 31-38.

[6] No melting point reported.

[7] J. P. Sonye, K. Koide, *J. Org. Chem*. **2006**, *71*, 6254-6257.

[8] X. Li, N. Liu, H. Zhang, S. E. Knudson, H.-J. Li, C.-T. Lai, C. Simmerling, R. A. Slayden, P. J. Tonge, *ACS. Med. Chem. Lett.* **2011**, *2*, 818-823.

[9] K. Bowden, M. P. Henry, *J. Chem. Soc. B.* **1971**, *0*, 156-160.

[10] M. D. Ronsheim, C. K. Zercher, *J. Org. Chem.* **2003**, *68*, 4535-4538.

[11] G. Blond, T. Billard, B. R. Langlois, *J. Org. Chem.* **2001**, *66*, 4826-4830.

[12] This isomeric ratio (E:Z 80:20) was typical for all trifluoromethyl enones **72-81**.

[13] G. Blay, I. Fernandez, M. C. Munoz, J. R. Pedro, C. Vila, *Chem. Eur. J.* **2010**, *16*, 9117-9122.

[14] C. Christophe, T. Billard, B. R. Langlois, *Eur. J. Org. Chem.* **2005**, *2005*, 3745-3748.

[15] T.-Y. Jian, P.-L. Shao, S. Ye, *Chem. Commun.* **2011**, *47*, 2381-2383.

[16] J. Wang, S. Wang, G. Wang, J. Zhang, X.-Q. Yu, *Chem. Commun.* **2012**, *48*, 11769-11771.

[17] a) P. M. Beswick, S. Modi, N. Pegg, J Skidmore, M. Swarkrick, *WO 2004/024691* **2004** b) J. J. Hagan, E. Ratti, C. Routledge, *WO2005/048999,* **2005.**

[18] C.-Q. Li, M. Shi, *Org. Lett.* **2003**, *5*, 4273-4276.

[19] H. Liu, Q. Zhang, L. Wang, X. Tong, *Chem. Commun.* **2010**, *46*, 312-314.

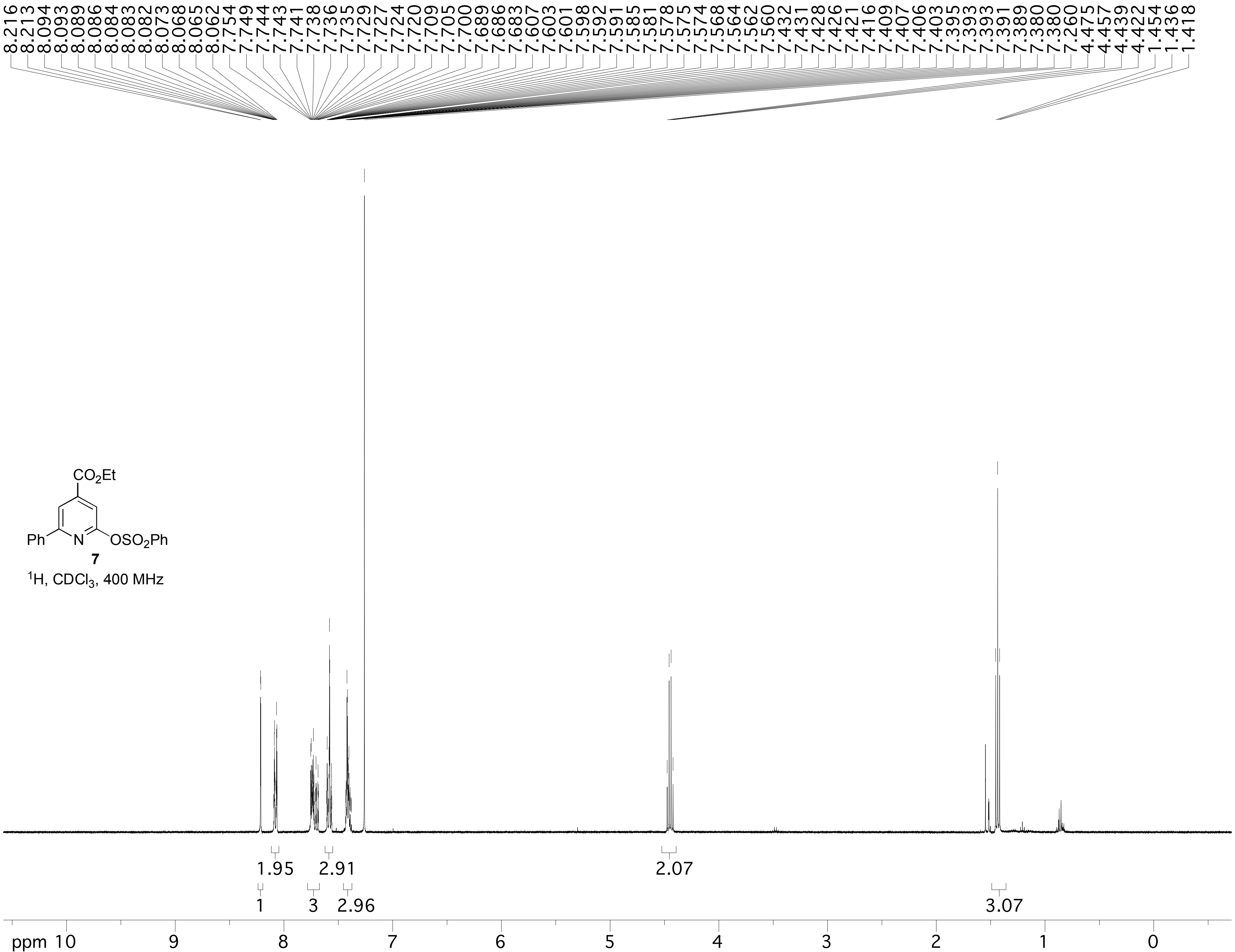

8.216
8.213
8.094
8.093
8.089
8.086
8.084
8.083
8.082
8.073
8.068
8.065
8.062
7.754
7.749
7.744
7.743
7.741
7.738
7.736
7.735
7.729
7.727
7.724
7.720
7.709
7.705
7.700
7.689
7.686
7.683
7.607
7.603
7.601
7.598
7.592
7.591
7.585
7.581
7.578
7.575
7.574
7.568
7.564
7.562
7.560
7.432
7.431
7.428
7.426
7.421
7.416
7.409
7.407
7.406
7.403
7.395
7.393
7.393
7.391
7.389
7.380
7.380
7.260
4.475
4.457
4.439
4.422
1.454
1.436
1.418
CO2Et
Ph
N
OSO2Ph
7
1H, CDCl3, 400 MHz
1.95
2.91
2.07
1
3
2.96
3.07
ppm 10
9
8
7
6
5
4
3
2
1
0

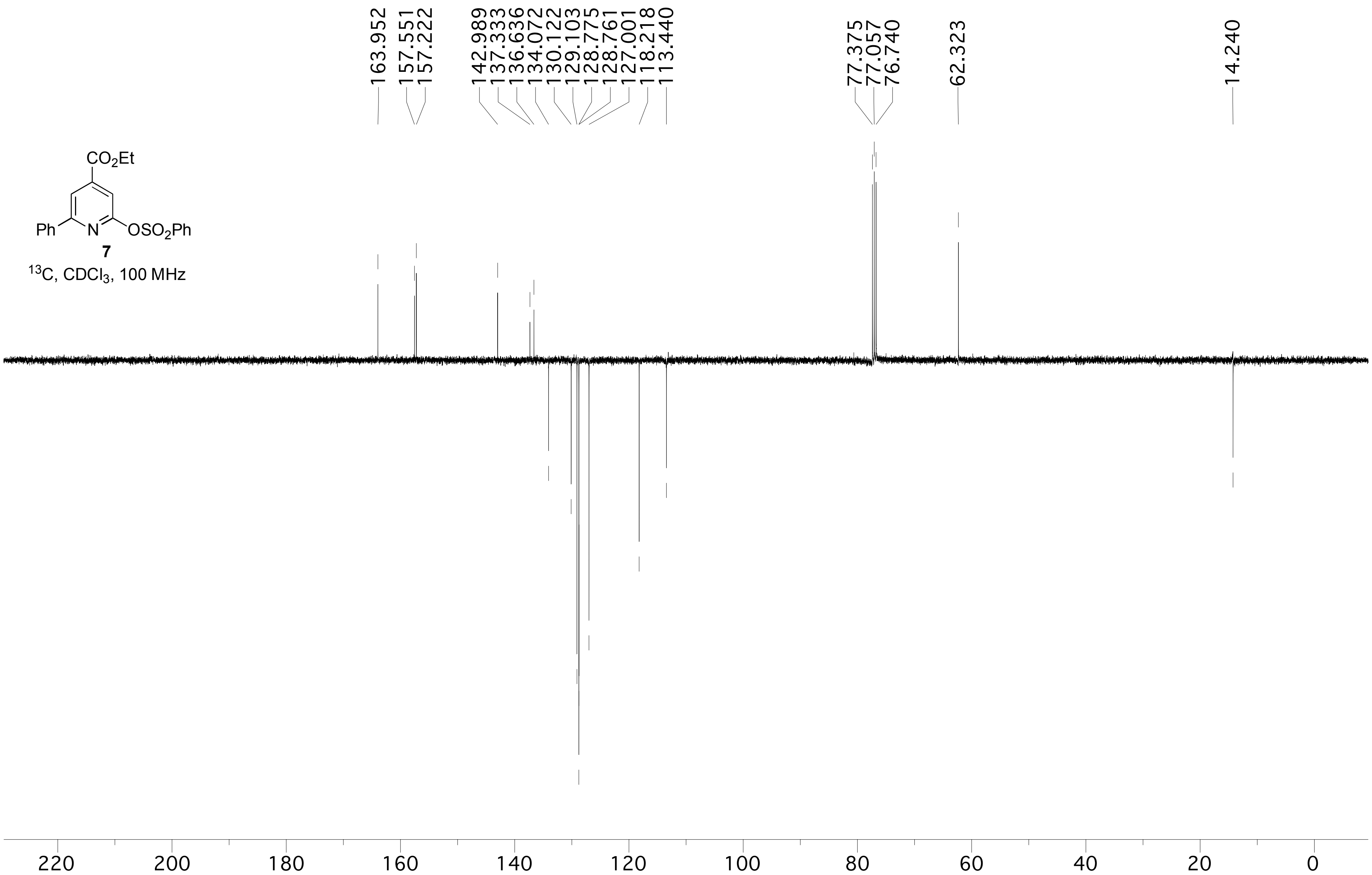
163.952
157.551
157.222
142.989
137.333
136.636
134.072
130.122
129.103
128.775
128.761
127.001
118.218
113.440
77.375
77.057
76.740
62.323
14.240
CO_2Et
Ph
N
OSO_2Ph
7
^{13}C, $CDCl_3$, 100 MHz
220 200 180 160 140 120 100 80 60 40 20 0

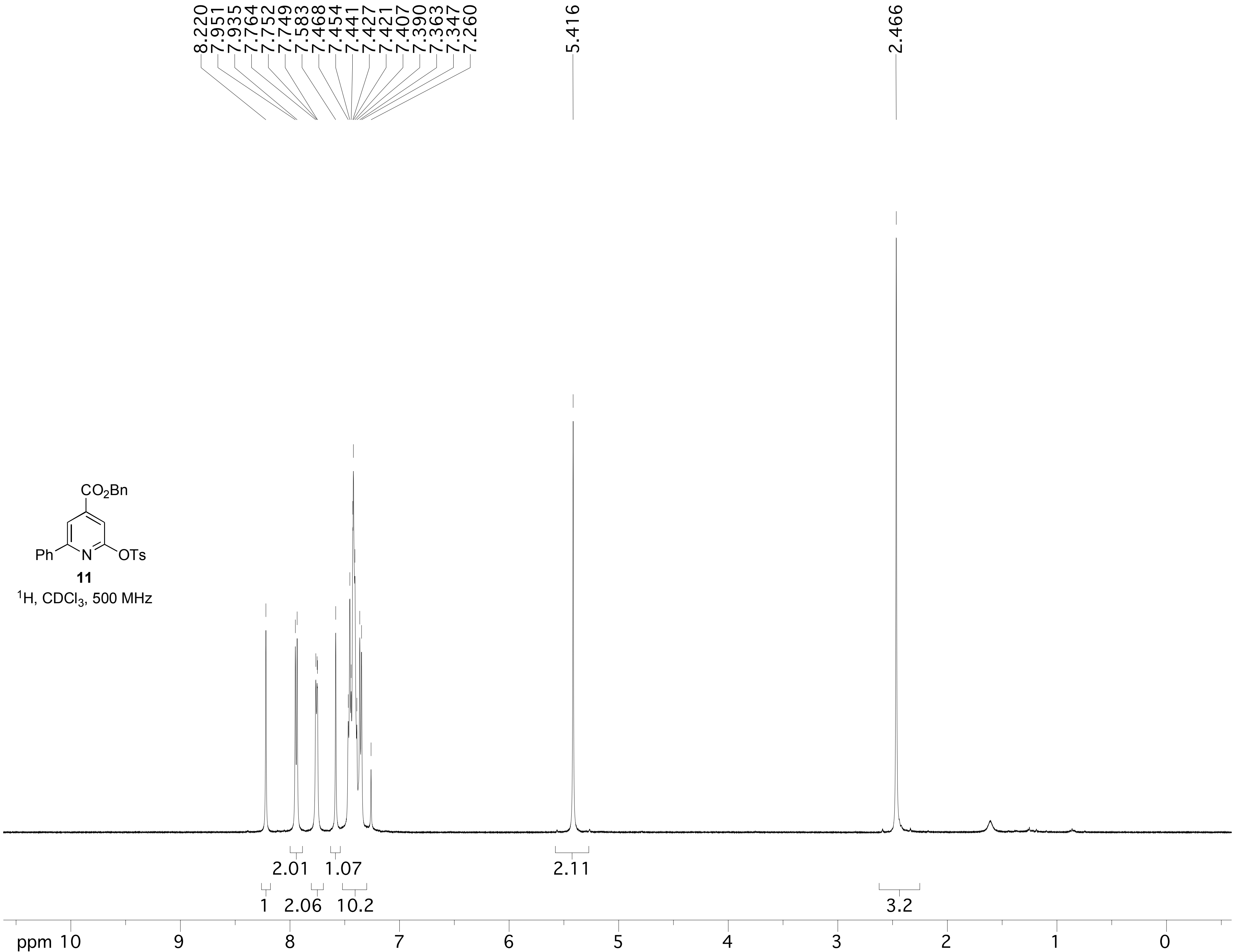
8.220
7.951
7.935
7.764
7.752
7.749
7.583
7.468
7.454
7.441
7.427
7.421
7.407
7.390
7.363
7.347
7.260
5.416
2.466
CO2Bn
Ph
N
OTs
11
1H, CDCl3, 500 MHz
2.01
1.07
2.11
1
2.06
10.2
3.2
ppm 10
9
8
7
6
5
4
3
2
1
0

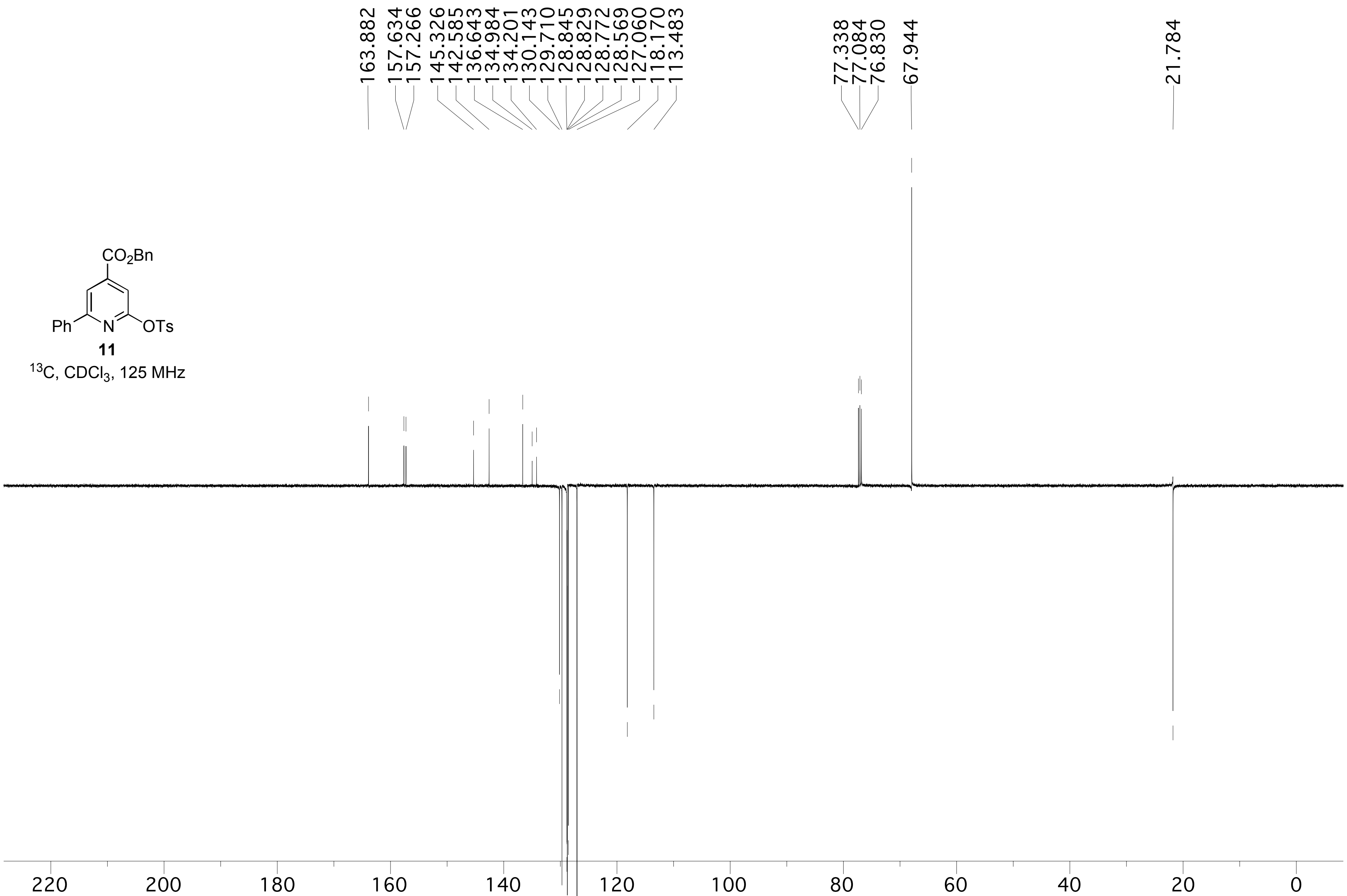
163.882
157.634
157.266
145.326
142.585
136.643
134.984
134.201
130.143
129.710
128.845
128.829
128.772
128.569
127.060
118.170
113.483
77.338
77.084
76.830
67.944
21.784
CO2Bn
Ph
N
OTs
11
13C, CDCl3, 125 MHz
220
200
180
160
140
120
100
80
60
40
20
0

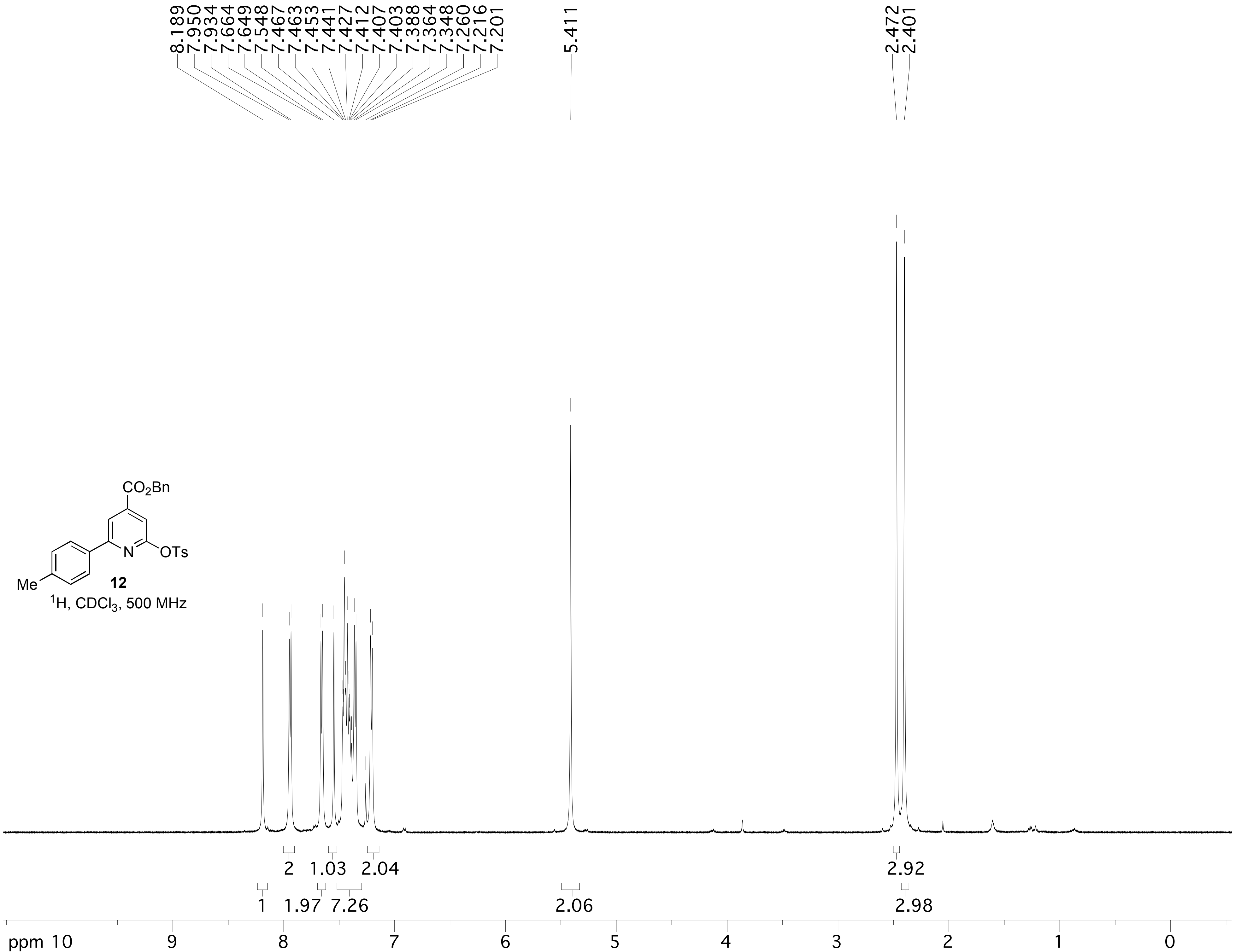
8.189
7.950
7.934
7.664
7.649
7.548
7.467
7.463
7.453
7.441
7.427
7.412
7.407
7.403
7.388
7.364
7.348
7.260
7.216
7.201
5.411
2.472
2.401
CO2Bn
N
OTs
Me
12
1H, CDCl3, 500 MHz
2
1.03
2.04
2.92
1
1.97
7.26
2.06
2.98
ppm 10
9
8
7
6
5
4
3
2
1
0

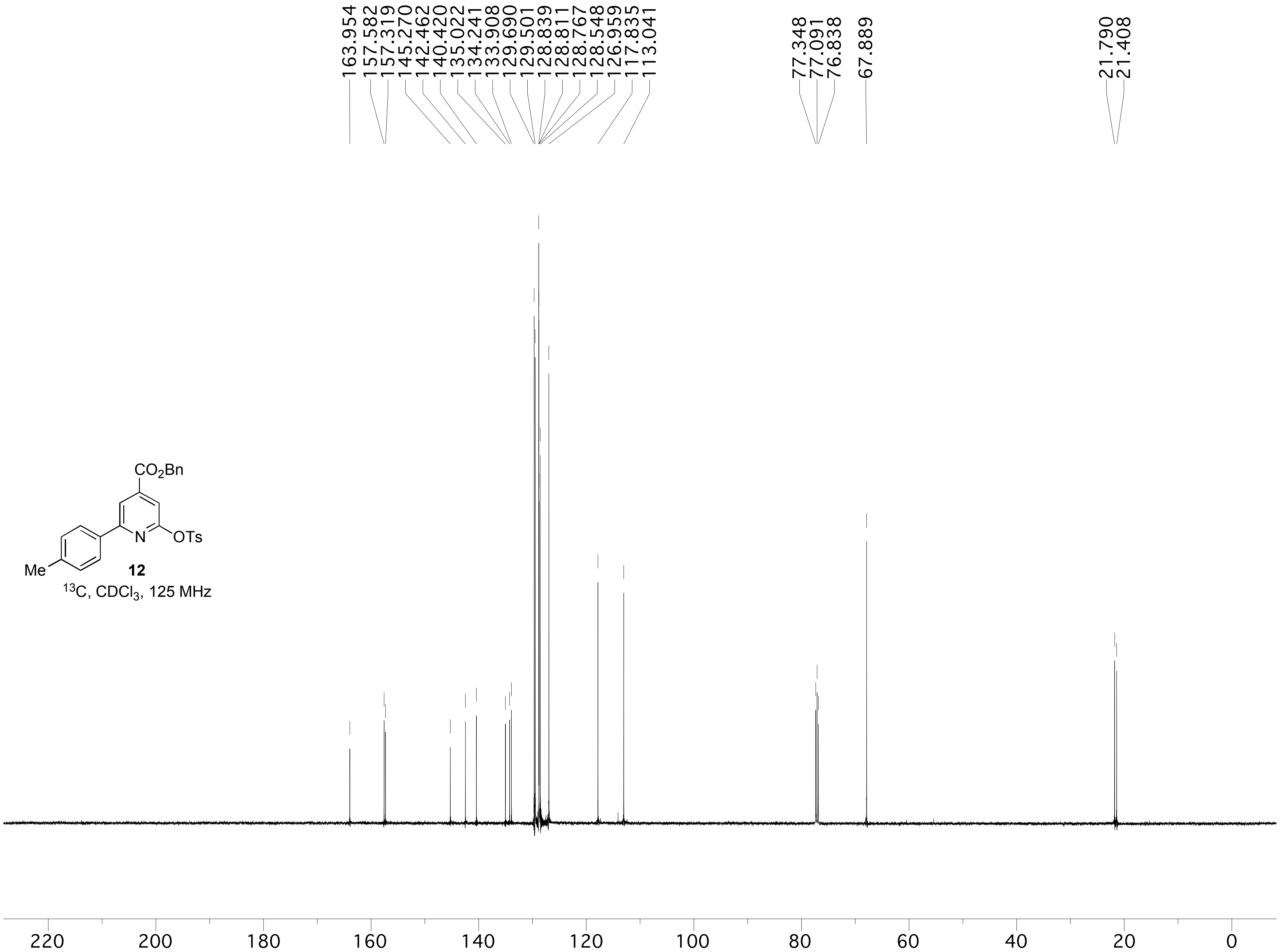

163.954
157.582
157.319
145.270
142.462
140.420
135.022
134.241
133.908
129.690
129.501
128.839
128.811
128.767
128.548
126.959
117.835
113.041
77.348
77.091
76.838
67.889
21.790
21.408
CO_2Bn
N
OTs
Me
12
^{13}C, $CDCl_3$, 125 MHz
220
200
180
160
140
120
100
80
60
40
20
0

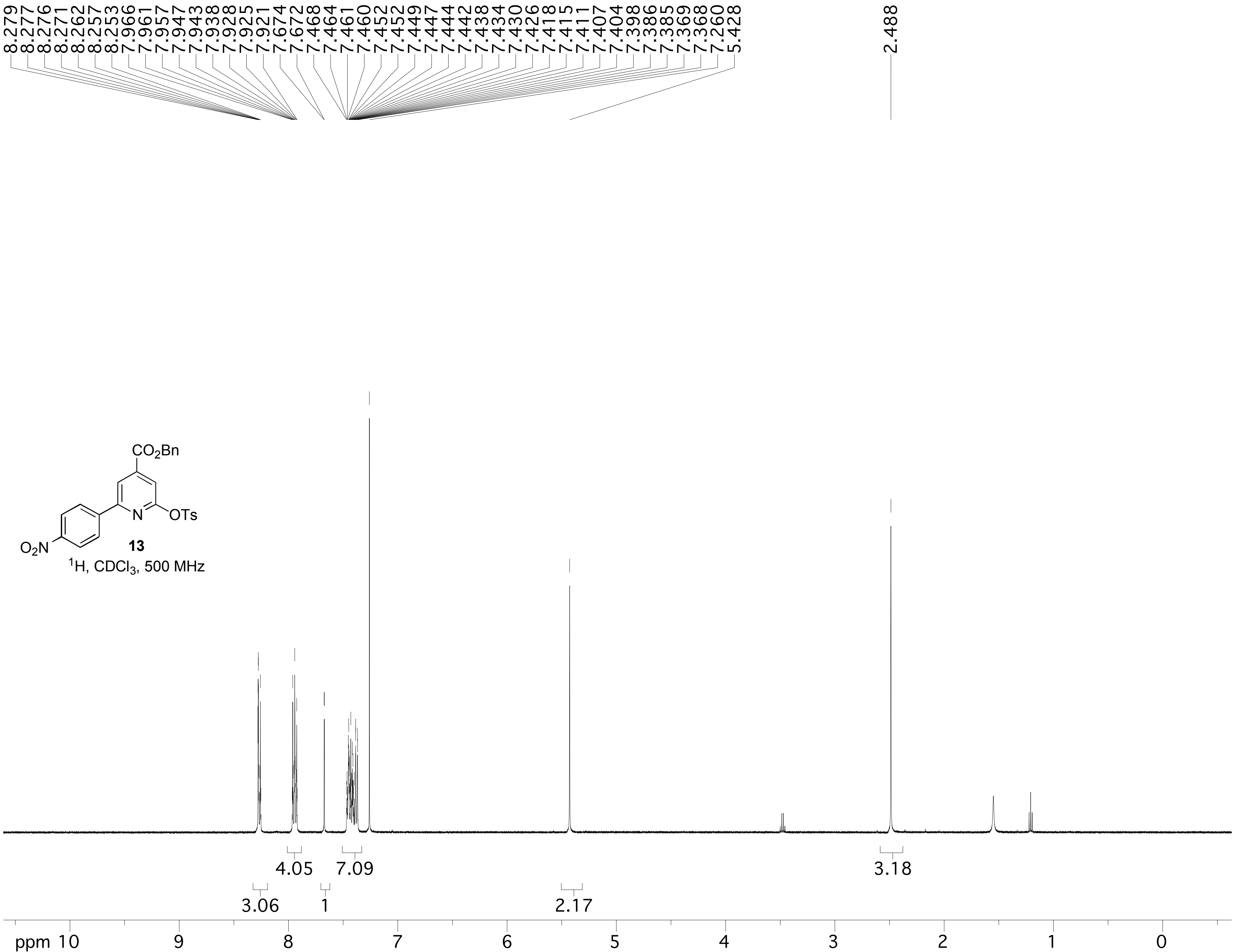
8.279
8.277
8.276
8.271
8.262
8.257
8.253
7.966
7.961
7.957
7.947
7.943
7.938
7.928
7.925
7.921
7.674
7.672
7.468
7.464
7.461
7.460
7.452
7.449
7.447
7.444
7.442
7.438
7.434
7.430
7.426
7.418
7.415
7.411
7.407
7.404
7.398
7.386
7.385
7.369
7.368
7.260
5.428
2.488
CO2Bn
N
OTs
O2N
13
1H, CDCl3, 500 MHz
4.05
7.09
3.18
3.06
1
2.17
ppm 10
9
8
7
6
5
4
3
2
1
0

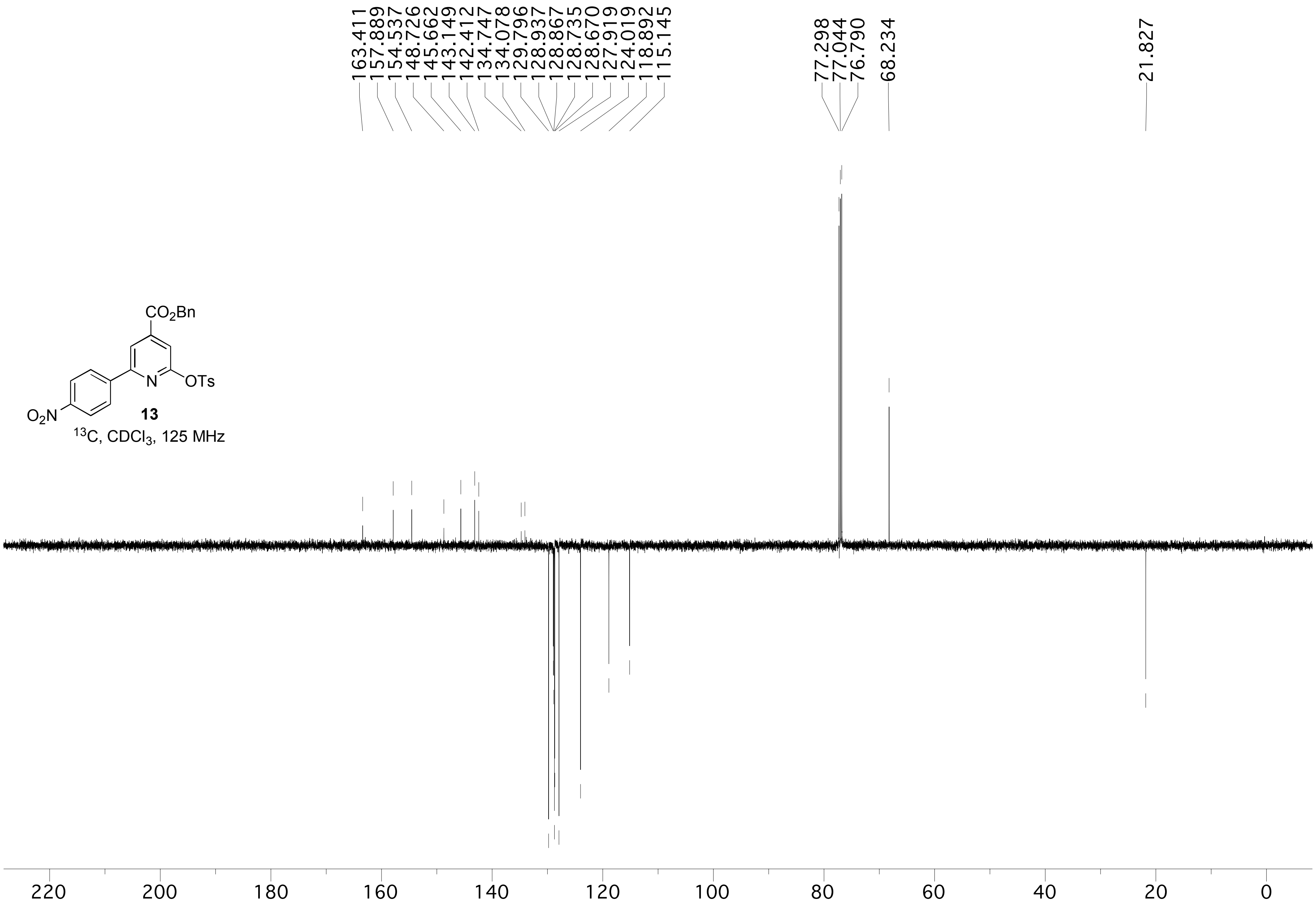
163.411
157.889
154.537
148.726
145.662
143.149
142.412
134.747
134.078
129.796
128.937
128.867
128.735
128.670
127.919
124.019
118.892
115.145
77.298
77.044
76.790
68.234
21.827
CO2Bn
N
OTs
O2N
13
13C, CDCl3, 125 MHz
220
200
180
160
140
120
100
80
60
40
20
0

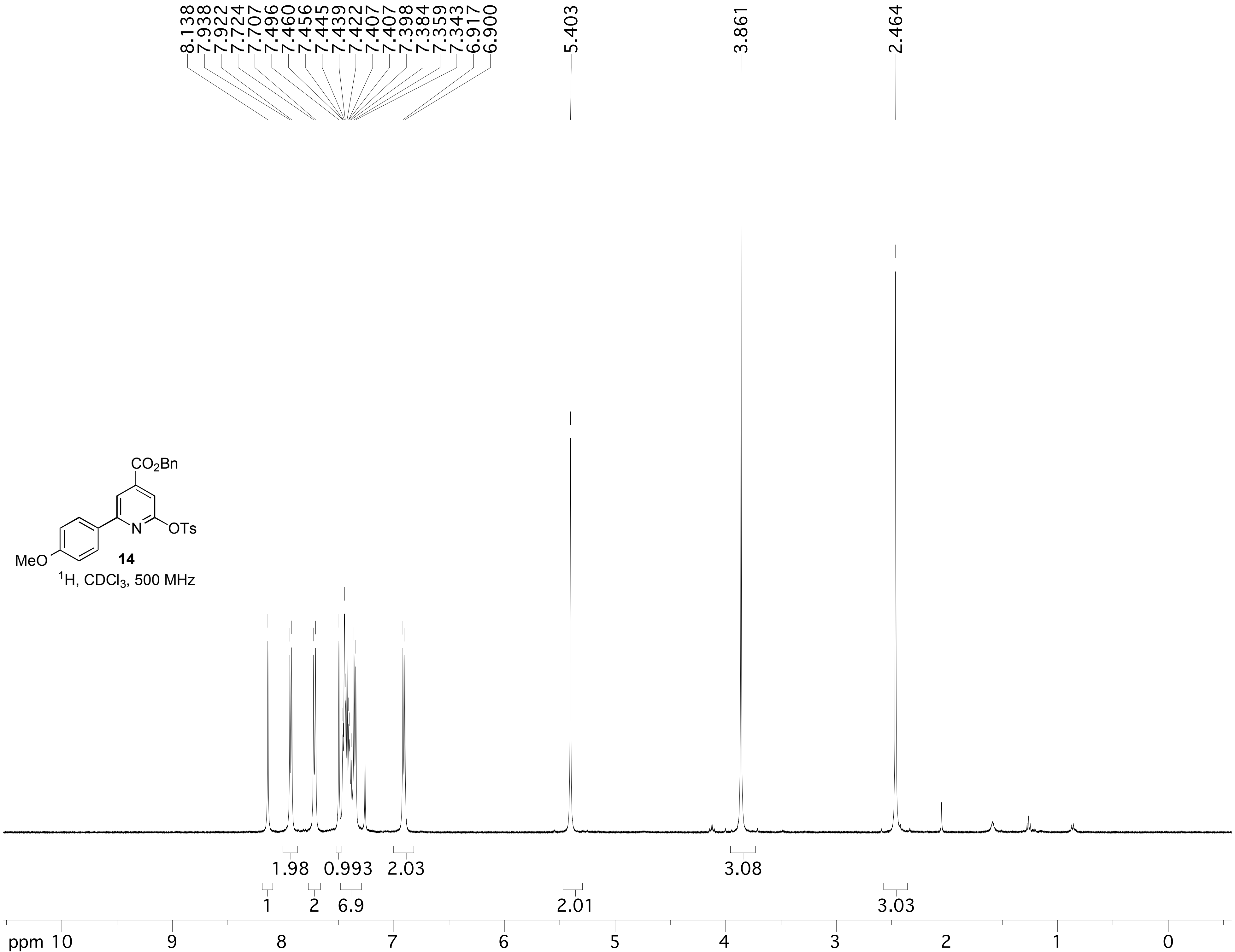

8.138
7.938
7.922
7.724
7.707
7.496
7.460
7.456
7.445
7.439
7.422
7.407
7.407
7.398
7.384
7.359
7.343
6.917
6.900
5.403
3.861
2.464
CO2Bn
N
OTs
MeO
14
1H, CDCl3, 500 MHz
1.98
0.993
2.03
3.08
1
2
6.9
2.01
3.03
ppm 10
9
8
7
6
5
4
3
2
1
0

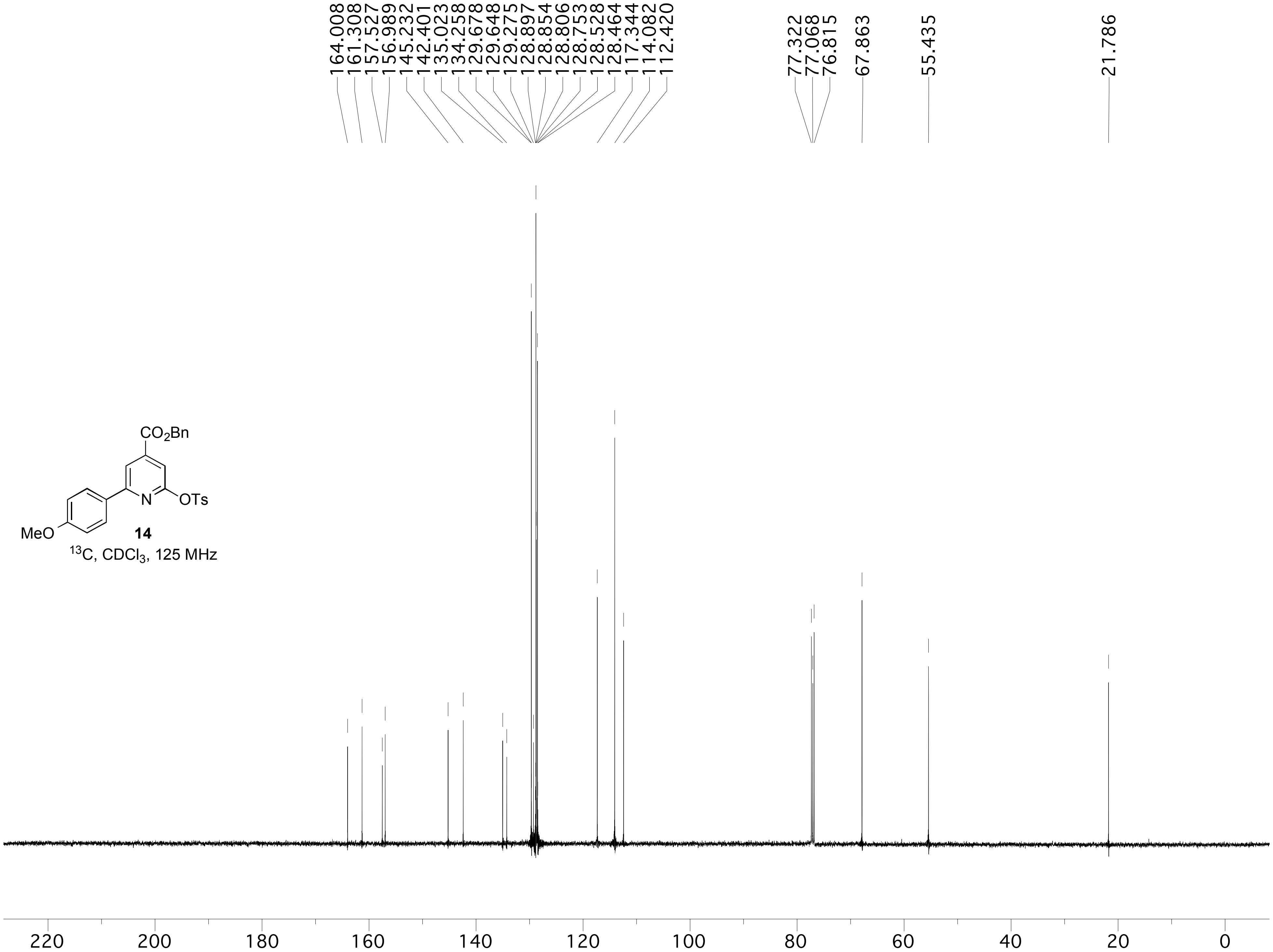
164.008
161.308
157.527
156.989
145.232
142.401
135.023
134.258
129.678
129.648
129.275
128.897
128.854
128.806
128.753
128.528
128.464
117.344
114.082
112.420
77.322
77.068
76.815
67.863
55.435
21.786
CO2Bn
N
OTs
MeO
14
13C, CDCl3, 125 MHz
220
200
180
160
140
120
100
80
60
40
20
0

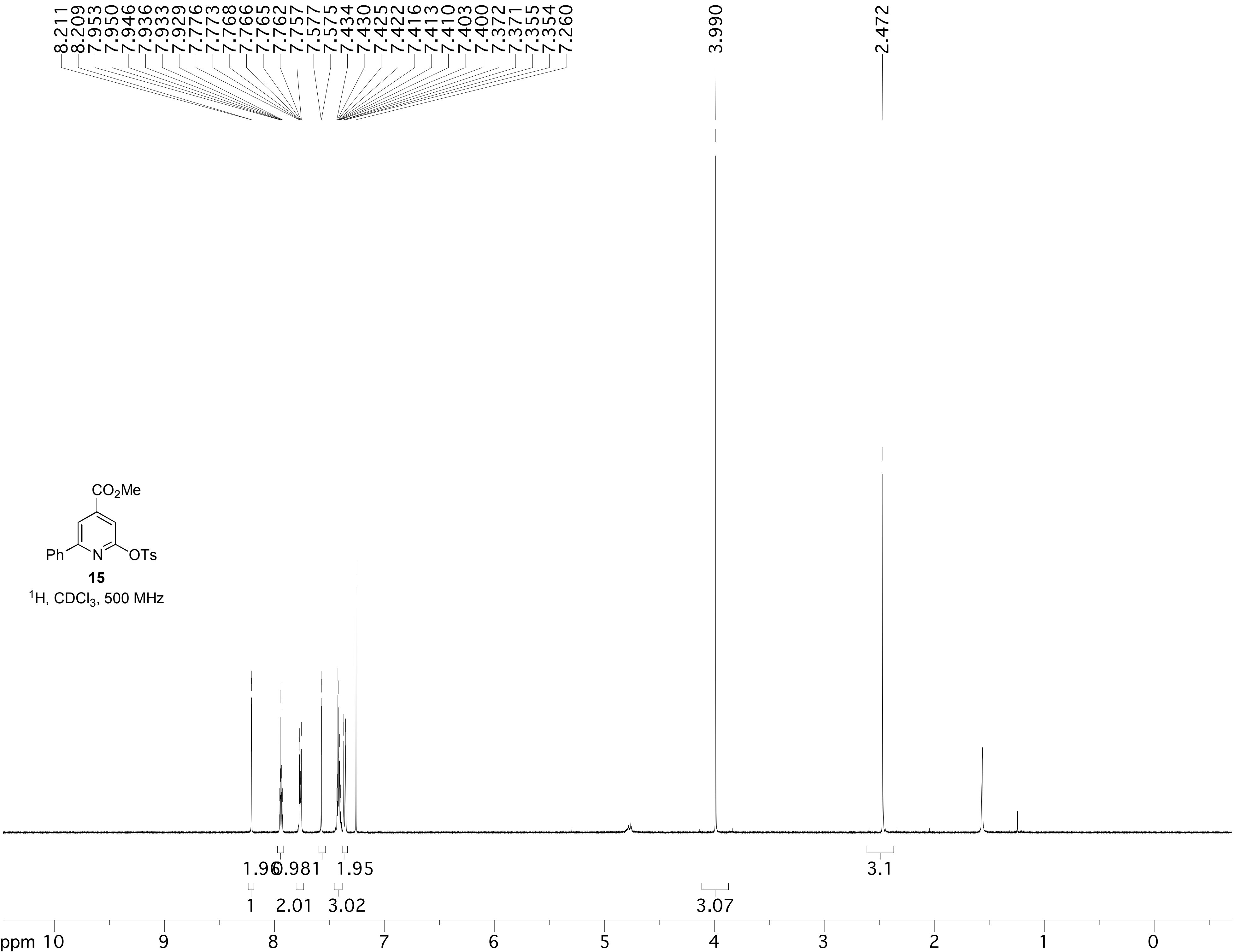
8.211
8.209
7.953
7.950
7.946
7.936
7.933
7.929
7.776
7.773
7.768
7.766
7.765
7.762
7.757
7.577
7.575
7.434
7.430
7.425
7.422
7.416
7.413
7.410
7.403
7.400
7.372
7.371
7.355
7.354
7.260
3.990
2.472
CO2Me
Ph
N
OTs
15
1H, CDCl3, 500 MHz
1.96
0.981
1.95
3.1
1
2.01
3.02
3.07
ppm 10
9
8
7
6
5
4
3
2
1
0

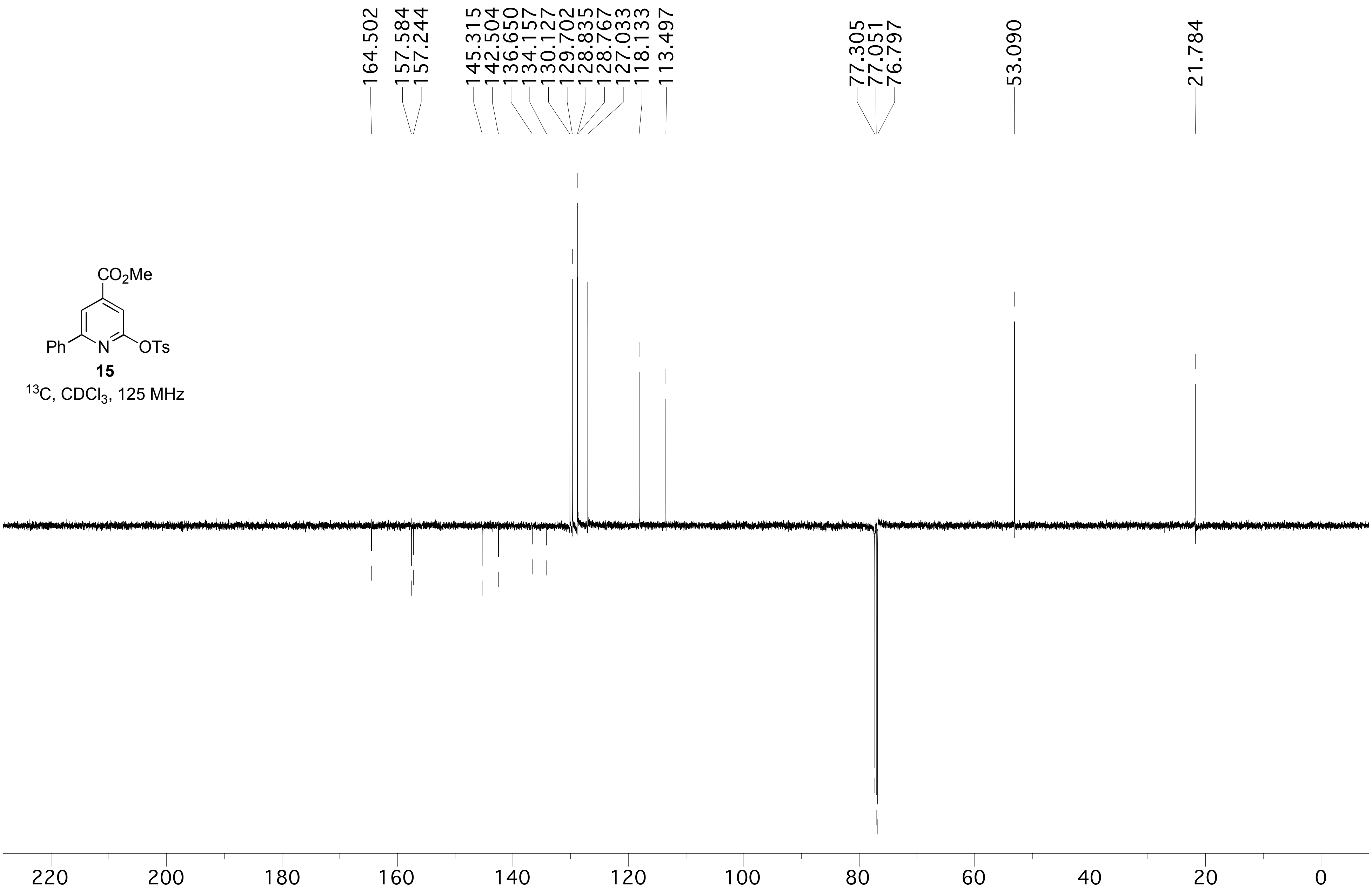

15

^{13}C, $CDCl_3$, 125 MHz

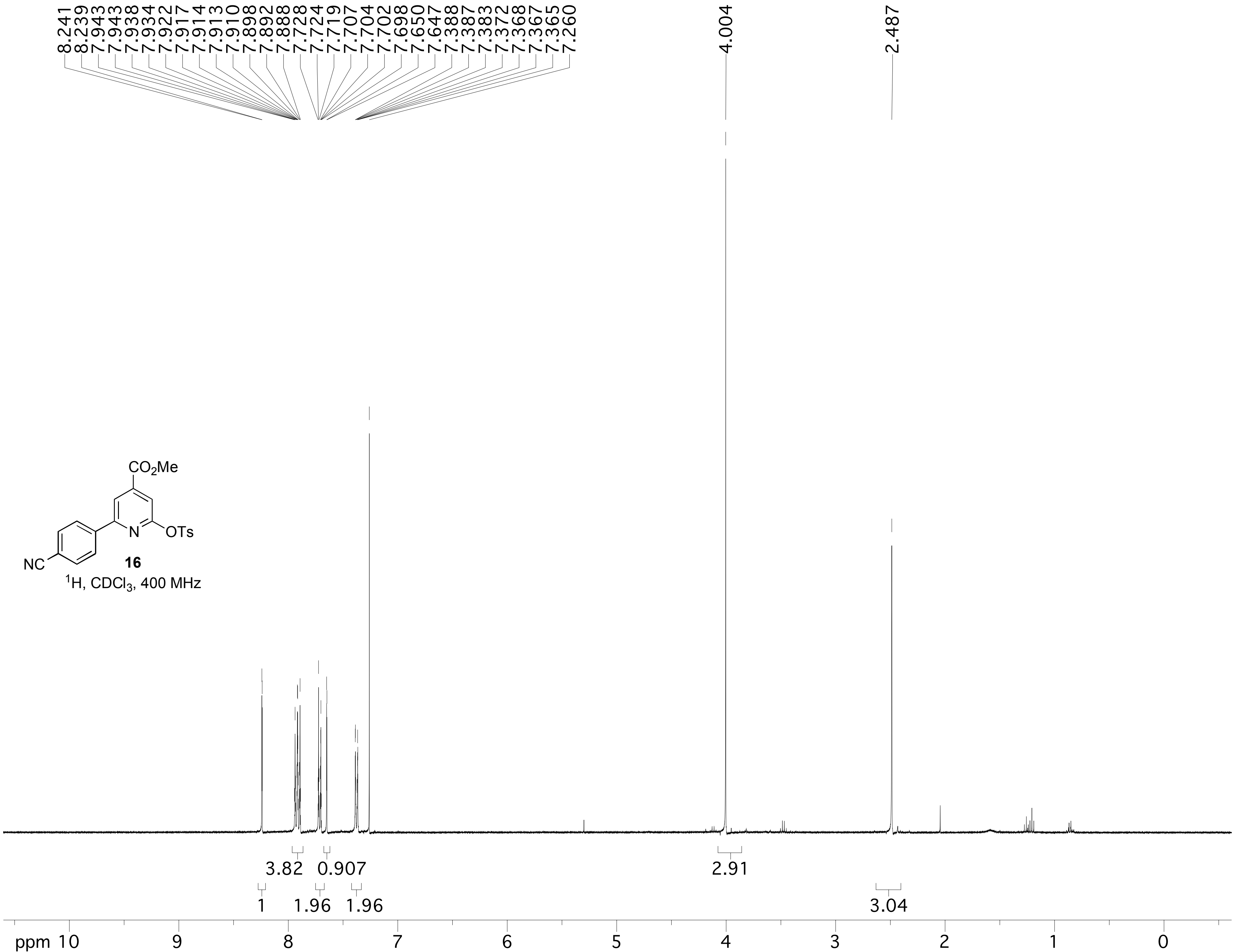
8.241
8.239
7.943
7.943
7.938
7.934
7.922
7.917
7.914
7.913
7.910
7.898
7.892
7.888
7.728
7.724
7.719
7.707
7.704
7.702
7.698
7.650
7.647
7.388
7.387
7.383
7.372
7.368
7.367
7.365
7.260
4.004
2.487
CO2Me
N
OTs
NC
16
1H, CDCl3, 400 MHz
3.82
0.907
2.91
1
1.96
1.96
3.04
ppm 10
9
8
7
6
5
4
3
2
1
0

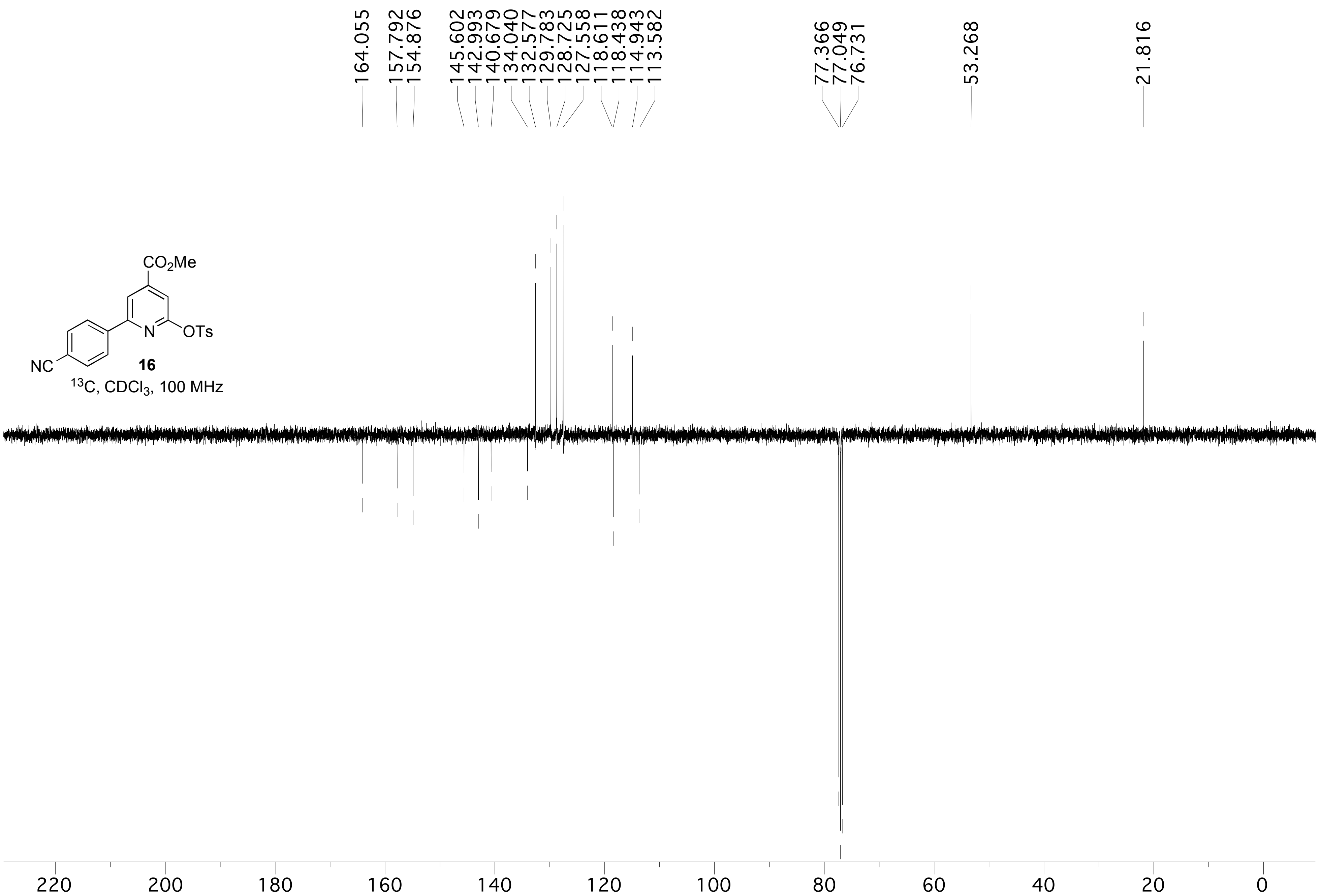

164.055
157.792
154.876
145.602
142.993
140.679
134.040
132.577
129.783
128.725
127.558
118.611
118.438
114.943
113.582
77.366
77.049
76.731
53.268
21.816
CO2Me
N
OTs
NC
16
13C, CDCl3, 100 MHz
220
200
180
160
140
120
100
80
60
40
20
0

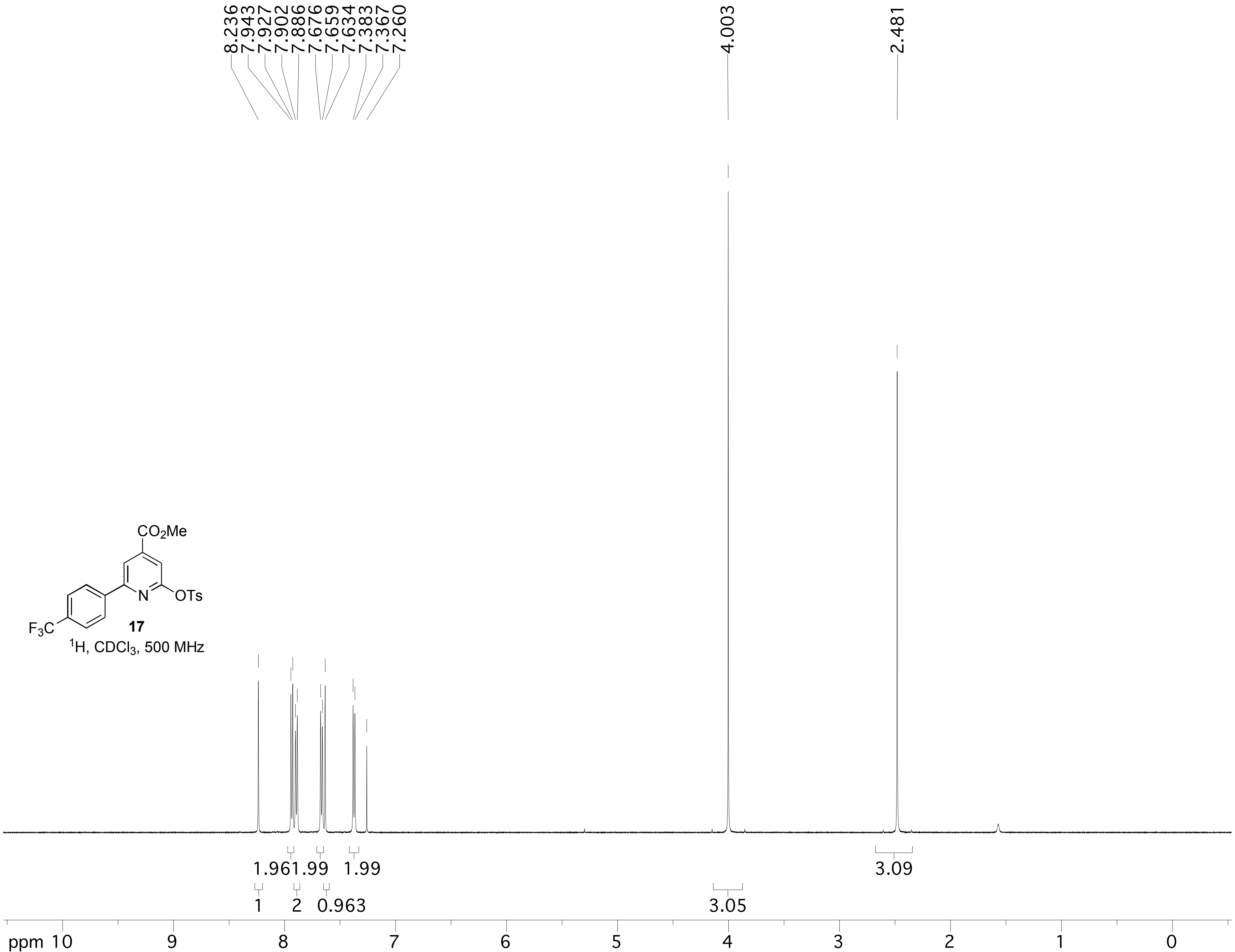

8.236
7.943
7.927
7.902
7.886
7.676
7.659
7.634
7.383
7.367
7.260
4.003
2.481
CO2Me
N
OTs
F3C
17
1H, CDCl3, 500 MHz
1.96
1.99
1.99
3.09
1
2
0.963
3.05
ppm 10
9
8
7
6
5
4
3
2
1
0

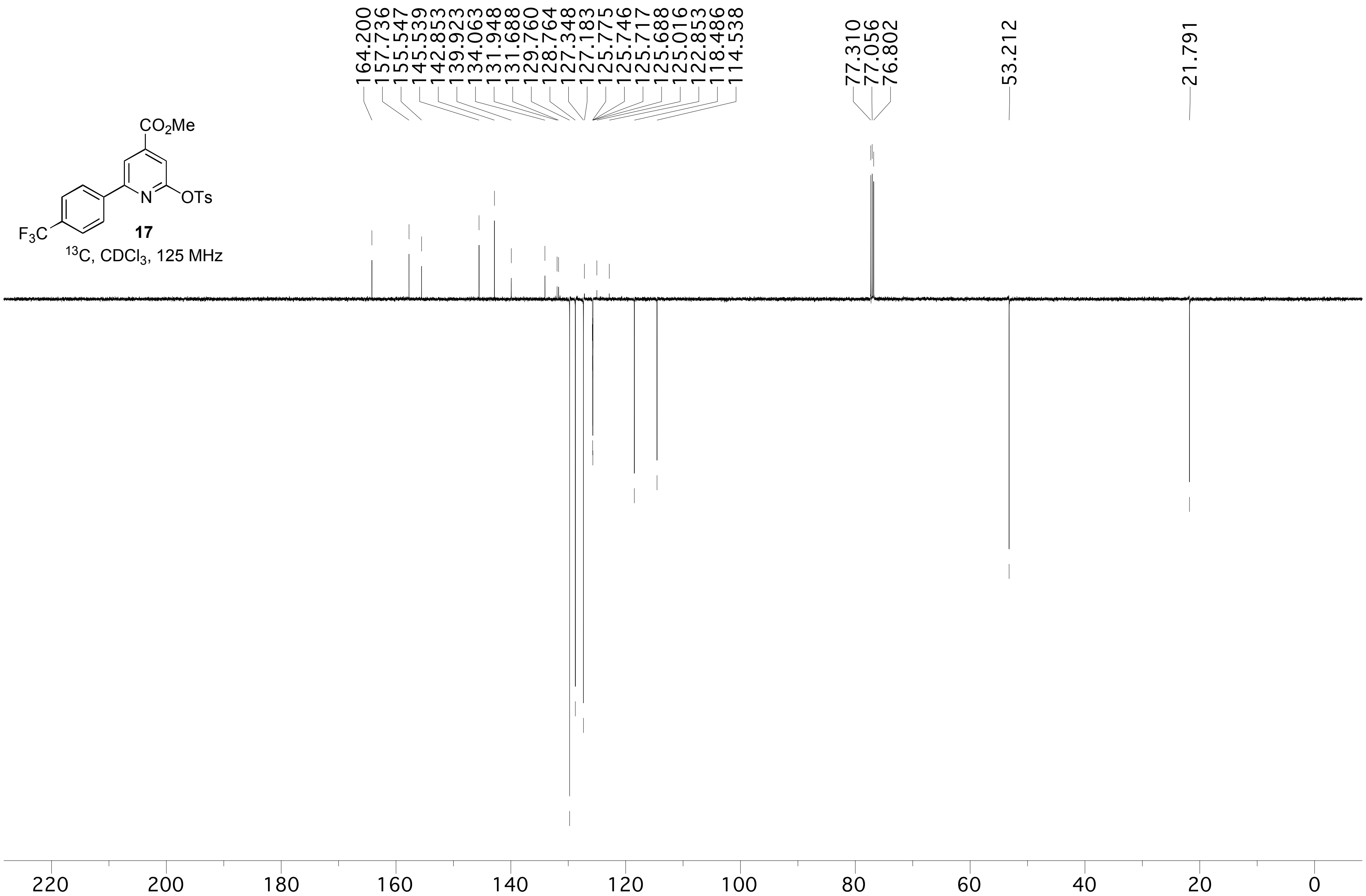

CO2Me
N
OTs
F3C
17
13C, CDCl3, 125 MHz
164.200
157.736
155.547
145.539
142.853
139.923
134.063
131.948
131.688
129.760
128.764
127.348
127.183
125.775
125.746
125.717
125.688
125.016
122.853
118.486
114.538
77.310
77.056
76.802
53.212
21.791
220
200
180
160
140
120
100
80
60
40
20
0

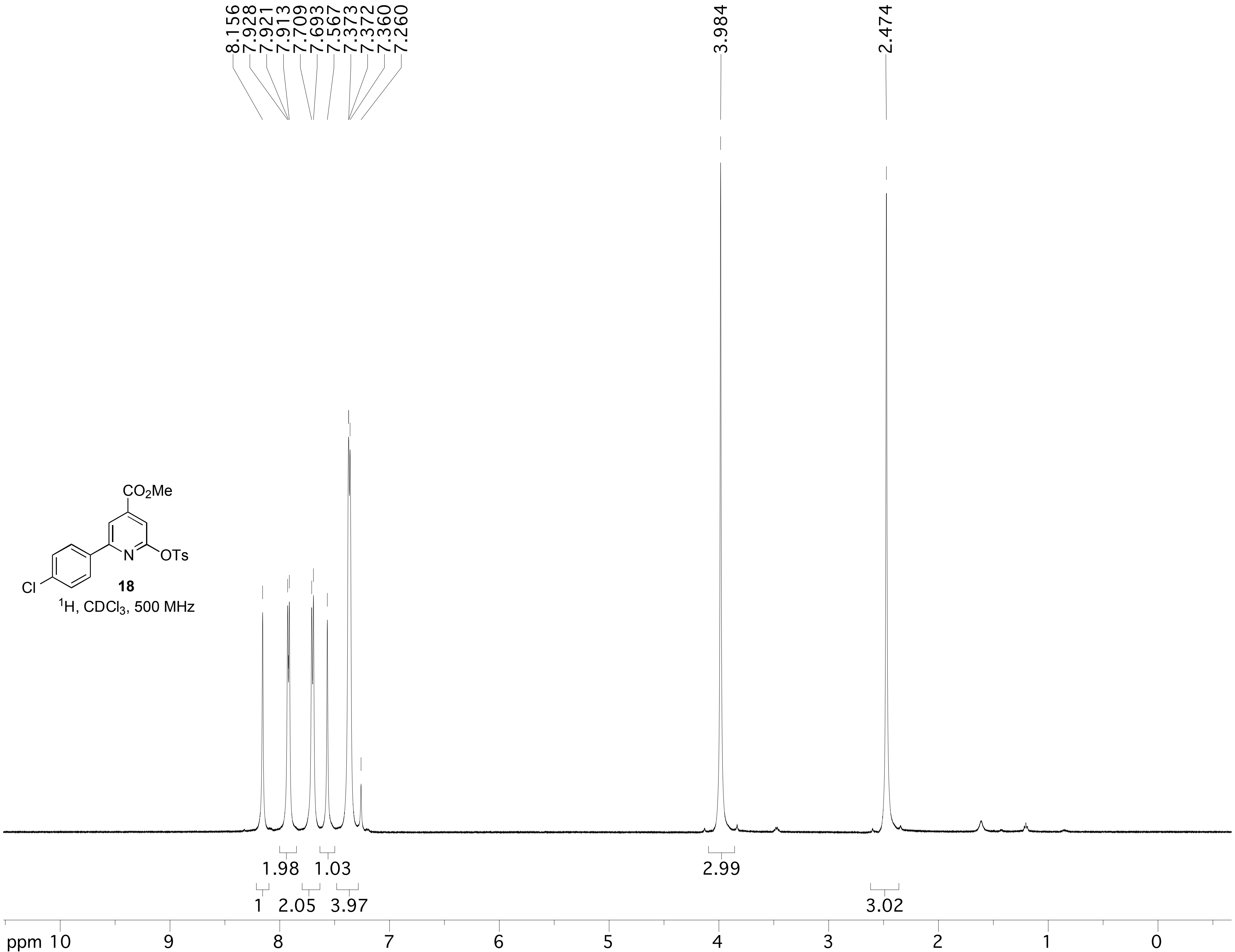

8.156
7.928
7.921
7.913
7.709
7.693
7.567
7.373
7.372
7.360
7.260
3.984
2.474
CO2Me
N
OTs
Cl
18
1H, CDCl3, 500 MHz
1.98
1.03
2.99
1
2.05
3.97
3.02
ppm 10
9
8
7
6
5
4
3
2
1
0

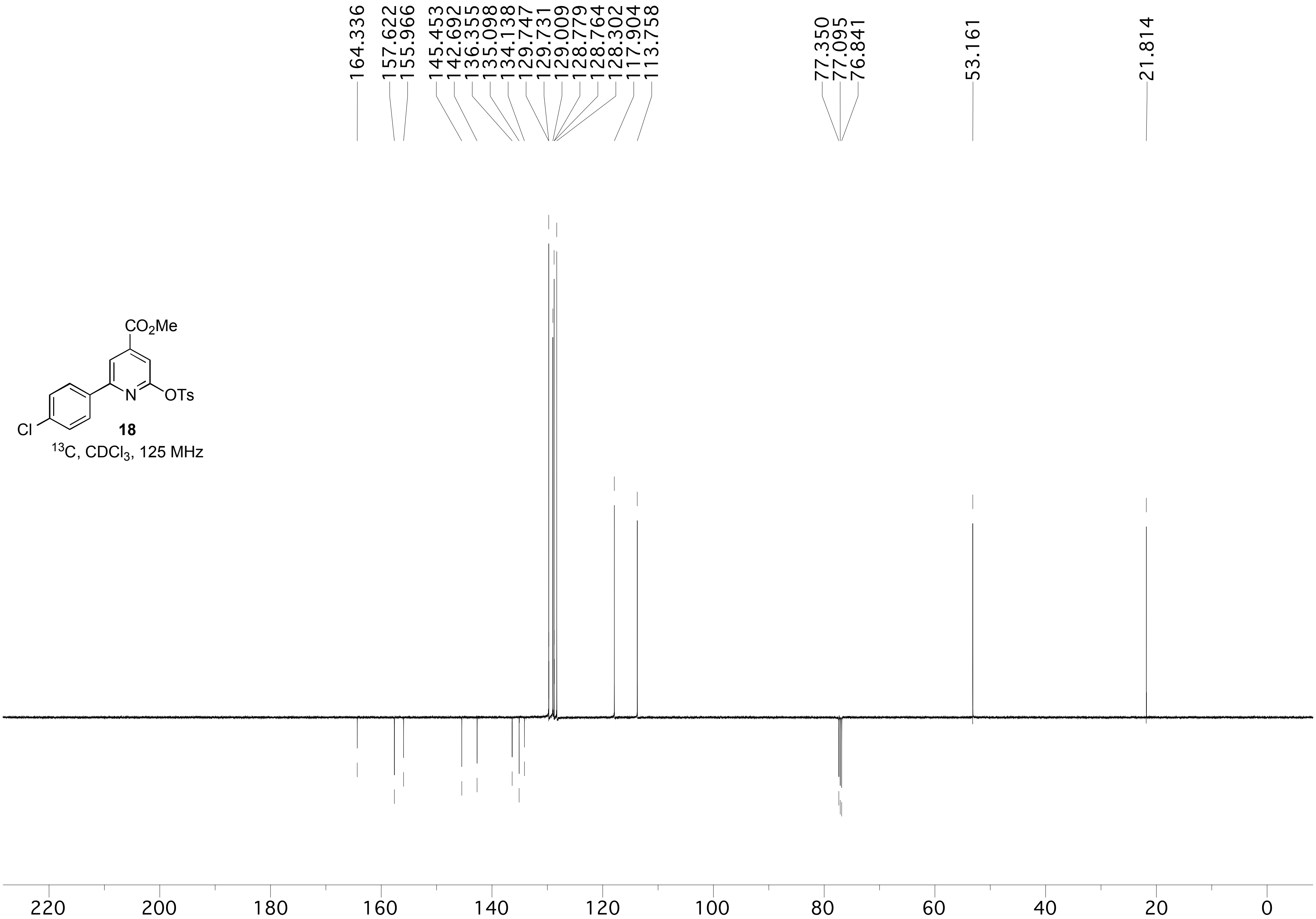
164.336
157.622
155.966
145.453
142.692
136.355
135.098
134.138
129.747
129.731
129.009
128.779
128.764
128.302
117.904
113.758
77.350
77.095
76.841
53.161
21.814
CO_2Me
N
OTs
Cl
18
^{13}C, $CDCl_3$, 125 MHz
220
200
180
160
140
120
100
80
60
40
20
0

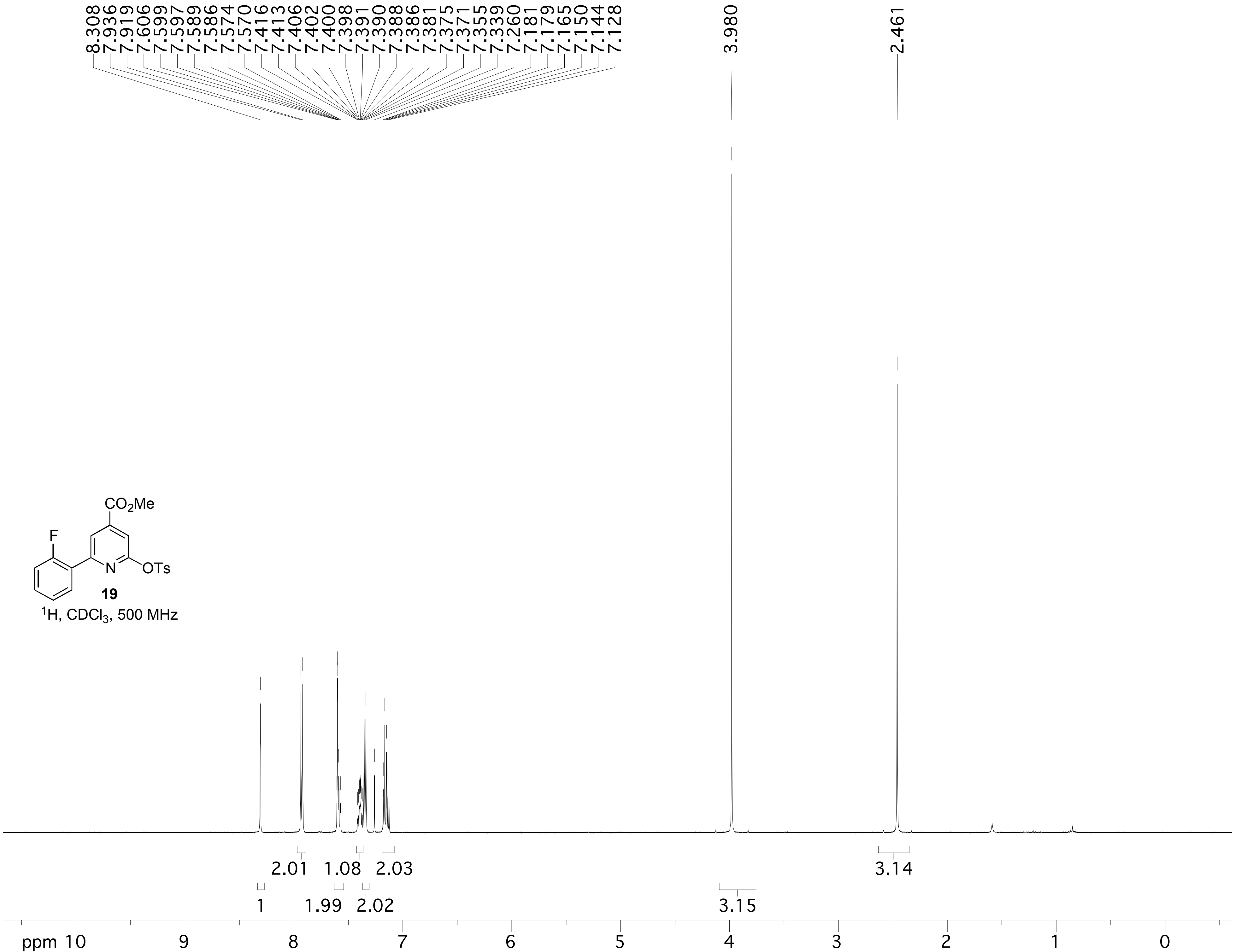
8.308
7.936
7.919
7.606
7.599
7.597
7.589
7.586
7.574
7.570
7.416
7.413
7.406
7.402
7.400
7.398
7.391
7.390
7.388
7.386
7.381
7.375
7.371
7.355
7.339
7.260
7.181
7.179
7.165
7.150
7.144
7.128
3.980
2.461
CO2Me
F
N
OTs
19
1H, CDCl3, 500 MHz
2.01
1.08
2.03
3.14
1
1.99
2.02
3.15
ppm 10
9
8
7
6
5
4
3
2
1
0

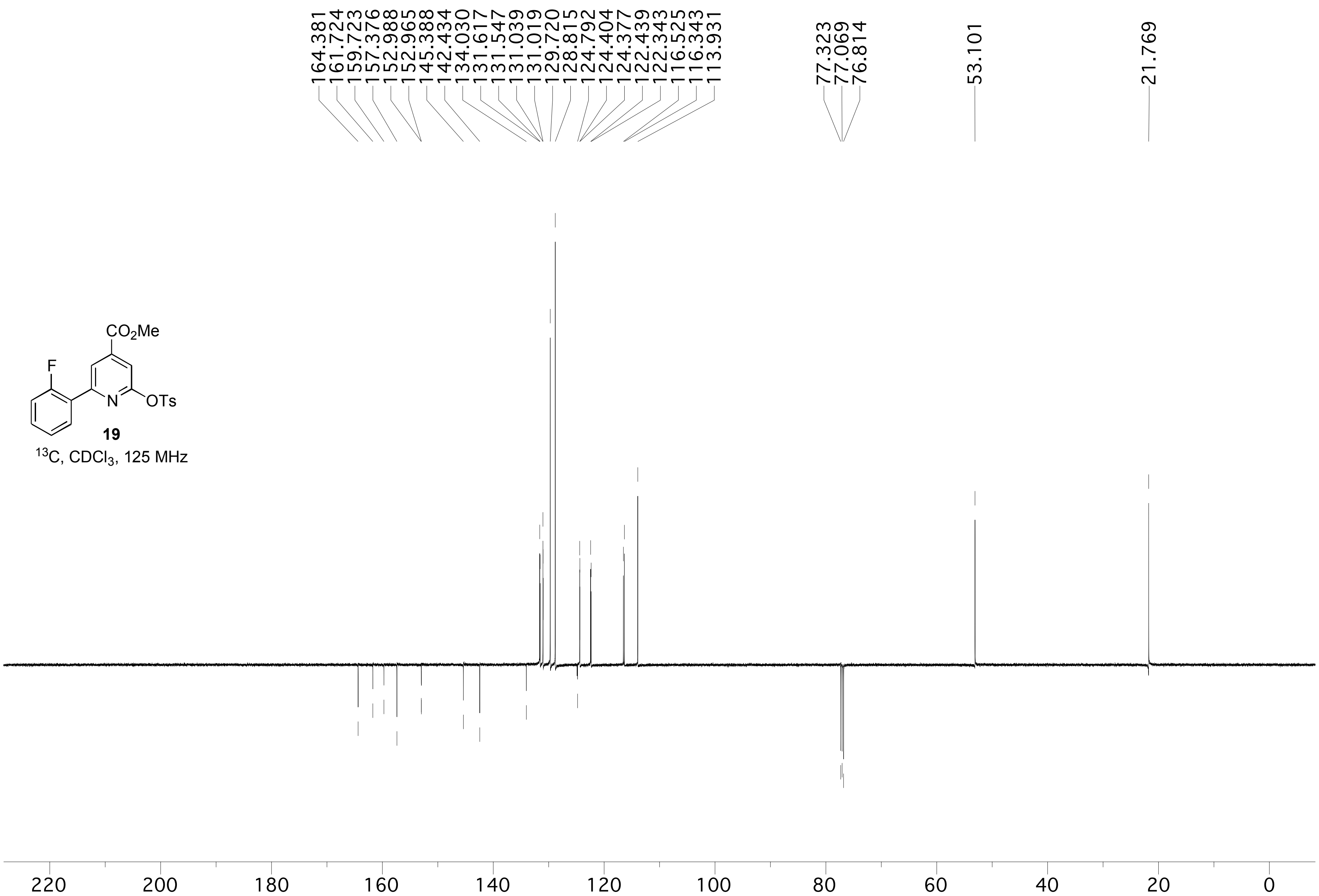
164.381
161.724
159.723
157.376
152.988
152.965
145.388
142.434
134.030
131.617
131.547
131.039
131.019
129.720
128.815
124.792
124.404
124.377
122.439
122.343
116.525
116.343
113.931
77.323
77.069
76.814
53.101
21.769
CO_2Me
F
N
OTs
19
^{13}C, $CDCl_3$, 125 MHz
220
200
180
160
140
120
100
80
60
40
20
0

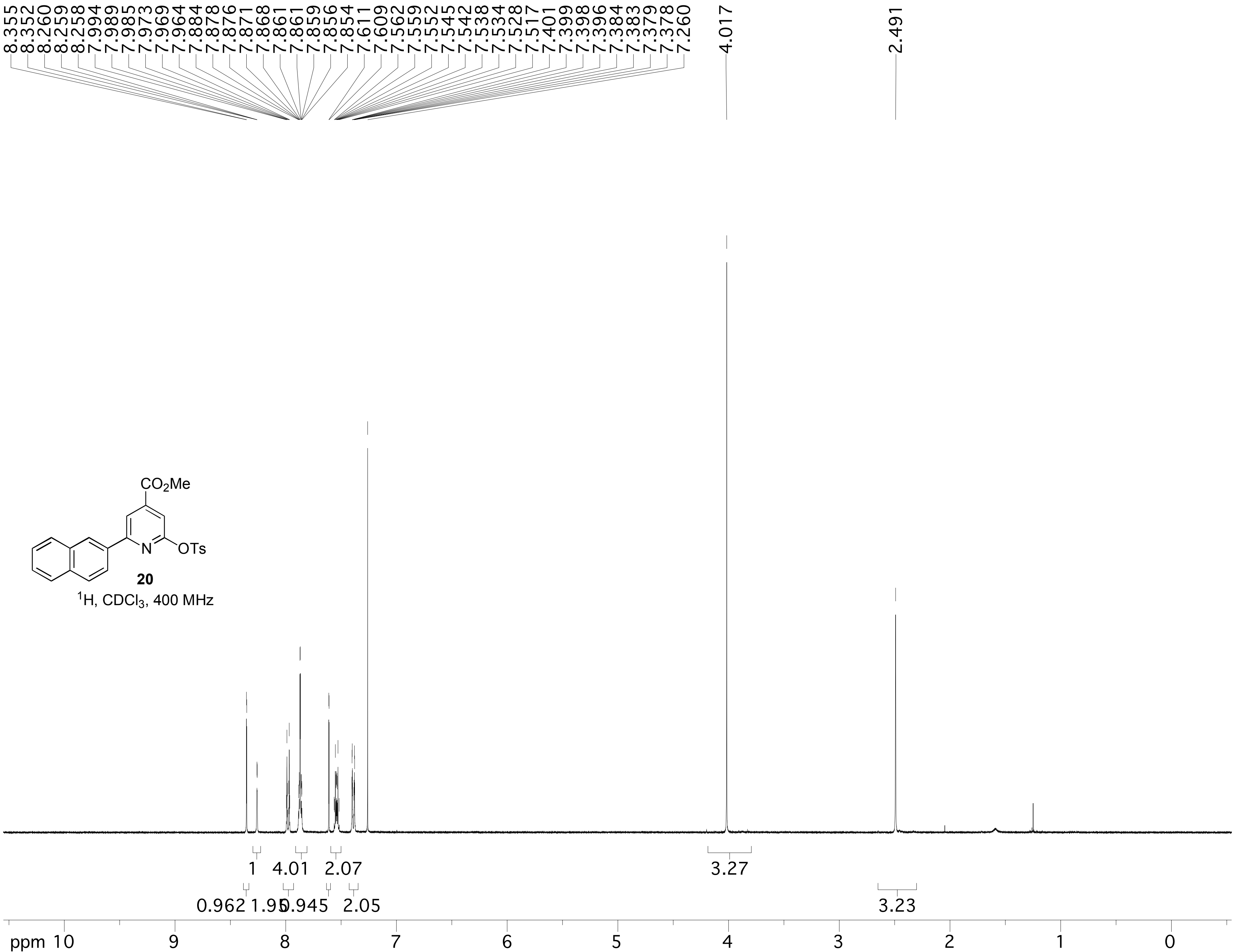

8.355 8.352 8.260 8.259 8.258 7.994 7.989 7.985 7.973 7.969 7.964 7.884 7.878 7.876 7.871 7.868 7.861 7.861 7.859 7.856 7.854 7.611 7.609 7.562 7.559 7.552 7.545 7.542 7.538 7.534 7.528 7.517 7.401 7.399 7.398 7.396 7.384 7.383 7.379 7.378 7.260
4.017
2.491
CO2Me
N
OTs
20
1H, CDCl3, 400 MHz
1
4.01
2.07
3.27
0.962
1.95
0.945
2.05
3.23
ppm 10 9 8 7 6 5 4 3 2 1 0

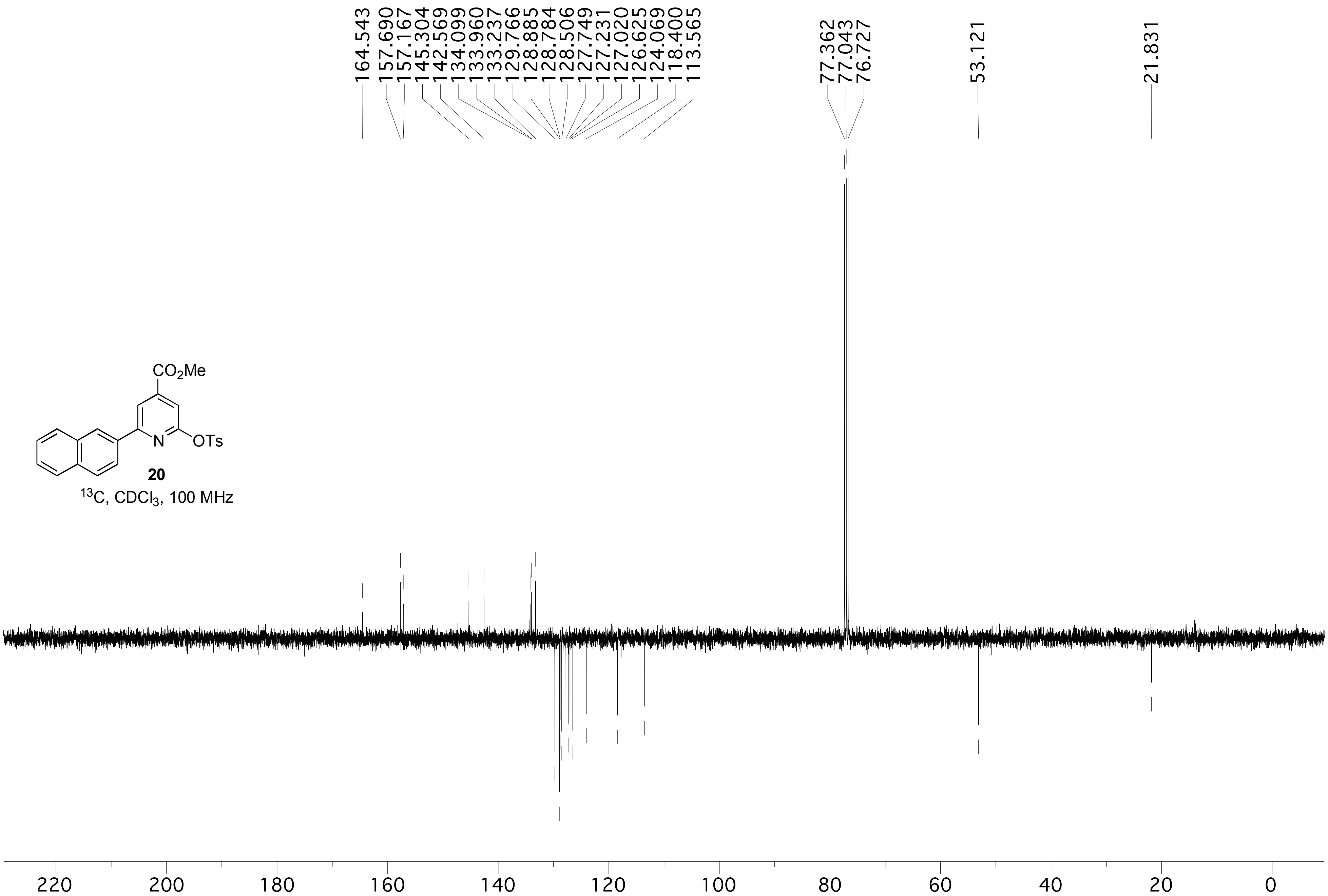
164.543
157.690
157.167
145.304
142.569
134.099
133.960
133.237
129.766
128.885
128.784
128.506
127.749
127.231
127.020
126.625
124.069
118.400
113.565
77.362
77.043
76.727
53.121
21.831
CO2Me
N
OTs
20
13C, CDCl3, 100 MHz
220
200
180
160
140
120
100
80
60
40
20
0

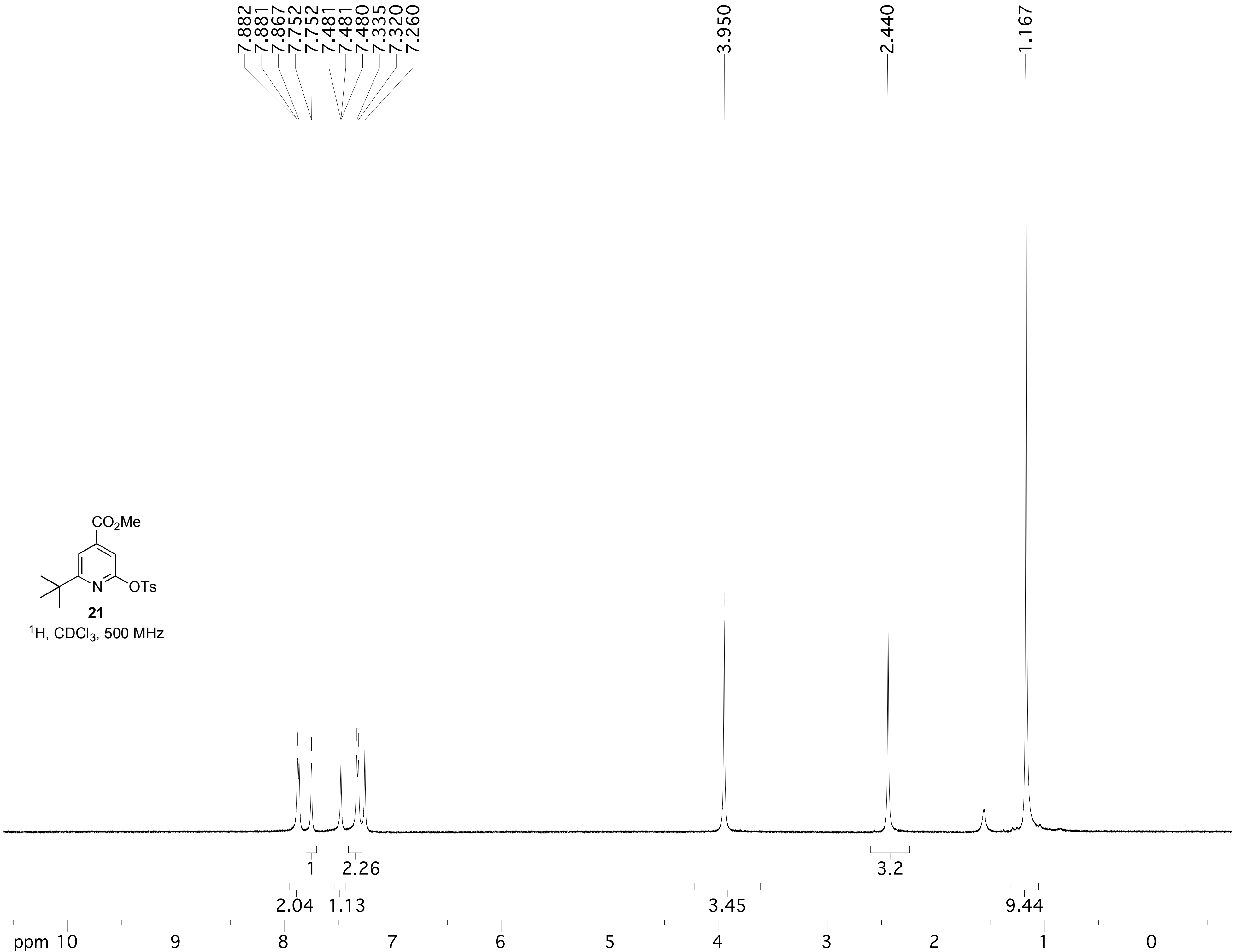
7.882
7.881
7.867
7.752
7.752
7.481
7.481
7.480
7.335
7.320
7.260
3.950
2.440
1.167
CO2Me
N
OTs
21
1H, CDCl3, 500 MHz
1
2.26
3.2
2.04
1.13
3.45
9.44
ppm 10
9
8
7
6
5
4
3
2
1
0

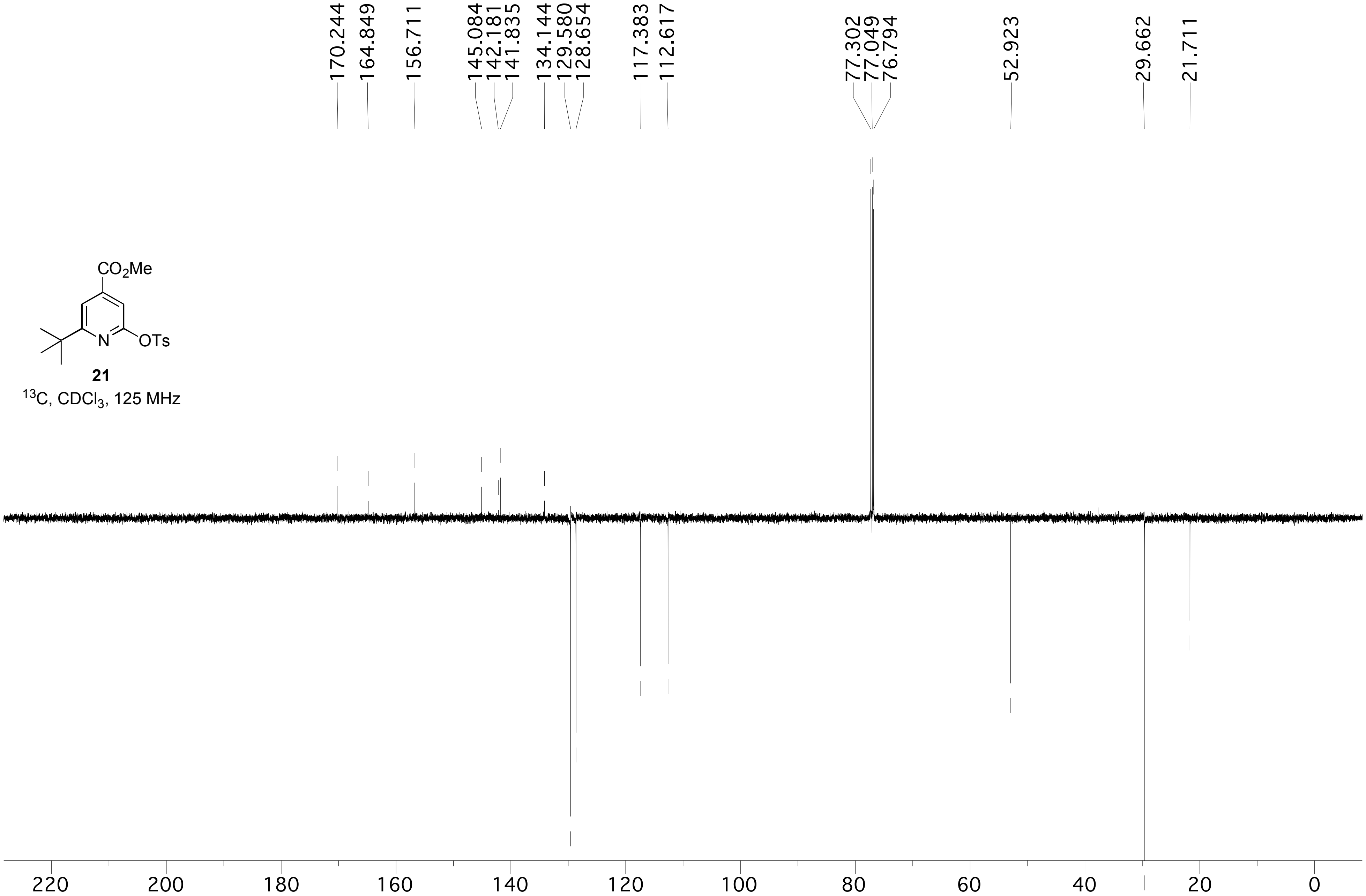

170.244
164.849
156.711
145.084
142.181
141.835
134.144
129.580
128.654
117.383
112.617
77.302
77.049
76.794
52.923
29.662
21.711
CO2Me
N
OTs
21
13C, CDCl3, 125 MHz
220
200
180
160
140
120
100
80
60
40
20
0

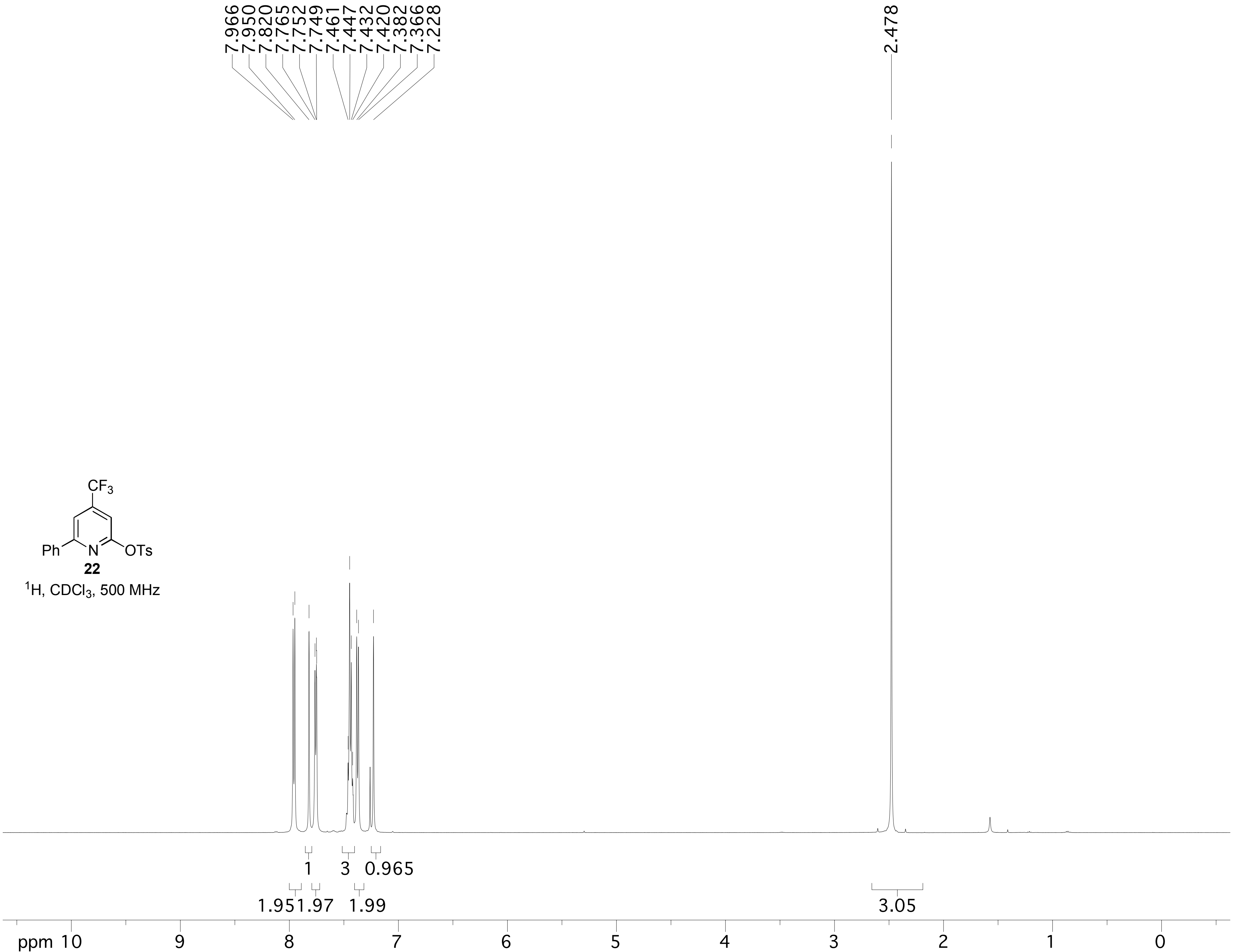

CF3
Ph
N
OTs
22
1H, CDCl3, 500 MHz
7.966
7.950
7.820
7.765
7.752
7.749
7.461
7.447
7.432
7.420
7.382
7.366
7.228
2.478
1
3
0.965
1.95
1.97
1.99
3.05
ppm 10
9
8
7
6
5
4
3
2
1
0

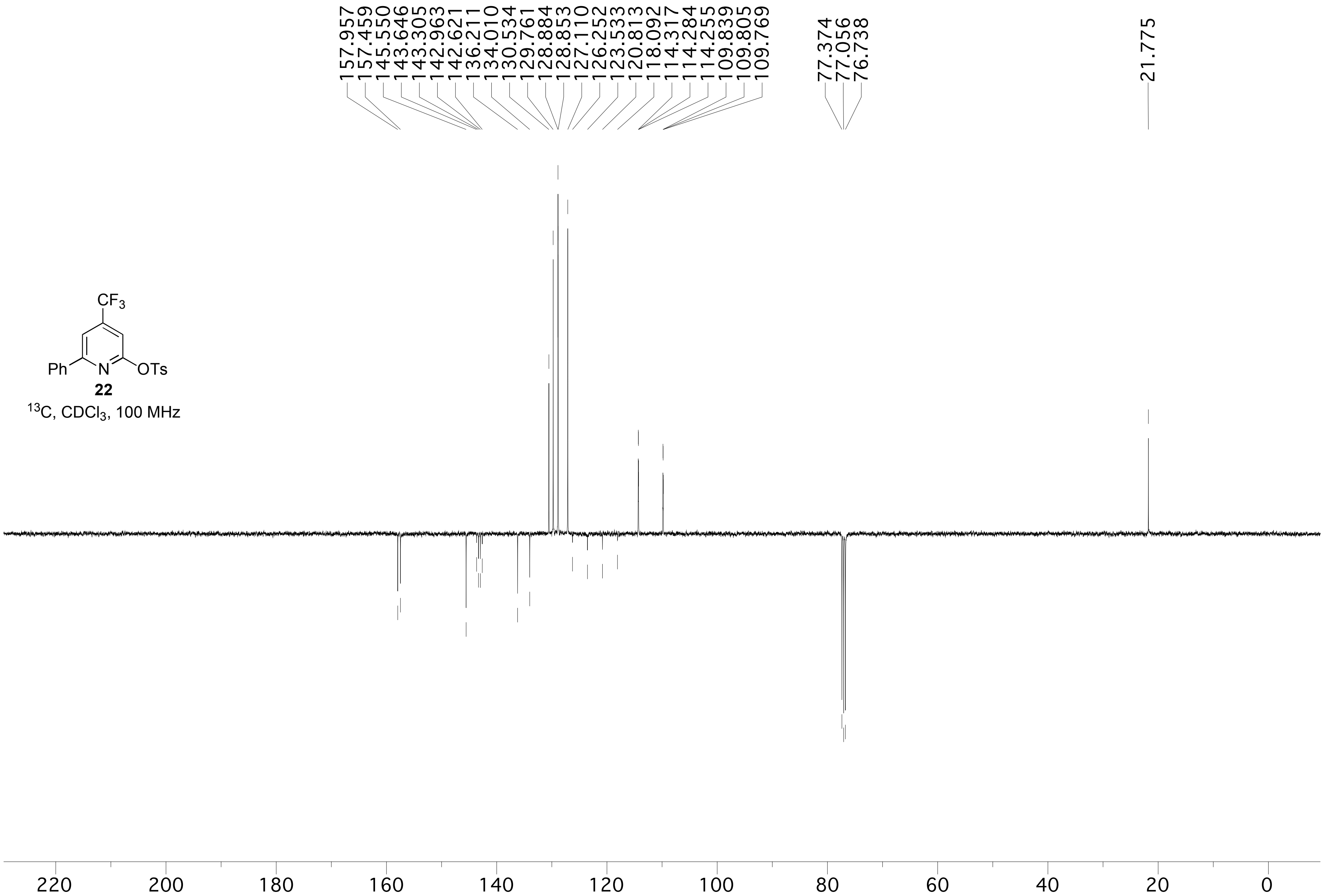
157.957
157.459
145.550
143.646
143.305
142.963
142.621
136.211
134.010
130.534
129.761
128.884
128.853
127.110
126.252
123.533
120.813
118.092
114.317
114.284
114.255
109.839
109.805
109.769
77.374
77.056
76.738
21.775
CF3
Ph
N
OTs
22
13C, CDCl3, 100 MHz
220
200
180
160
140
120
100
80
60
40
20
0

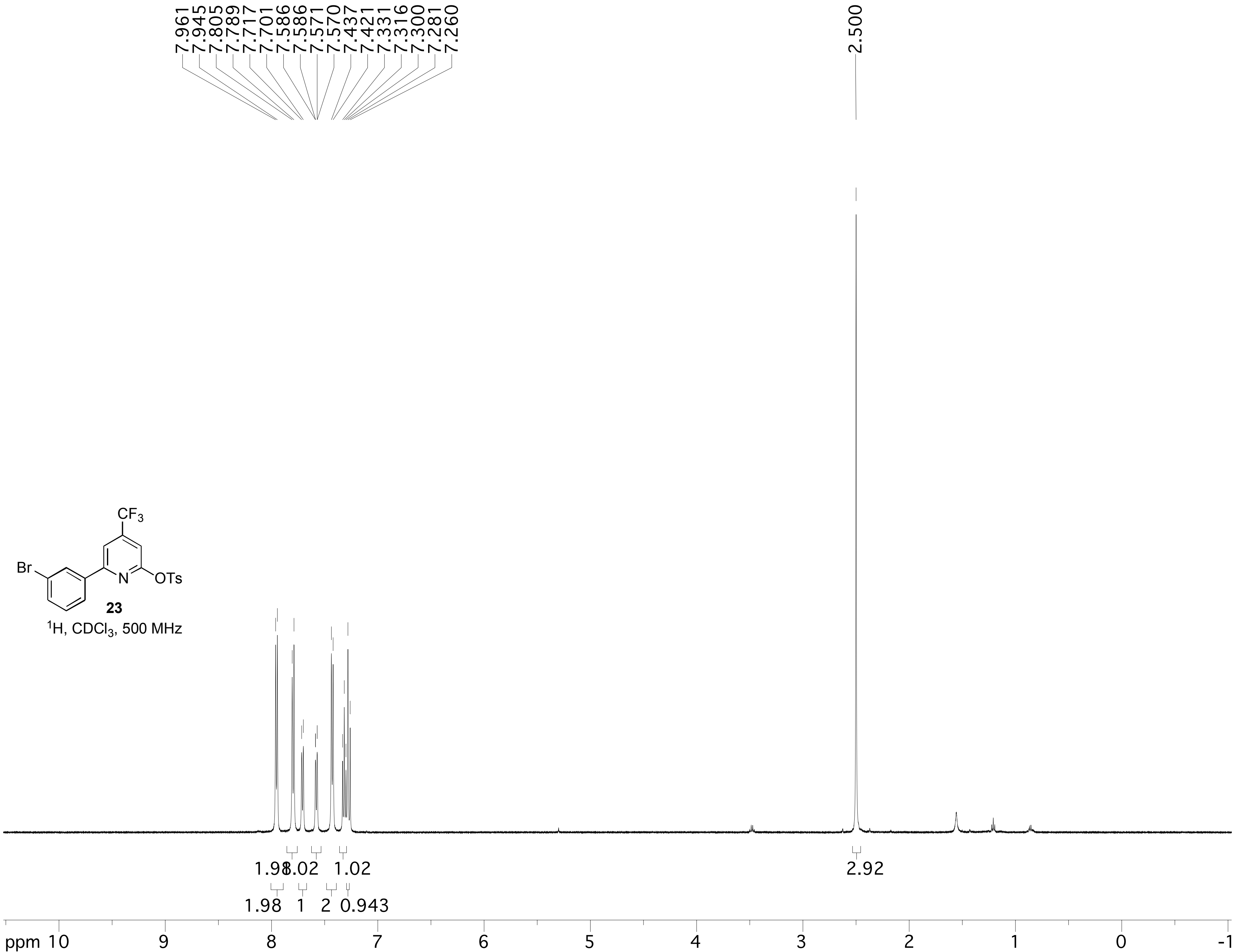

7.961
7.945
7.805
7.789
7.717
7.701
7.586
7.586
7.571
7.570
7.437
7.421
7.331
7.316
7.300
7.281
7.260
2.500
CF3
Br
N
OTs
23
1H, CDCl3, 500 MHz
1.98
1.02
1.02
2.92
1.98
1
2
0.943
ppm 10
9
8
7
6
5
4
3
2
1
0
-1

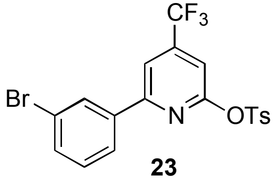

23

^{13}C, $CDCl_3$, 125 MHz

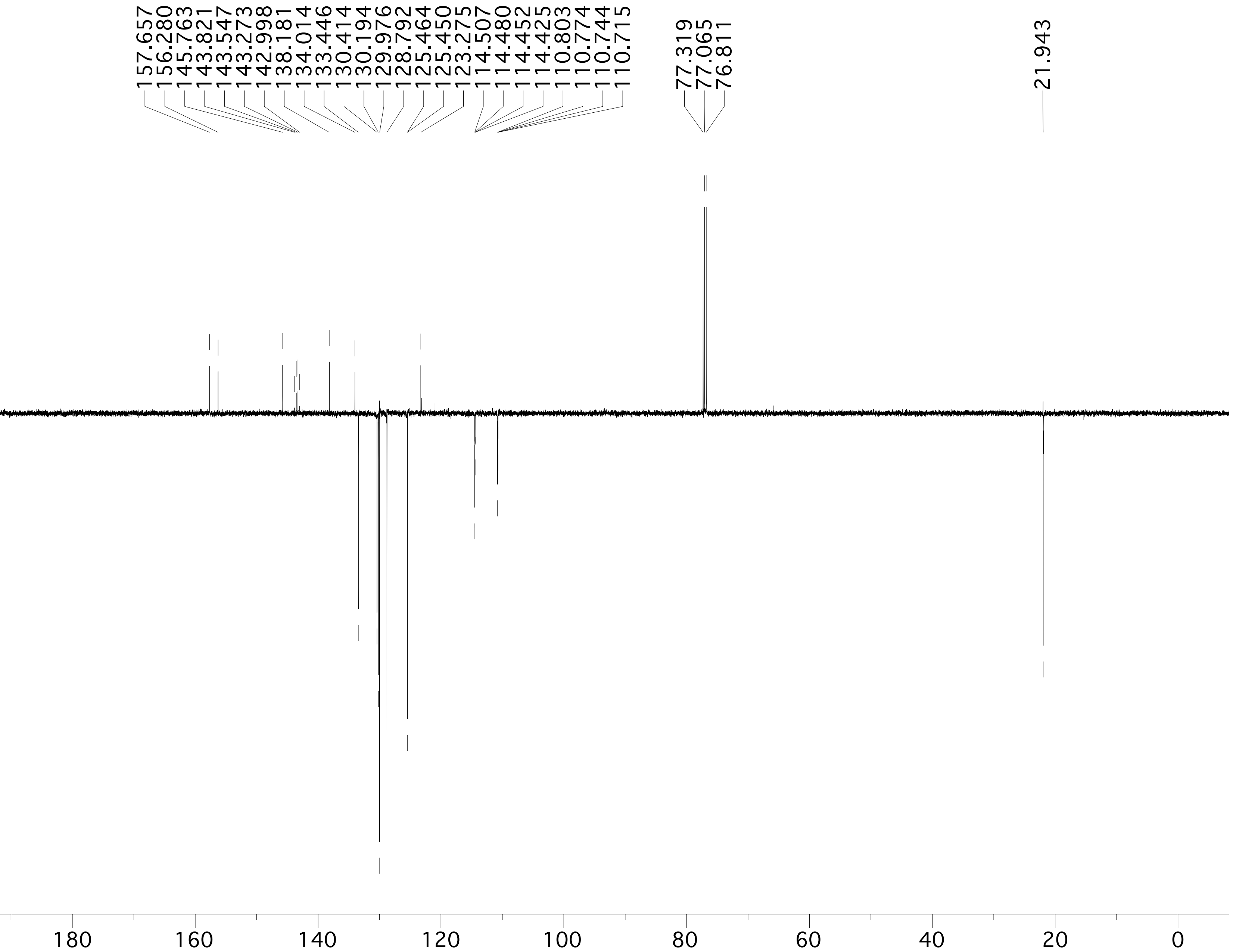

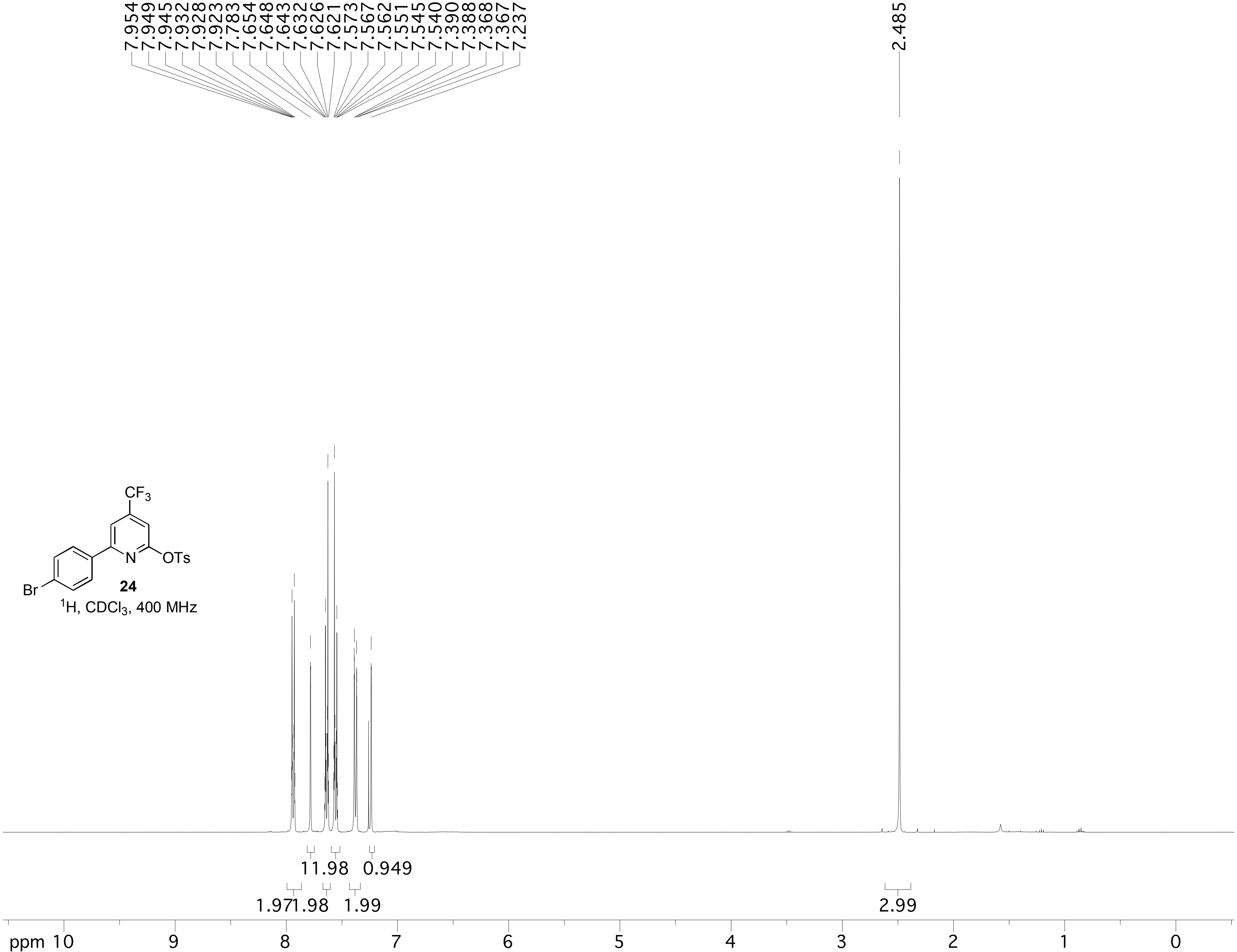
7.954
7.949
7.945
7.932
7.928
7.923
7.783
7.654
7.648
7.643
7.632
7.626
7.621
7.573
7.567
7.562
7.551
7.545
7.540
7.390
7.388
7.368
7.367
7.237
2.485
CF3
N
OTs
Br
24
1H, CDCl3, 400 MHz
11.98
0.949
1.97
1.98
1.99
2.99
ppm 10
9
8
7
6
5
4
3
2
1
0

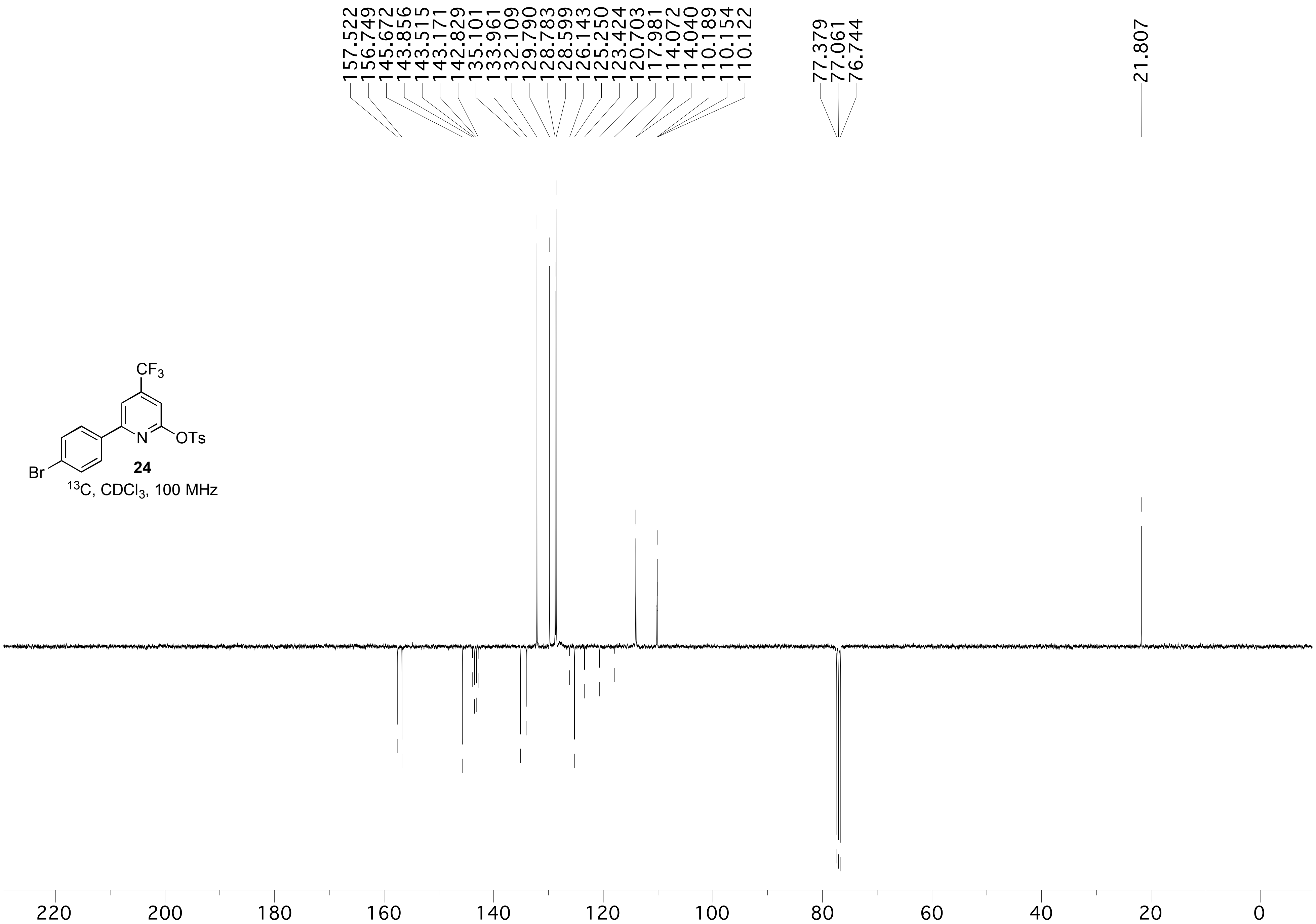
157.522
156.749
145.672
143.856
143.515
143.171
142.829
135.101
133.961
132.109
129.790
128.783
128.599
126.143
125.250
123.424
120.703
117.981
114.072
114.040
110.189
110.154
110.122
77.379
77.061
76.744
21.807
CF3
N
OTs
Br
24
13C, CDCl3, 100 MHz
220
200
180
160
140
120
100
80
60
40
20
0

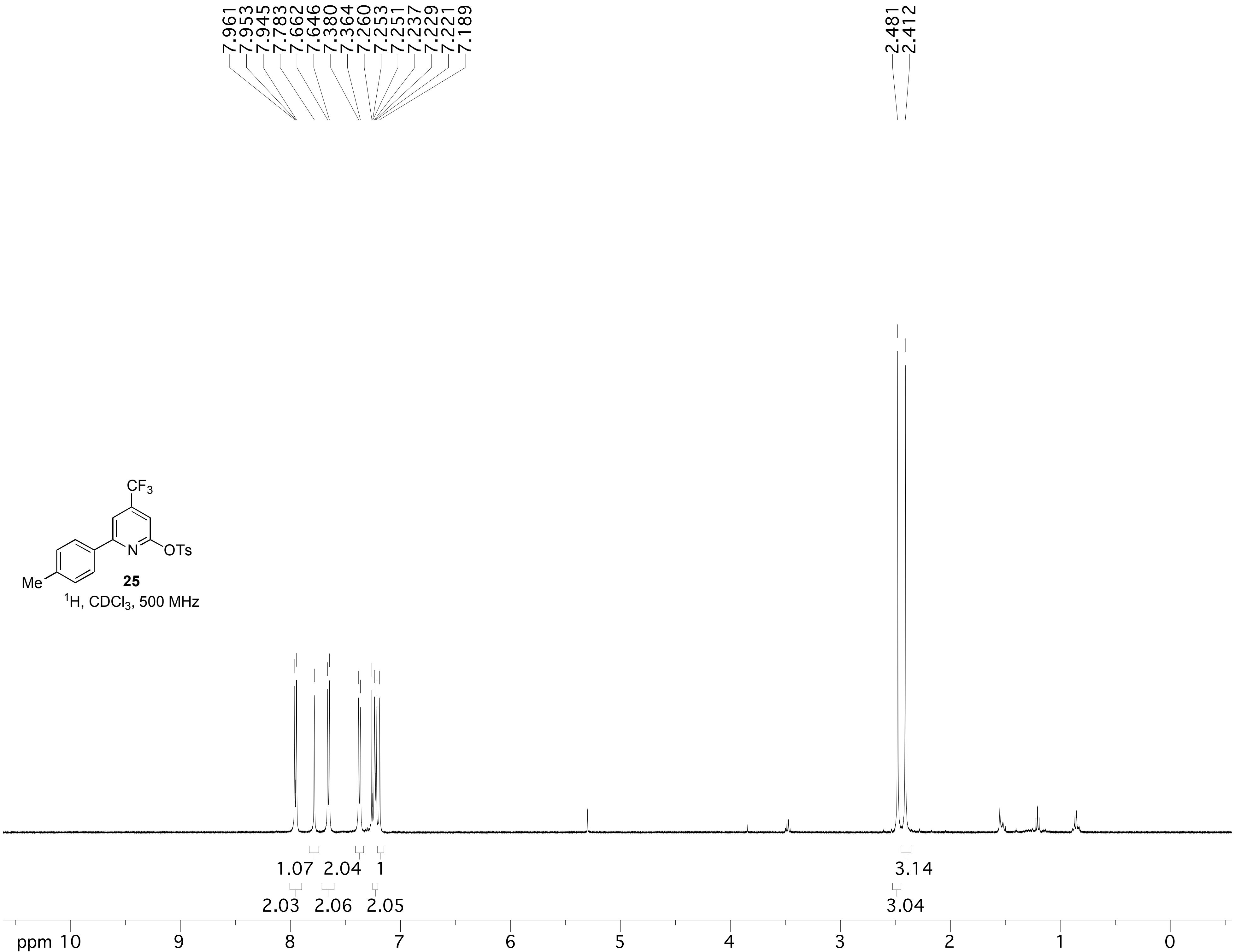

7.961
7.953
7.945
7.783
7.662
7.646
7.380
7.364
7.260
7.253
7.251
7.237
7.229
7.221
7.189
2.481
2.412
CF3
N
OTs
Me
25
1H, CDCl3, 500 MHz
1.07
2.04
1
3.14
2.03
2.06
2.05
3.04
ppm 10
9
8
7
6
5
4
3
2
1
0

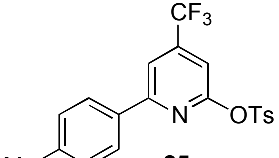

25

^{13}C, $CDCl_3$, 125 MHz

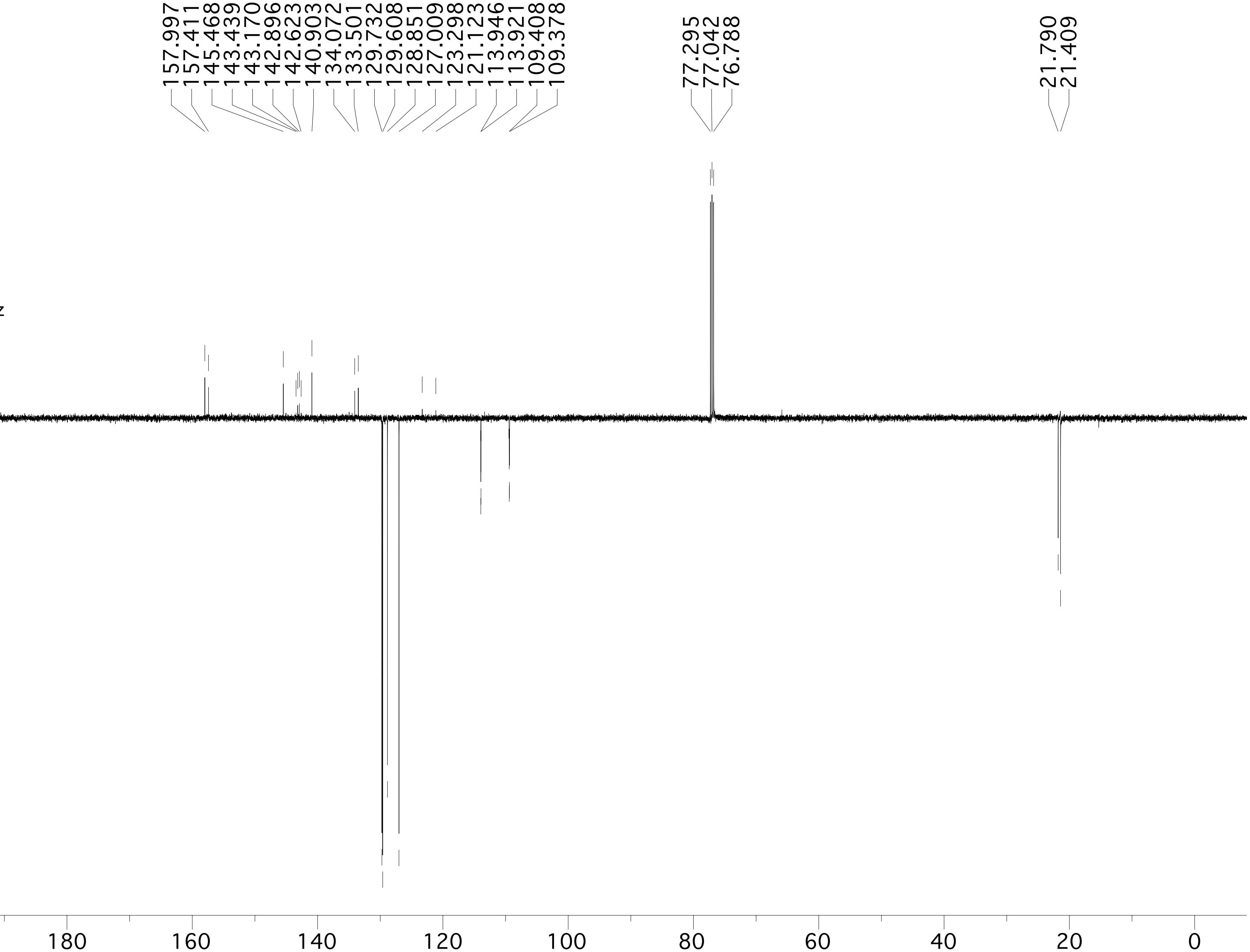

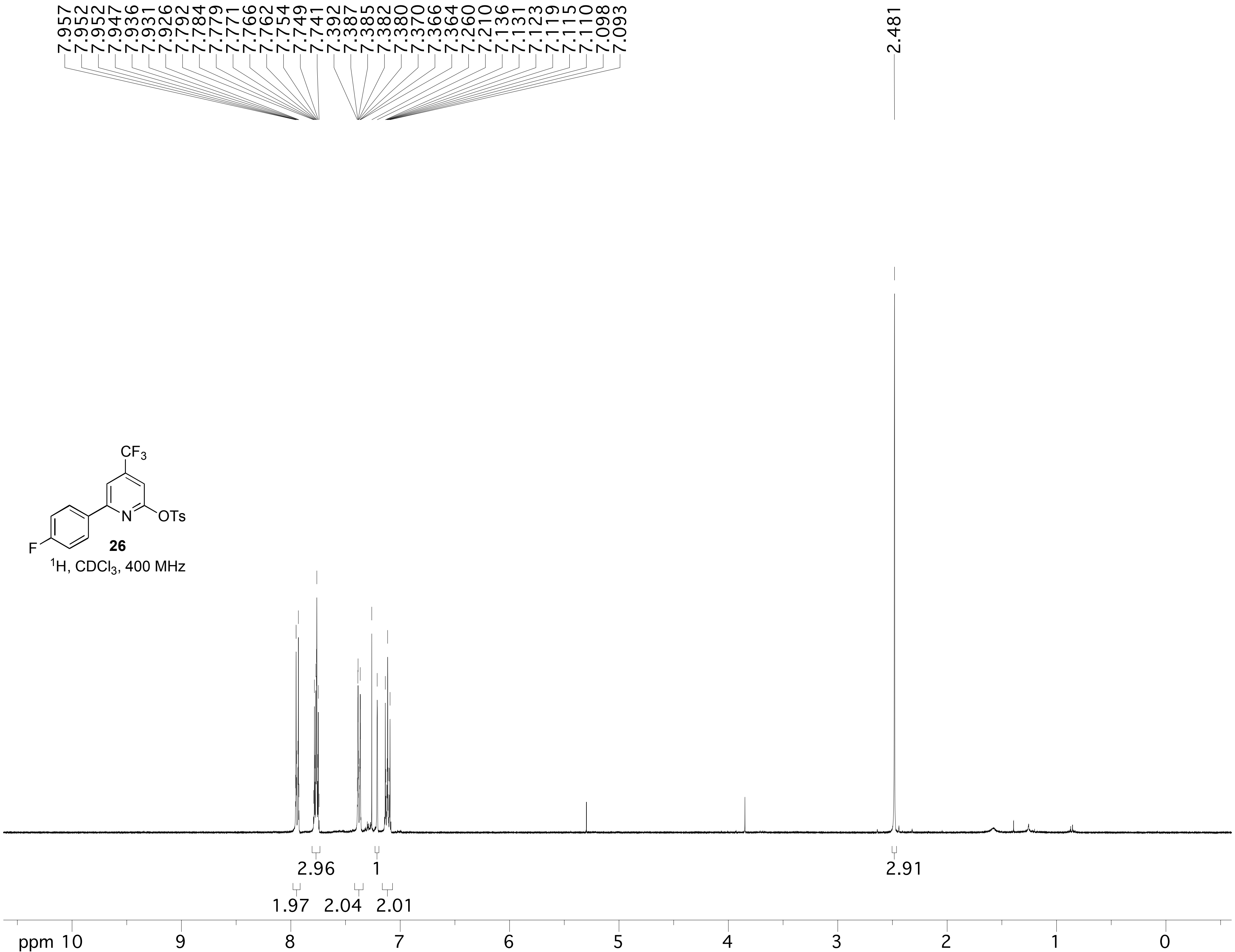

7.957
7.952
7.952
7.947
7.936
7.931
7.926
7.792
7.784
7.779
7.771
7.766
7.762
7.754
7.749
7.741
7.392
7.387
7.385
7.382
7.380
7.370
7.366
7.364
7.260
7.210
7.136
7.131
7.123
7.119
7.115
7.110
7.098
7.093
2.481
CF3
N
OTs
F
26
1H, CDCl3, 400 MHz
2.96
1
2.91
1.97
2.04
2.01
ppm 10
9
8
7
6
5
4
3
2
1
0

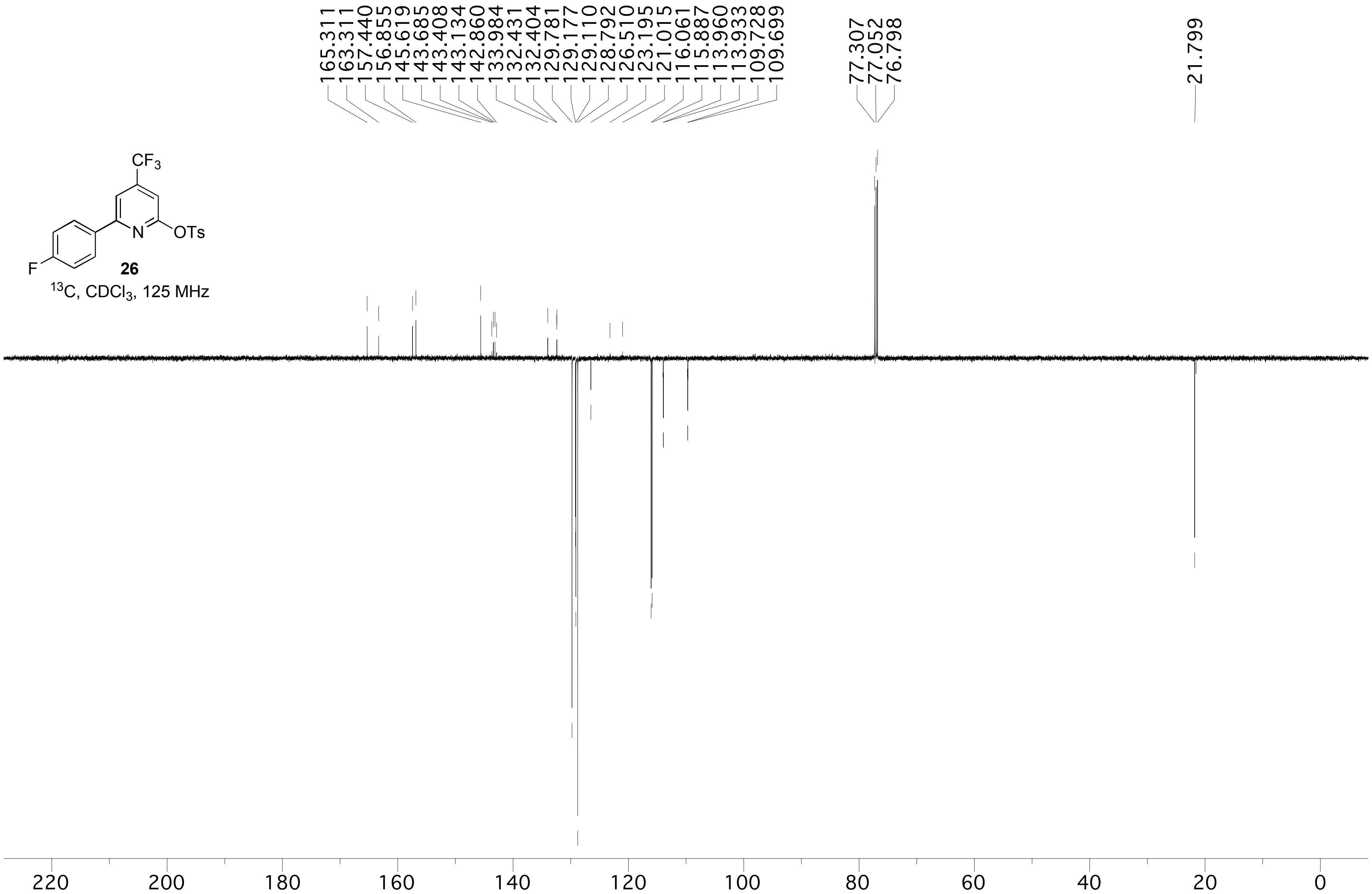
165.311
163.311
157.440
156.855
145.619
143.685
143.408
143.134
142.860
133.984
132.431
132.404
129.781
129.177
129.110
128.792
126.510
123.195
121.015
116.061
115.887
113.960
113.933
109.728
109.699
77.307
77.052
76.798
21.799
CF3
N
OTs
F
26
13C, CDCl3, 125 MHz
220
200
180
160
140
120
100
80
60
40
20
0

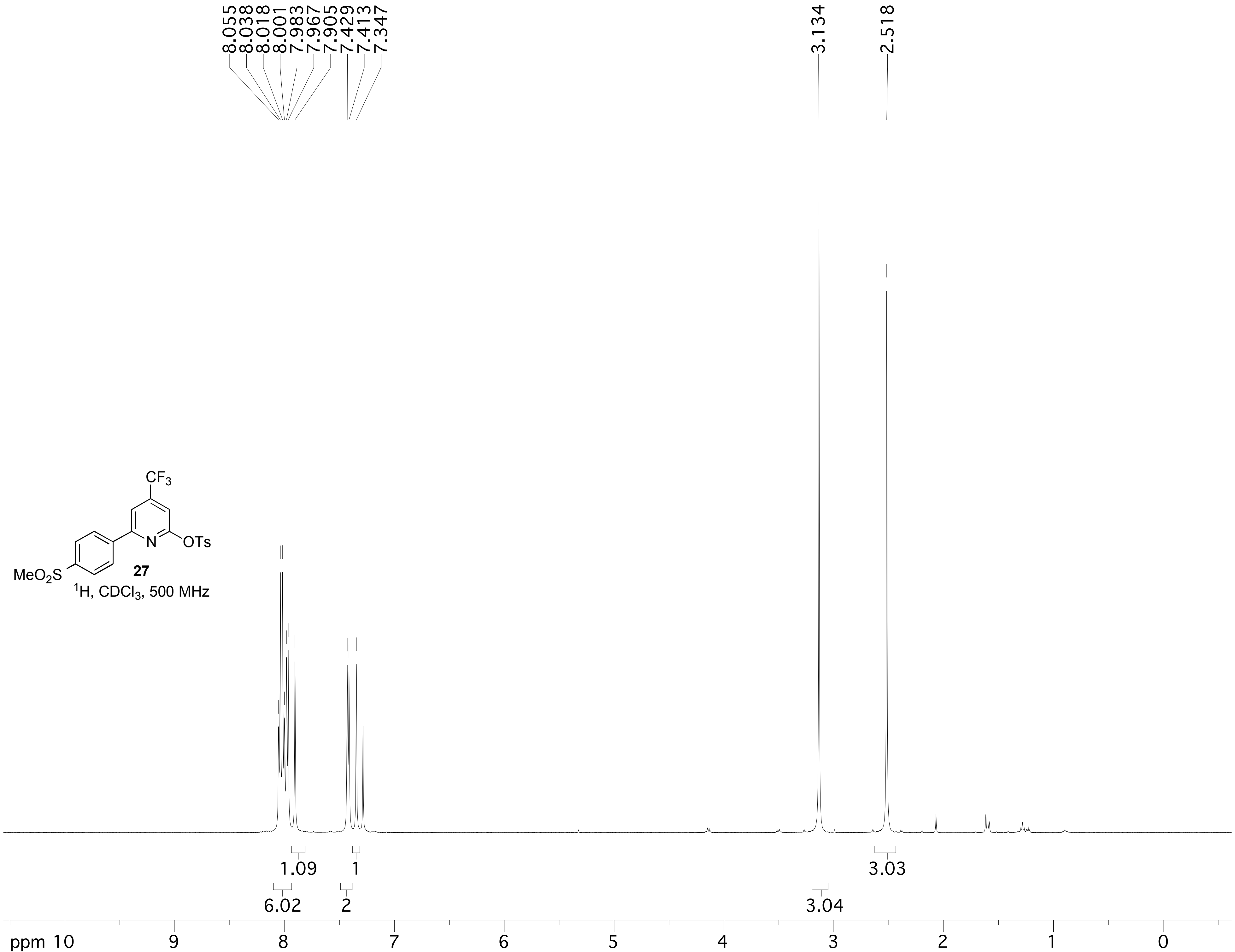

8.055
8.038
8.018
8.001
7.983
7.967
7.905
7.429
7.413
7.347
3.134
2.518
CF3
N
OTs
MeO2S
27
1H, CDCl3, 500 MHz
1.09
1
3.03
6.02
2
3.04
ppm 10
9
8
7
6
5
4
3
2
1
0

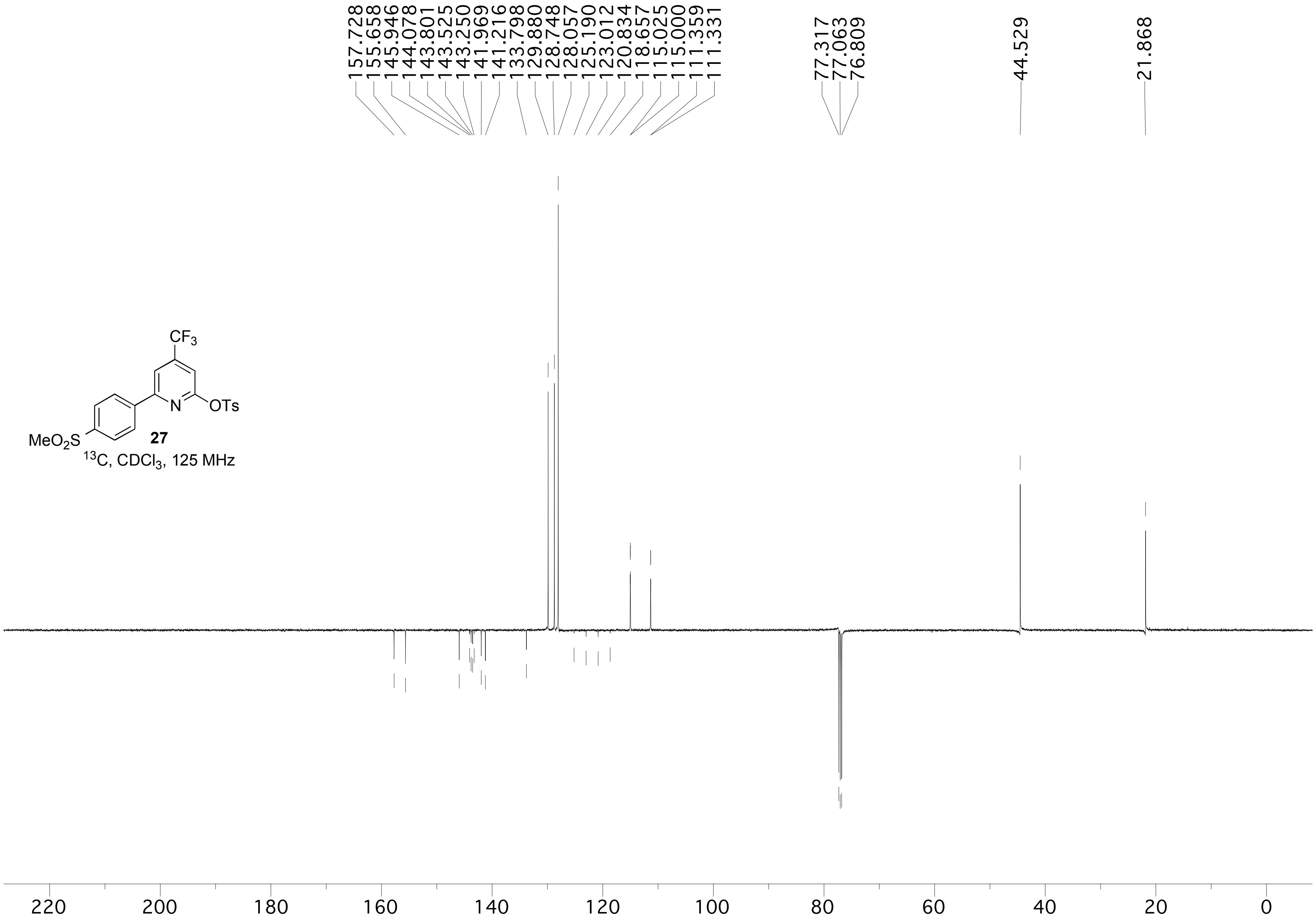
157.728
155.658
145.946
144.078
143.801
143.525
143.250
141.969
141.216
133.798
129.880
128.748
128.057
125.190
123.012
120.834
118.657
115.025
115.000
111.359
111.331
77.317
77.063
76.809
44.529
21.868
CF3
N
OTs
MeO2S
27
13C, CDCl3, 125 MHz
220 200 180 160 140 120 100 80 60 40 20 0

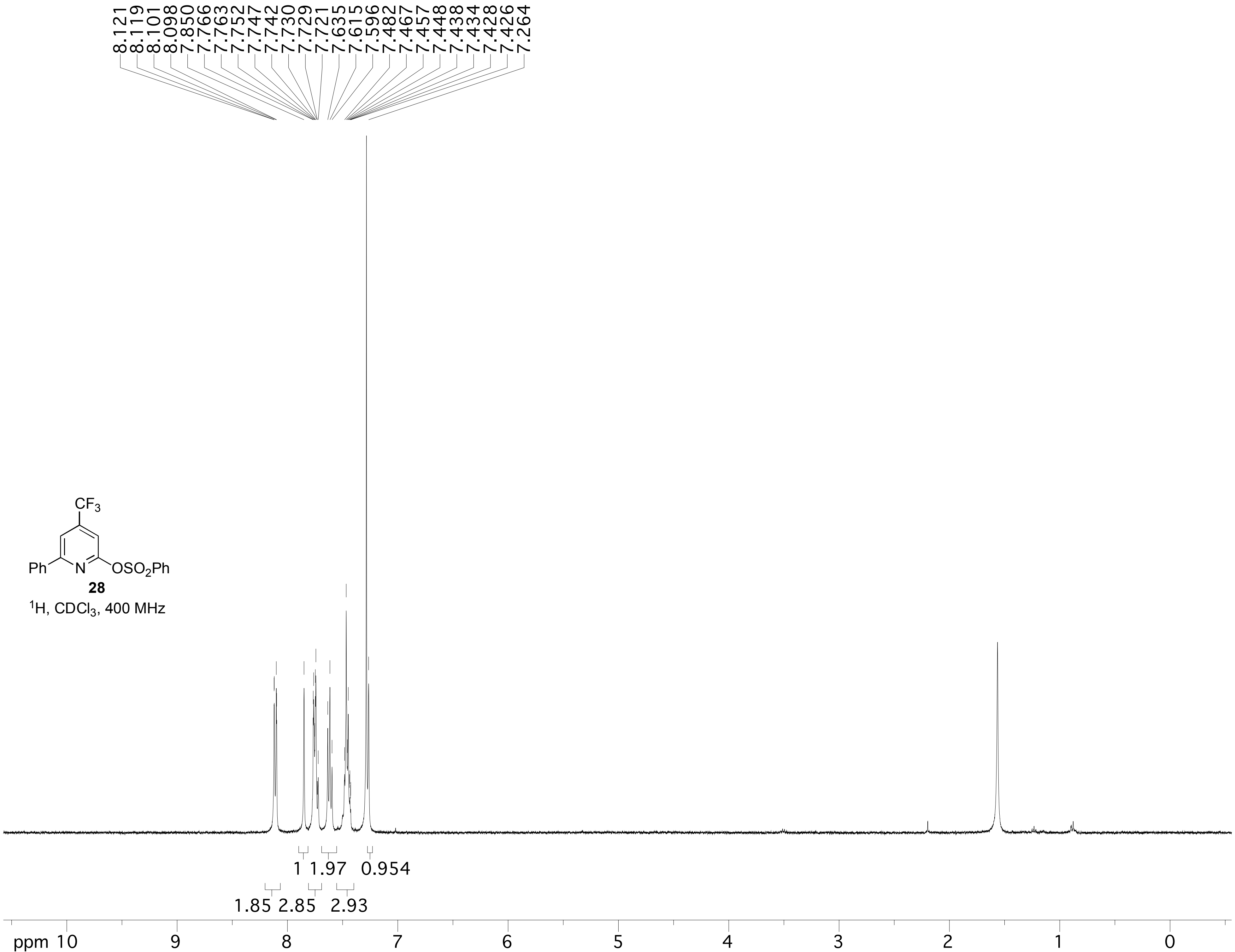
8.121
8.119
8.101
8.098
7.850
7.766
7.763
7.752
7.747
7.742
7.730
7.729
7.721
7.635
7.615
7.596
7.482
7.467
7.457
7.448
7.438
7.434
7.428
7.426
7.264
CF3
Ph
N
OSO2Ph
28
1H, CDCl3, 400 MHz
1
1.97
0.954
1.85
2.85
2.93
ppm 10
9
8
7
6
5
4
3
2
1
0

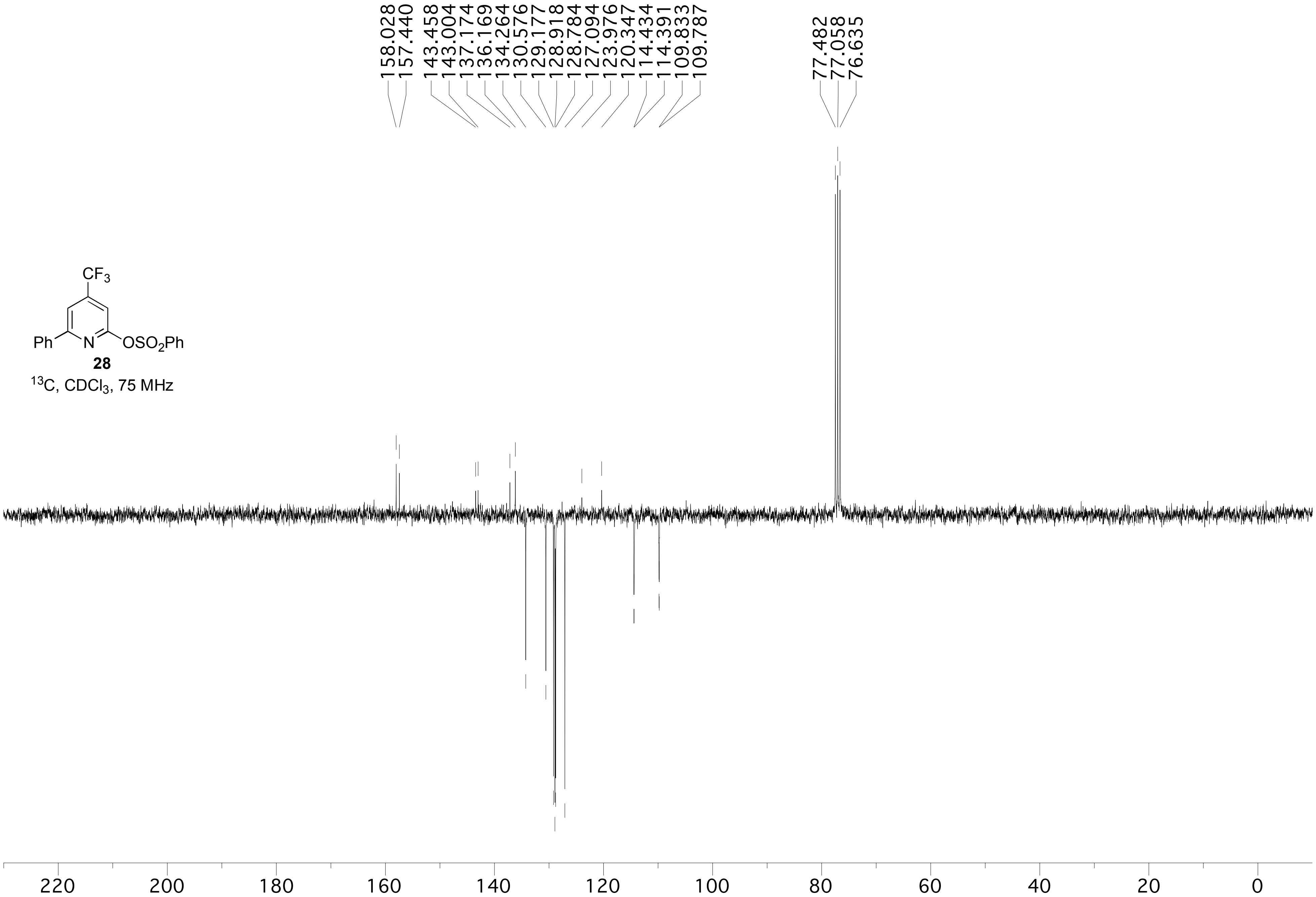

28

^{13}C, $CDCl_3$, 75 MHz

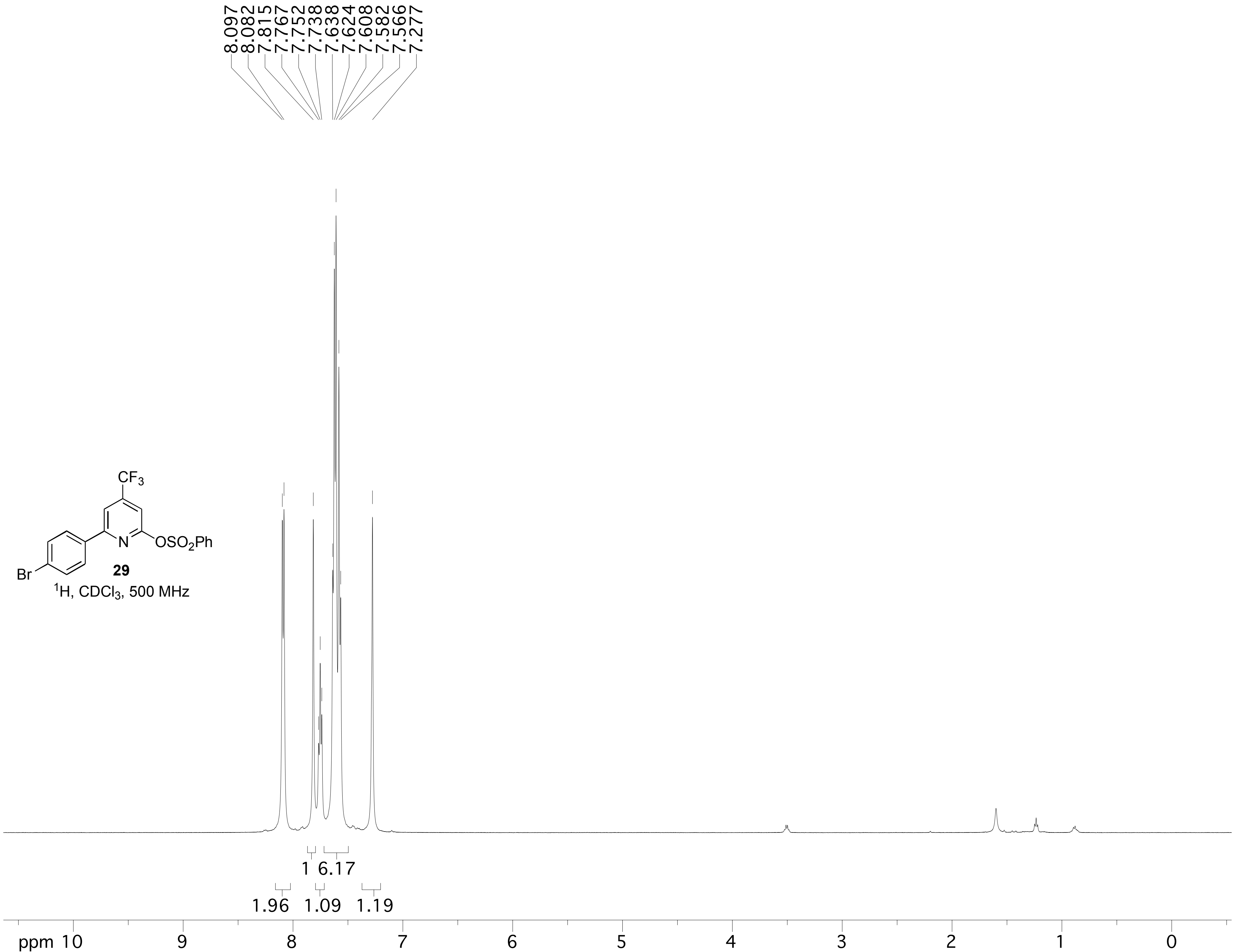
8.097
8.082
7.815
7.767
7.752
7.738
7.638
7.624
7.608
7.582
7.566
7.277
CF3
N
OSO2Ph
Br
29
1H, CDCl3, 500 MHz
1
6.17
1.96
1.09
1.19
ppm 10
9
8
7
6
5
4
3
2
1
0

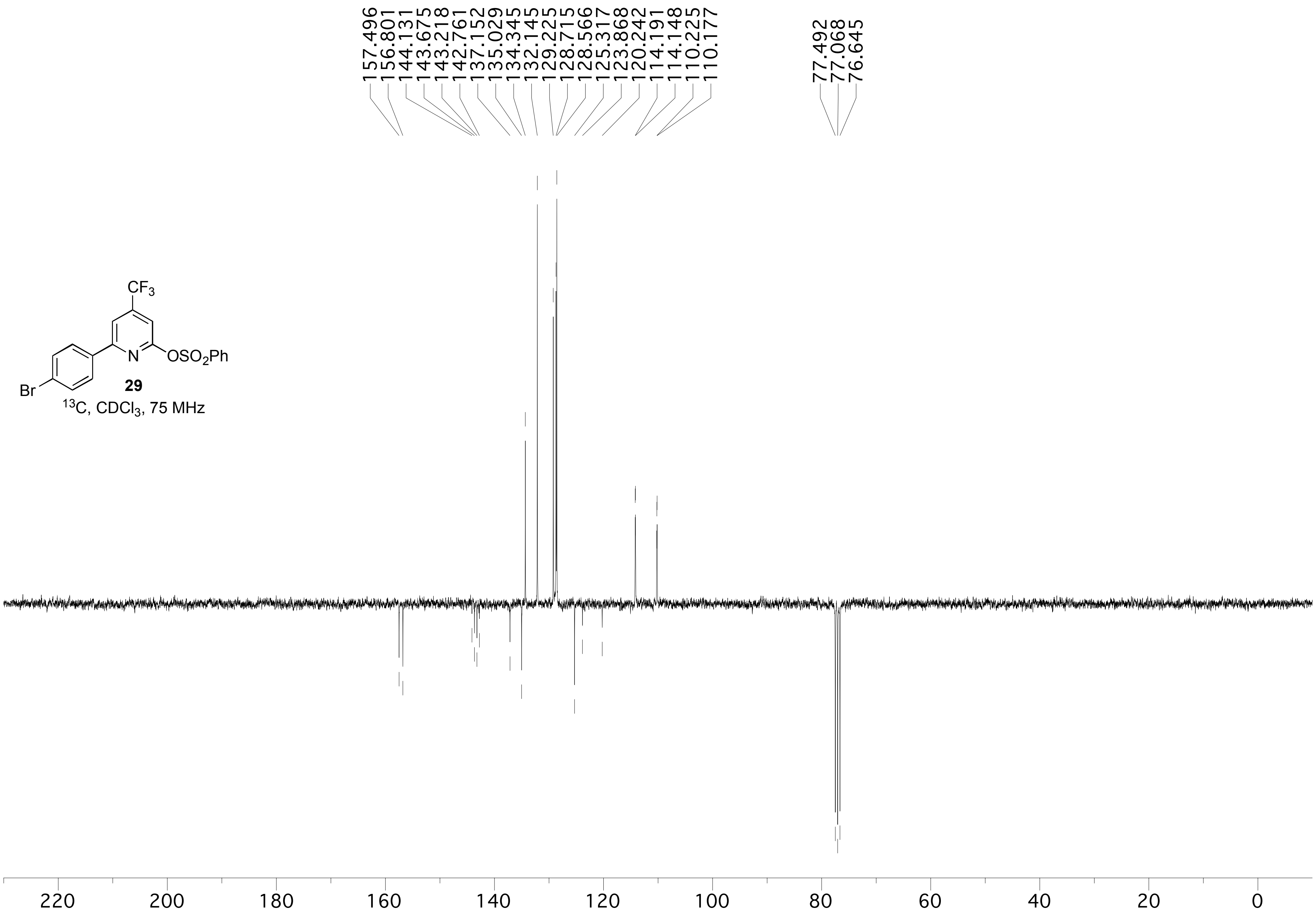
157.496
156.801
144.131
143.675
143.218
142.761
137.152
135.029
134.345
132.145
129.225
128.715
128.566
125.317
123.868
120.242
114.191
114.148
110.225
110.177
77.492
77.068
76.645
CF3
N
OSO2Ph
Br
29
13C, CDCl3, 75 MHz
220
200
180
160
140
120
100
80
60
40
20
0

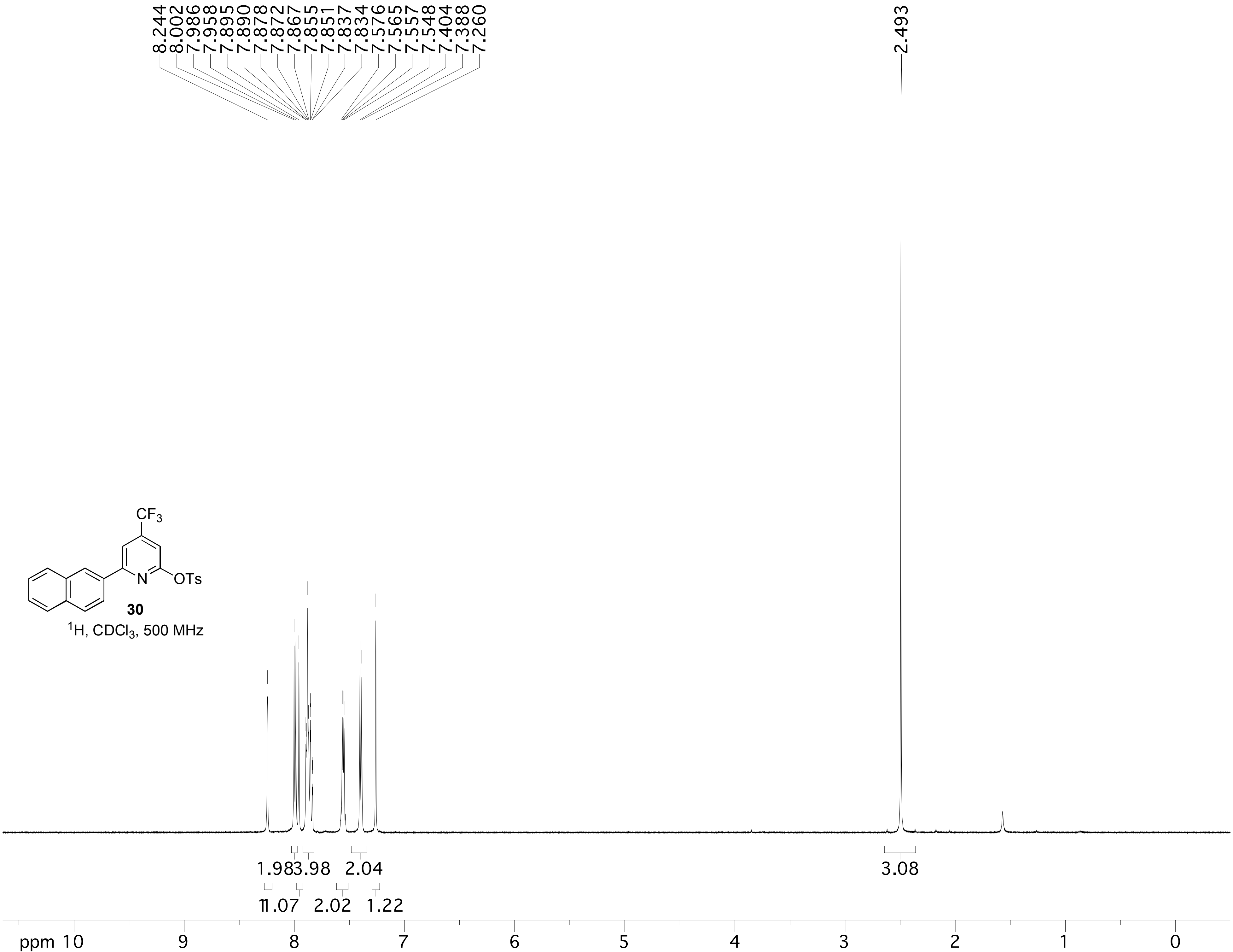
8.244
8.002
7.986
7.958
7.895
7.890
7.878
7.872
7.867
7.855
7.851
7.837
7.834
7.576
7.565
7.557
7.548
7.404
7.388
7.260
2.493
CF3
N
OTs
30
1H, CDCl3, 500 MHz
1.98
3.98
2.04
3.08
1.07
2.02
1.22
ppm 10
9
8
7
6
5
4
3
2
1
0

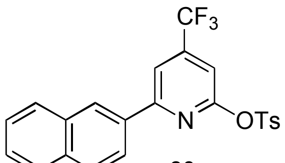

30

^{13}C, CDCl$_3$, 125 MHz

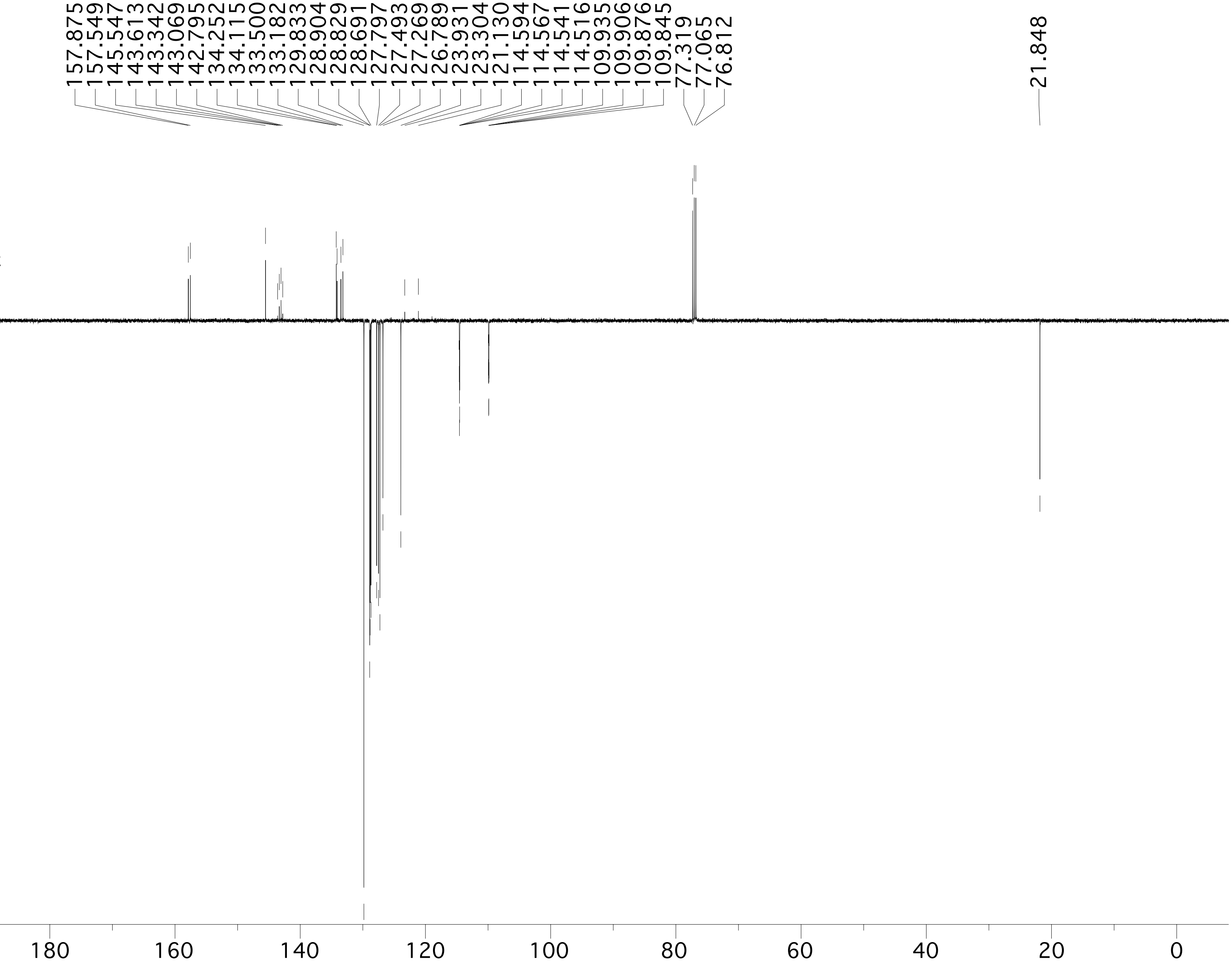

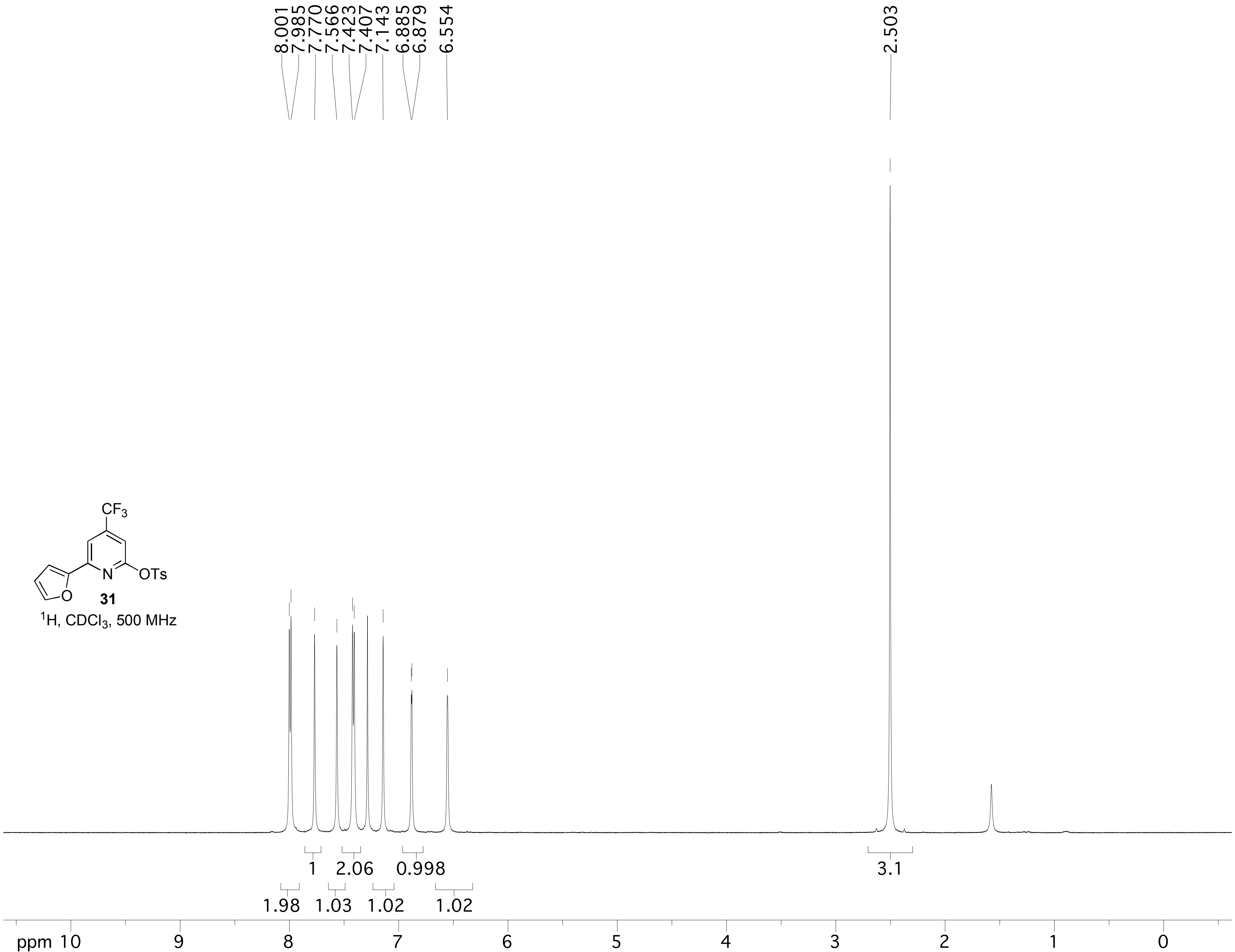

8.001
7.985
7.770
7.566
7.423
7.407
7.143
6.885
6.879
6.554
2.503
CF3
N
OTs
O
31
1H, CDCl3, 500 MHz
1
2.06
0.998
3.1
1.98
1.03
1.02
1.02
ppm 10
9
8
7
6
5
4
3
2
1
0

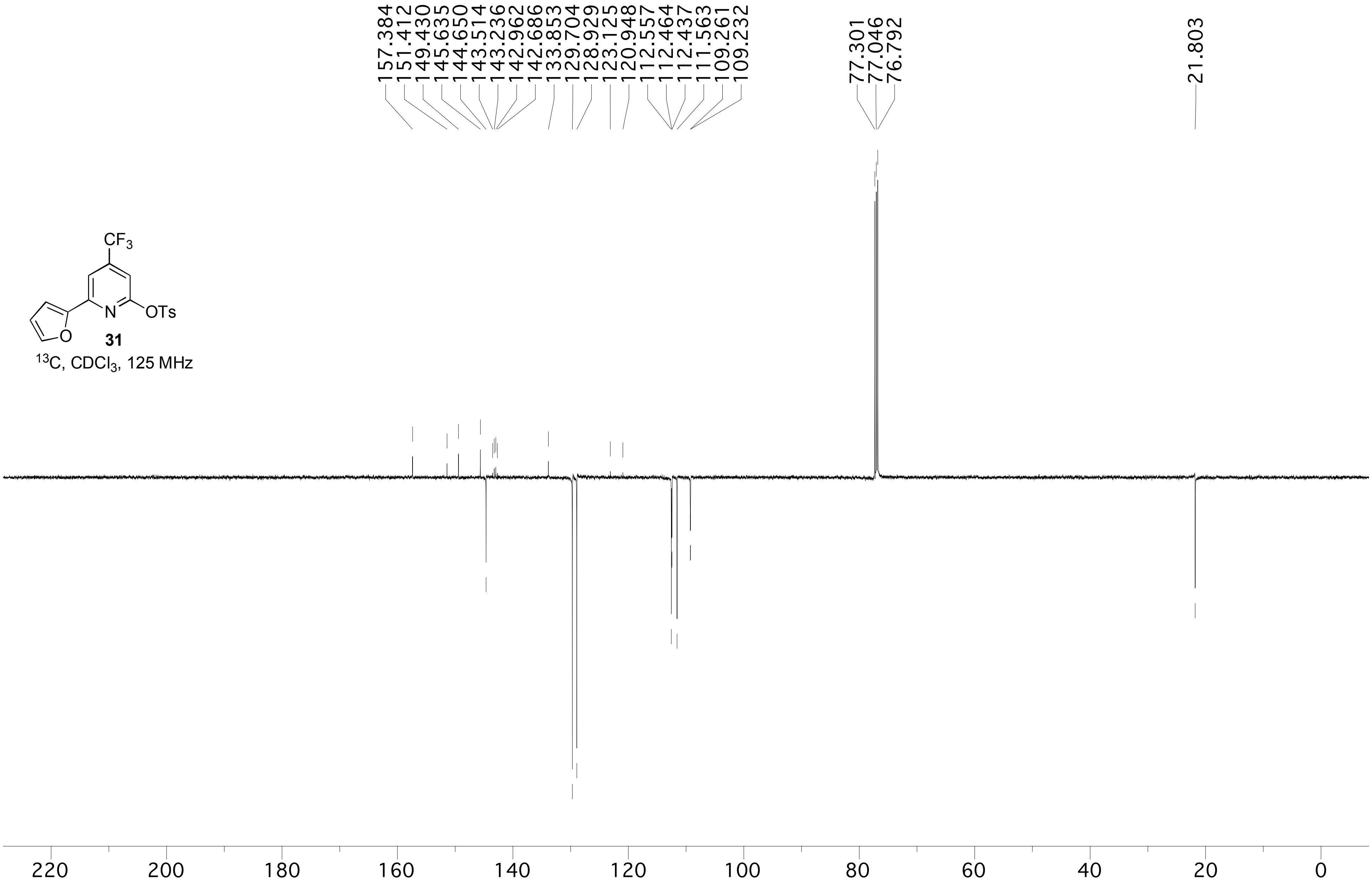

31

^{13}C, $CDCl_3$, 125 MHz

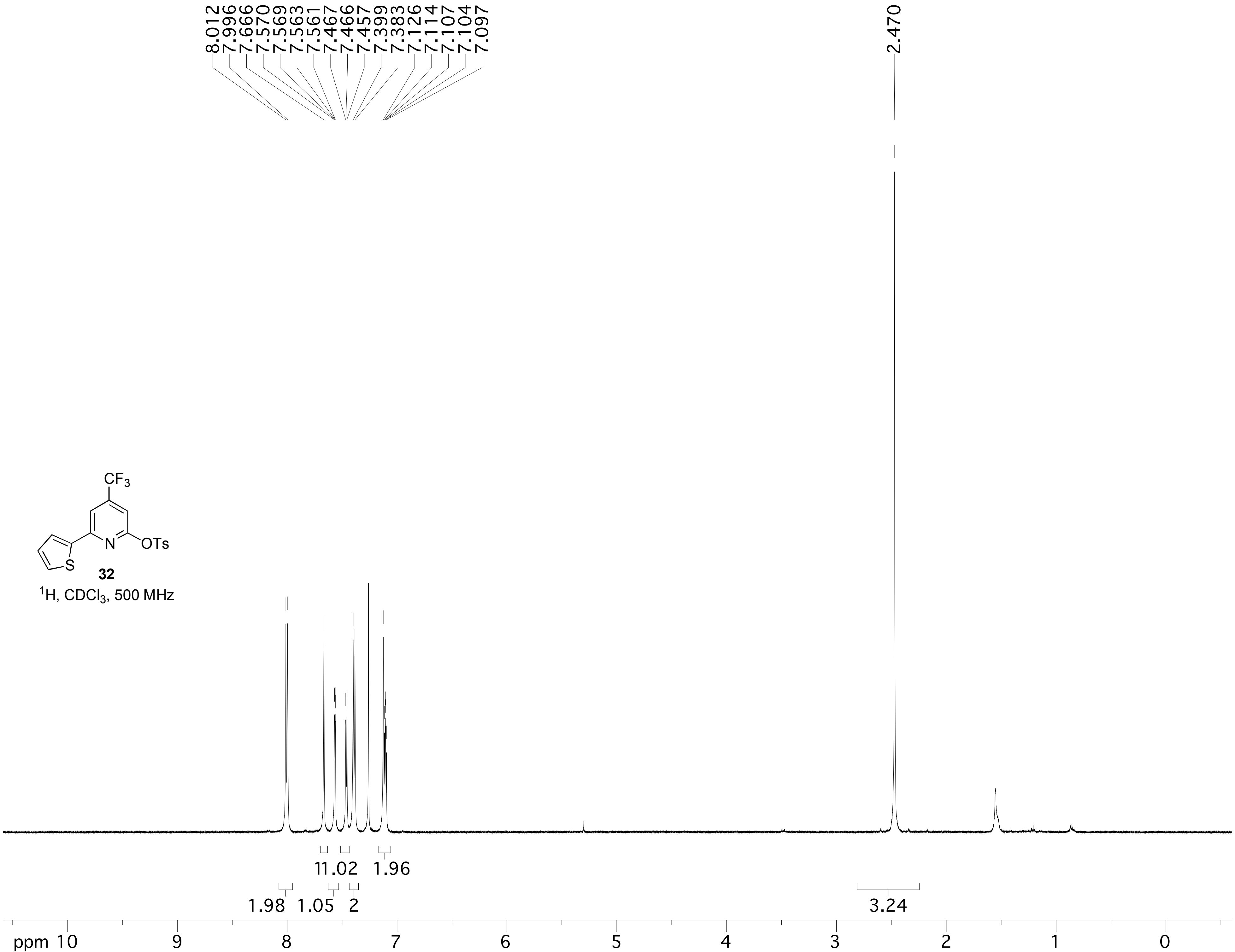

8.012
7.996
7.666
7.570
7.569
7.563
7.561
7.467
7.466
7.457
7.399
7.383
7.126
7.114
7.107
7.104
7.097
2.470
CF3
N
OTs
S
32
1H, CDCl3, 500 MHz
1.98
1.05
11.02
2
1.96
3.24
ppm 10
9
8
7
6
5
4
3
2
1
0

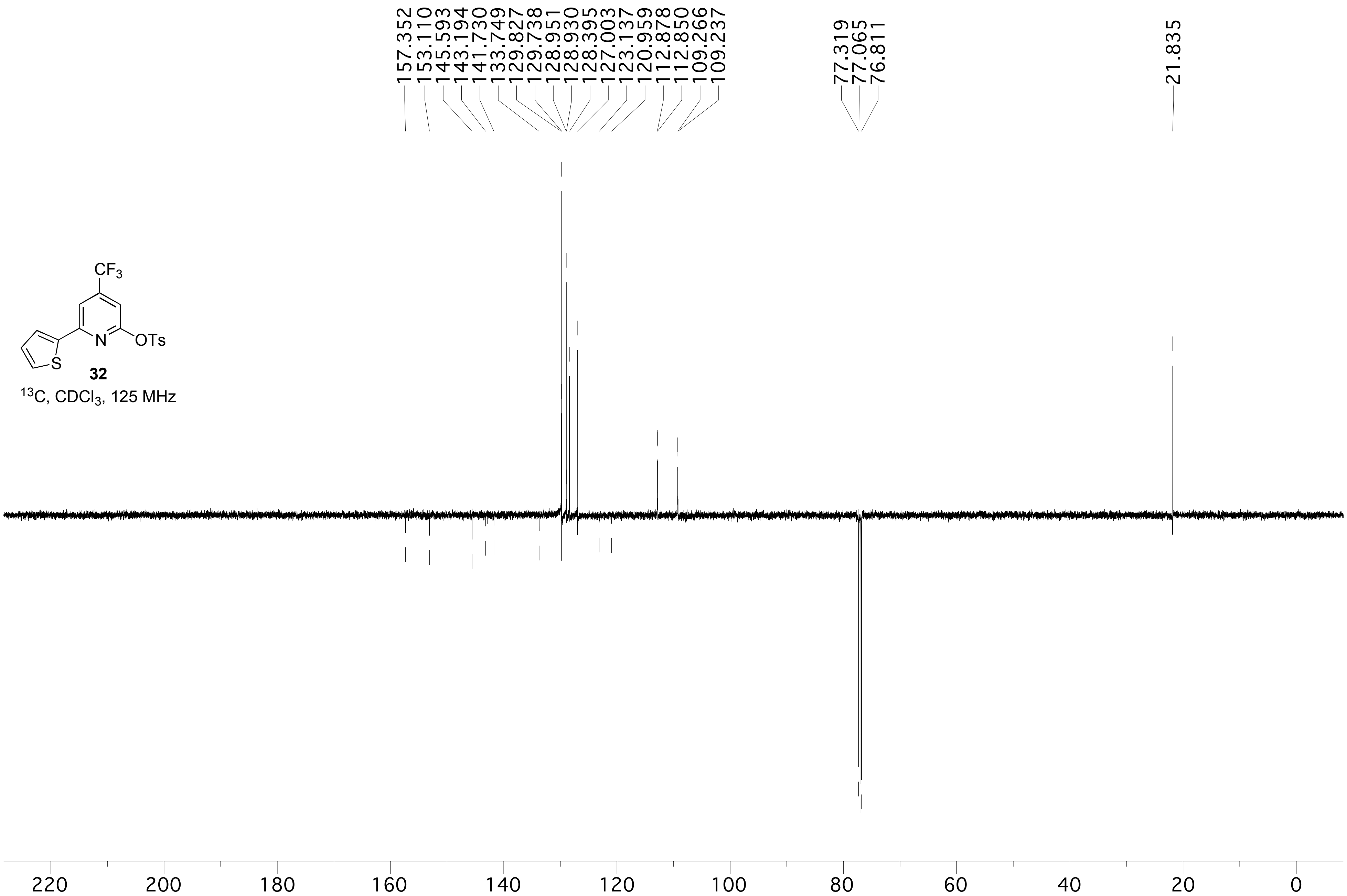
157.352
153.110
145.593
143.194
141.730
133.749
129.827
129.738
128.951
128.930
128.395
127.003
123.137
120.959
112.878
112.850
109.266
109.237
77.319
77.065
76.811
21.835
CF3
N
OTs
S
32
13C, CDCl3, 125 MHz
220
200
180
160
140
120
100
80
60
40
20
0

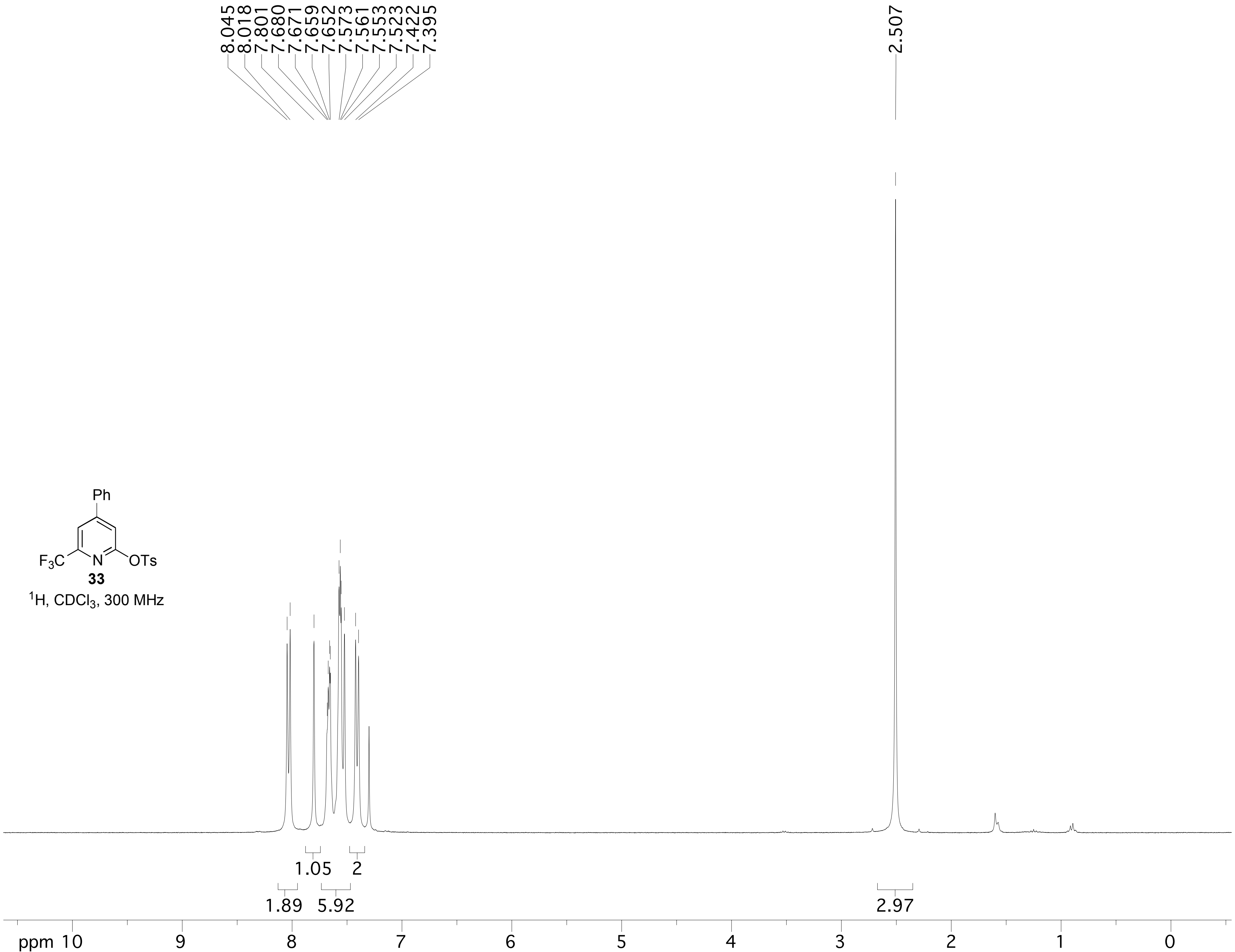

8.045
8.018
7.801
7.680
7.671
7.659
7.652
7.573
7.561
7.553
7.523
7.422
7.395
2.507
Ph
F3C
N
OTs
33
1H, CDCl3, 300 MHz
1.05
2
1.89
5.92
2.97
ppm 10
9
8
7
6
5
4
3
2
1
0

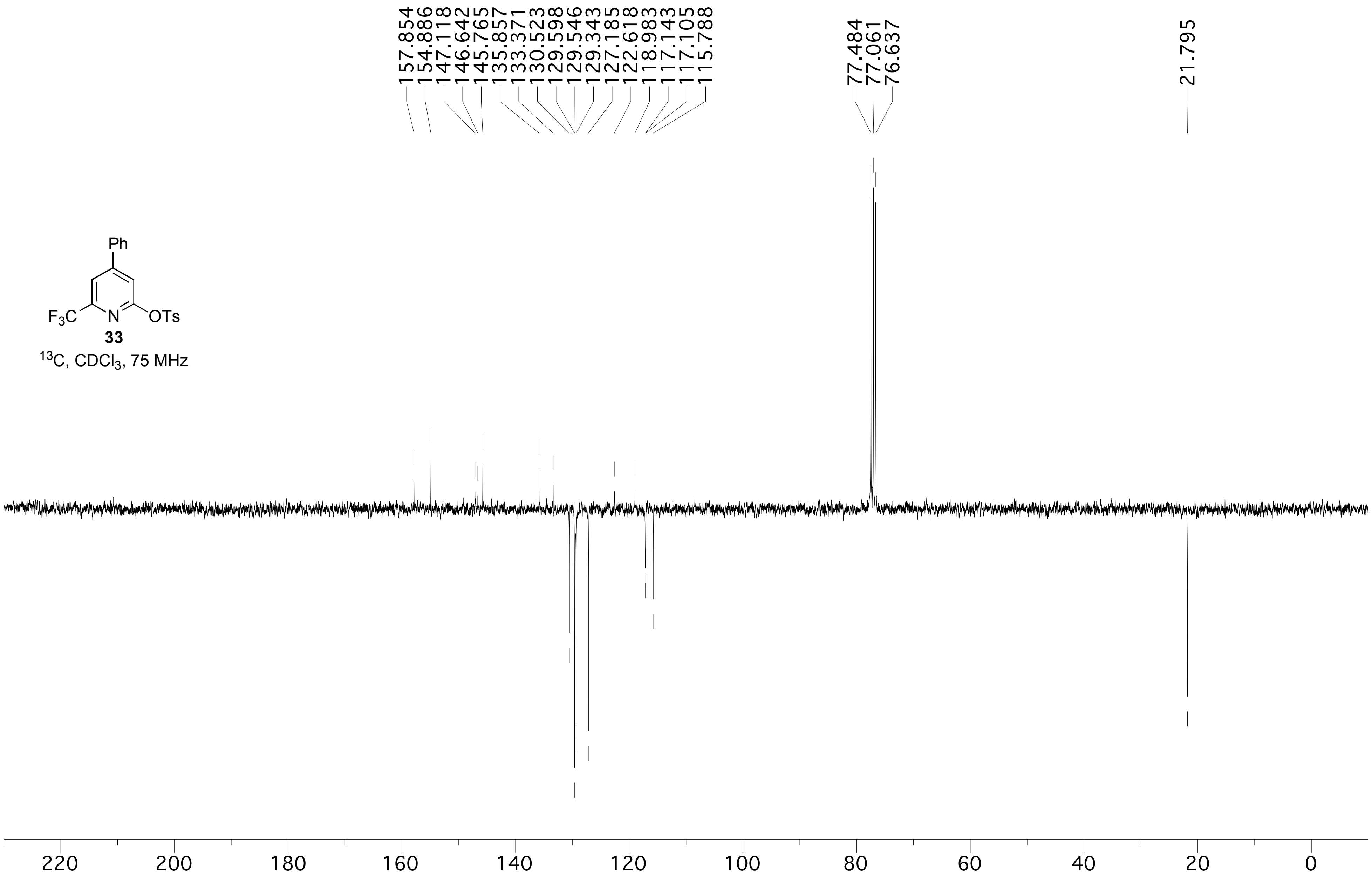

157.854
154.886
147.118
146.642
145.765
135.857
133.371
130.523
129.598
129.546
129.343
127.185
122.618
118.983
117.143
117.105
115.788
77.484
77.061
76.637
21.795
Ph
F3C
N
OTs
33
13C, CDCl3, 75 MHz
220
200
180
160
140
120
100
80
60
40
20
0

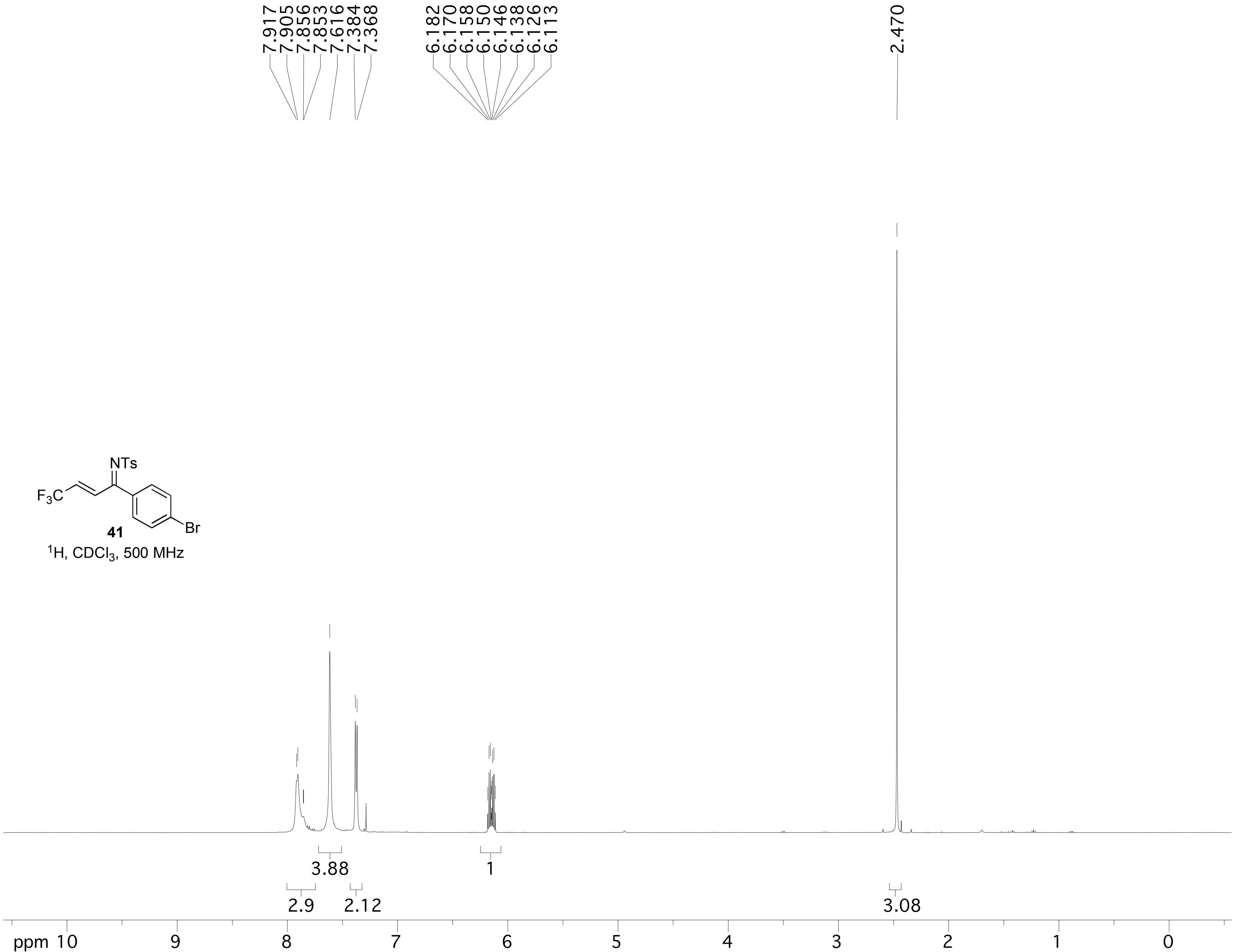
7.917
7.905
7.856
7.853
7.616
7.384
7.368
6.182
6.170
6.158
6.150
6.146
6.138
6.126
6.113
2.470
NTs
F_3C
Br
41
^1H, $CDCl_3$, 500 MHz
3.88
1
2.9
2.12
3.08
ppm 10
9
8
7
6
5
4
3
2
1
0

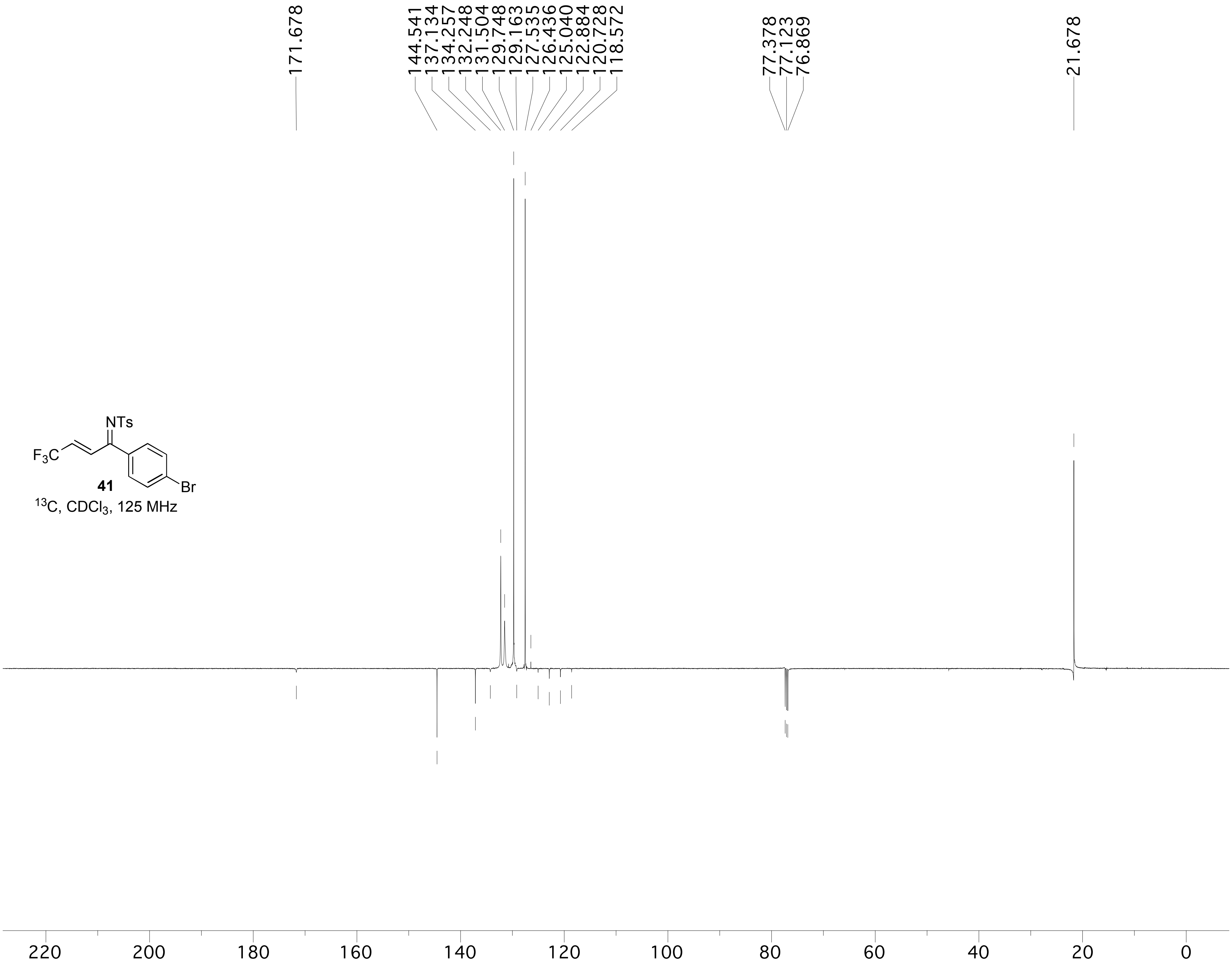

171.678
144.541
137.134
134.257
132.248
131.504
129.748
129.163
127.535
126.436
125.040
122.884
120.728
118.572
77.378
77.123
76.869
21.678
NTs
F3C
41
Br
13C, CDCl3, 125 MHz
220
200
180
160
140
120
100
80
60
40
20
0

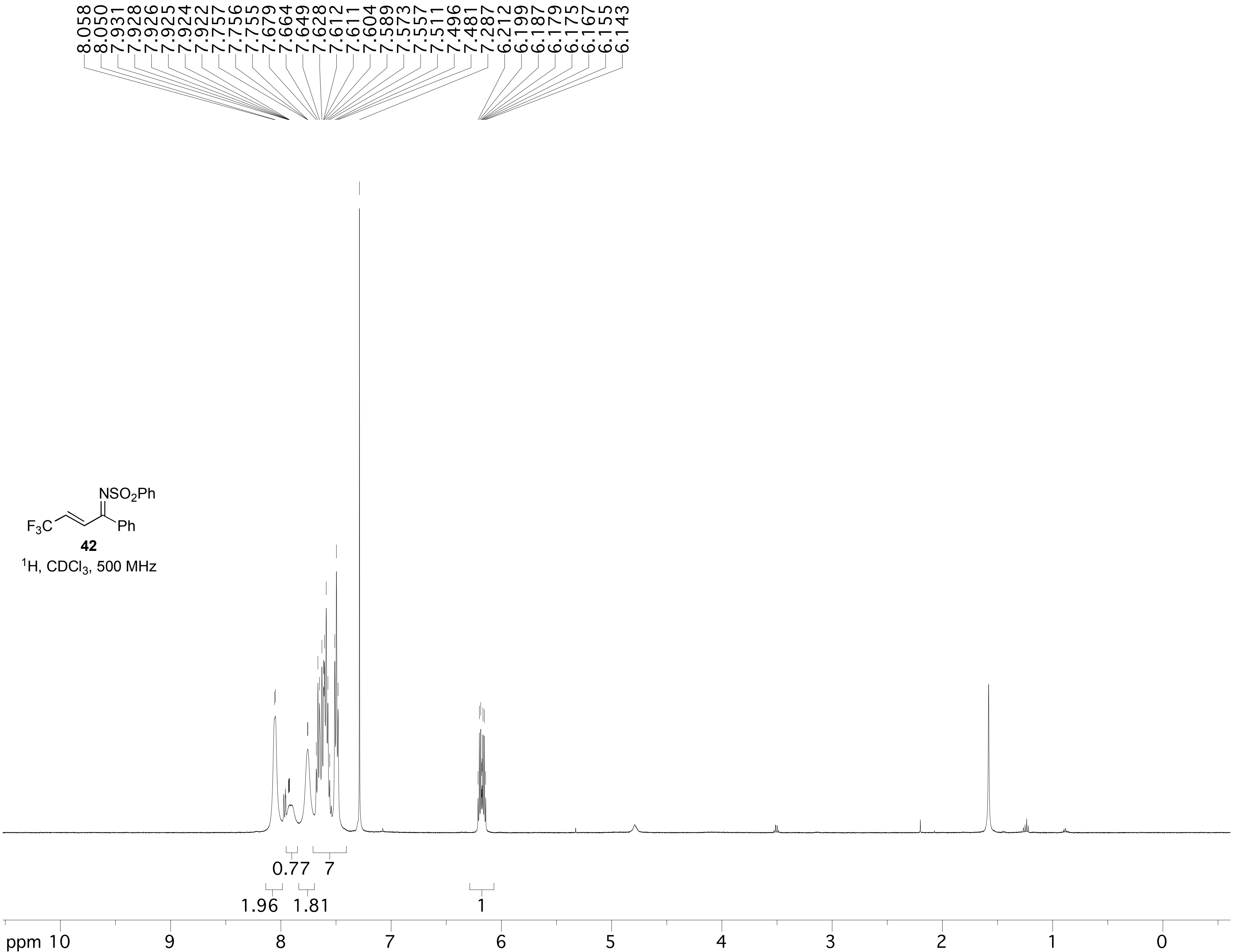
8.058 8.050 7.931 7.928 7.926 7.925 7.924 7.922 7.757 7.756 7.755 7.679 7.664 7.649 7.628 7.612 7.611 7.604 7.589 7.573 7.557 7.511 7.496 7.481 7.287 6.212 6.199 6.187 6.179 6.175 6.167 6.155 6.143
NSO2Ph
F3C
Ph
42
1H, CDCl3, 500 MHz
0.77
7
1.96
1.81
1
ppm 10
9
8
7
6
5
4
3
2
1
0

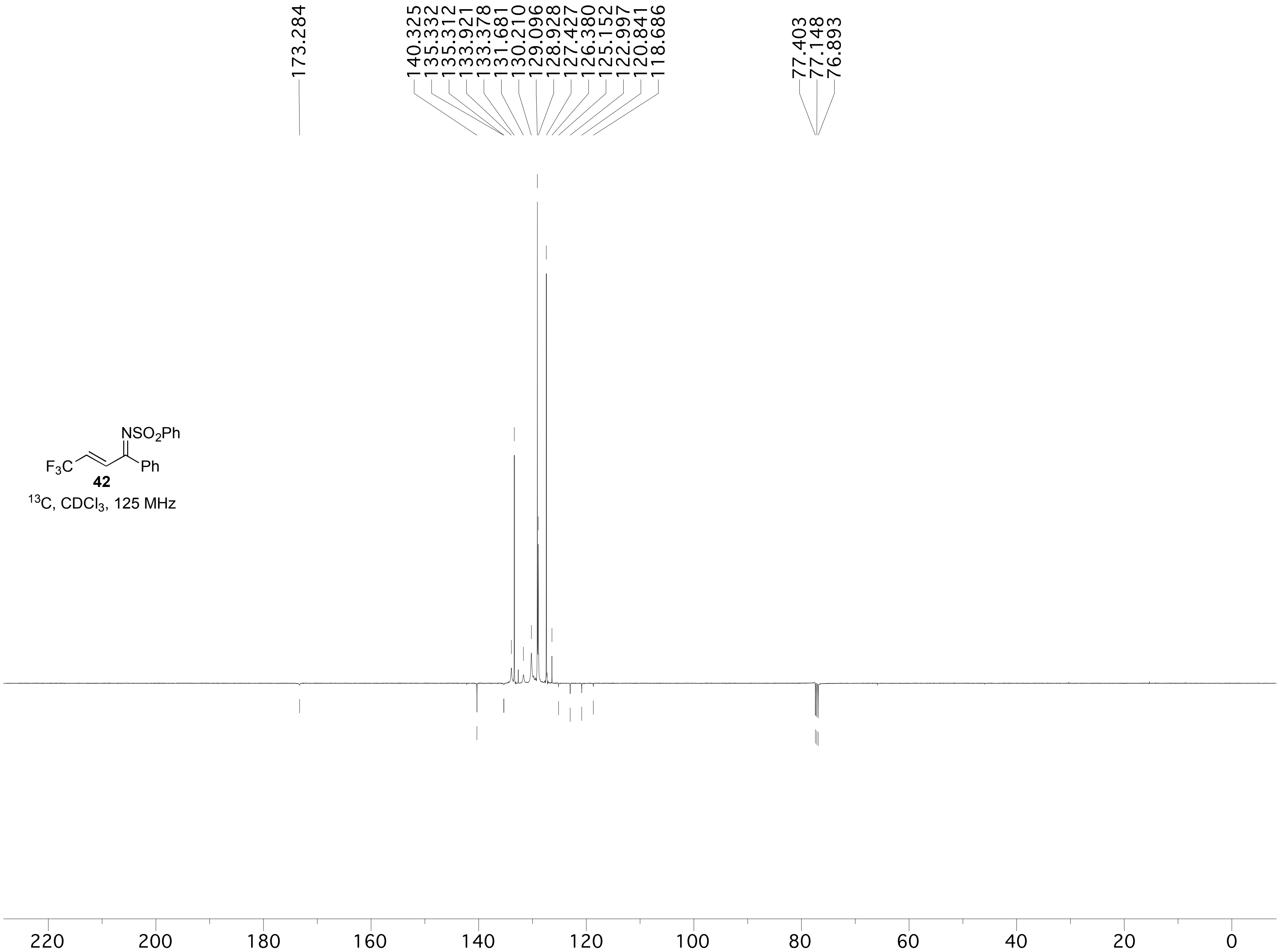
173.284
140.325
135.332
135.312
133.921
133.378
131.681
130.210
129.096
128.928
127.427
126.380
125.152
122.997
120.841
118.686
77.403
77.148
76.893
NSO2Ph
F3C
Ph
42
13C, CDCl3, 125 MHz
220
200
180
160
140
120
100
80
60
40
20
0

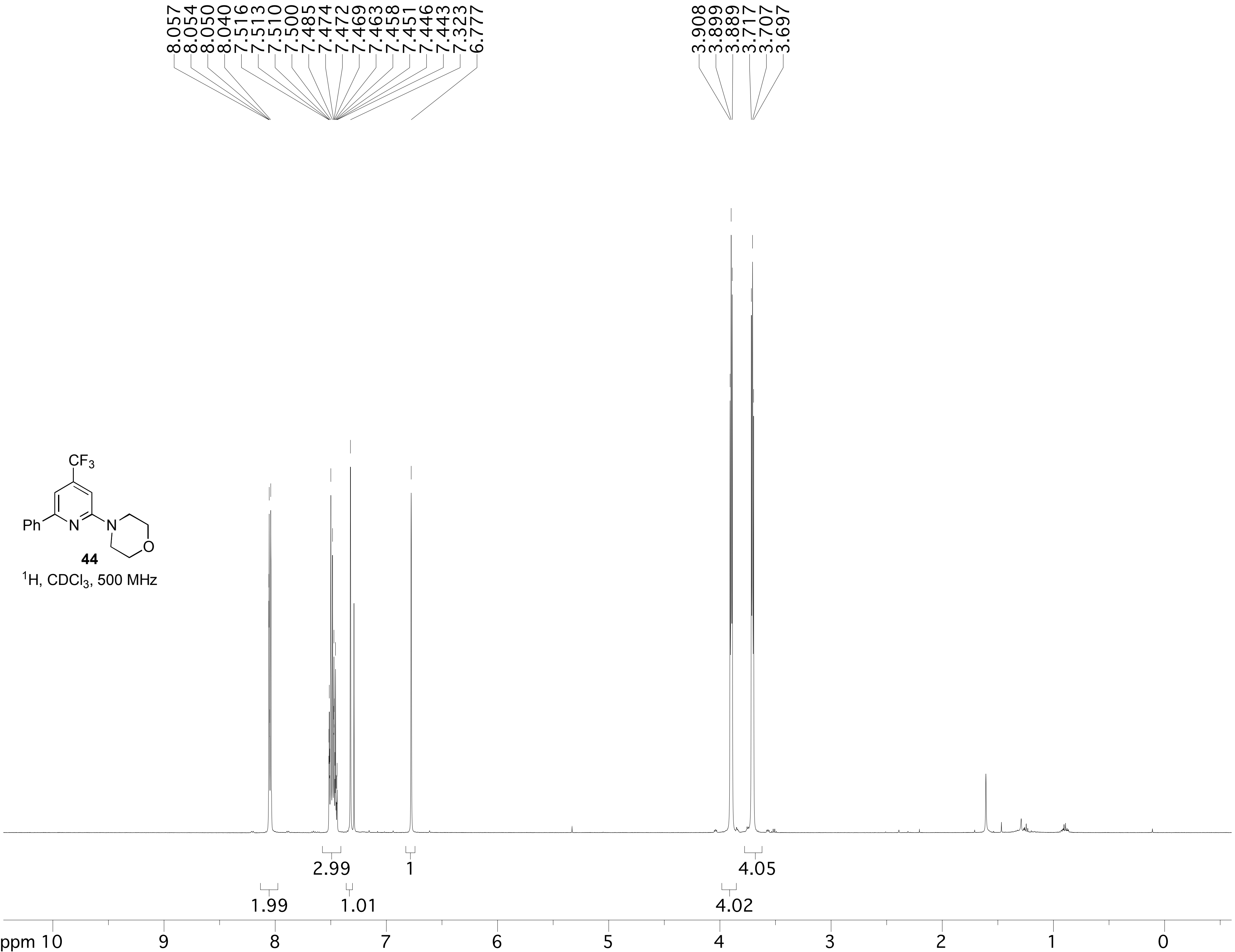

44

^{1}H, $CDCl_3$, 500 MHz

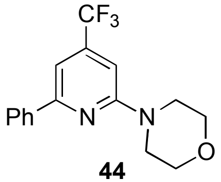

44

^{13}C, $CDCl_3$, 100 MHz

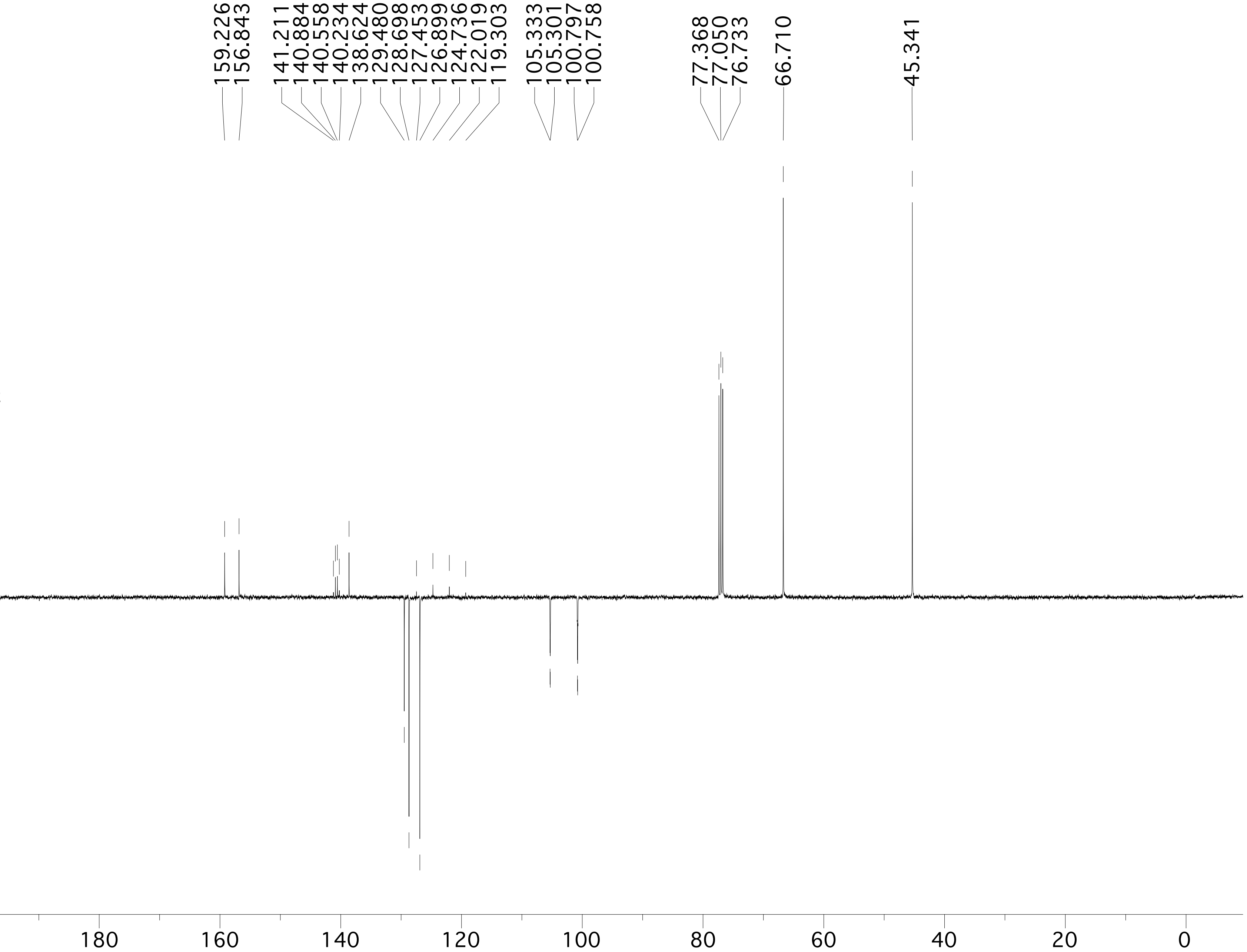

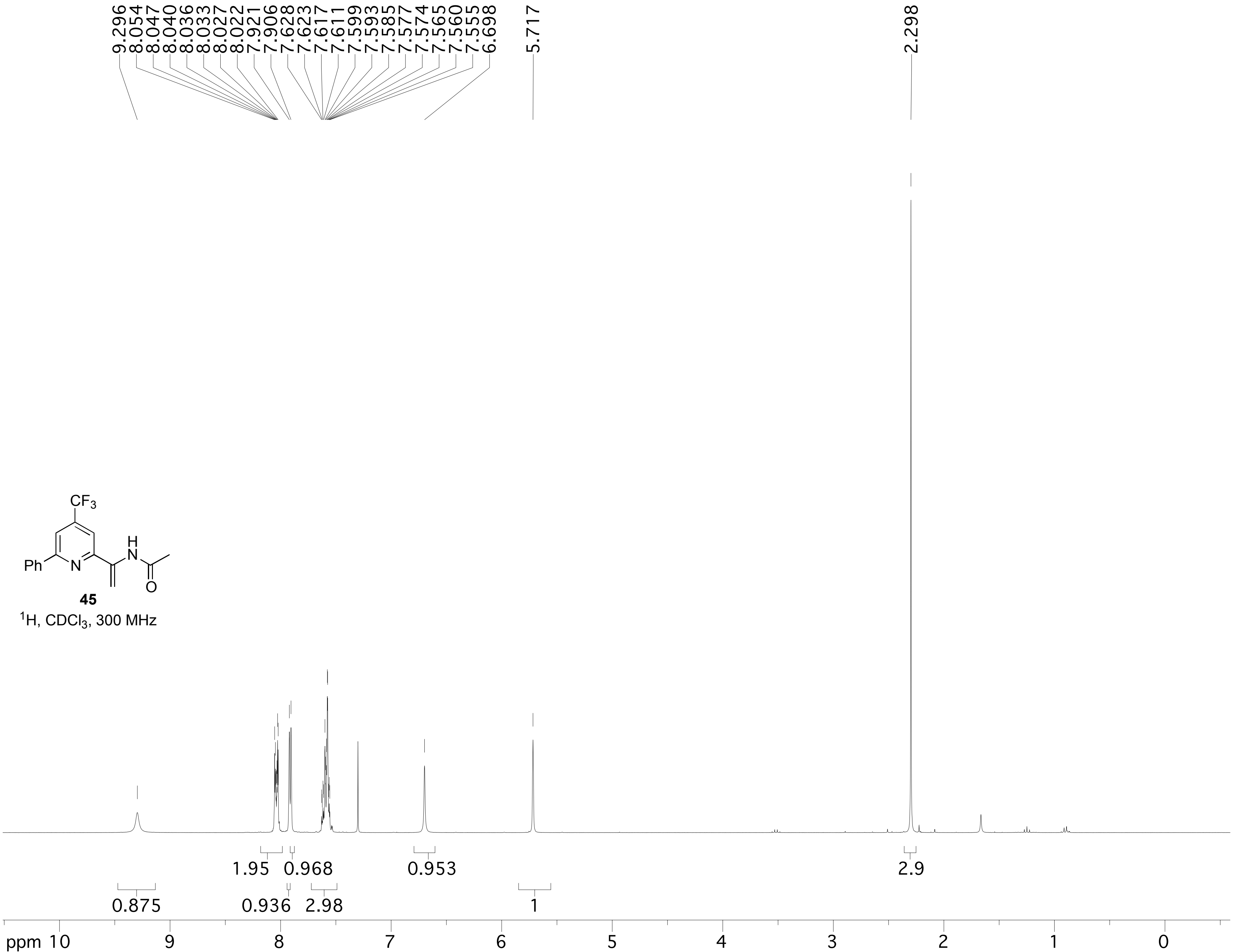
9.296
8.054
8.047
8.040
8.036
8.033
8.027
8.022
7.921
7.906
7.628
7.623
7.617
7.611
7.599
7.593
7.585
7.577
7.574
7.565
7.560
7.555
6.698
5.717
2.298
CF3
H
N
Ph
N
O
45
1H, CDCl3, 300 MHz
1.95
0.968
0.953
2.9
0.875
0.936
2.98
1
ppm 10
9
8
7
6
5
4
3
2
1
0

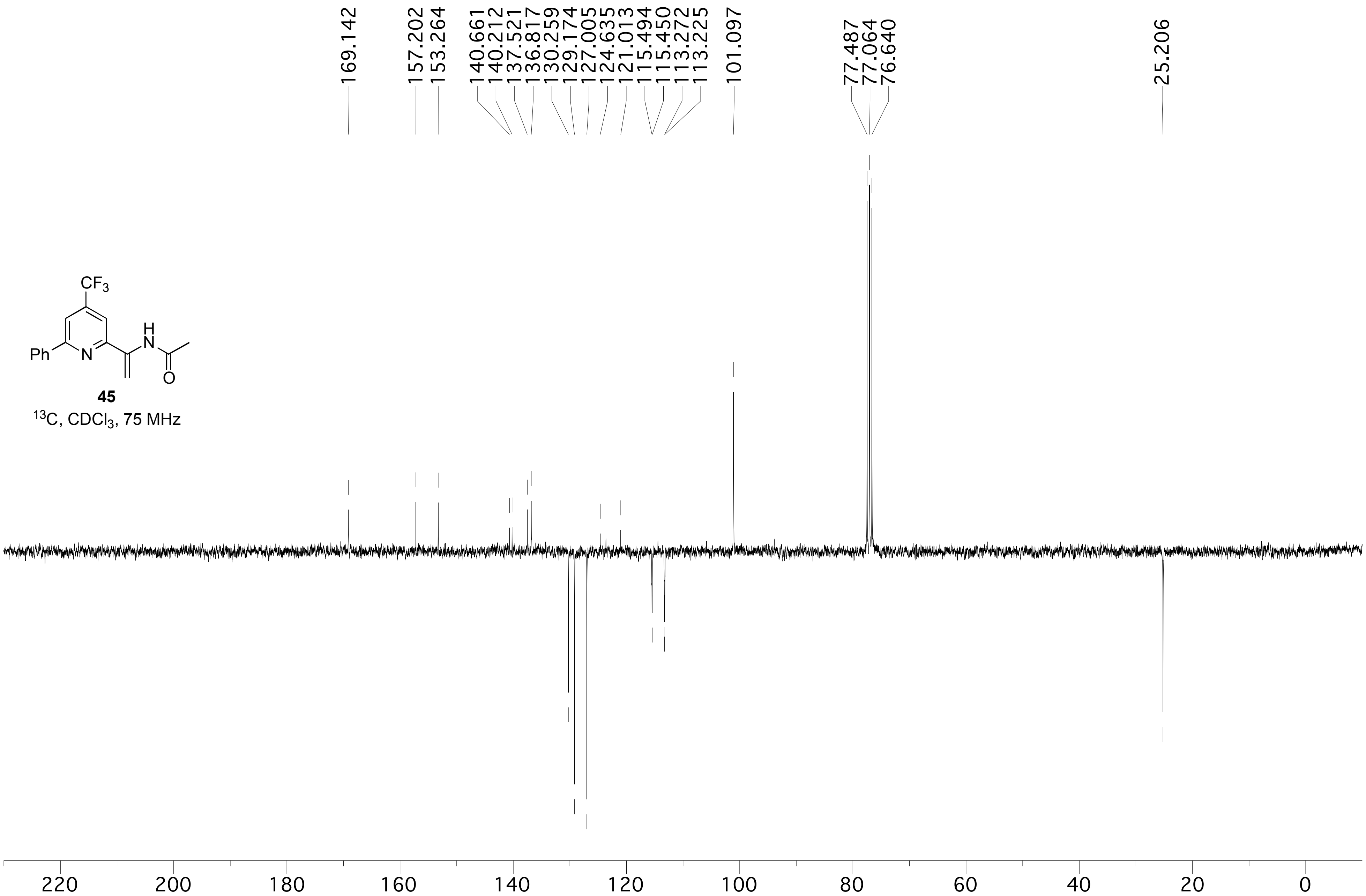
169.142
157.202
153.264
140.661
140.212
137.521
136.817
130.259
129.174
127.005
124.635
121.013
115.494
115.450
113.272
113.225
101.097
77.487
77.064
76.640
25.206
CF3
H
N
Ph
N
O
45
13C, CDCl3, 75 MHz
220
200
180
160
140
120
100
80
60
40
20
0

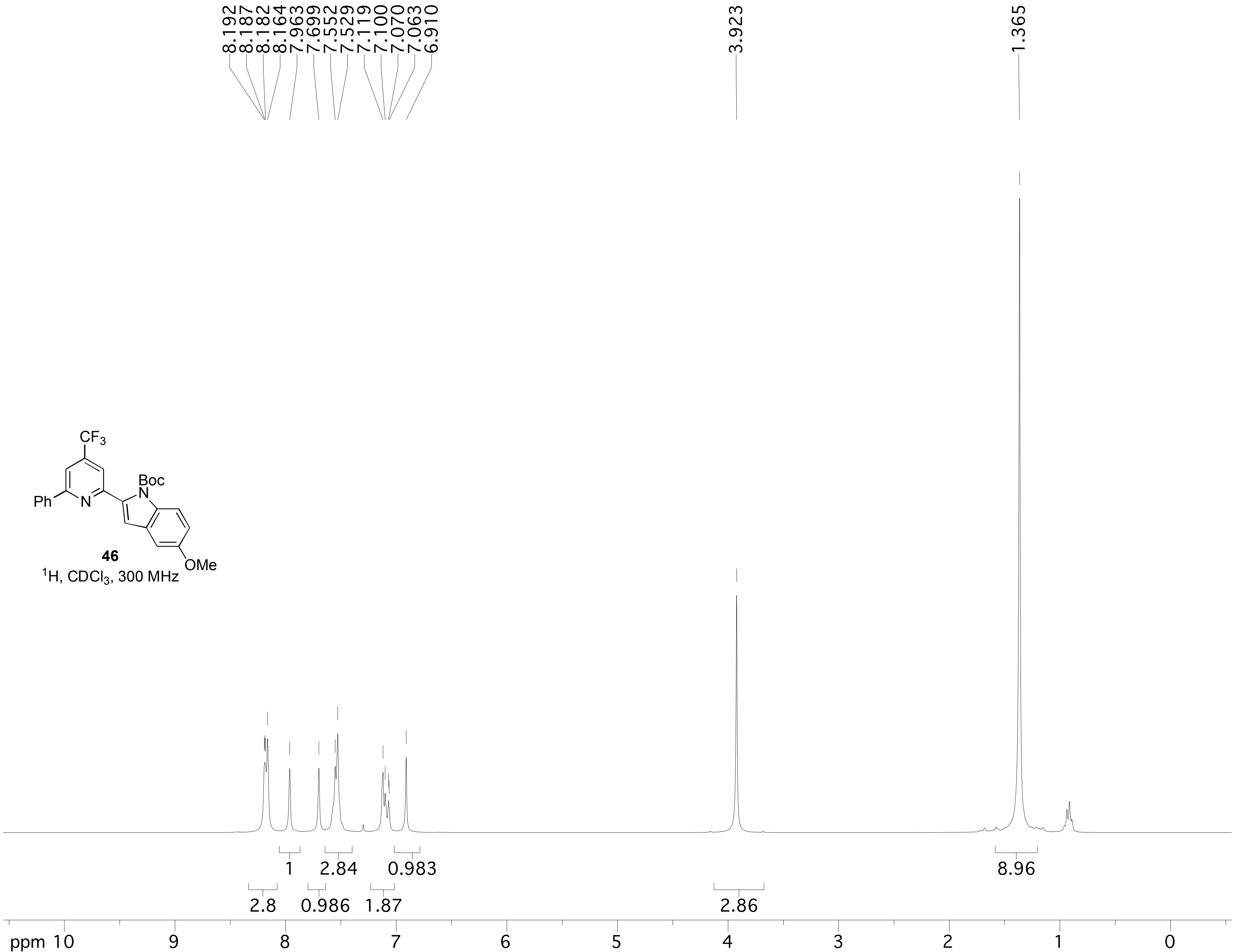
8.192
8.187
8.182
8.164
7.963
7.699
7.552
7.529
7.119
7.100
7.070
7.063
6.910
3.923
1.365
CF_3
Boc
N
Ph
N
OMe
46
^1H, $CDCl_3$, 300 MHz
1
2.84
0.983
8.96
2.8
0.986
1.87
2.86
ppm 10
9
8
7
6
5
4
3
2
1
0

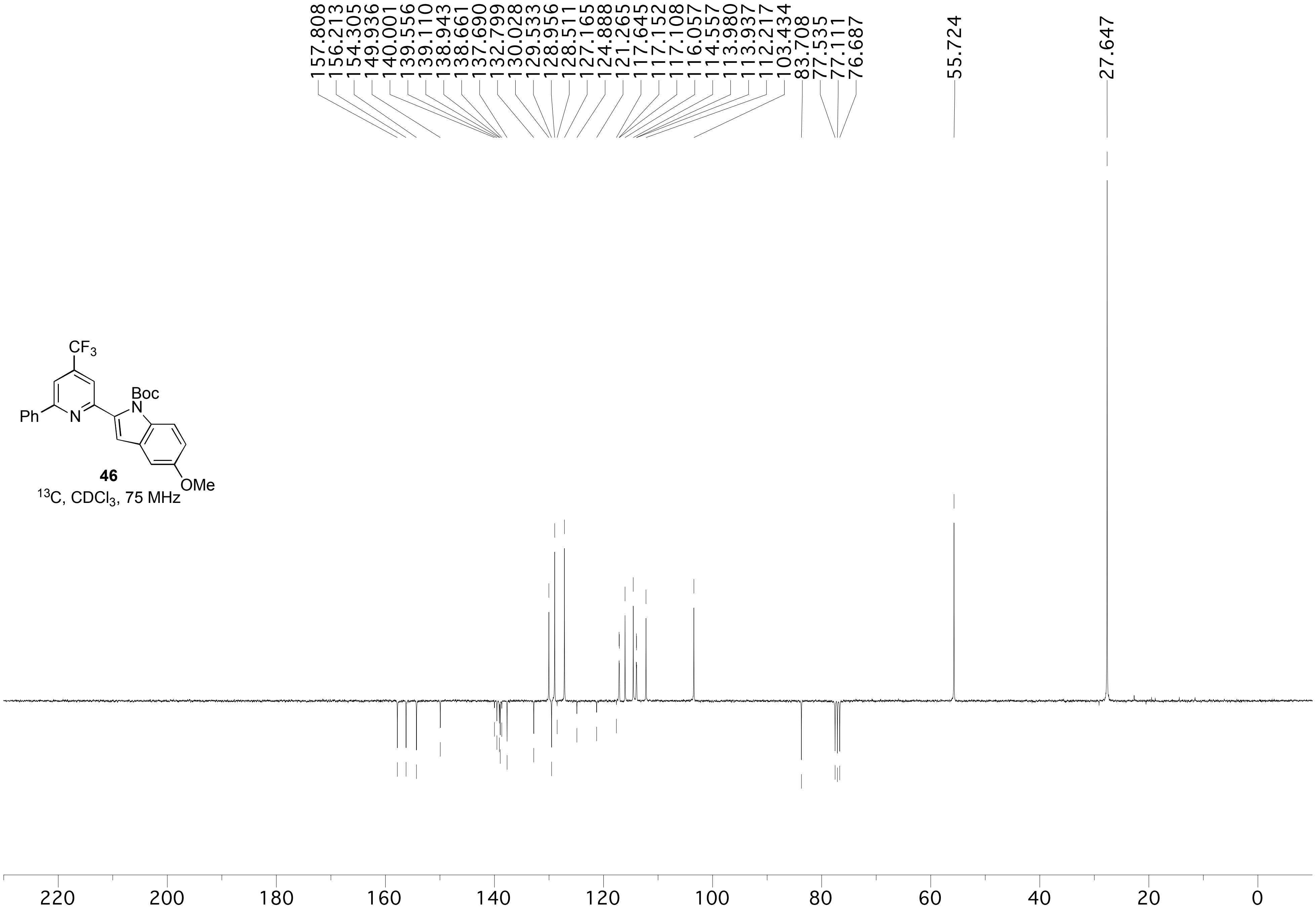
157.808
156.213
154.305
149.936
140.001
139.556
139.110
138.943
138.661
137.690
132.799
130.028
129.533
128.956
128.511
127.165
124.888
121.265
117.645
117.152
117.108
116.057
114.557
113.980
113.937
112.217
103.434
83.708
77.535
77.111
76.687
55.724
27.647
CF3
Boc
N
Ph
N
OMe
46
13C, CDCl3, 75 MHz
220
200
180
160
140
120
100
80
60
40
20
0

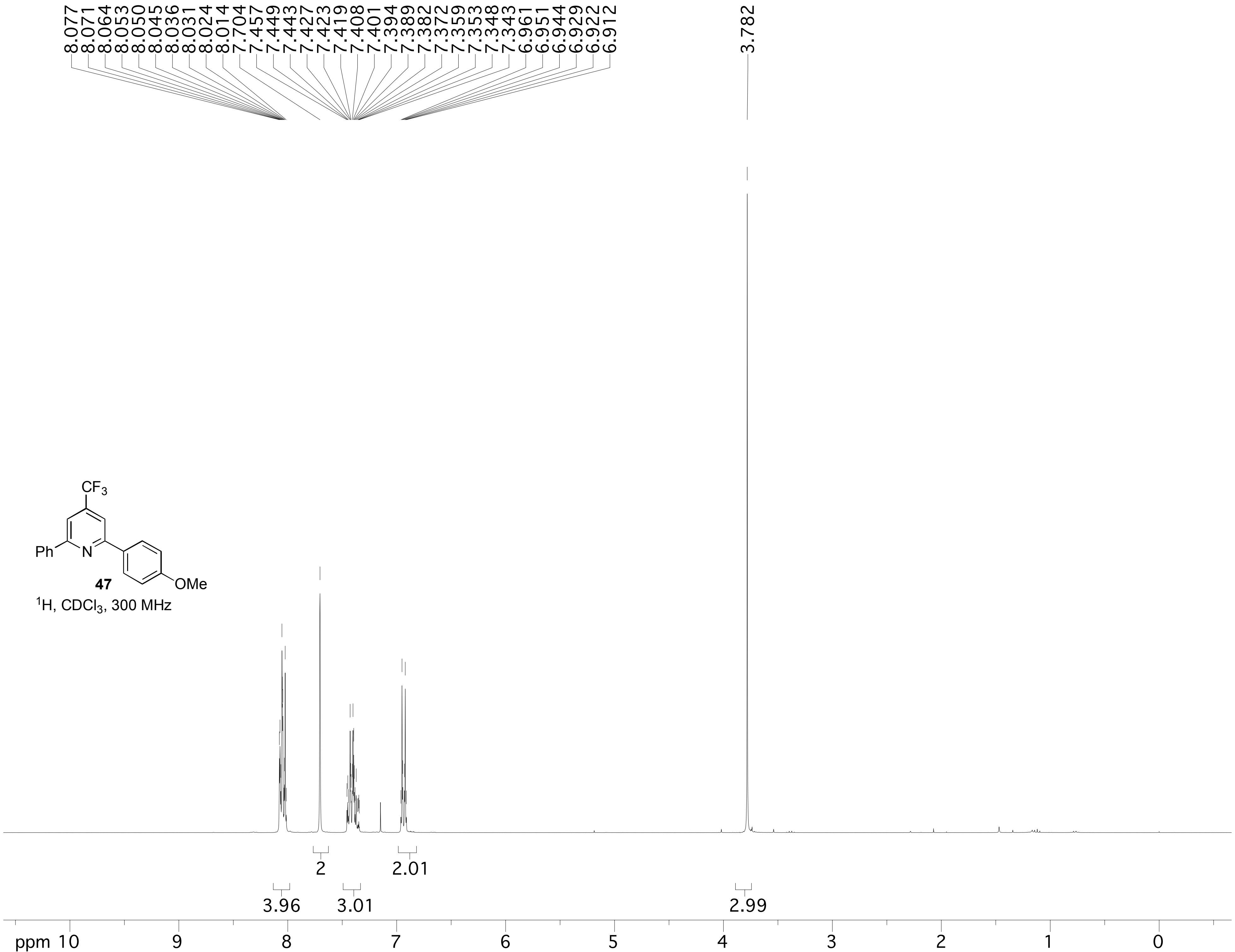
8.077
8.071
8.064
8.053
8.050
8.045
8.036
8.031
8.024
8.014
7.704
7.457
7.449
7.443
7.427
7.423
7.419
7.408
7.401
7.394
7.389
7.382
7.372
7.359
7.353
7.348
7.343
6.961
6.951
6.944
6.929
6.922
6.912
3.782
CF3
Ph
N
OMe
47
1H, CDCl3, 300 MHz
2
2.01
3.96
3.01
2.99
ppm 10
9
8
7
6
5
4
3
2
1
0

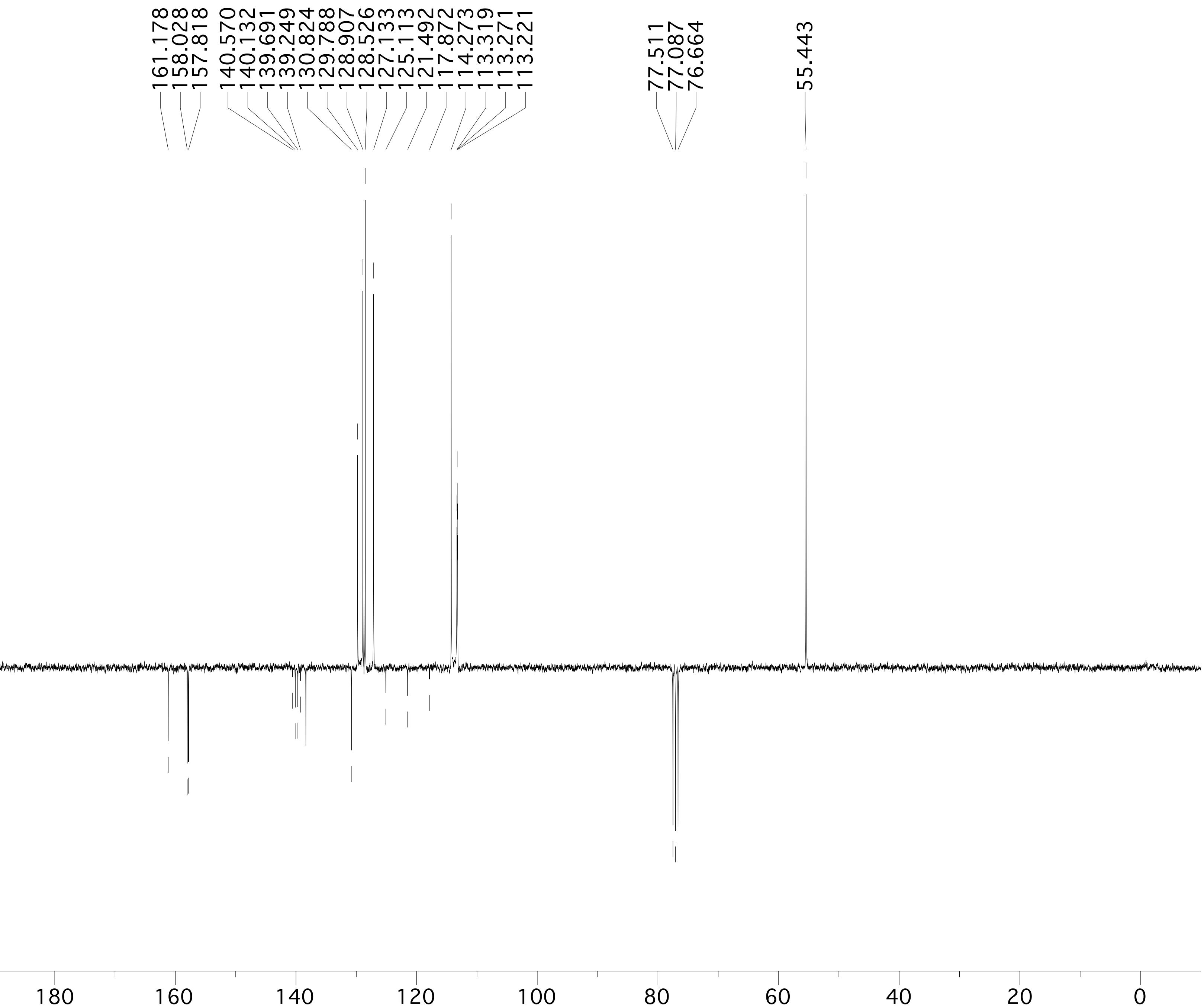

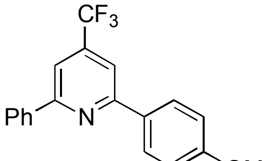

47

^{13}C, $CDCl_3$, 75 MHz

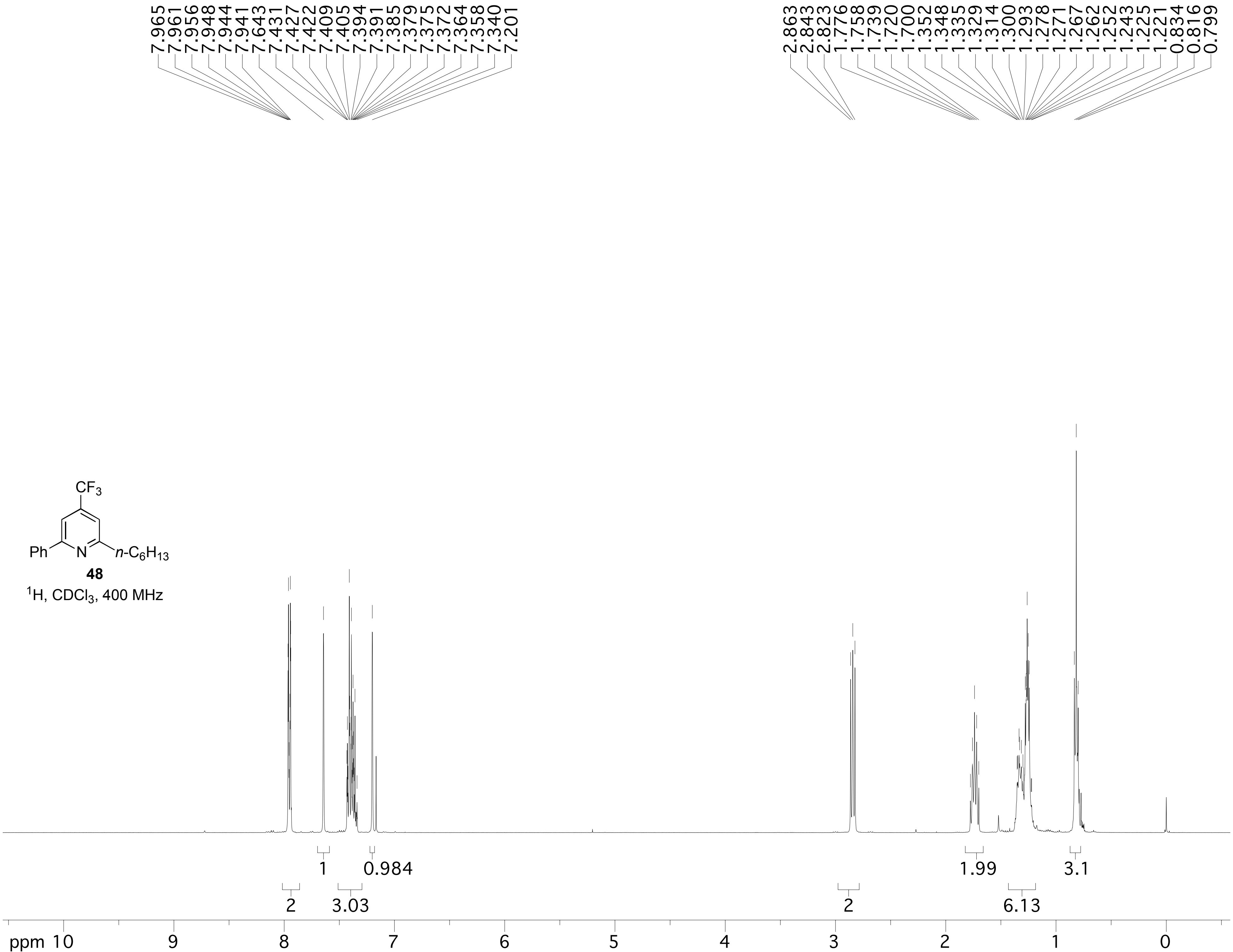

7.965 7.961 7.956 7.948 7.944 7.941 7.643 7.431 7.427 7.422 7.409 7.405 7.394 7.391 7.385 7.379 7.375 7.372 7.364 7.358 7.340 7.201
2.863 2.843 2.823 1.776 1.758 1.739 1.720 1.700 1.352 1.348 1.335 1.329 1.314 1.300 1.293 1.278 1.271 1.267 1.262 1.252 1.243 1.225 1.221 0.834 0.816 0.799
CF3
Ph
N
n-C6H13
48
1H, CDCl3, 400 MHz
1
0.984
1.99
3.1
2
3.03
2
6.13
ppm 10
9
8
7
6
5
4
3
2
1
0

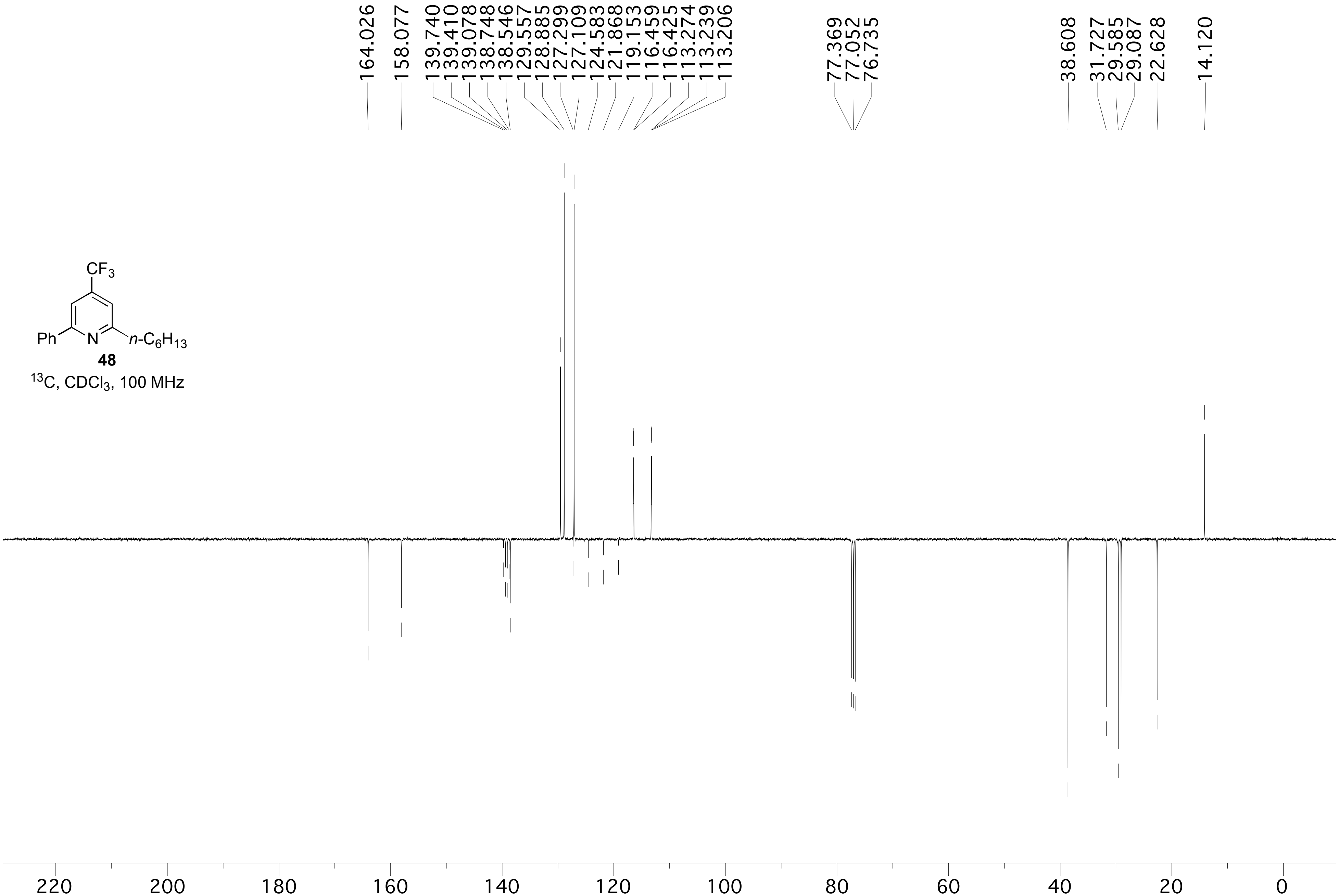

48

^{13}C, $CDCl_3$, 100 MHz

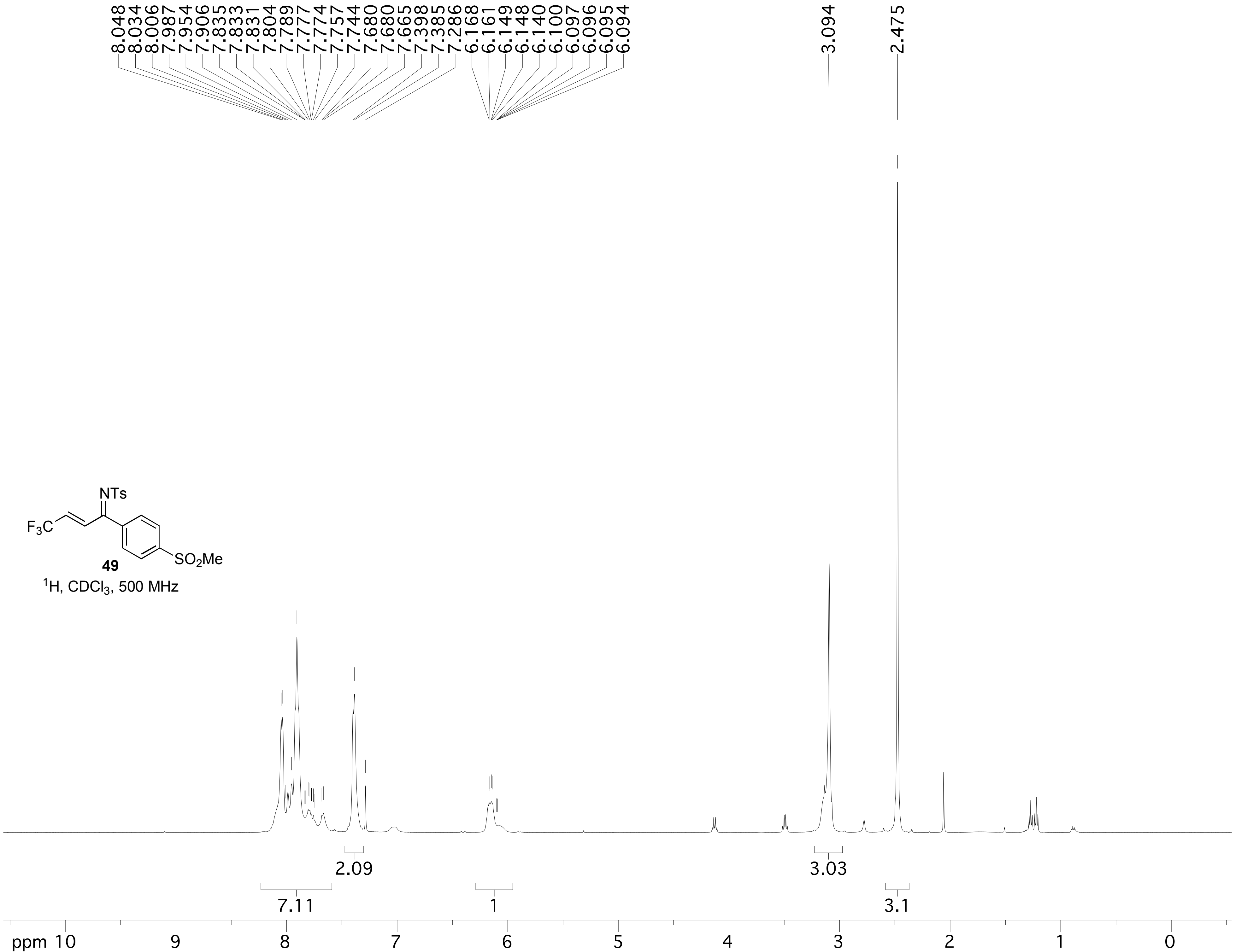
8.048 8.034 8.006 7.987 7.954 7.906 7.835 7.833 7.831 7.804 7.789 7.777 7.774 7.757 7.744 7.680 7.680 7.665 7.398 7.385 7.286 6.168 6.161 6.149 6.148 6.140 6.100 6.097 6.096 6.095 6.094
3.094
2.475
NTs
F3C
SO2Me
49
1H, CDCl3, 500 MHz
2.09
3.03
7.11
1
3.1
ppm 10 9 8 7 6 5 4 3 2 1 0

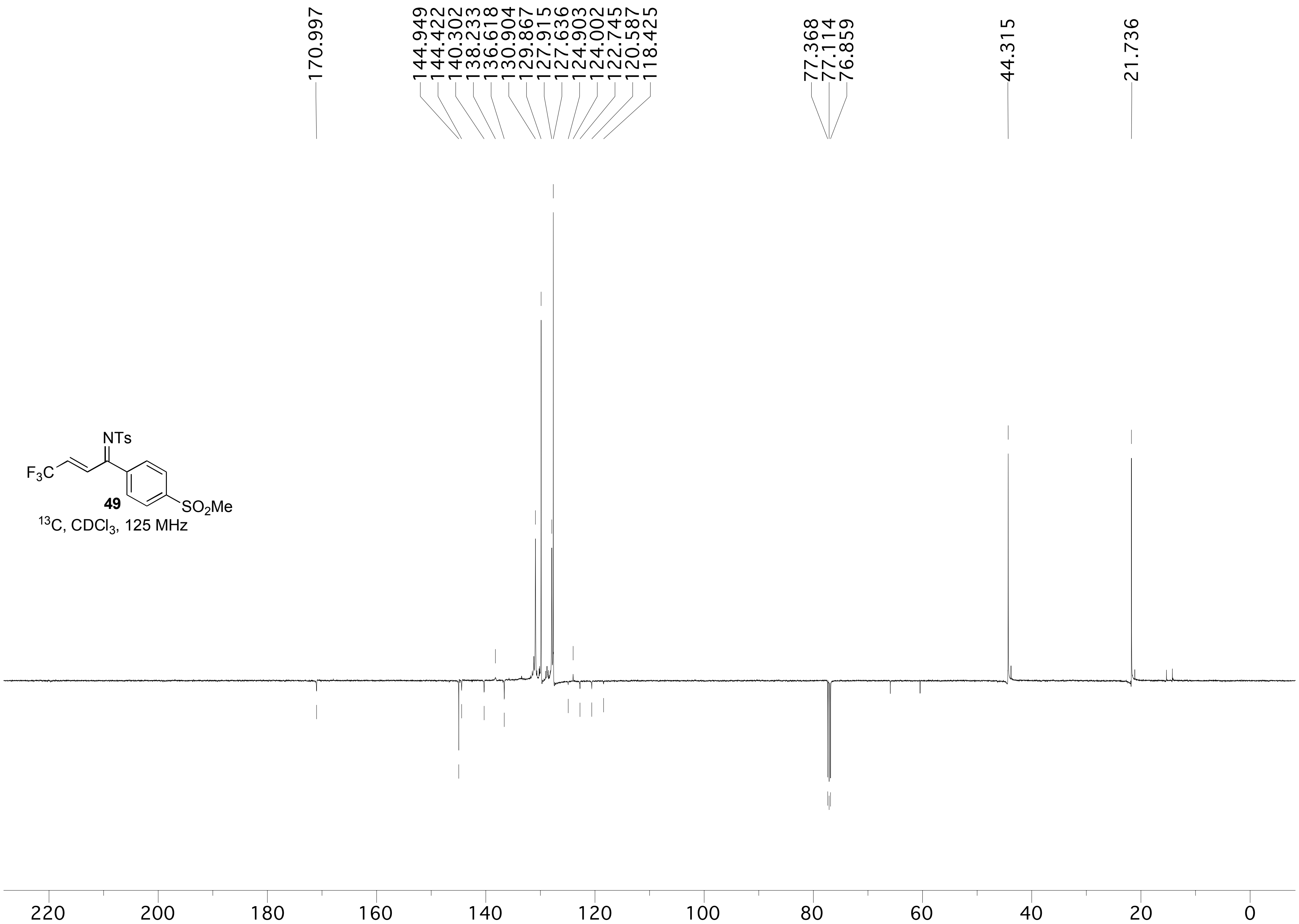
170.997
144.949
144.422
140.302
138.233
136.618
130.904
129.867
127.915
127.636
124.903
124.002
122.745
120.587
118.425
77.368
77.114
76.859
44.315
21.736
NTs
F_3C
49
SO_2Me
^{13}C, $CDCl_3$, 125 MHz
220
200
180
160
140
120
100
80
60
40
20
0

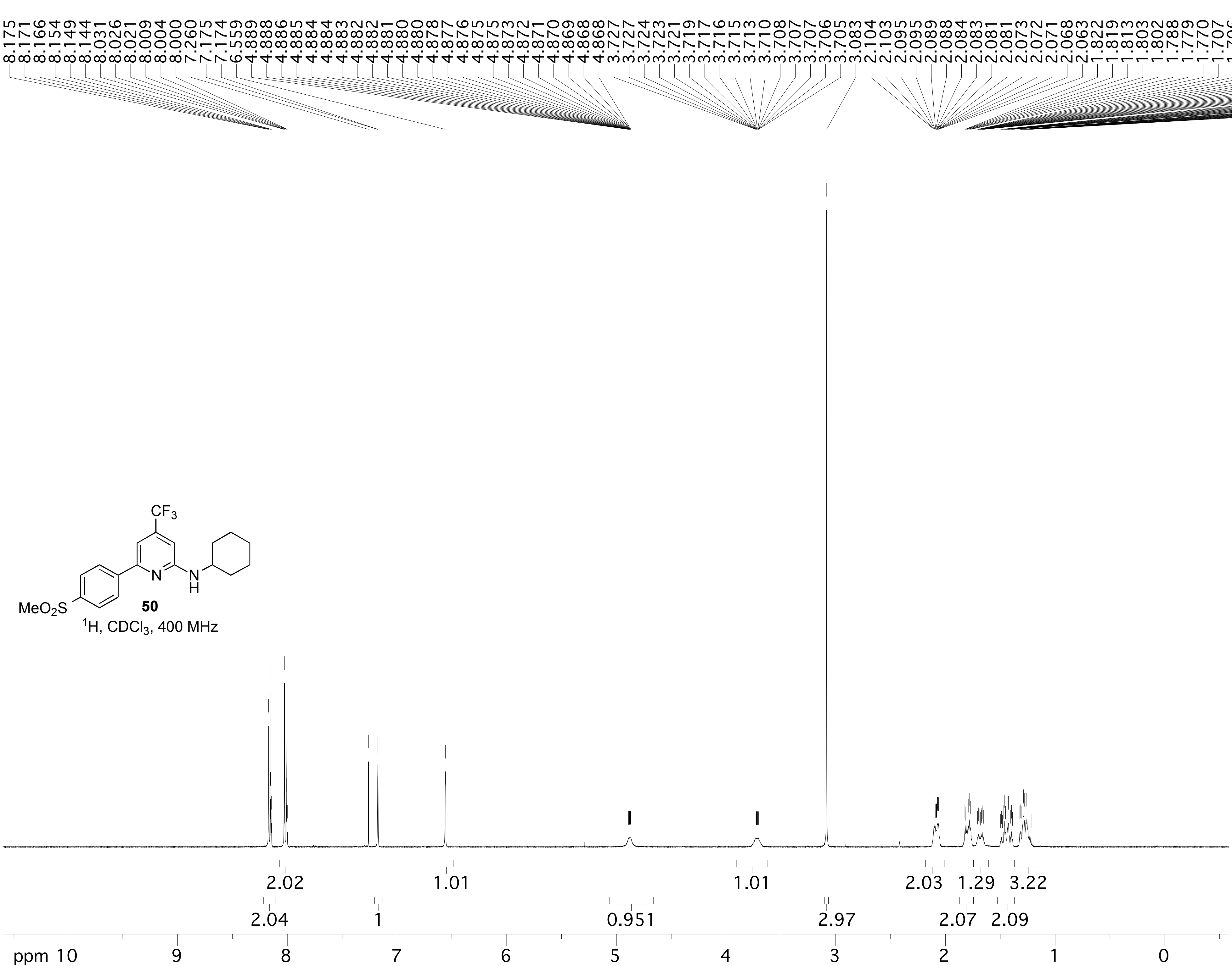

50

^{1}H, $CDCl_3$, 400 MHz

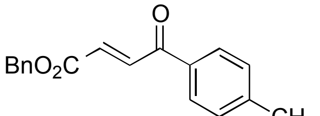

62

^1H, $CDCl_3$, 300 MHz

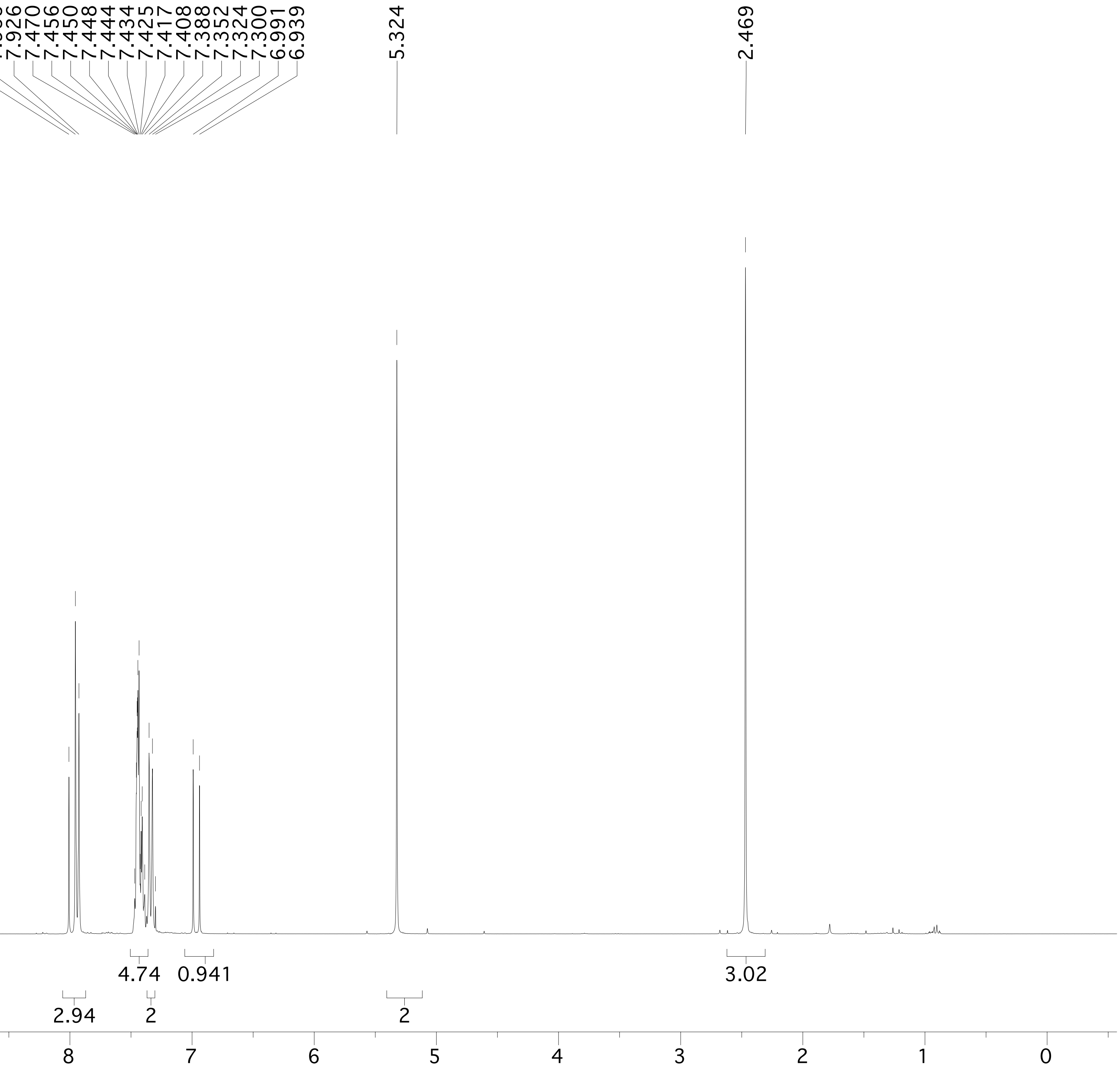

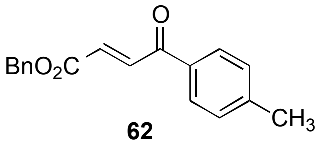

62

^{13}C, $CDCl_3$, 75 MHz

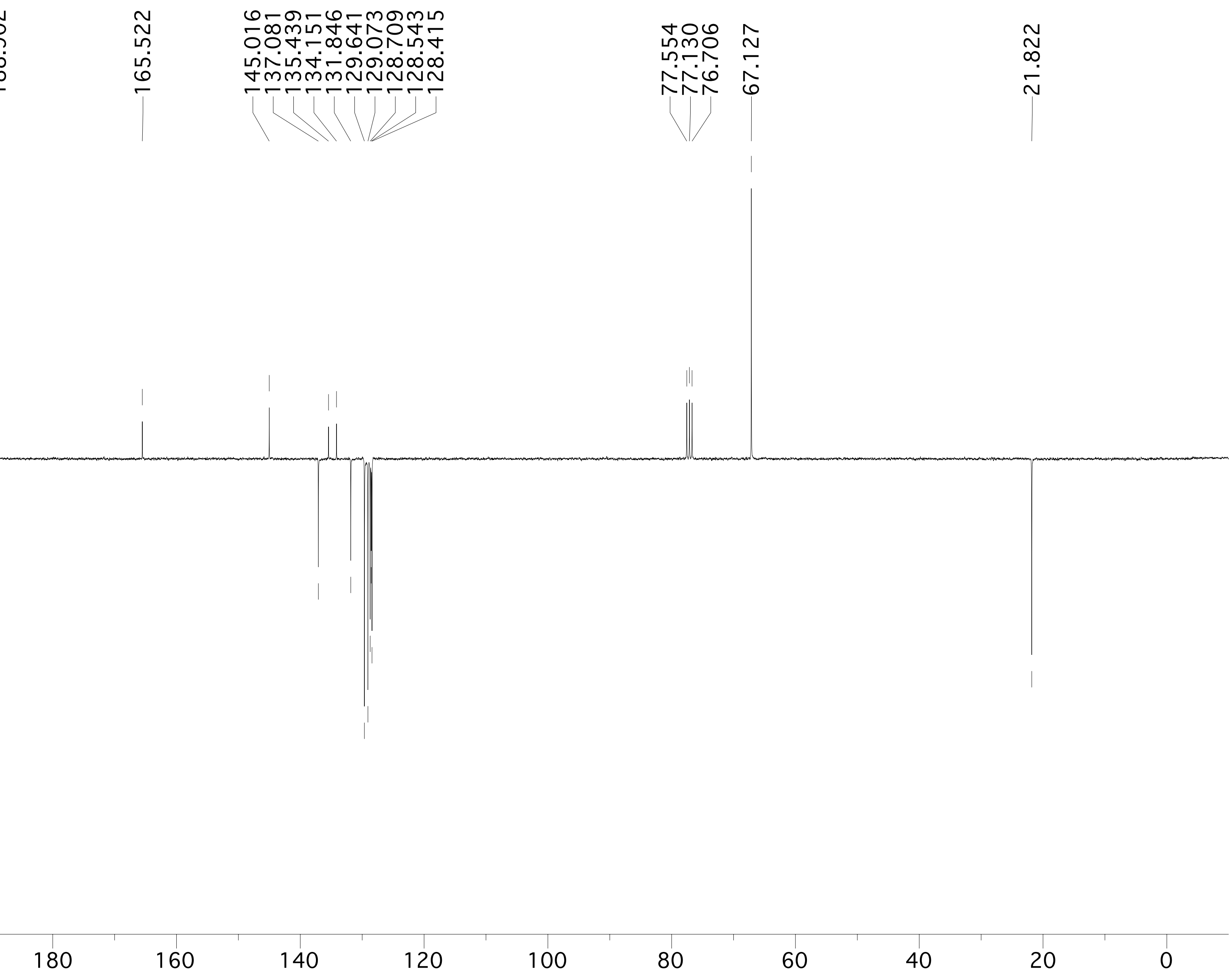

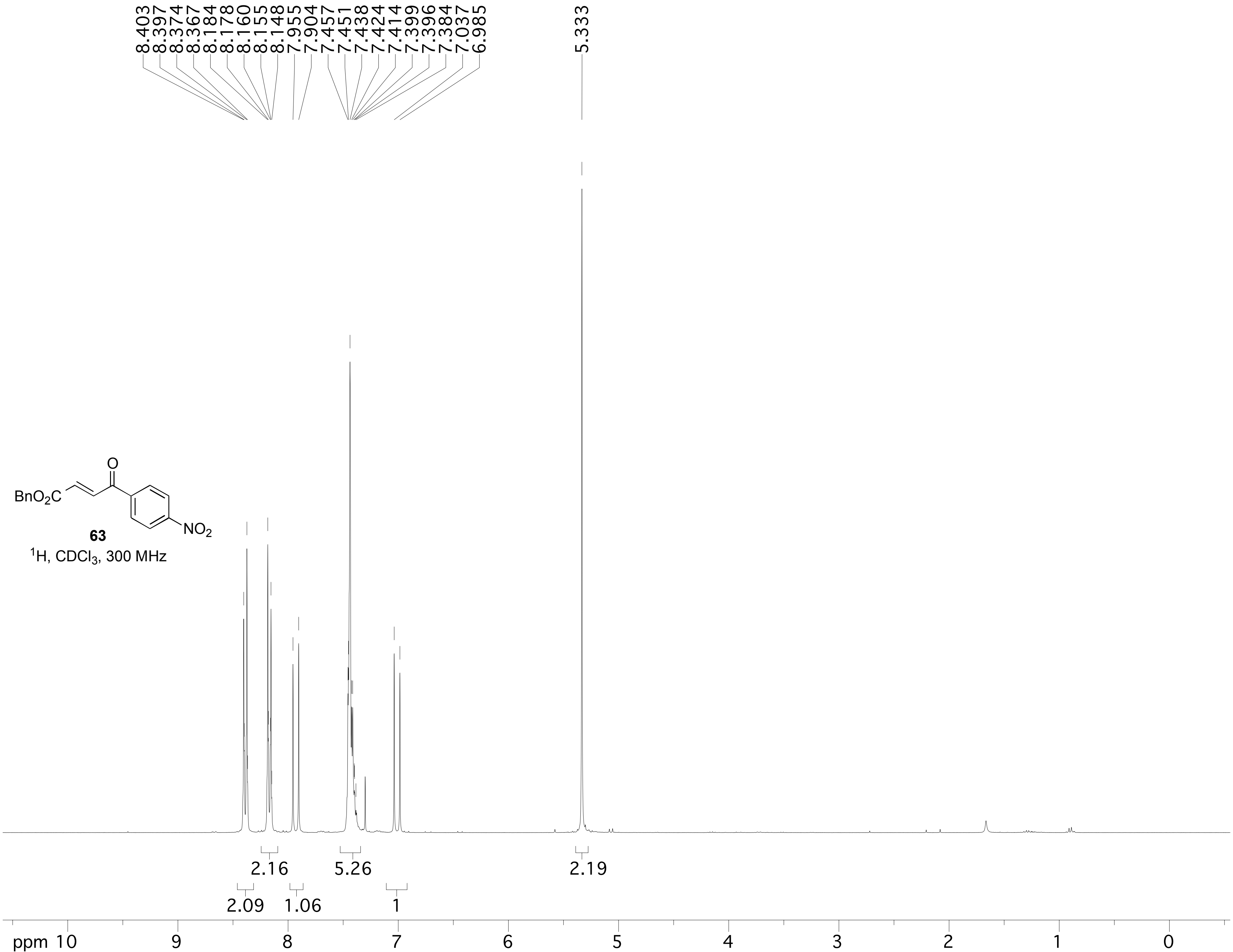
8.403
8.397
8.374
8.367
8.184
8.178
8.160
8.155
8.148
7.955
7.904
7.457
7.451
7.438
7.424
7.414
7.399
7.396
7.384
7.037
6.985
5.333
BnO2C
O
NO2
63
1H, CDCl3, 300 MHz
2.16
5.26
2.19
2.09
1.06
1
ppm 10
9
8
7
6
5
4
3
2
1
0

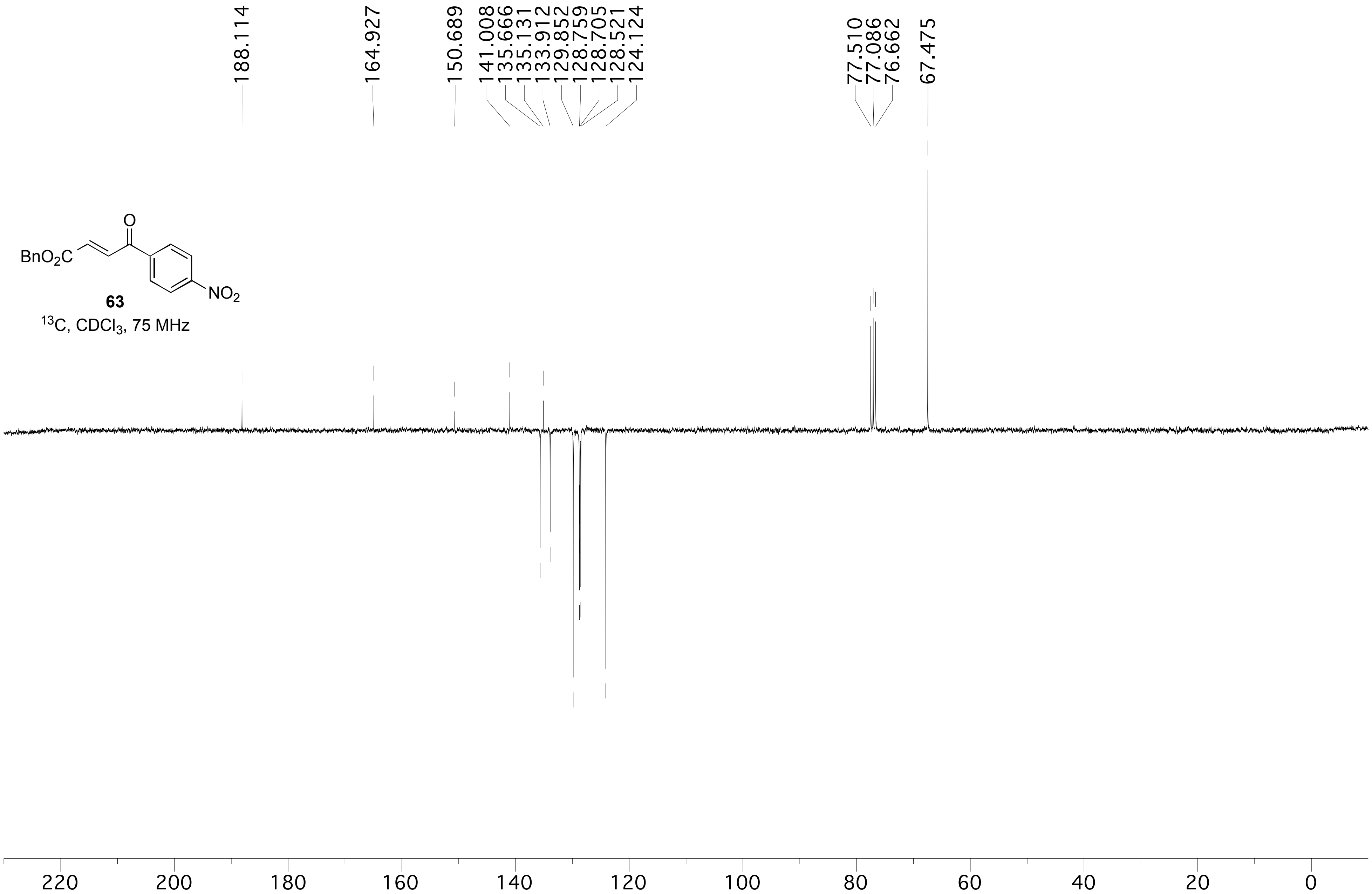

BnO2C
O
NO2
63
13C, CDCl3, 75 MHz
188.114
164.927
150.689
141.008
135.666
135.131
133.912
129.852
128.759
128.705
128.521
124.124
77.510
77.086
76.662
67.475
220
200
180
160
140
120
100
80
60
40
20
0

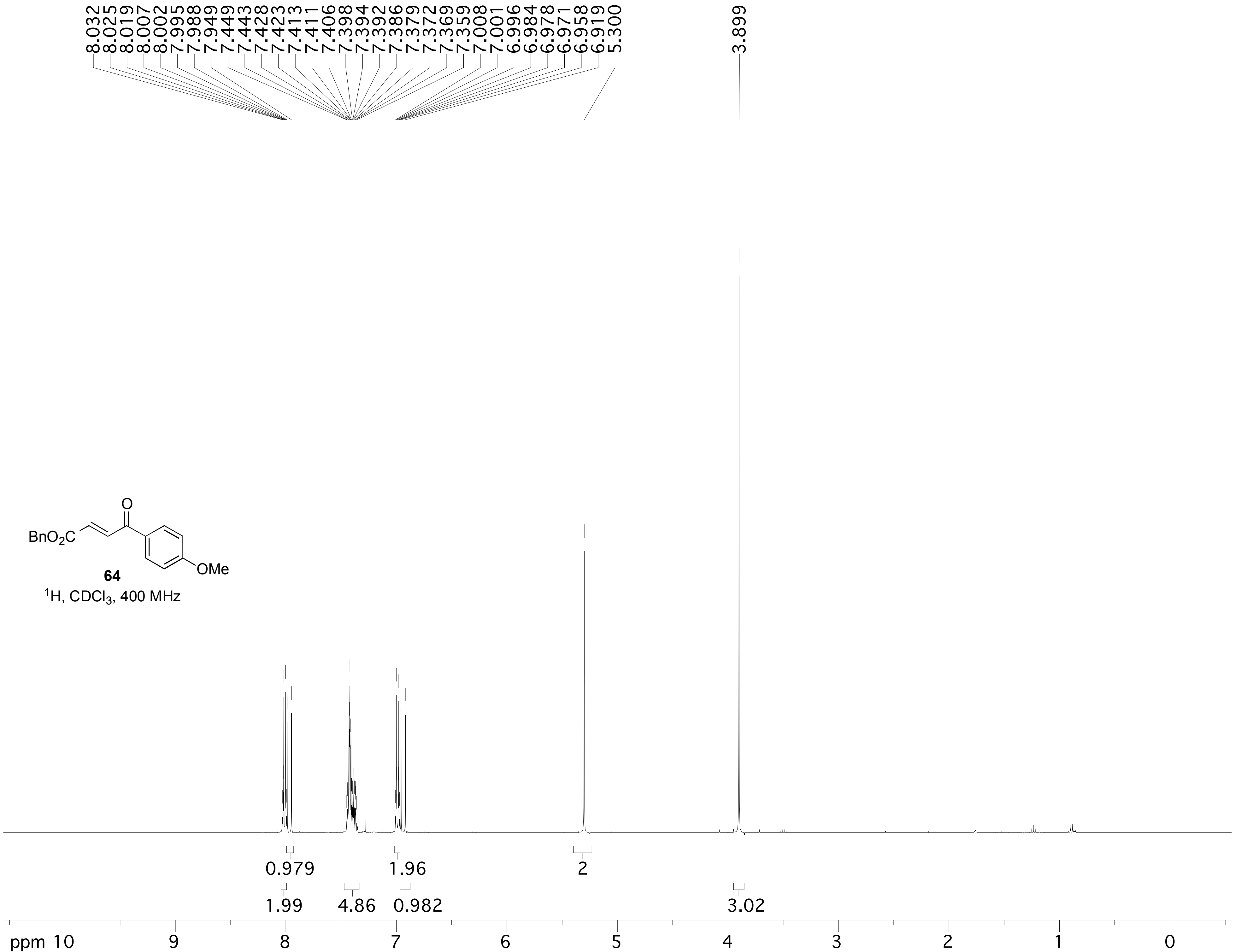

64

^{1}H, $CDCl_3$, 400 MHz

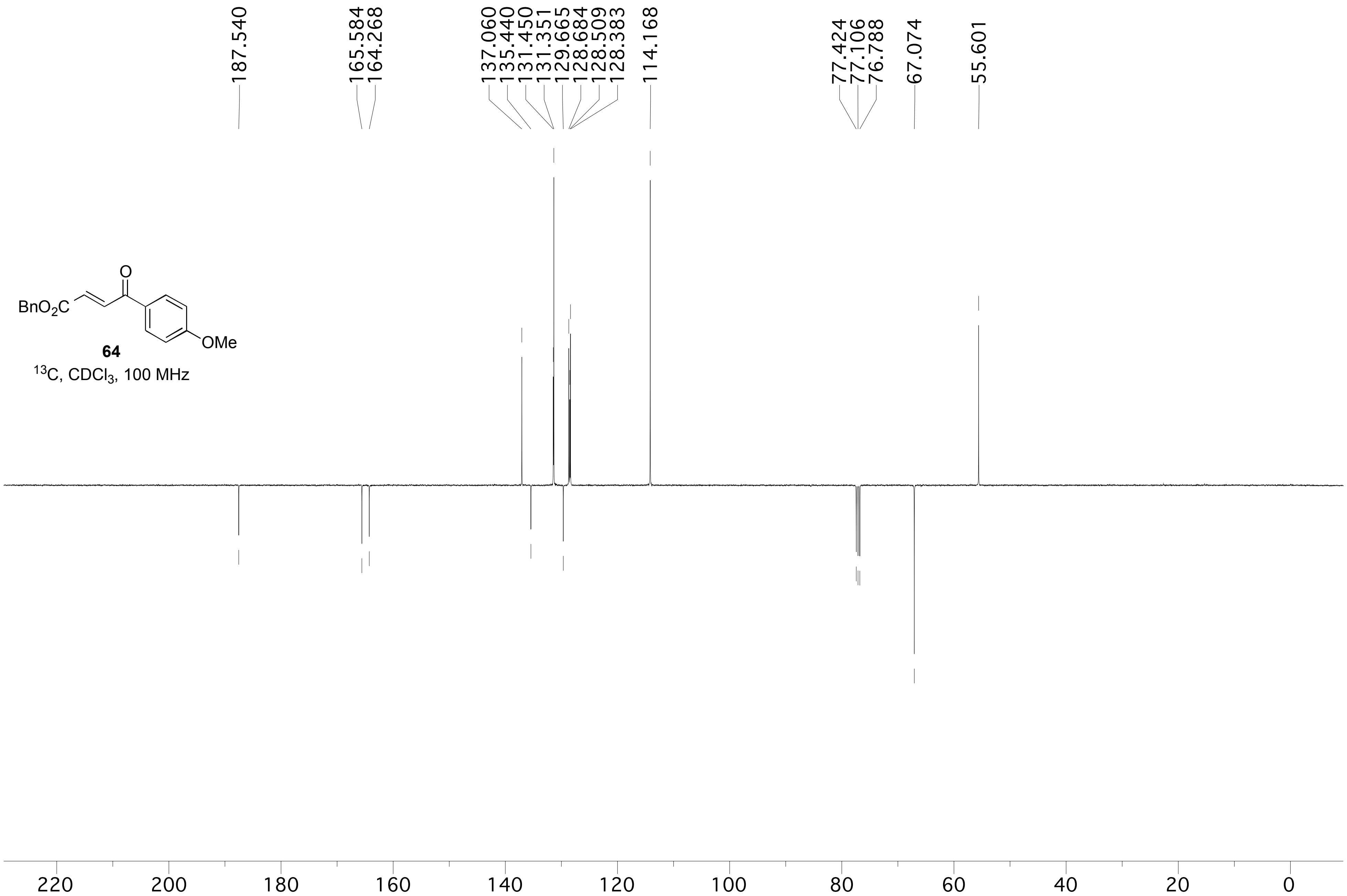

64

^{13}C, $CDCl_3$, 100 MHz

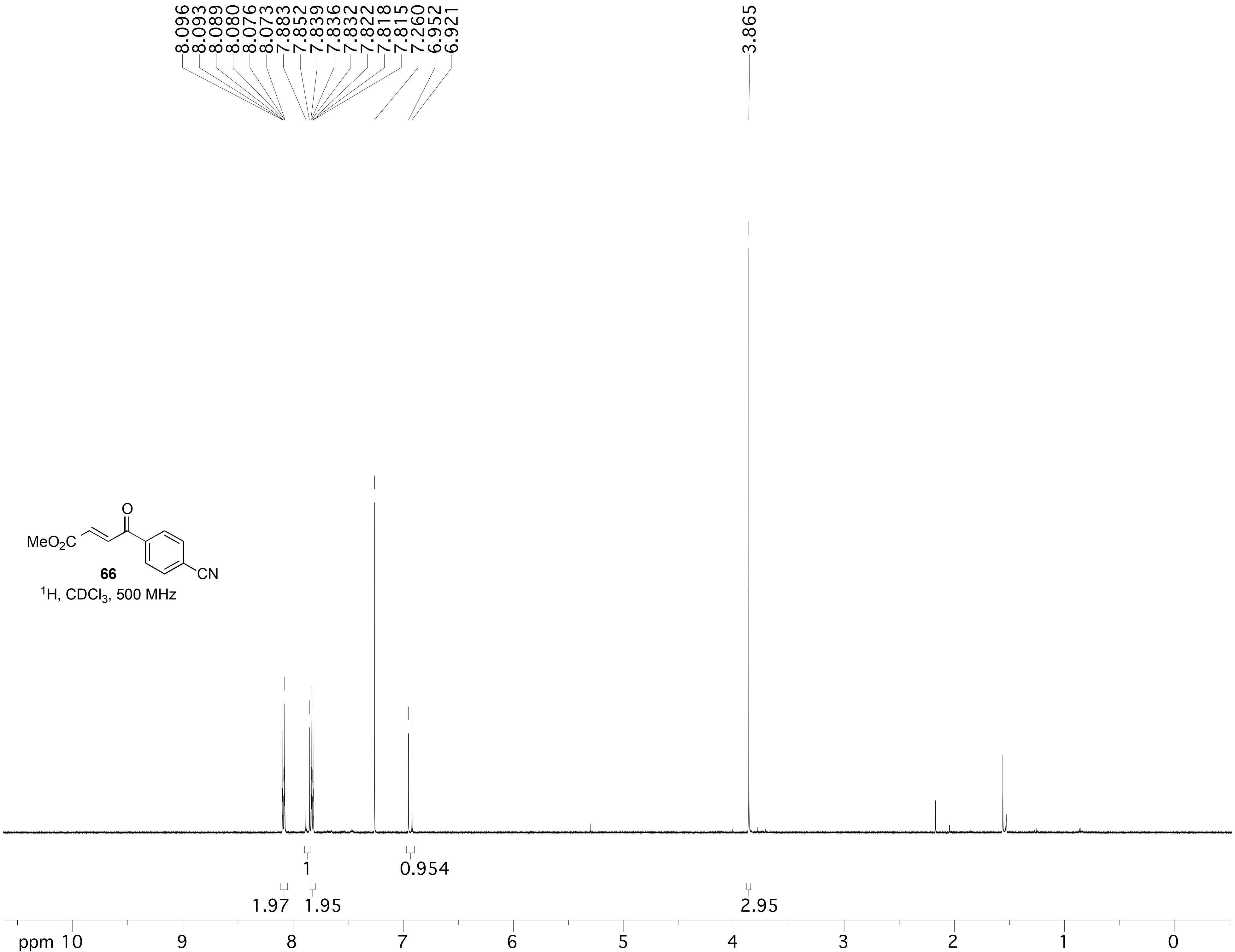

66

^{1}H, $CDCl_3$, 500 MHz

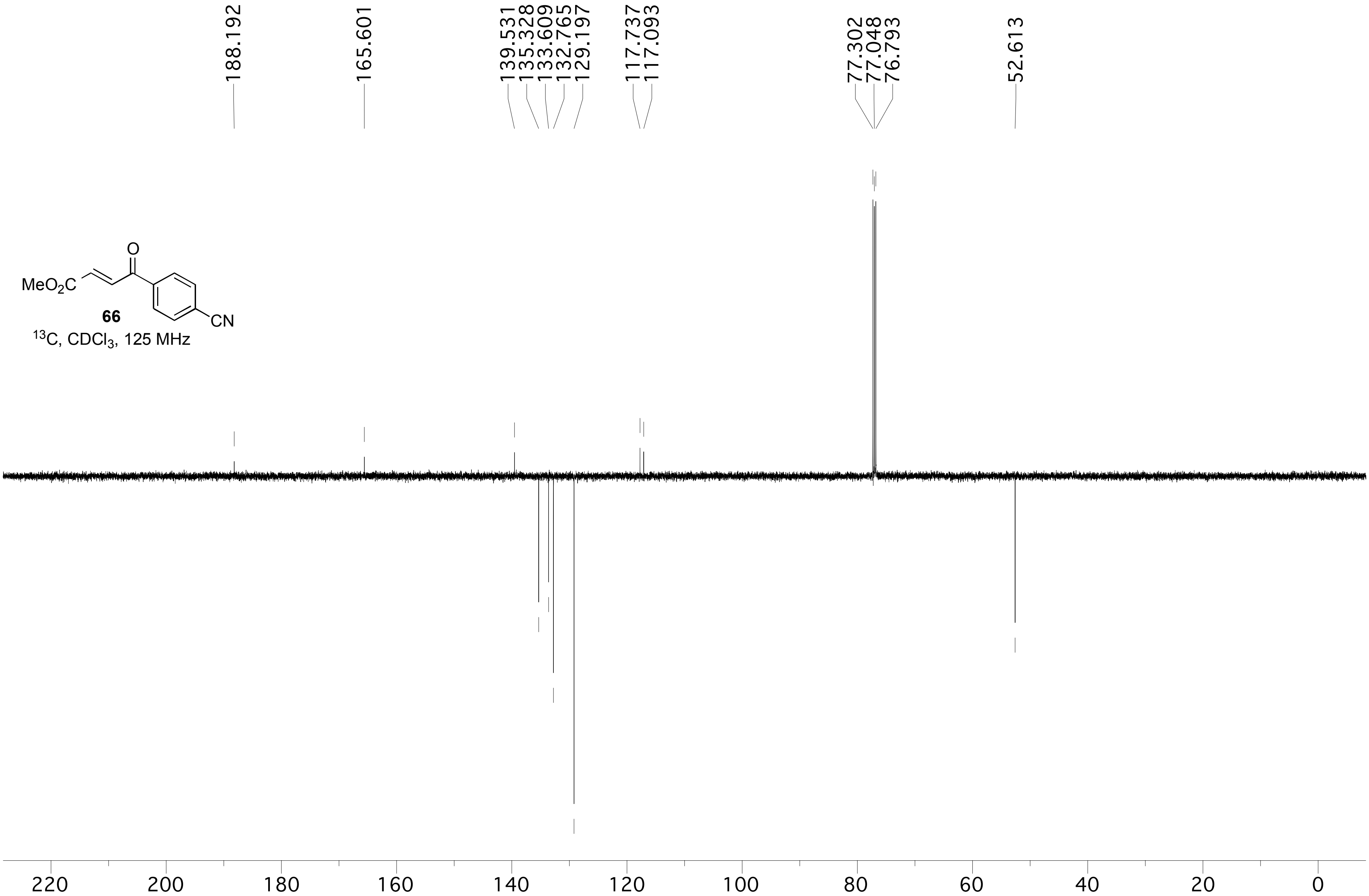

188.192
165.601
139.531
135.328
133.609
132.765
129.197
117.737
117.093
77.302
77.048
76.793
52.613
O
MeO2C
CN
66
13C, CDCl3, 125 MHz
220 200 180 160 140 120 100 80 60 40 20 0

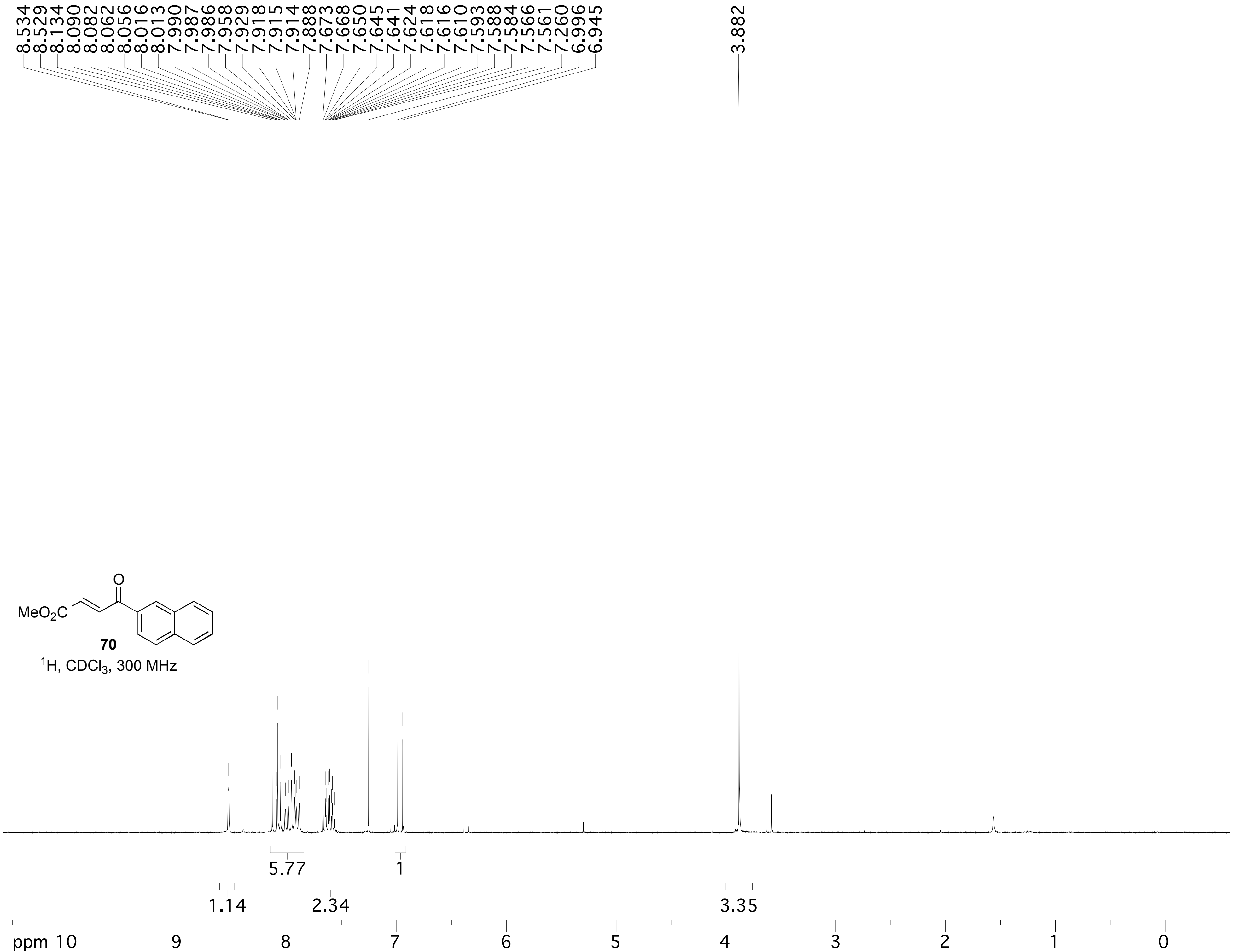

70

^1H, $CDCl_3$, 300 MHz

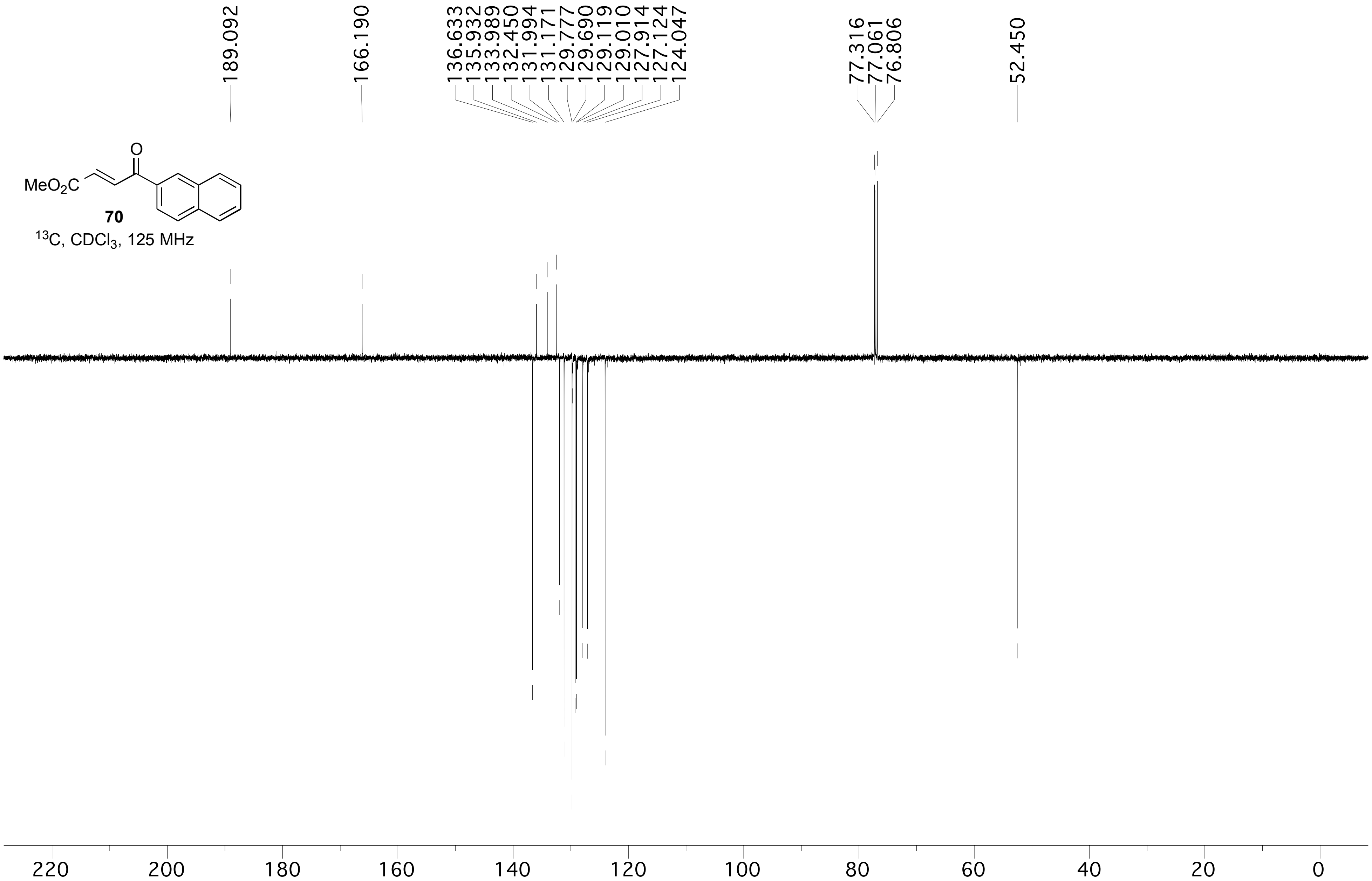

189.092
166.190
136.633
135.932
133.989
132.450
131.994
131.171
129.777
129.690
129.119
129.010
127.914
127.124
124.047
77.316
77.061
76.806
52.450
MeO2C
O
70
13C, CDCl3, 125 MHz
220
200
180
160
140
120
100
80
60
40
20
0

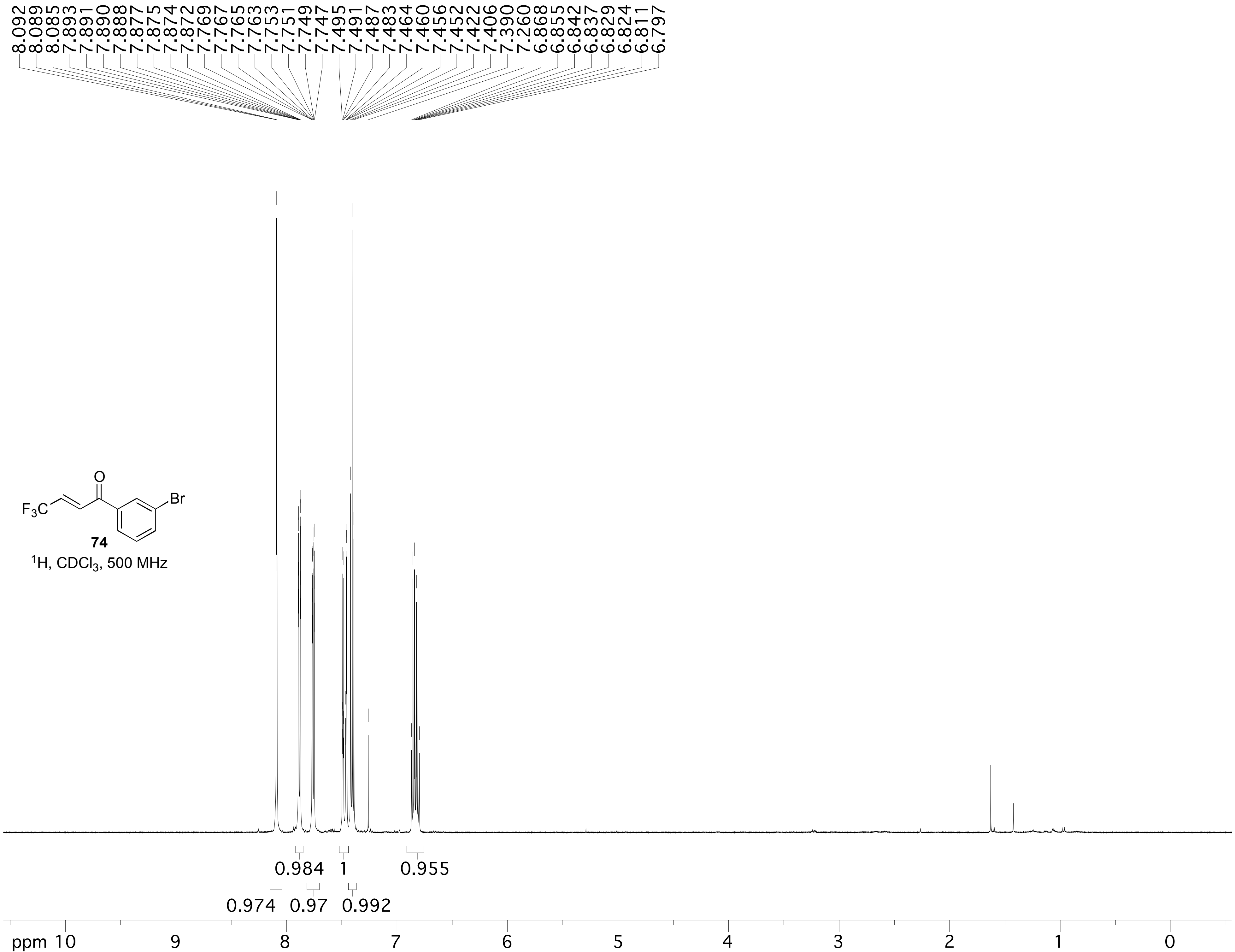
8.092
8.089
8.085
7.893
7.891
7.890
7.888
7.877
7.875
7.874
7.872
7.769
7.767
7.765
7.763
7.753
7.751
7.749
7.747
7.495
7.491
7.487
7.483
7.464
7.460
7.456
7.452
7.422
7.406
7.390
7.260
6.868
6.855
6.842
6.837
6.829
6.824
6.811
6.797
O
Br
F_3C
74
^1H, $CDCl_3$, 500 MHz
0.984
1
0.955
0.974
0.97
0.992
ppm 10
9
8
7
6
5
4
3
2
1
0

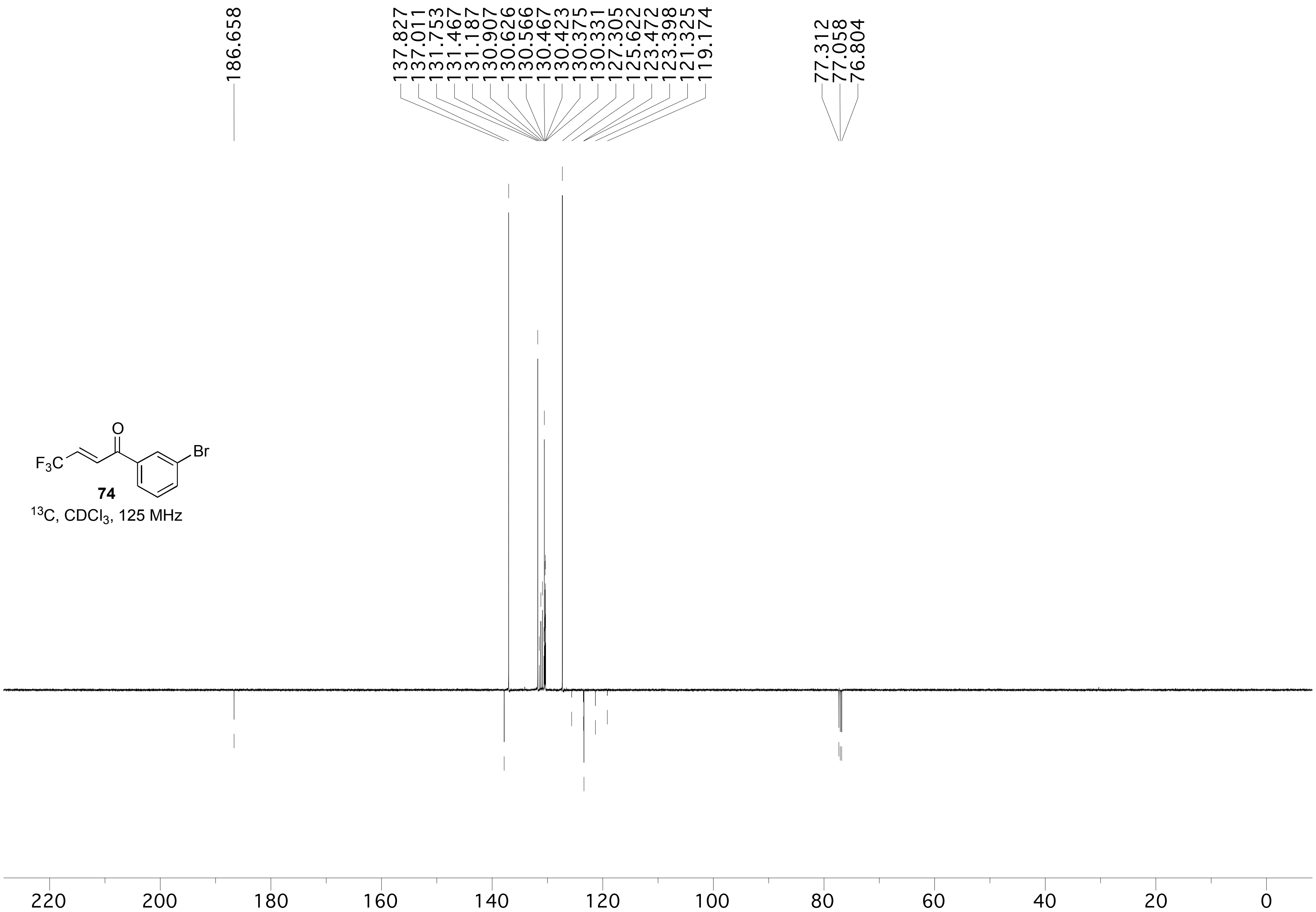
186.658
137.827
137.011
131.753
131.467
131.187
130.907
130.626
130.566
130.467
130.423
130.375
130.331
127.305
125.622
123.472
123.398
121.325
119.174
77.312
77.058
76.804
O
Br
F3C
74
13C, CDCl3, 125 MHz
220
200
180
160
140
120
100
80
60
40
20
0

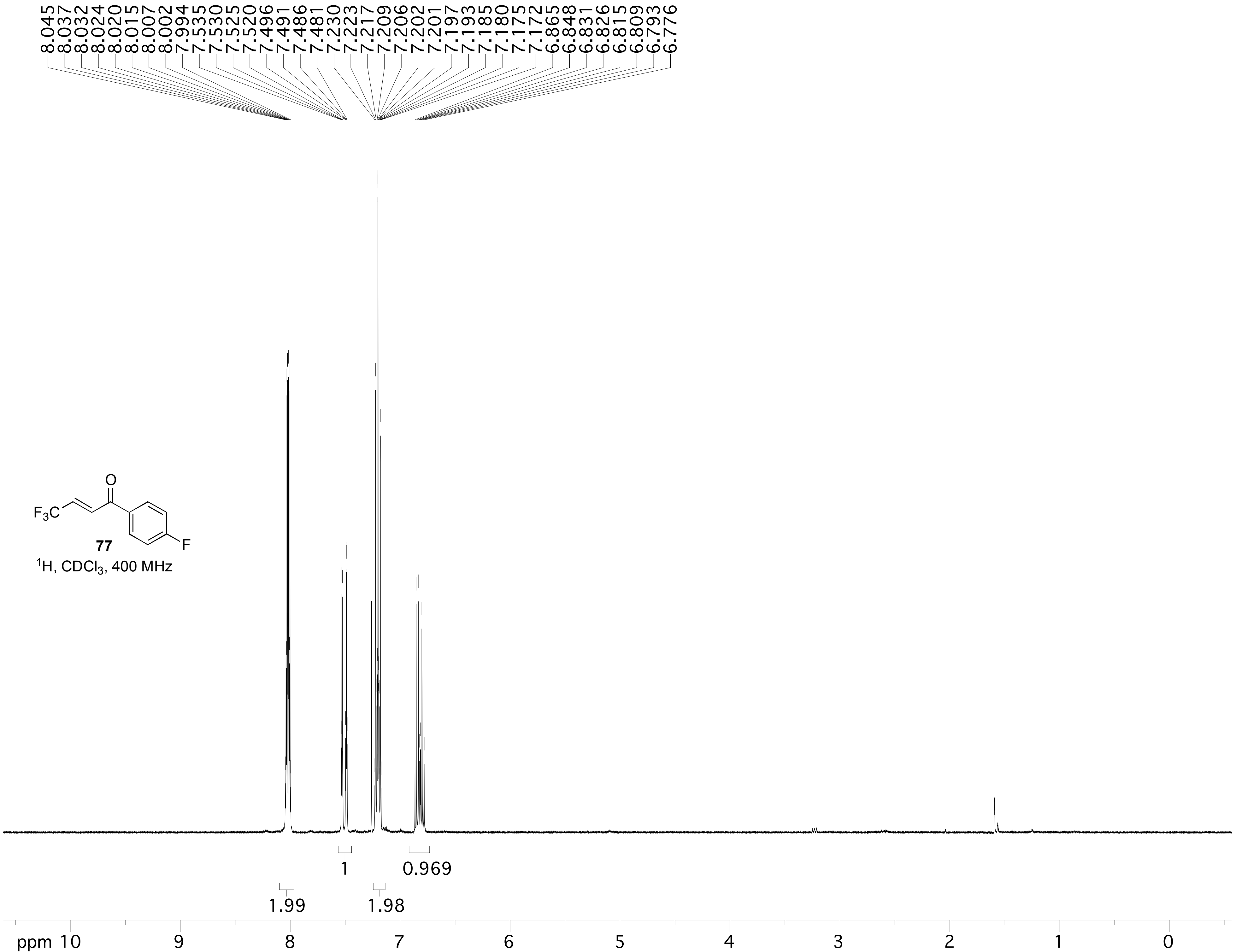

8.045
8.037
8.032
8.024
8.020
8.015
8.007
8.002
7.994
7.535
7.530
7.525
7.520
7.496
7.491
7.486
7.481
7.230
7.223
7.217
7.209
7.206
7.202
7.201
7.197
7.193
7.185
7.180
7.175
7.172
6.865
6.848
6.831
6.826
6.815
6.809
6.793
6.776
O
F3C
F
77
1H, CDCl3, 400 MHz
1
0.969
1.99
1.98
ppm 10
9
8
7
6
5
4
3
2
1
0

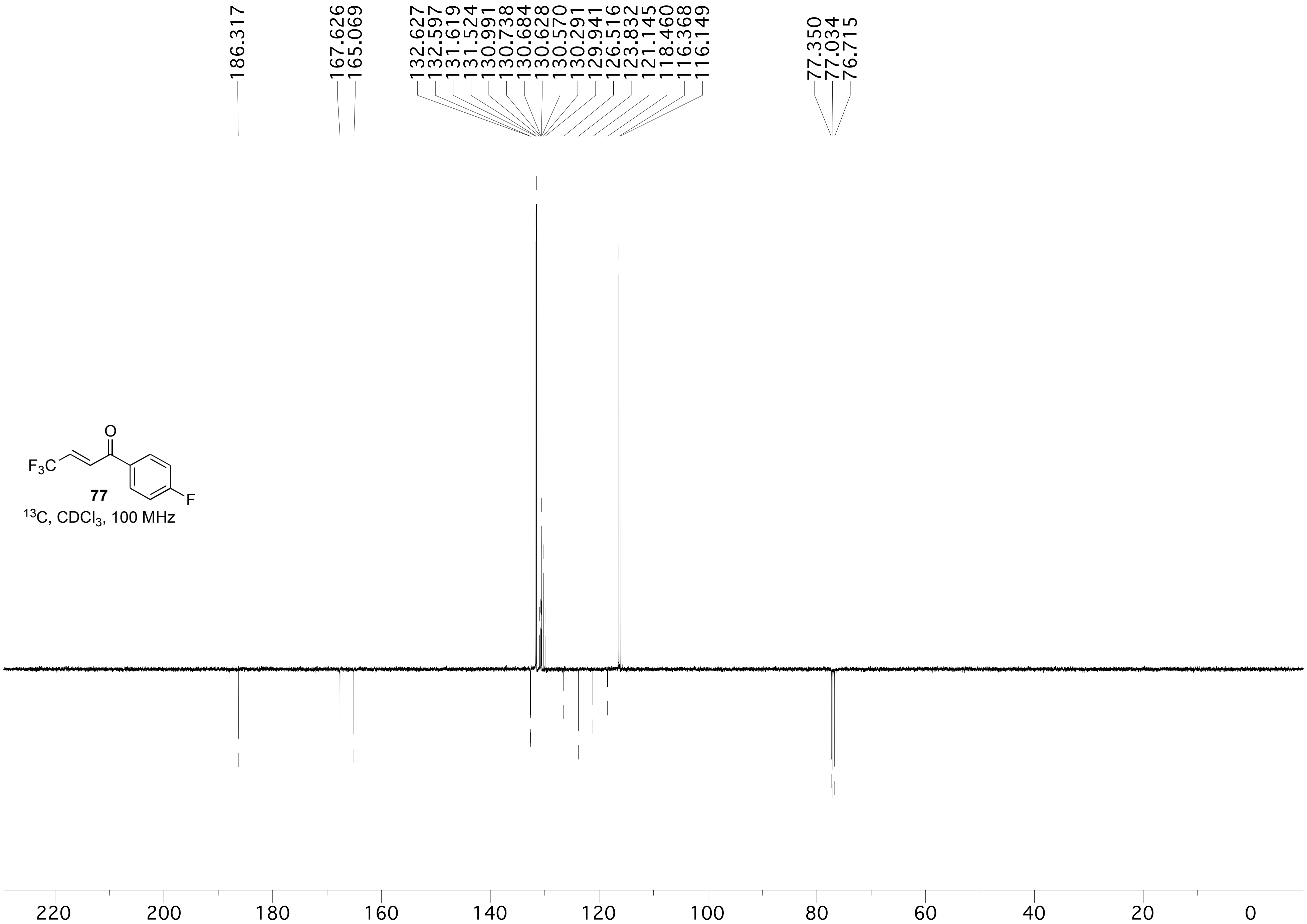

186.317
167.626
165.069
132.627
132.597
131.619
131.524
130.991
130.738
130.684
130.628
130.570
130.291
129.941
126.516
123.832
121.145
118.460
116.368
116.149
77.350
77.034
76.715
O
F3C
F
77
13C, CDCl3, 100 MHz
220
200
180
160
140
120
100
80
60
40
20
0

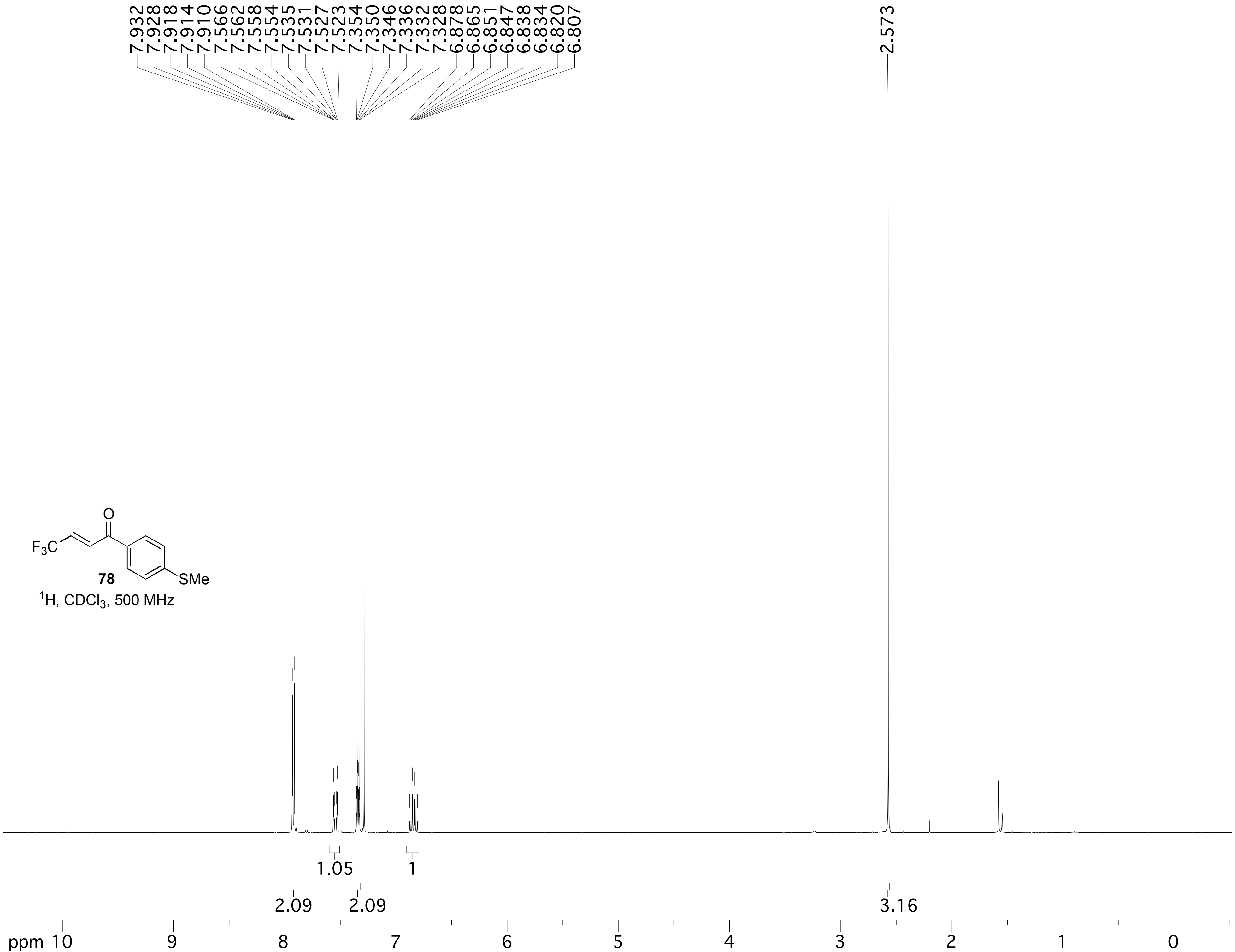

7.932
7.928
7.918
7.914
7.910
7.566
7.562
7.558
7.554
7.535
7.531
7.527
7.523
7.354
7.350
7.346
7.336
7.332
7.328
6.878
6.865
6.851
6.847
6.838
6.834
6.820
6.807
2.573
O
F3C
SMe
78
1H, CDCl3, 500 MHz
1.05
1
2.09
2.09
3.16
ppm 10
9
8
7
6
5
4
3
2
1
0

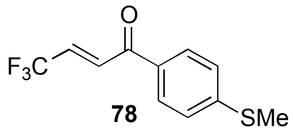

78

^{13}C, $CDCl_3$, 125 MHz

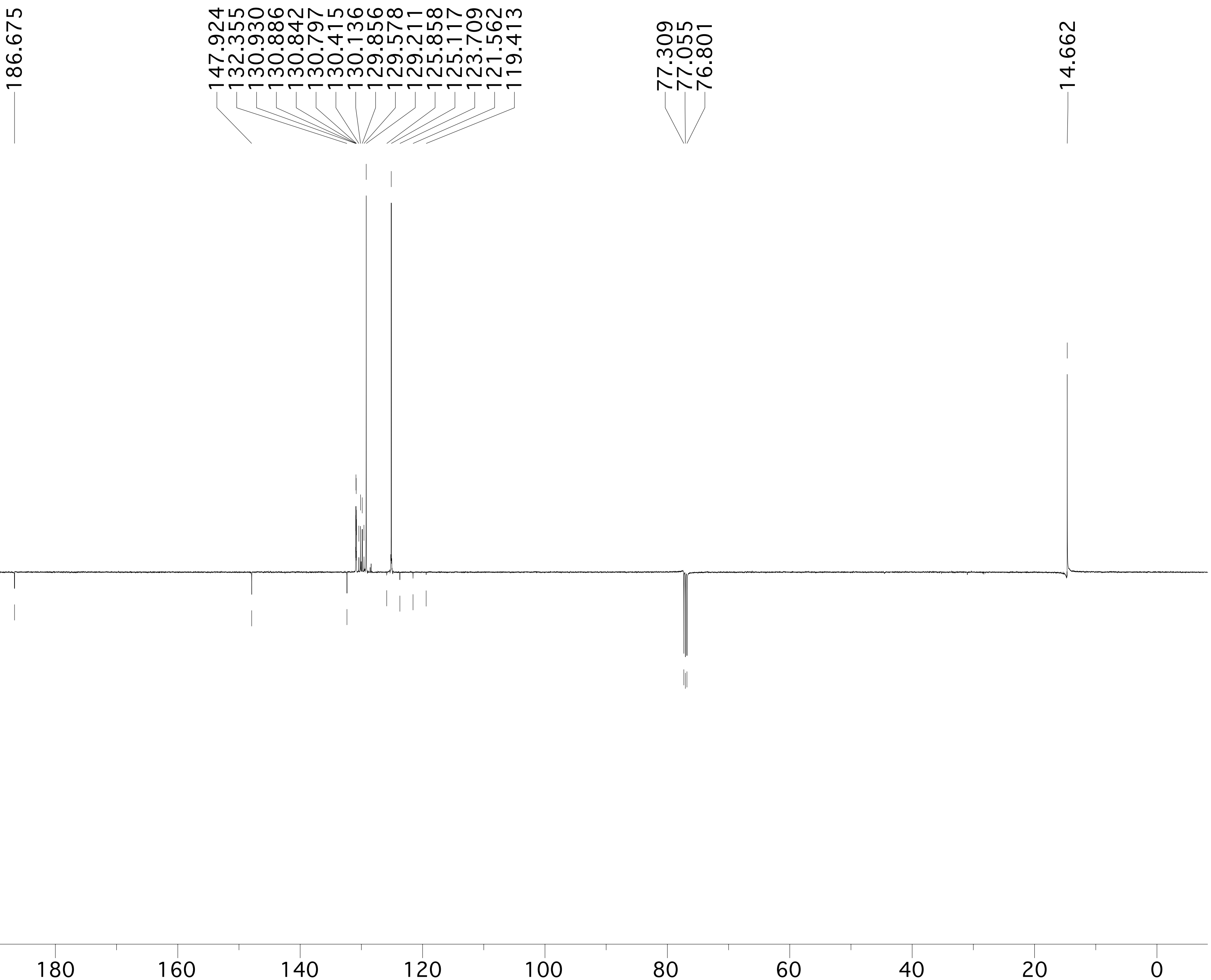

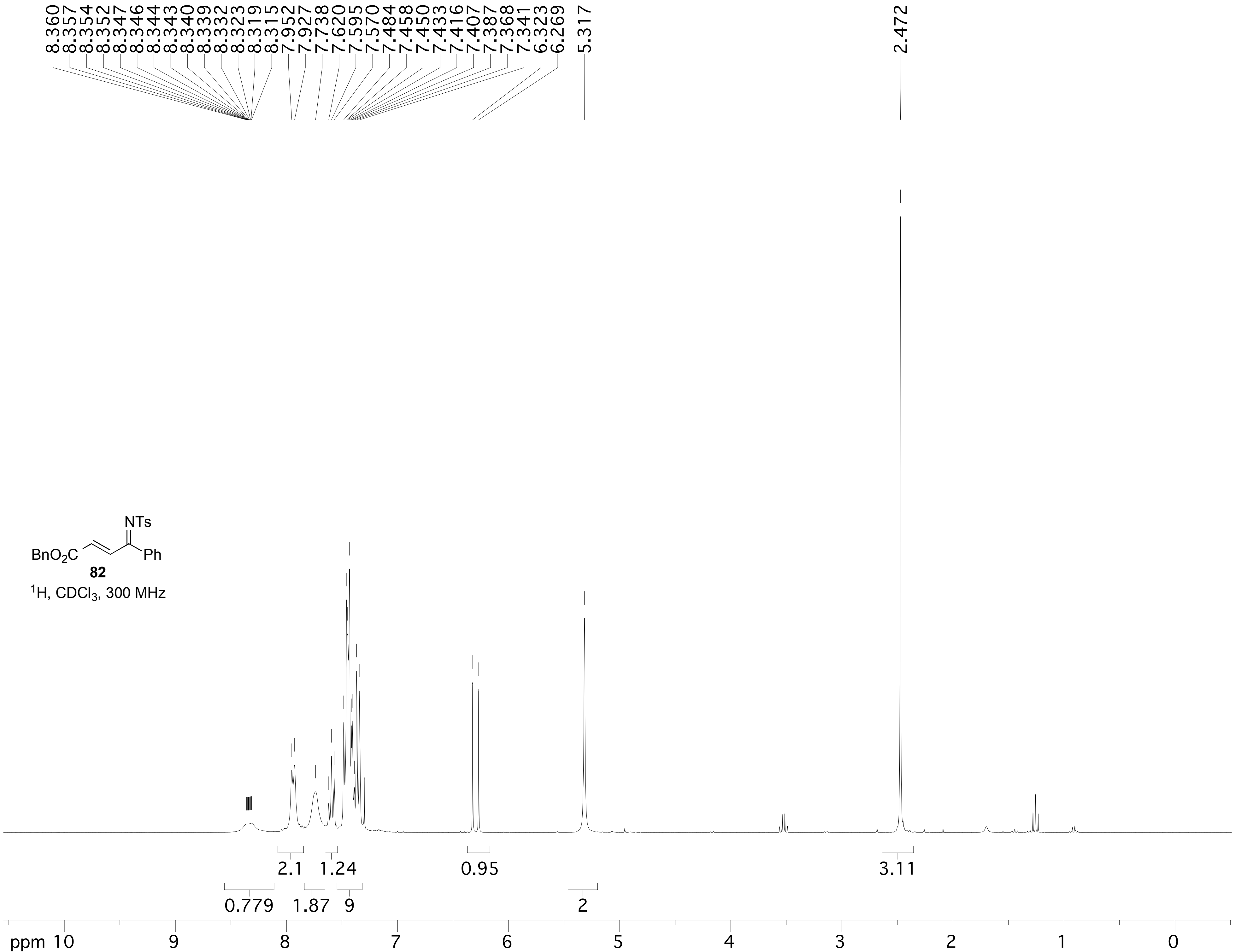
8.360
8.357
8.354
8.352
8.347
8.346
8.344
8.343
8.340
8.339
8.332
8.323
8.319
8.315
7.952
7.927
7.738
7.620
7.595
7.570
7.484
7.458
7.450
7.433
7.416
7.407
7.387
7.368
7.341
6.323
6.269
5.317
2.472
NTs
BnO2C
Ph
82
1H, CDCl3, 300 MHz
2.1
1.24
0.95
3.11
0.779
1.87
9
2
ppm 10
9
8
7
6
5
4
3
2
1
0

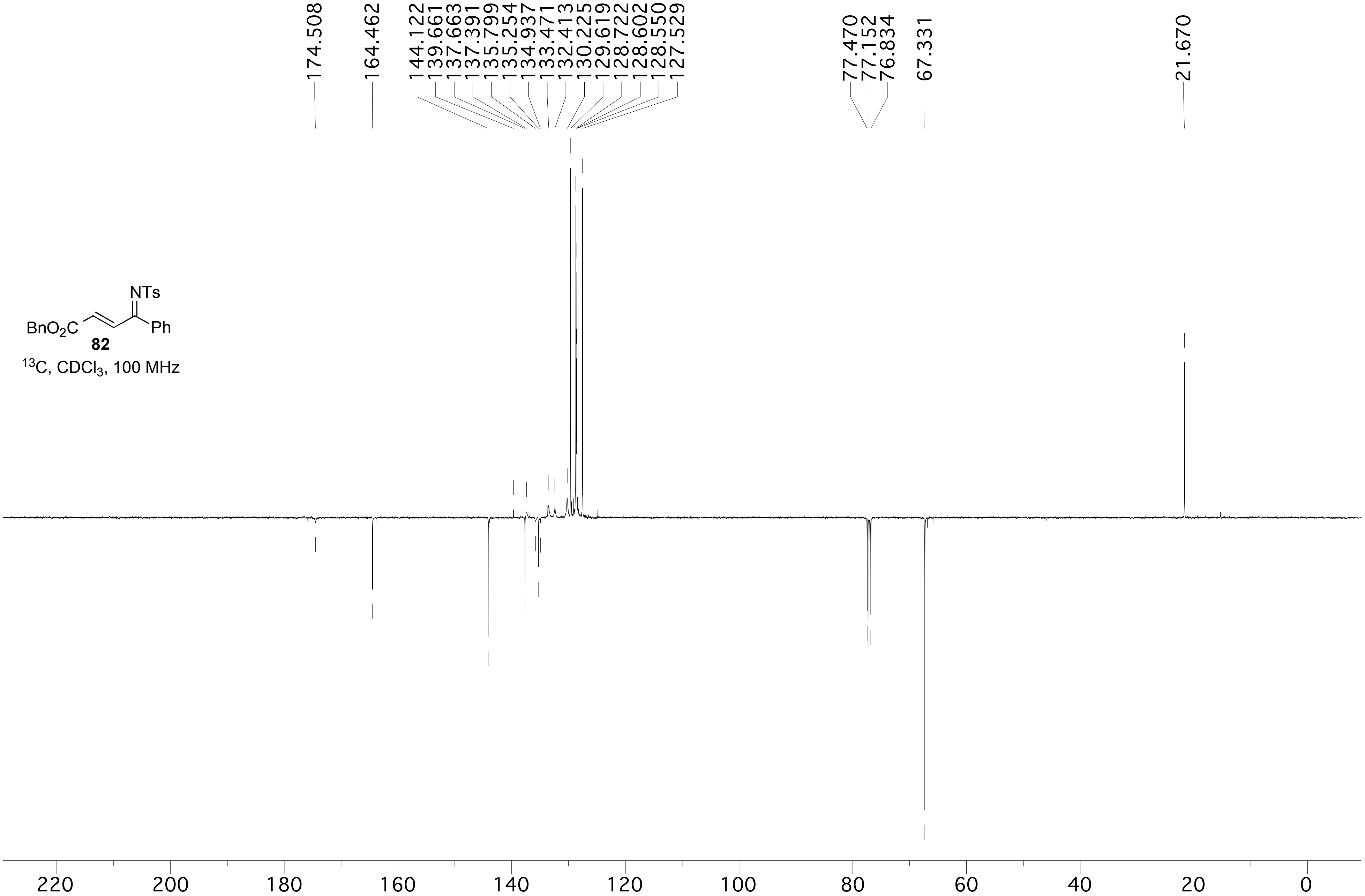

82

^{13}C, $CDCl_3$, 100 MHz

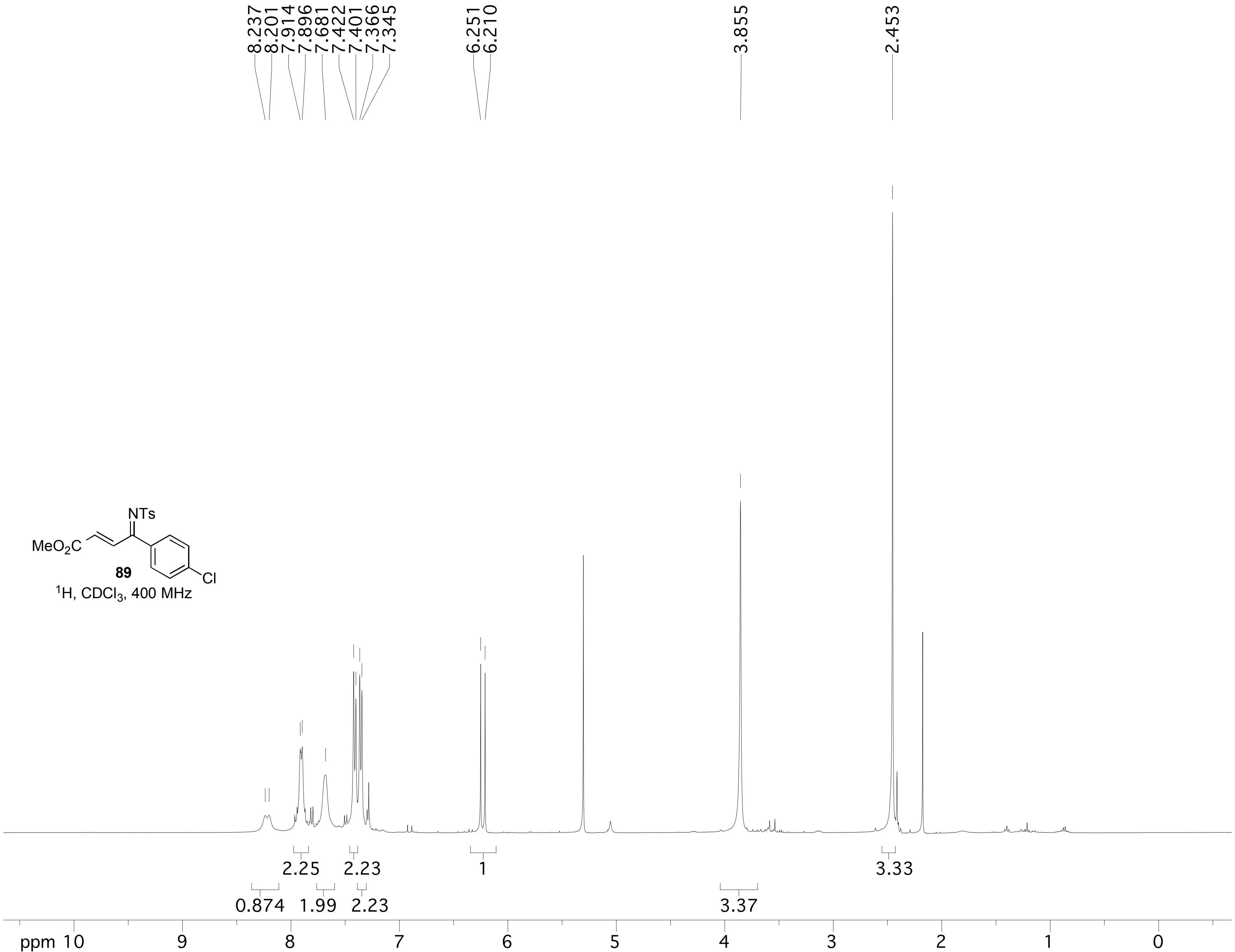
8.237
8.201
7.914
7.896
7.681
7.422
7.401
7.366
7.345
6.251
6.210
3.855
2.453
NTs
MeO2C
Cl
89
1H, CDCl3, 400 MHz
2.25
2.23
1
3.33
0.874
1.99
2.23
3.37
ppm 10
9
8
7
6
5
4
3
2
1
0

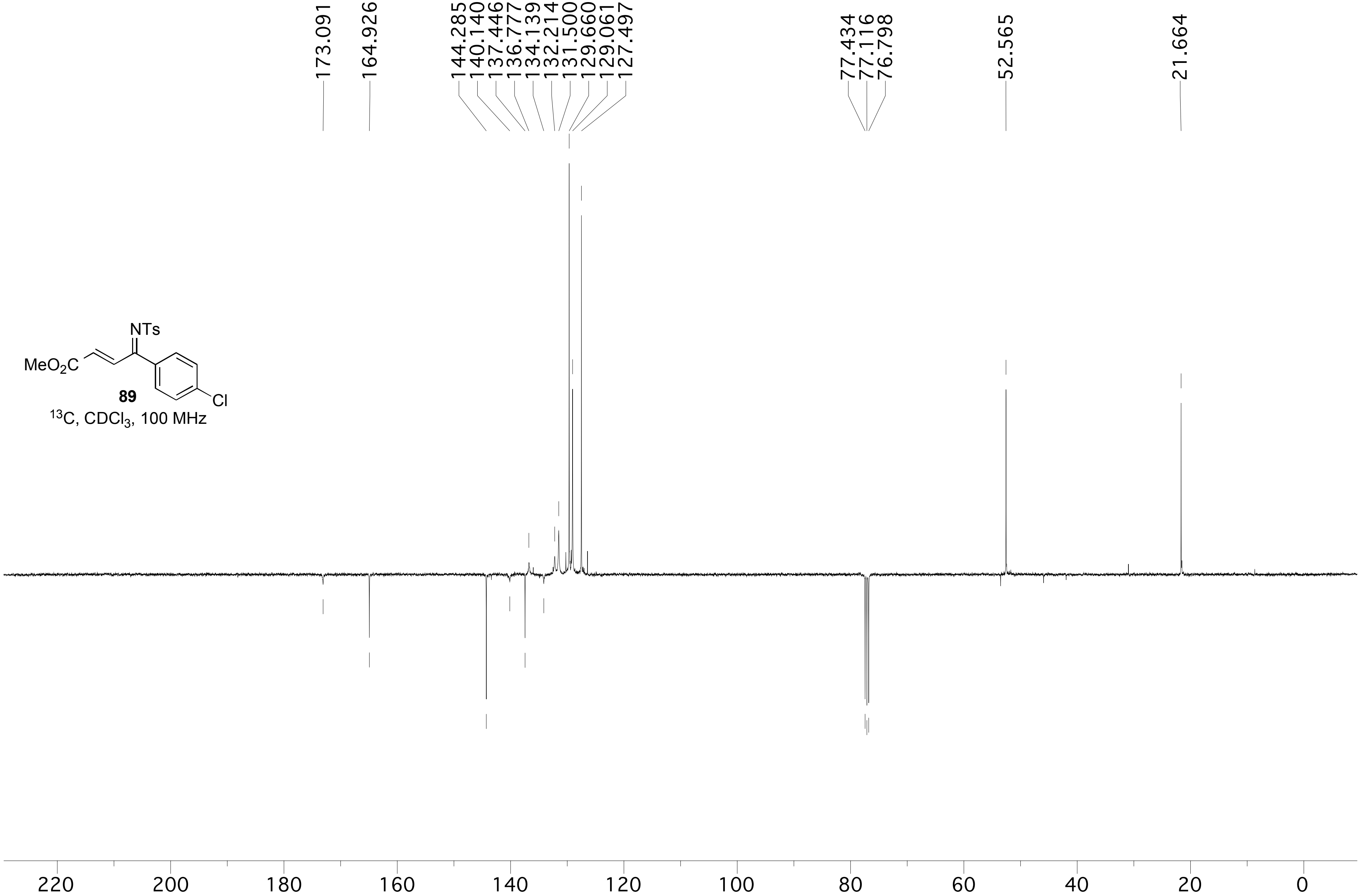
173.091
164.926
144.285
140.140
137.446
136.777
134.139
132.214
131.500
129.660
129.061
127.497
77.434
77.116
76.798
52.565
21.664
NTs
MeO2C
Cl
89
13C, CDCl3, 100 MHz
220
200
180
160
140
120
100
80
60
40
20
0

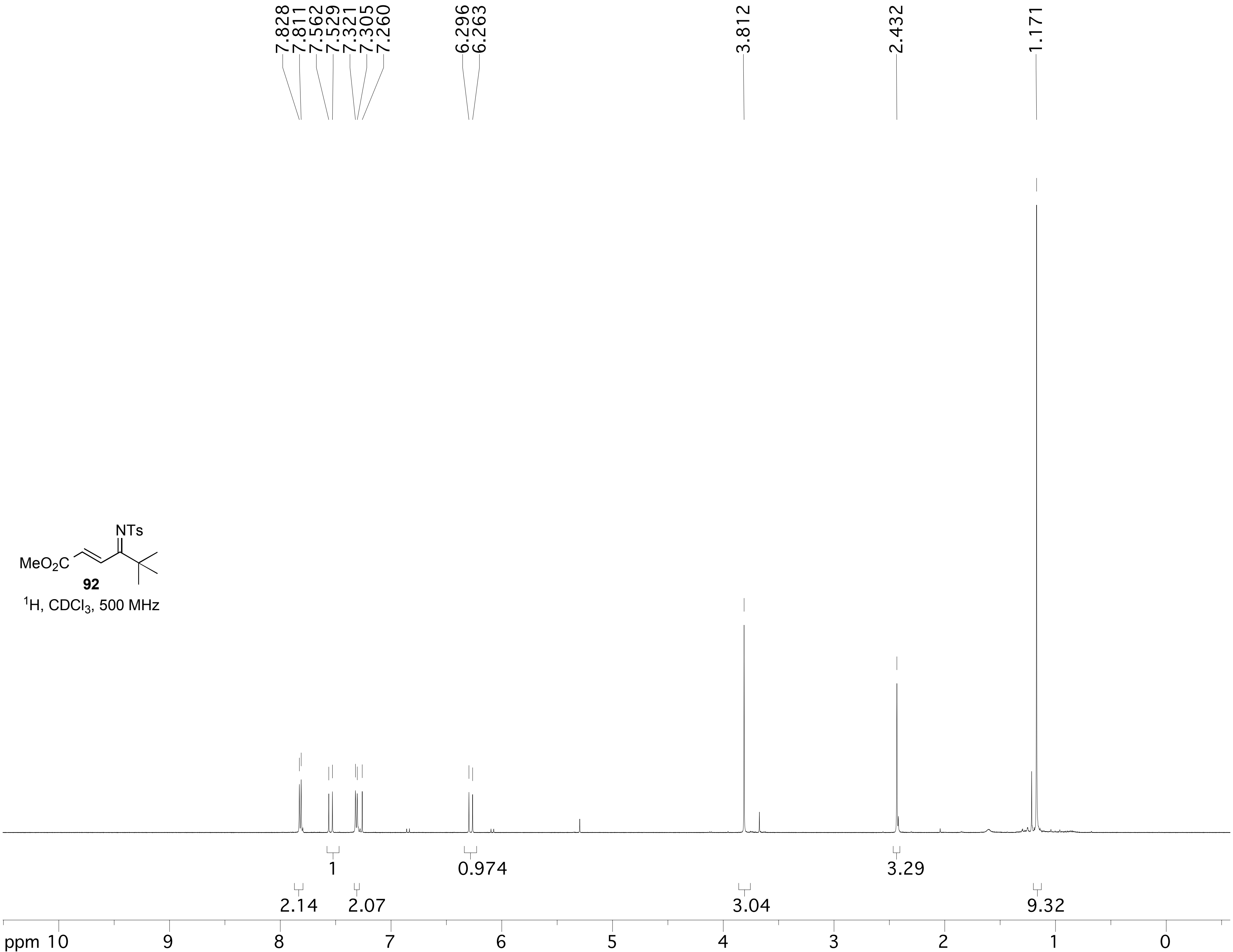
7.828
7.811
7.562
7.529
7.321
7.305
7.260
6.296
6.263
3.812
2.432
1.171
NTs
MeO2C
92
1H, CDCl3, 500 MHz
1
0.974
3.29
2.14
2.07
3.04
9.32
ppm 10
9
8
7
6
5
4
3
2
1
0

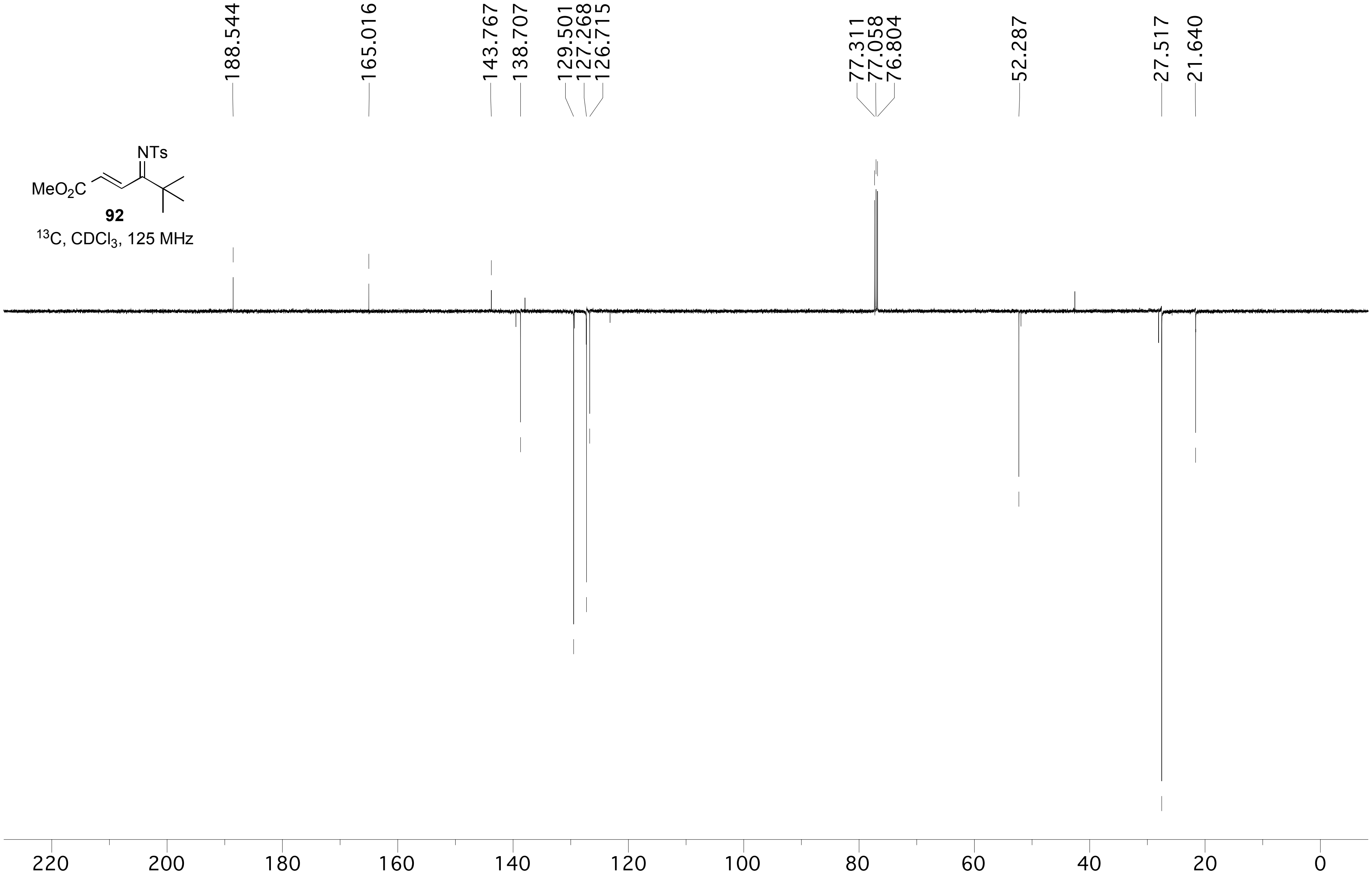
188.544
165.016
143.767
138.707
129.501
127.268
126.715
77.311
77.058
76.804
52.287
27.517
21.640
NTs
MeO2C
92
13C, CDCl3, 125 MHz
220
200
180
160
140
120
100
80
60
40
20
0

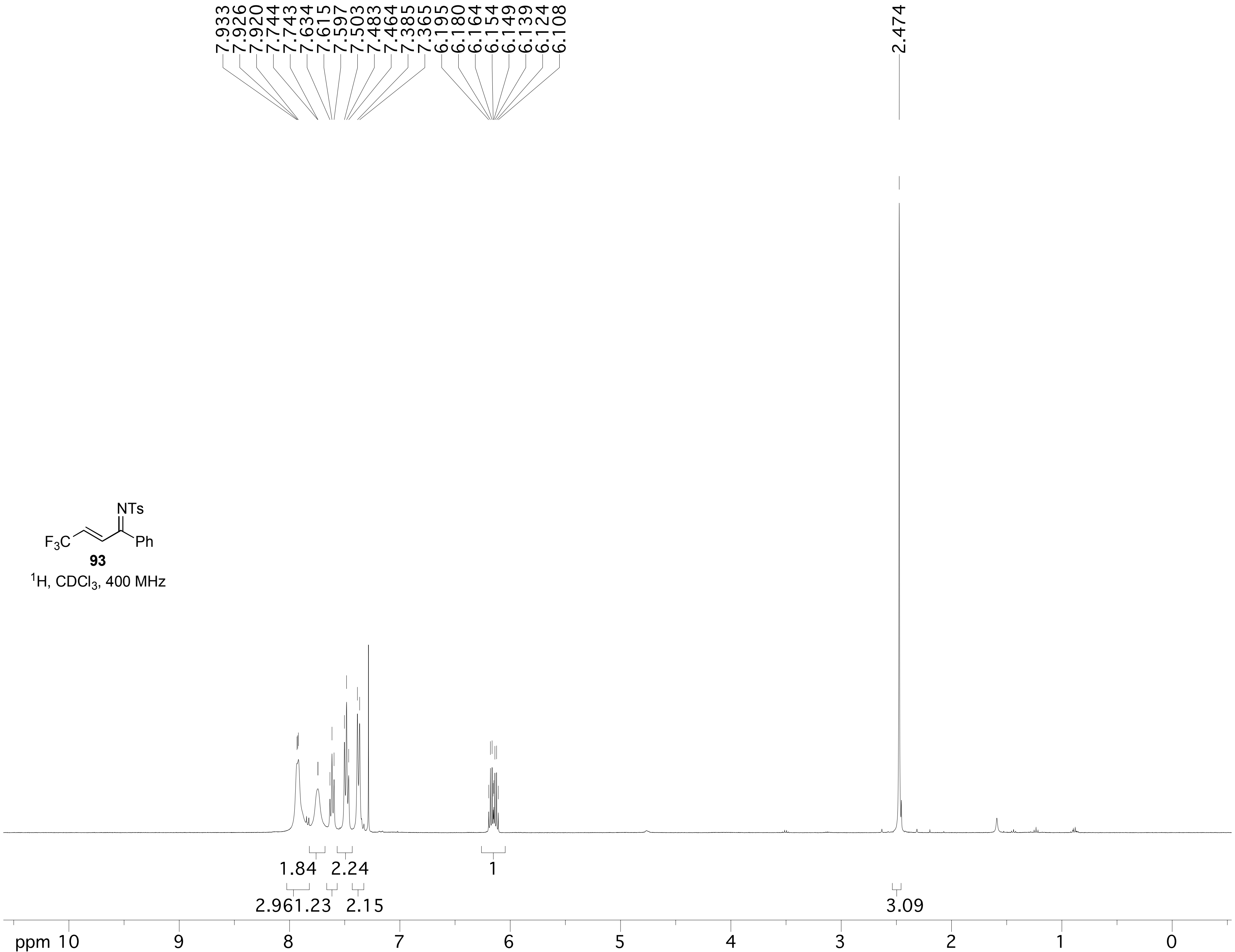
7.933 7.926 7.920 7.744 7.743 7.634 7.615 7.597 7.503 7.483 7.464 7.385 7.365 6.195 6.180 6.164 6.154 6.149 6.139 6.124 6.108
2.474
NTs
F3C
Ph
93
1H, CDCl3, 400 MHz
1.84 2.24
1
2.96 1.23 2.15
3.09
ppm 10 9 8 7 6 5 4 3 2 1 0

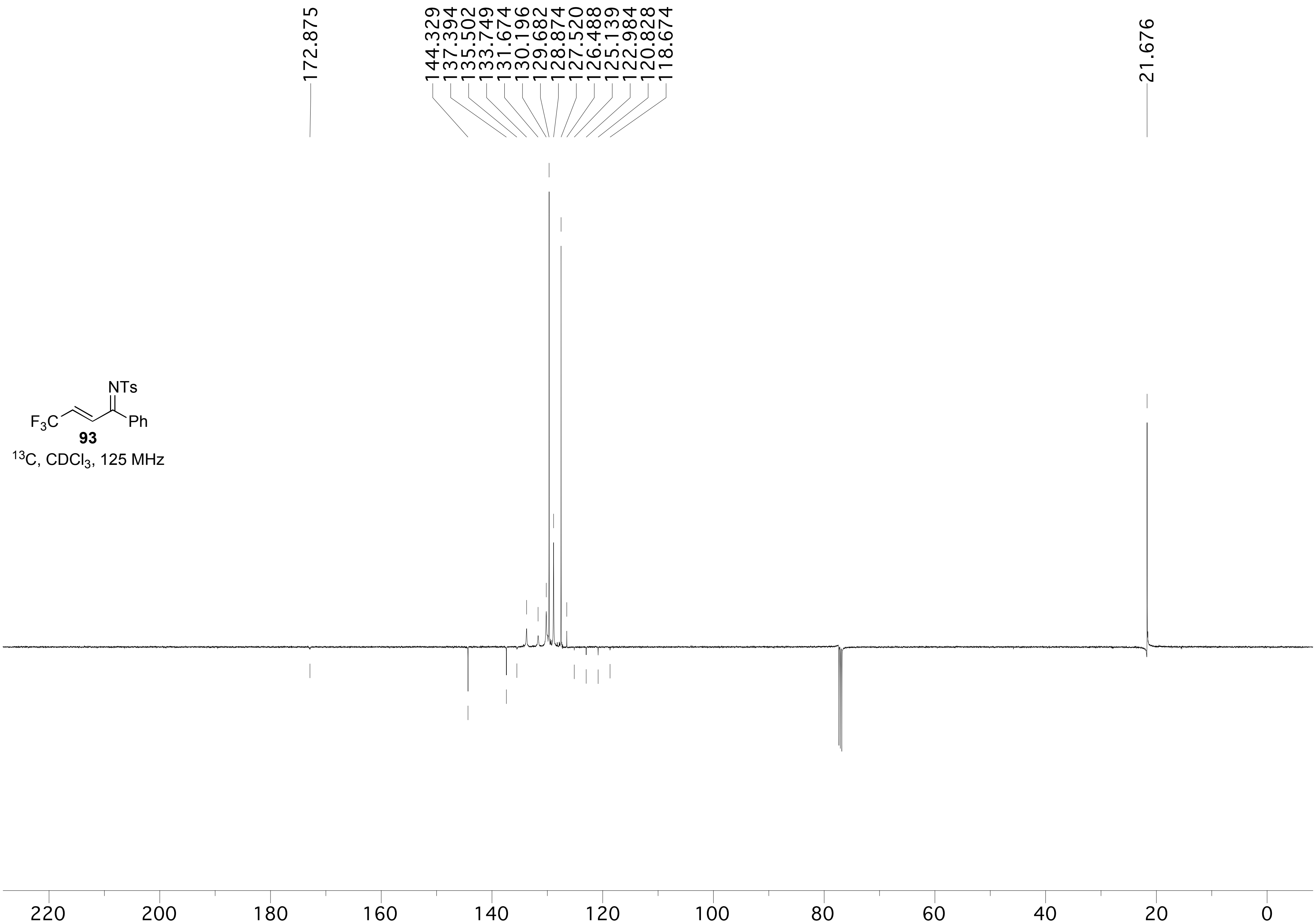

172.875
144.329
137.394
135.502
133.749
131.674
130.196
129.682
128.874
127.520
126.488
125.139
122.984
120.828
118.674
21.676
NTs
F3C
Ph
93
13C, CDCl3, 125 MHz
220
200
180
160
140
120
100
80
60
40
20
0

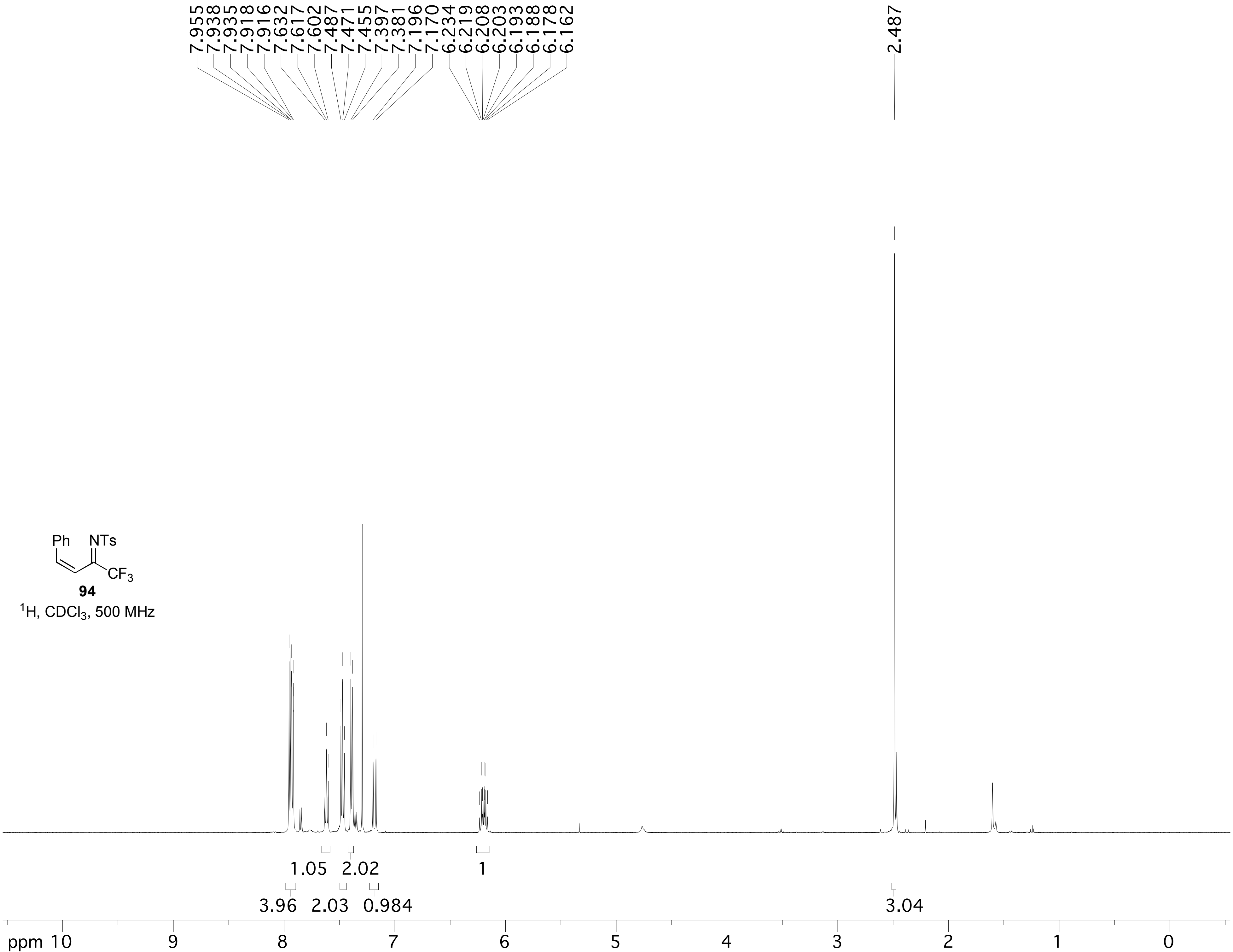
7.955
7.938
7.935
7.918
7.916
7.632
7.617
7.602
7.487
7.471
7.455
7.397
7.381
7.196
7.170
6.234
6.219
6.208
6.203
6.193
6.188
6.178
6.162
2.487
Ph
NTs
CF3
94
1H, CDCl3, 500 MHz
1.05
2.02
1
3.96
2.03
0.984
3.04
ppm 10
9
8
7
6
5
4
3
2
1
0

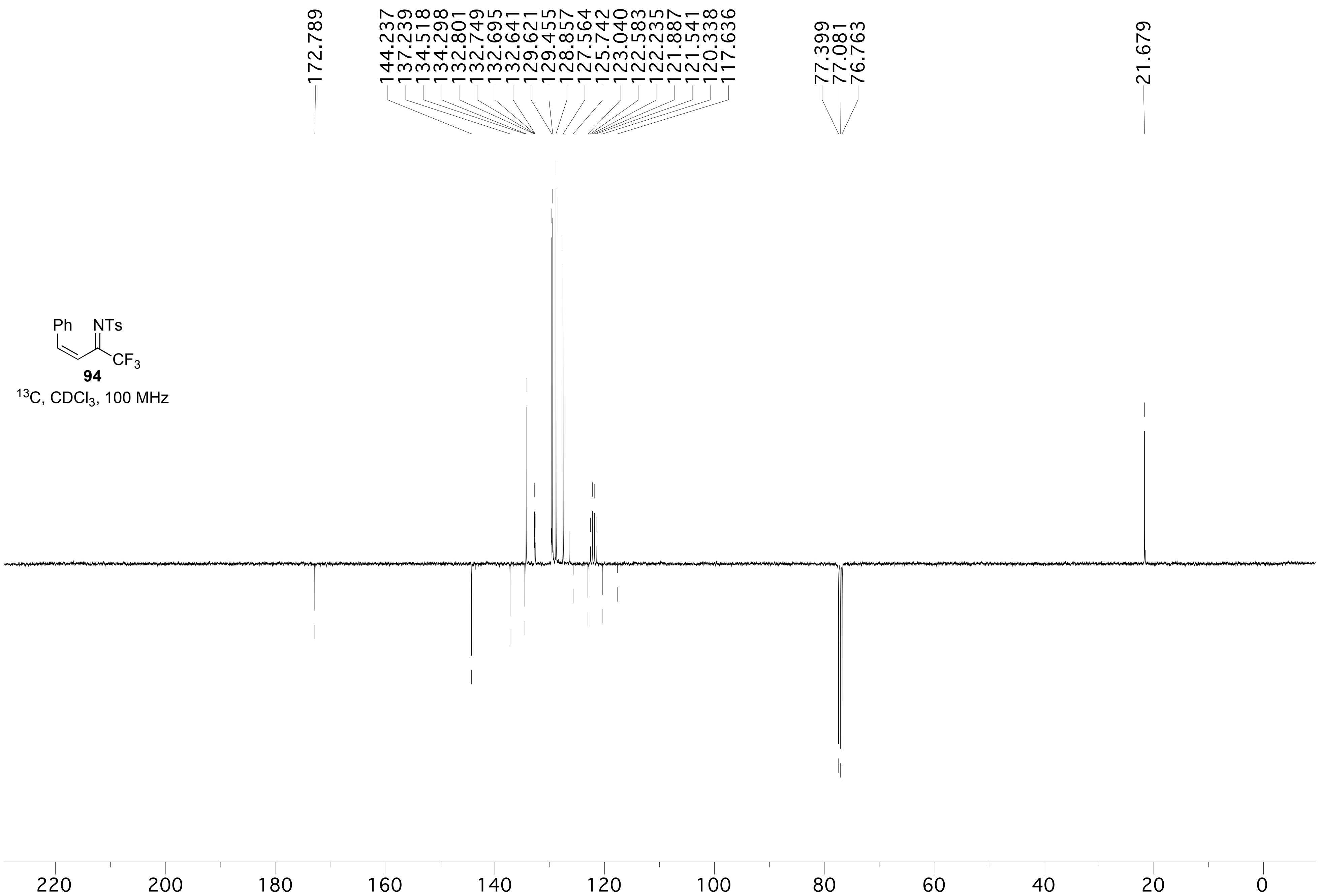

172.789
144.237
137.239
134.518
134.298
132.801
132.749
132.695
132.641
129.621
129.455
128.857
127.564
125.742
123.040
122.583
122.235
121.887
121.541
120.338
117.636
77.399
77.081
76.763
21.679
Ph
NTs
CF3
94
13C, CDCl3, 100 MHz
220
200
180
160
140
120
100
80
60
40
20
0

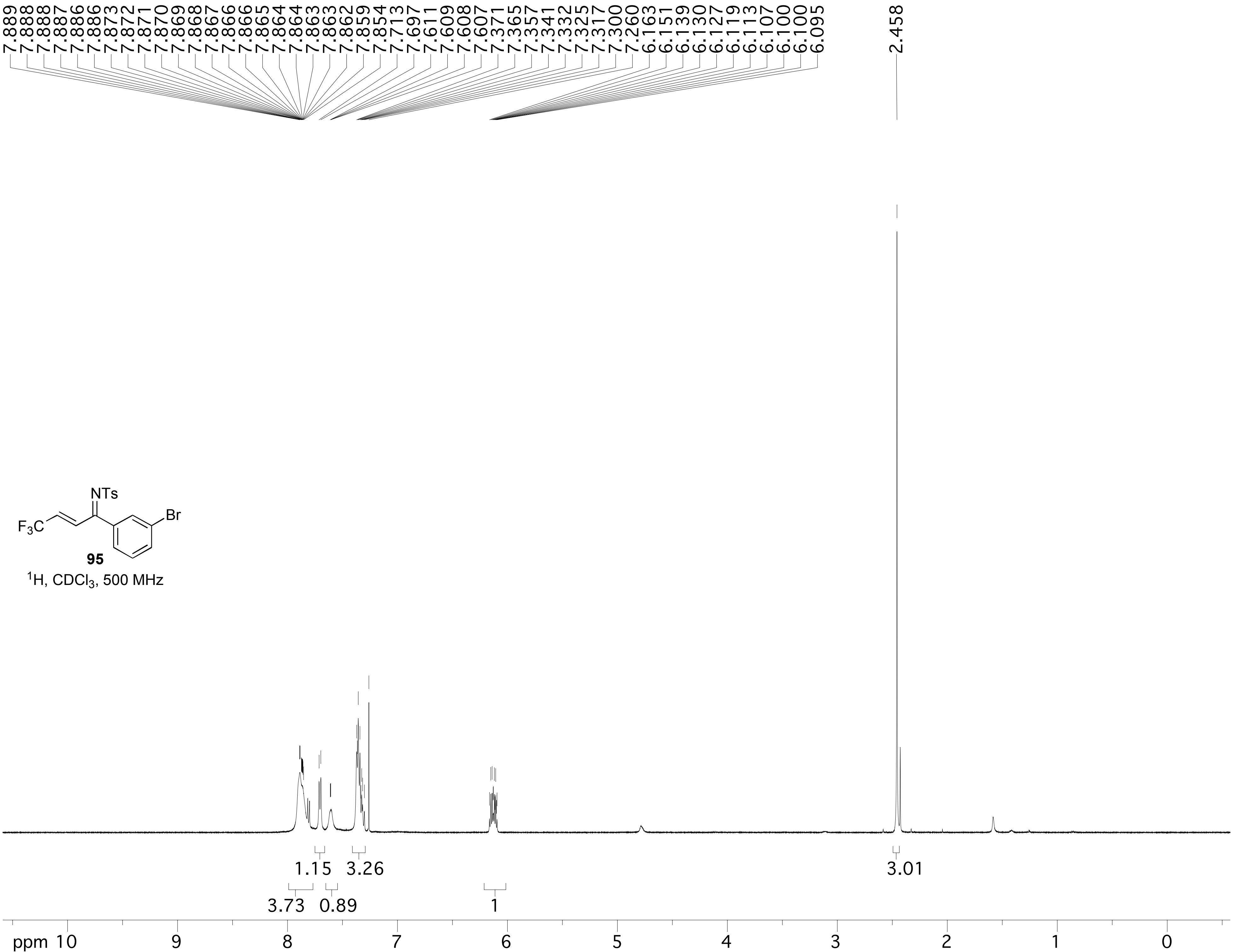

7.889
7.888
7.888
7.887
7.886
7.886
7.873
7.872
7.871
7.870
7.869
7.868
7.867
7.866
7.866
7.865
7.864
7.864
7.863
7.863
7.862
7.859
7.854
7.713
7.697
7.611
7.609
7.608
7.607
7.371
7.365
7.357
7.341
7.332
7.325
7.317
7.300
7.260
6.163
6.151
6.139
6.130
6.127
6.119
6.113
6.107
6.100
6.100
6.095
2.458
NTs
Br
F3C
95
1H, CDCl3, 500 MHz
1.15
3.26
3.01
3.73
0.89
1
ppm 10
9
8
7
6
5
4
3
2
1
0

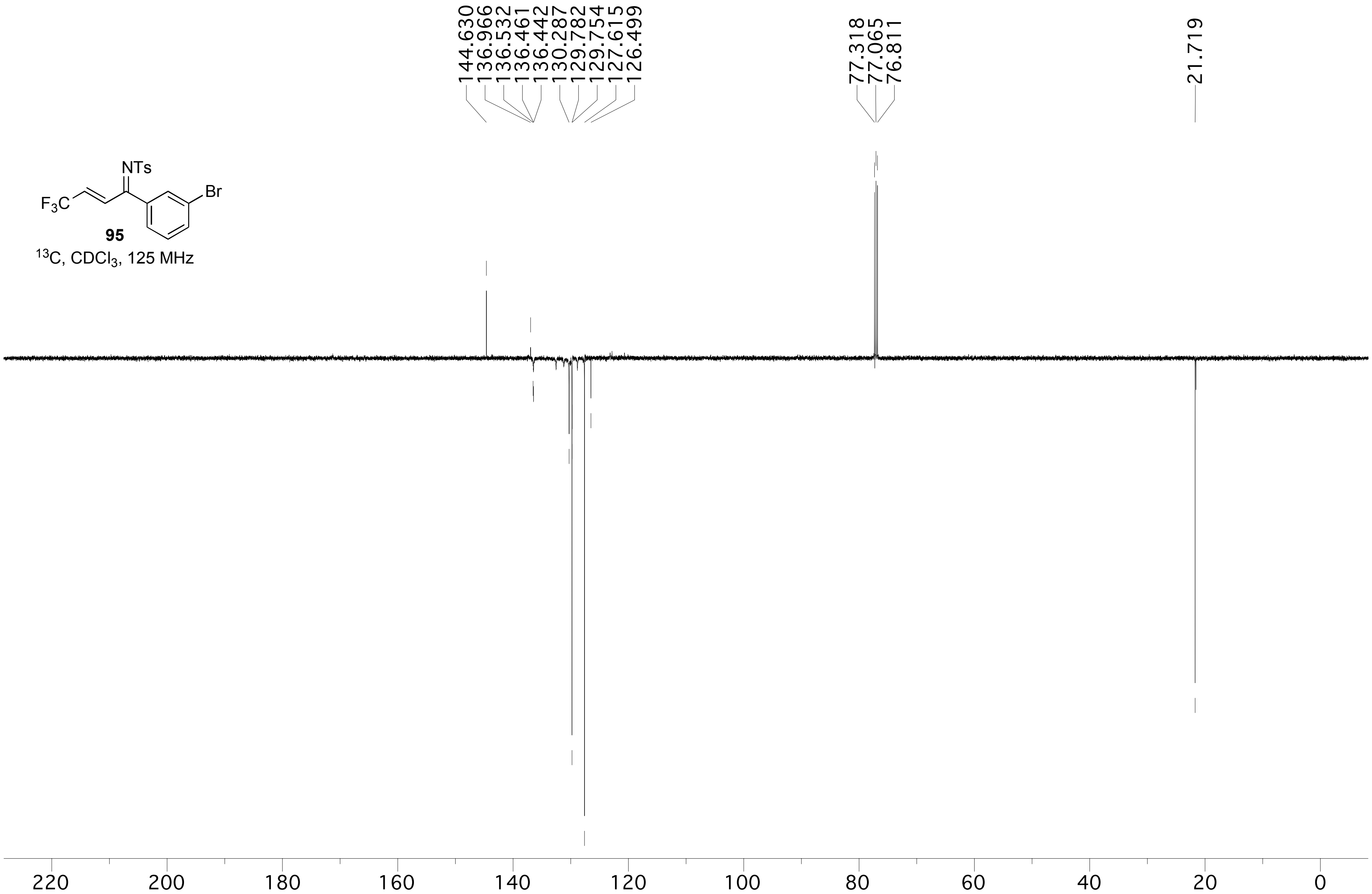

NTs
F_3C
Br
95
^{13}C, $CDCl_3$, 125 MHz
144.630
136.966
136.532
136.461
136.442
130.287
129.782
129.754
127.615
126.499
77.318
77.065
76.811
21.719
220
200
180
160
140
120
100
80
60
40
20
0

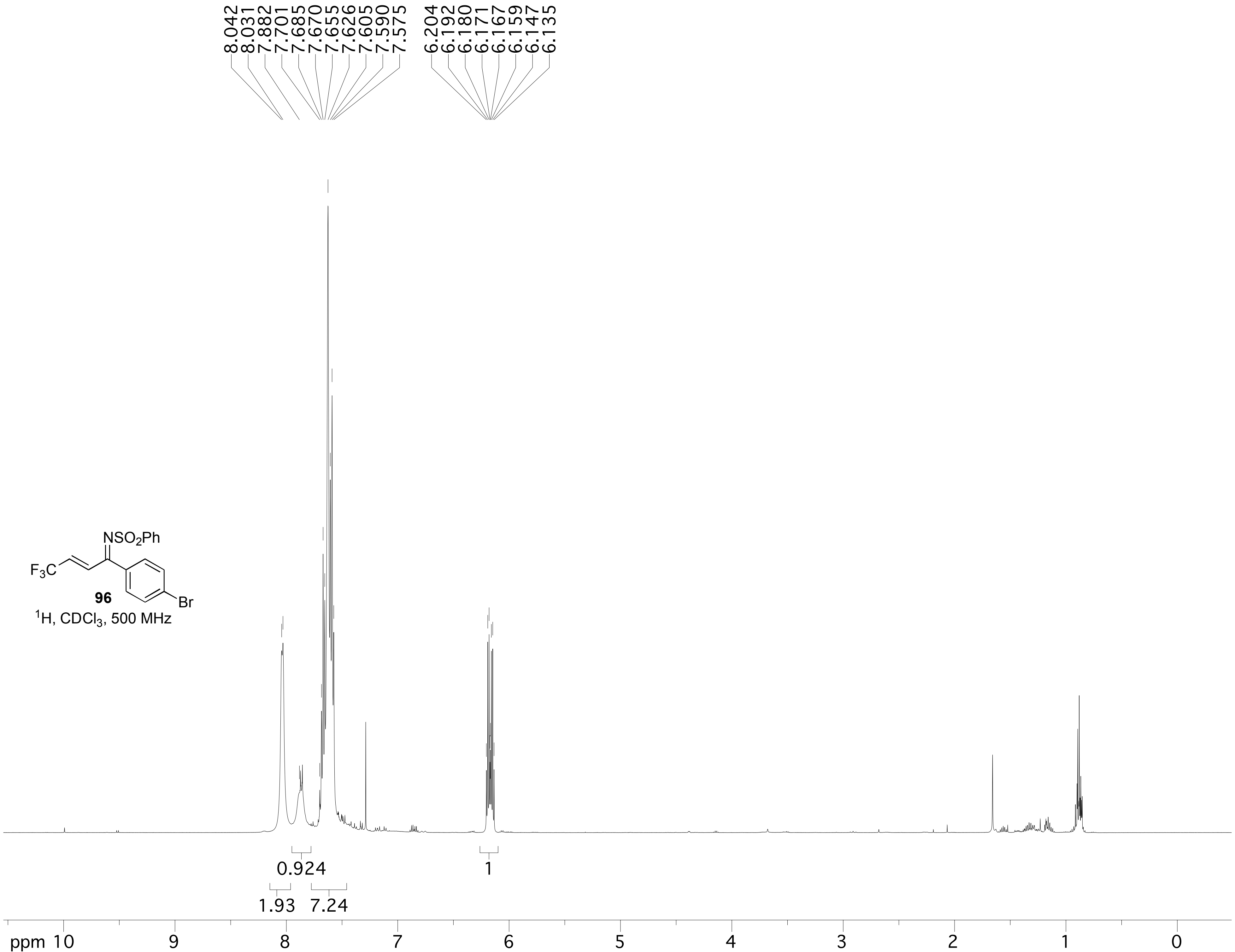
8.042
8.031
7.882
7.701
7.685
7.670
7.655
7.626
7.605
7.590
7.575
6.204
6.192
6.180
6.171
6.167
6.159
6.147
6.135
NSO2Ph
F3C
Br
96
1H, CDCl3, 500 MHz
0.924
1
1.93
7.24
ppm 10
9
8
7
6
5
4
3
2
1
0

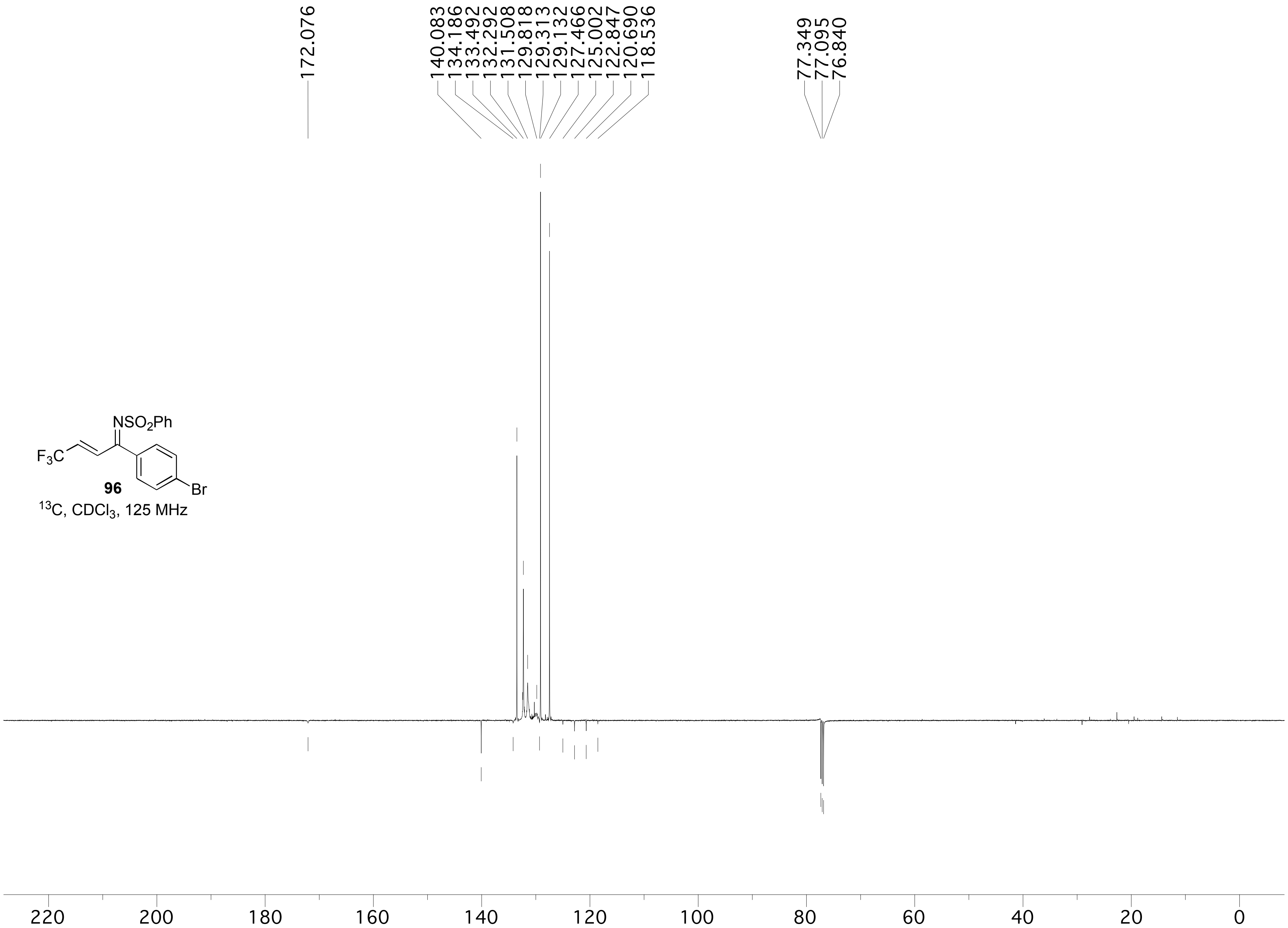
172.076
140.083
134.186
133.492
132.292
131.508
129.818
129.313
129.132
127.466
125.002
122.847
120.690
118.536
77.349
77.095
76.840
NSO2Ph
F3C
96
Br
13C, CDCl3, 125 MHz
220
200
180
160
140
120
100
80
60
40
20
0

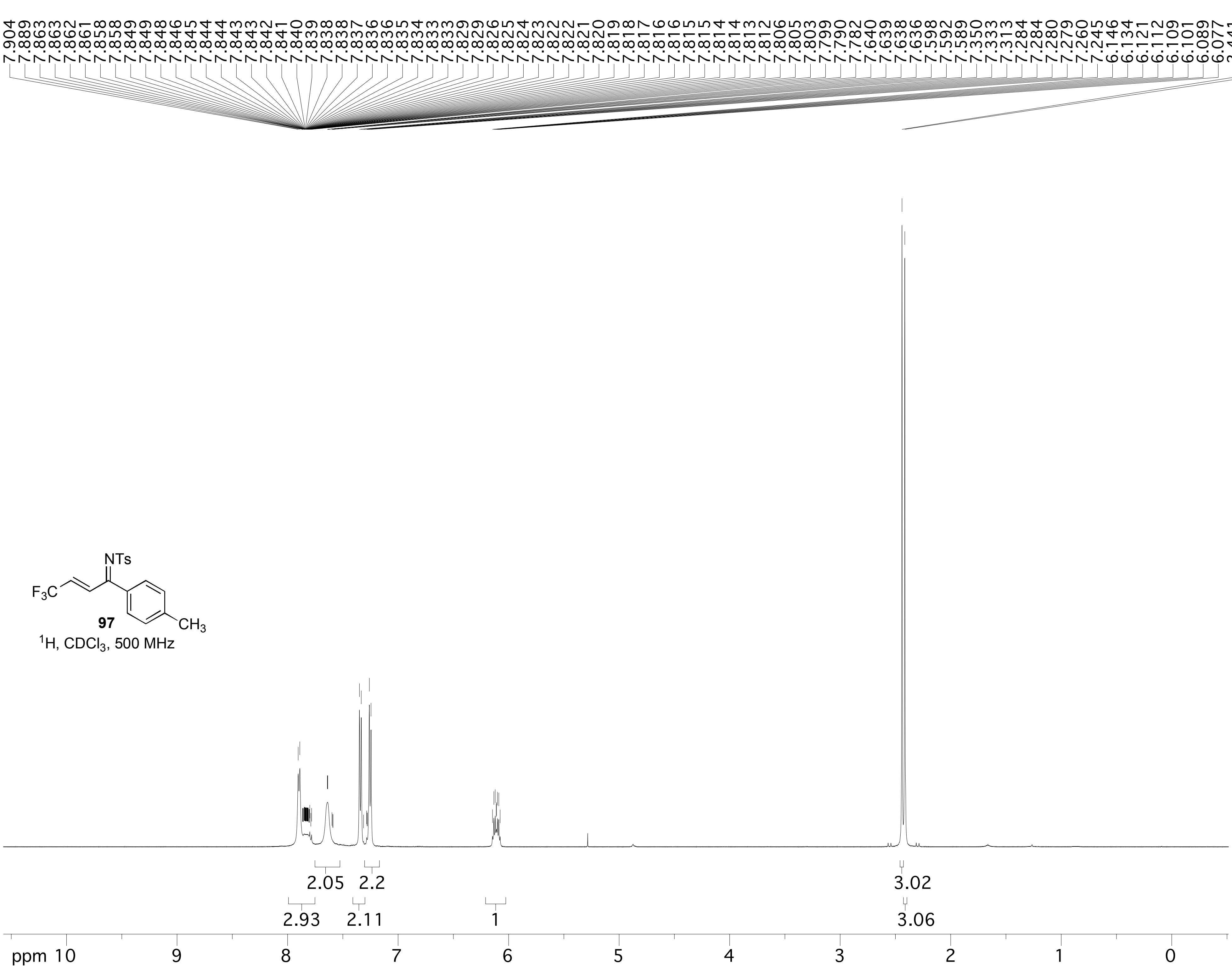

7.904 7.889 7.863 7.862 7.861 7.858 7.858 7.849 7.848 7.846 7.845 7.844 7.843 7.843 7.842 7.841 7.840 7.839 7.838 7.838 7.837 7.836 7.836 7.835 7.834 7.833 7.833 7.829 7.829 7.826 7.825 7.824 7.823 7.822 7.822 7.821 7.820 7.819 7.818 7.817 7.816 7.816 7.815 7.815 7.814 7.814 7.813 7.812 7.806 7.805 7.803 7.799 7.790 7.782 7.640 7.639 7.638 7.636 7.598 7.592 7.589 7.350 7.333 7.313 7.284 7.284 7.280 7.279 7.260 7.245 6.146 6.134 6.121 6.112 6.109 6.101 6.089 6.077
NTs
F_3C
CH_3
97
^1H, $CDCl_3$, 500 MHz
2.05
2.2
3.02
2.93
2.11
1
3.06
ppm 10 9 8 7 6 5 4 3 2 1 0

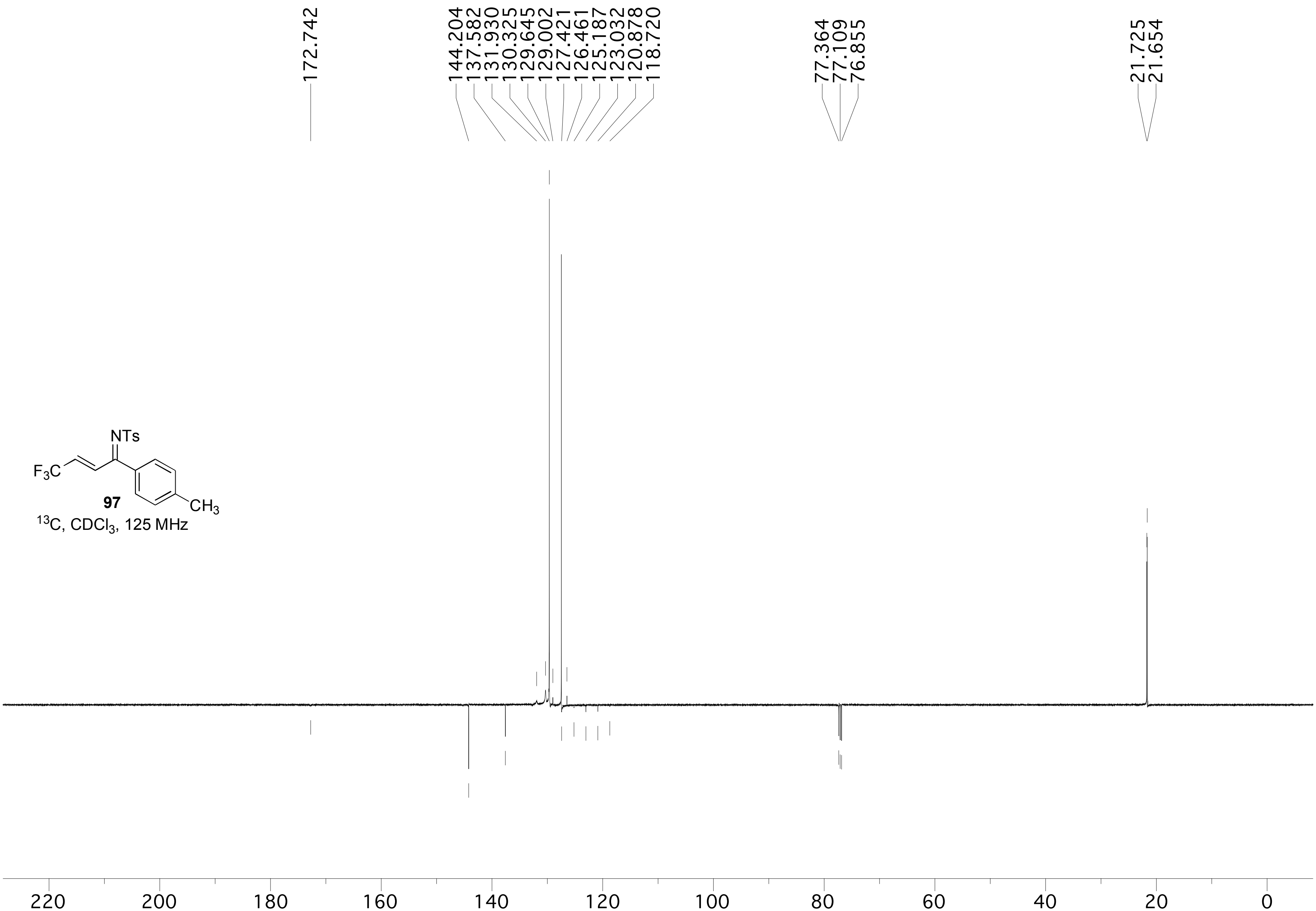
172.742
144.204
137.582
131.930
130.325
129.645
129.002
127.421
126.461
125.187
123.032
120.878
118.720
77.364
77.109
76.855
21.725
21.654
NTs
F3C
CH3
97
13C, CDCl3, 125 MHz
220
200
180
160
140
120
100
80
60
40
20
0

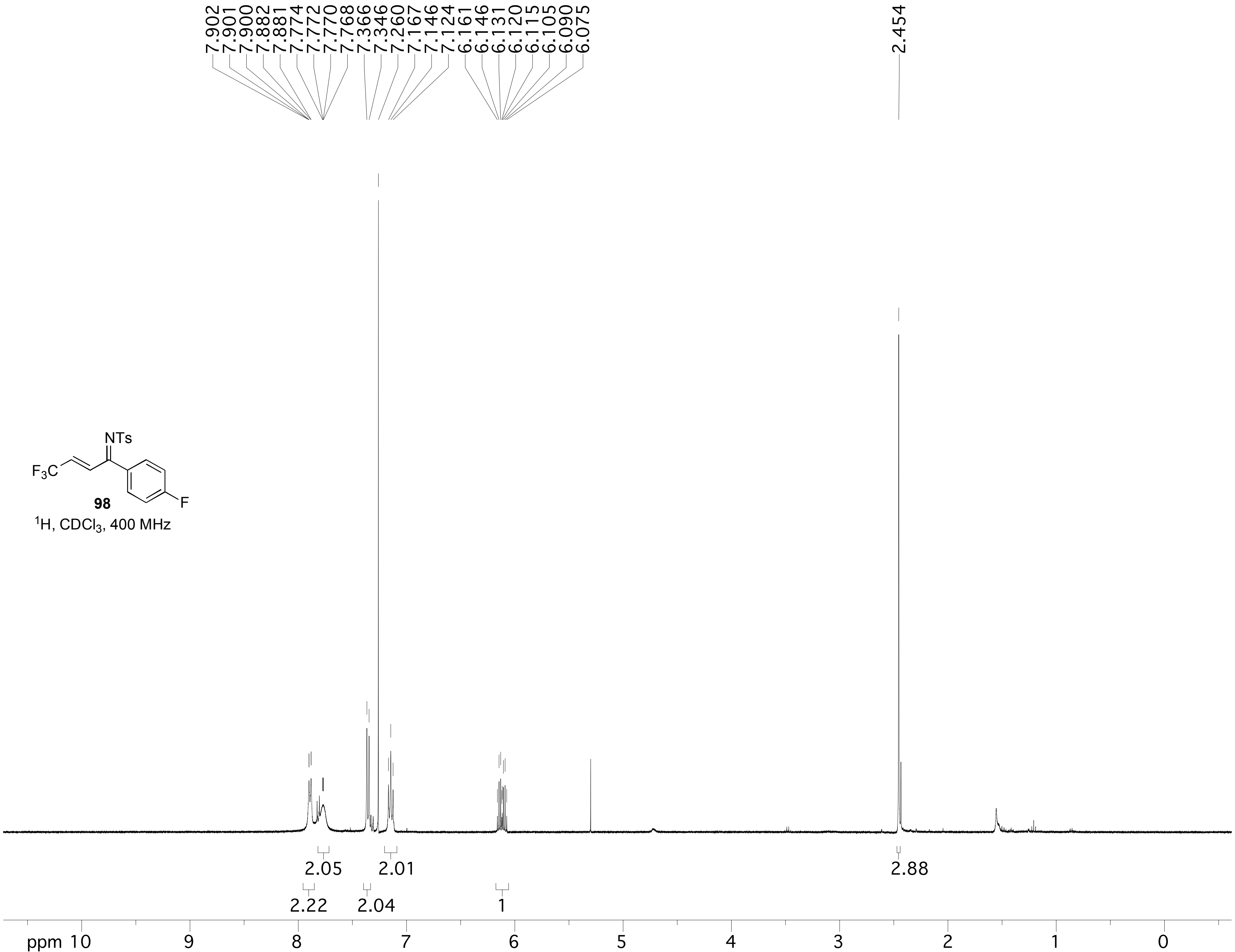

7.902
7.901
7.900
7.882
7.881
7.774
7.772
7.770
7.768
7.366
7.346
7.260
7.167
7.146
7.124
6.161
6.146
6.131
6.120
6.115
6.105
6.090
6.075
2.454
NTs
F3C
F
98
1H, CDCl3, 400 MHz
2.05
2.01
2.88
2.22
2.04
1
ppm 10
9
8
7
6
5
4
3
2
1
0

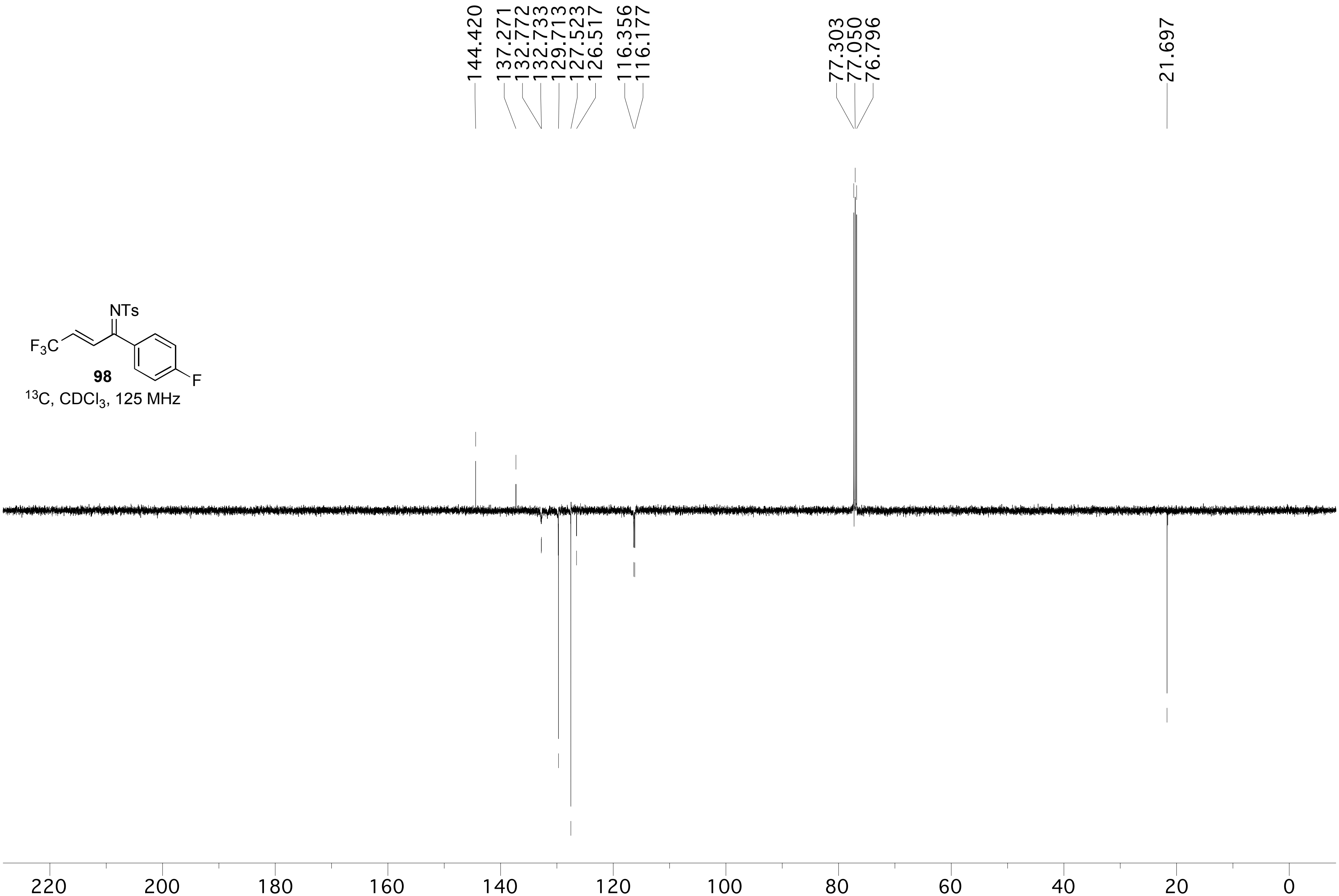

144.420
137.271
132.772
132.733
129.713
127.523
126.517
116.356
116.177
77.303
77.050
76.796
21.697
NTs
F_3C
98
F
^{13}C, $CDCl_3$, 125 MHz
220
200
180
160
140
120
100
80
60
40
20
0

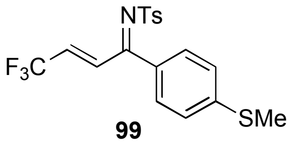

99

^1H, $CDCl_3$, 500 MHz

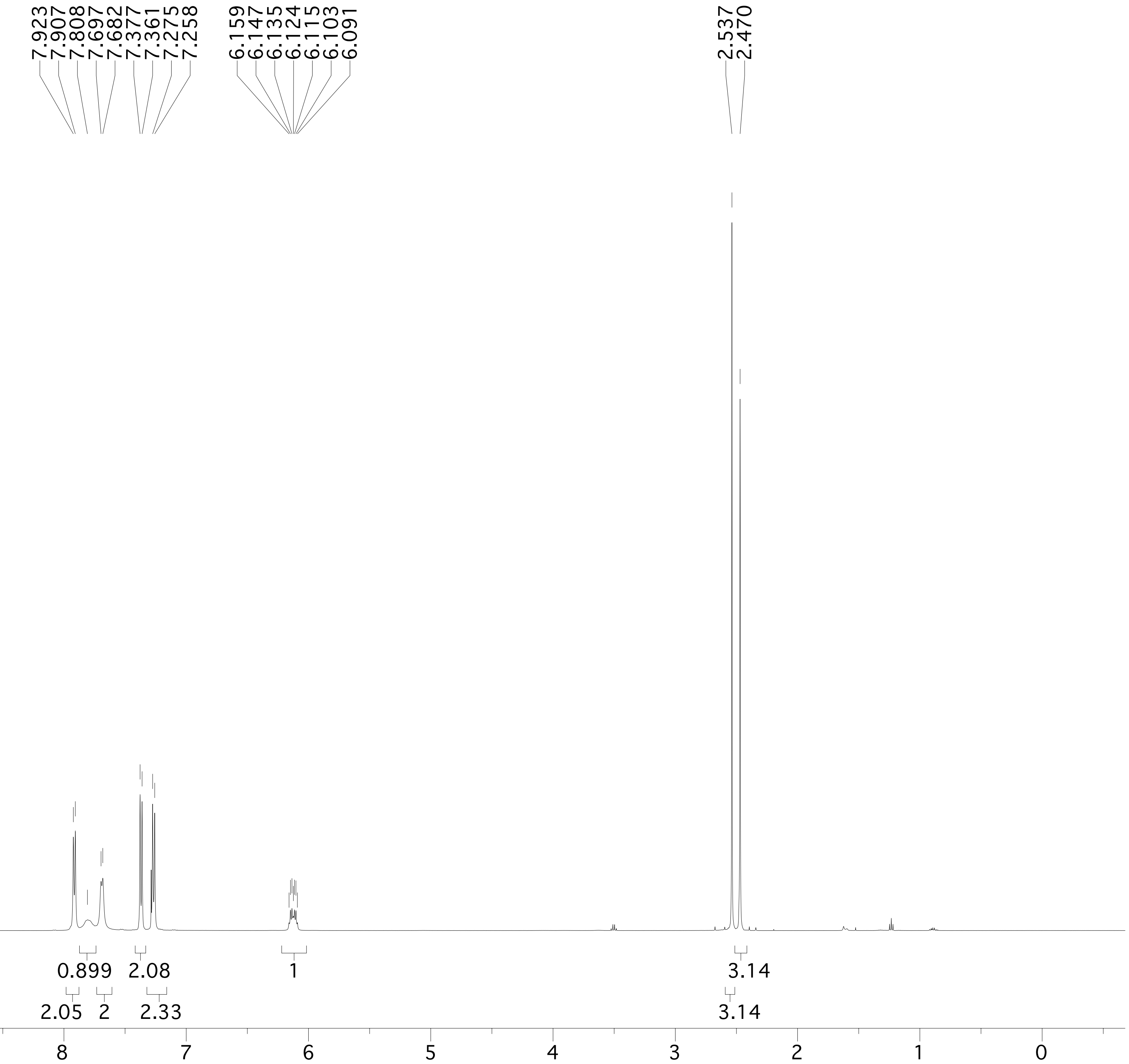

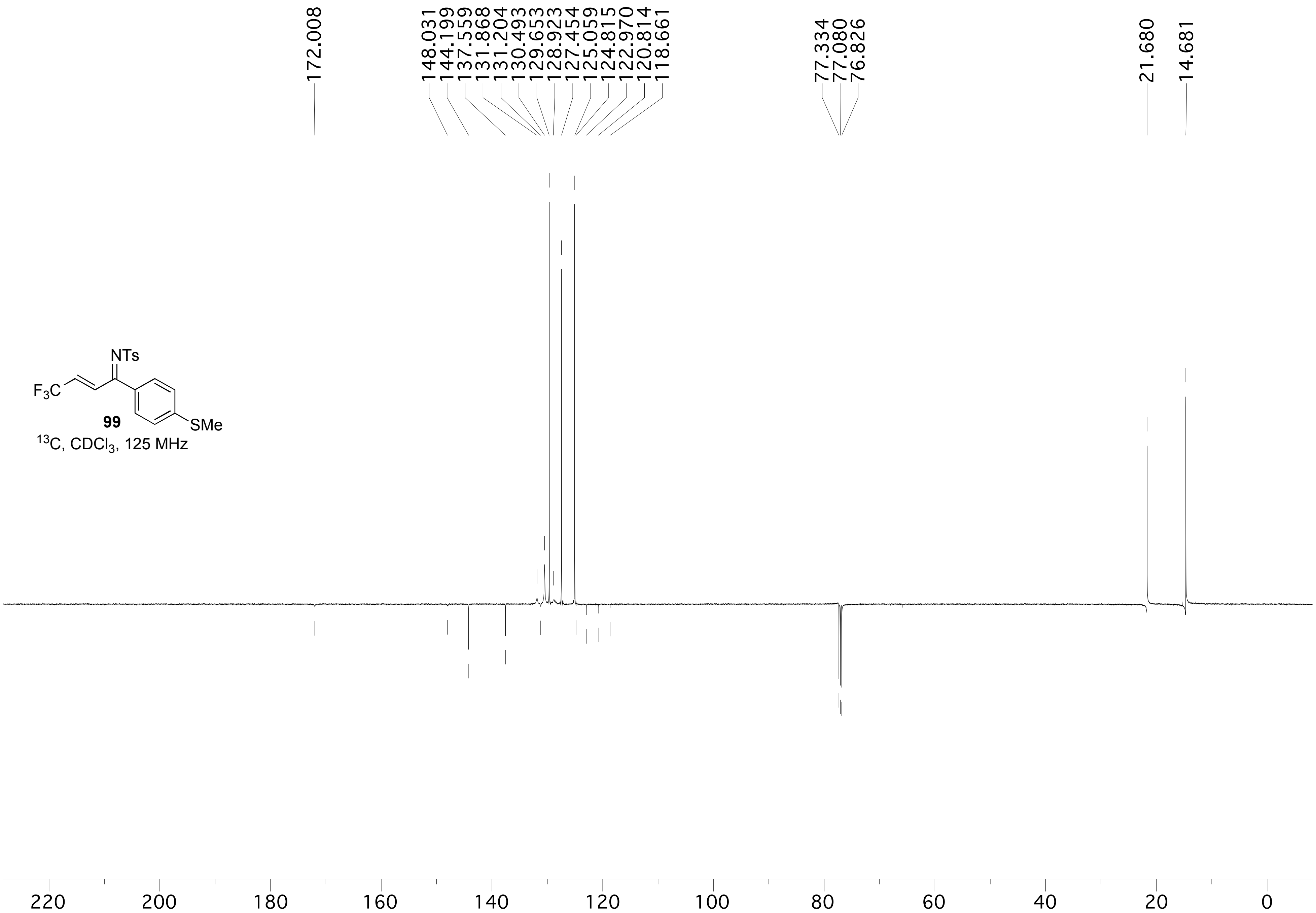
172.008
148.031
144.199
137.559
131.868
131.204
130.493
129.653
128.923
127.454
125.059
124.815
122.970
120.814
118.661
77.334
77.080
76.826
21.680
14.681
NTs
F3C
SMe
99
13C, CDCl3, 125 MHz
220
200
180
160
140
120
100
80
60
40
20
0

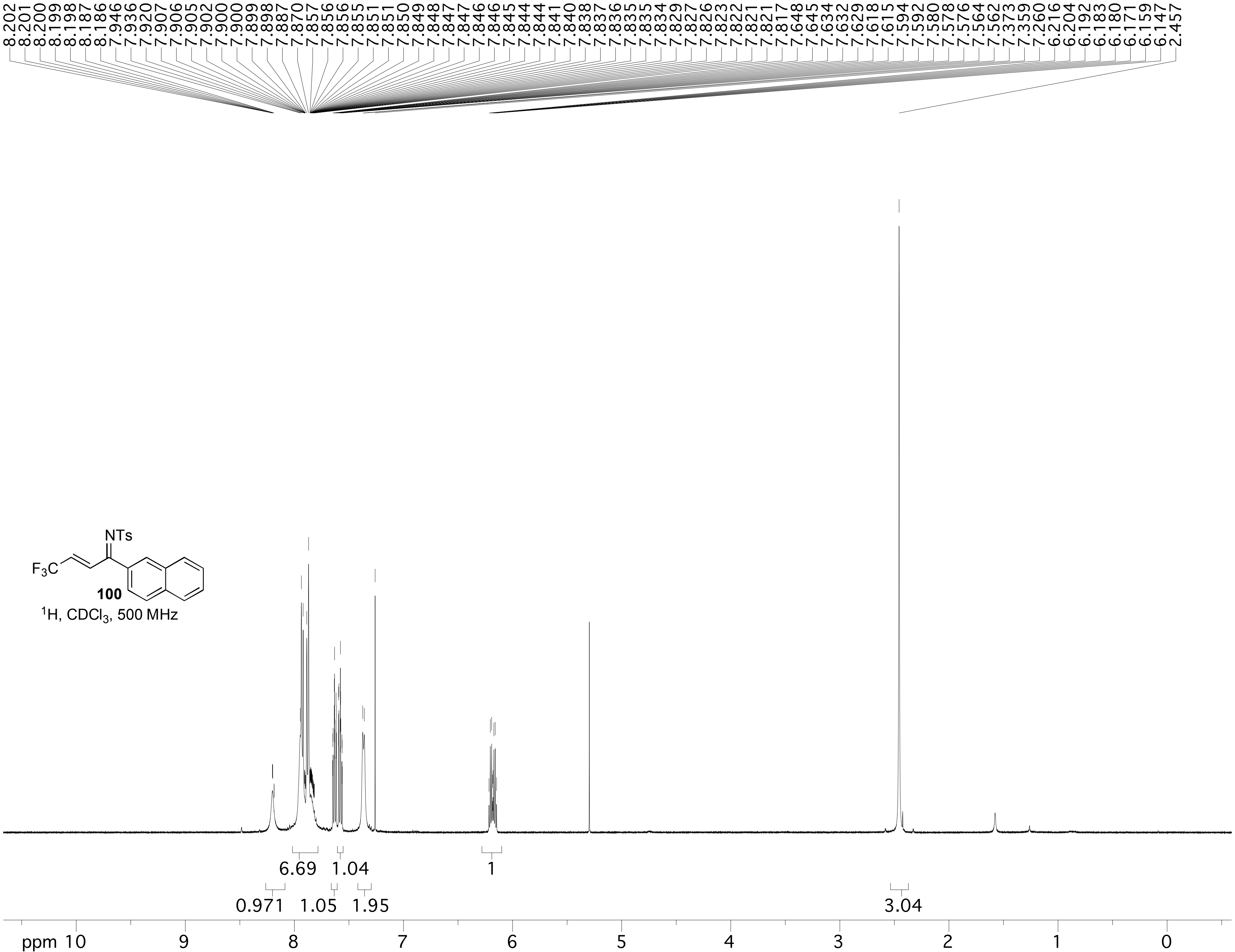
8.202
8.201
8.200
8.199
8.198
8.187
8.186
7.946
7.936
7.920
7.907
7.906
7.905
7.902
7.900
7.900
7.899
7.898
7.887
7.870
7.857
7.856
7.856
7.855
7.851
7.851
7.850
7.849
7.848
7.847
7.847
7.846
7.846
7.845
7.844
7.844
7.841
7.840
7.838
7.837
7.836
7.835
7.835
7.834
7.829
7.827
7.826
7.823
7.822
7.821
7.821
7.817
7.648
7.645
7.634
7.632
7.629
7.618
7.615
7.594
7.592
7.580
7.578
7.576
7.564
7.562
7.373
7.359
7.260
6.216
6.204
6.192
6.183
6.180
6.171
6.159
6.147
2.457
NTs
F3C
100
1H, CDCl3, 500 MHz
6.69
1.04
1
0.971
1.05
1.95
3.04
ppm 10
9
8
7
6
5
4
3
2
1
0

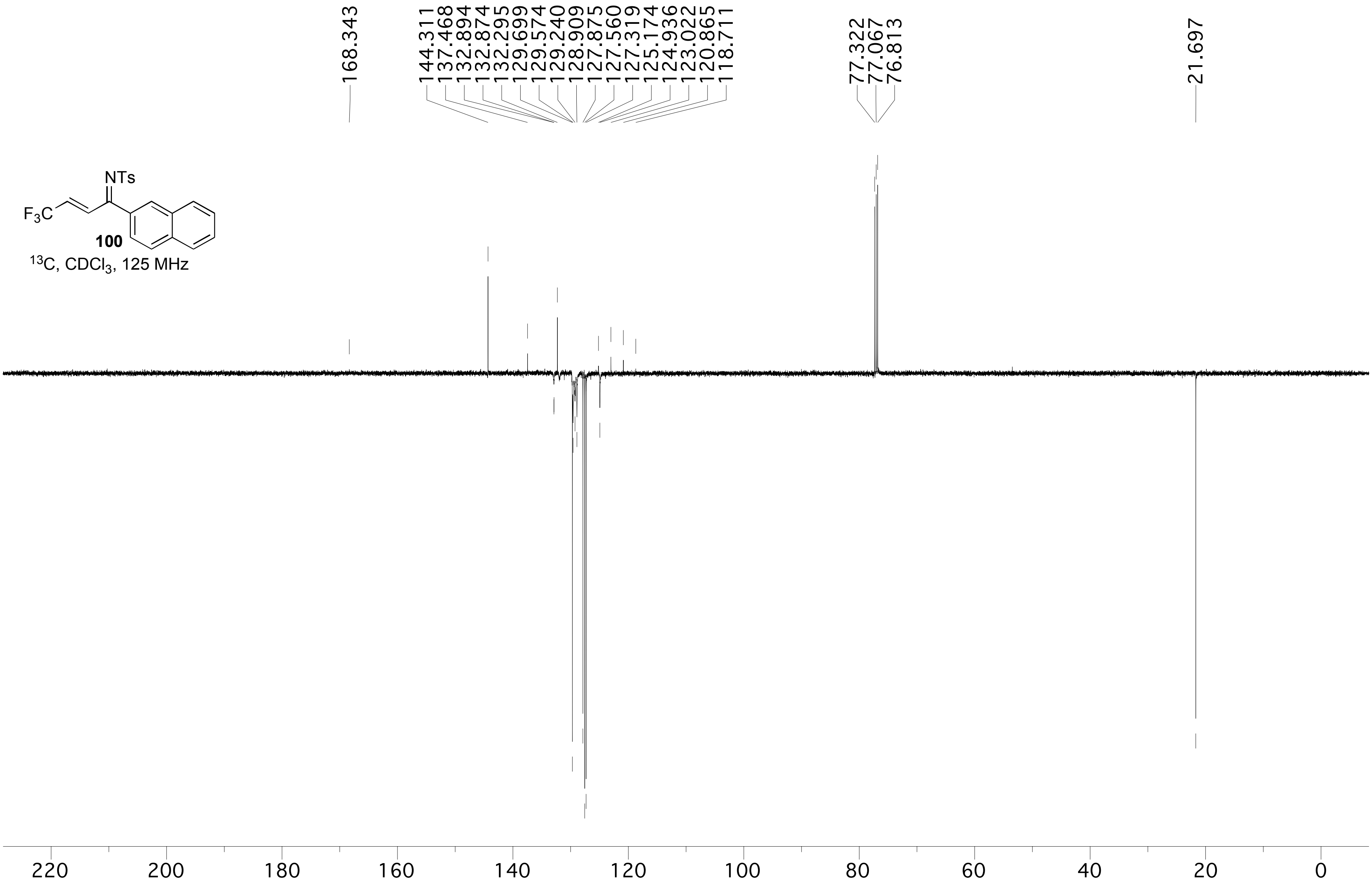
NTs
F_3C
100
^{13}C, $CDCl_3$, 125 MHz
168.343
144.311
137.468
132.894
132.874
132.295
129.699
129.574
129.240
128.909
127.875
127.560
127.319
125.174
124.936
123.022
120.865
118.711
77.322
77.067
76.813
21.697
220
200
180
160
140
120
100
80
60
40
20
0

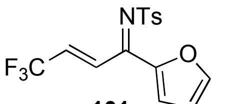

101

^{1}H, $(CD_3)S{=}O$, 300 MHz

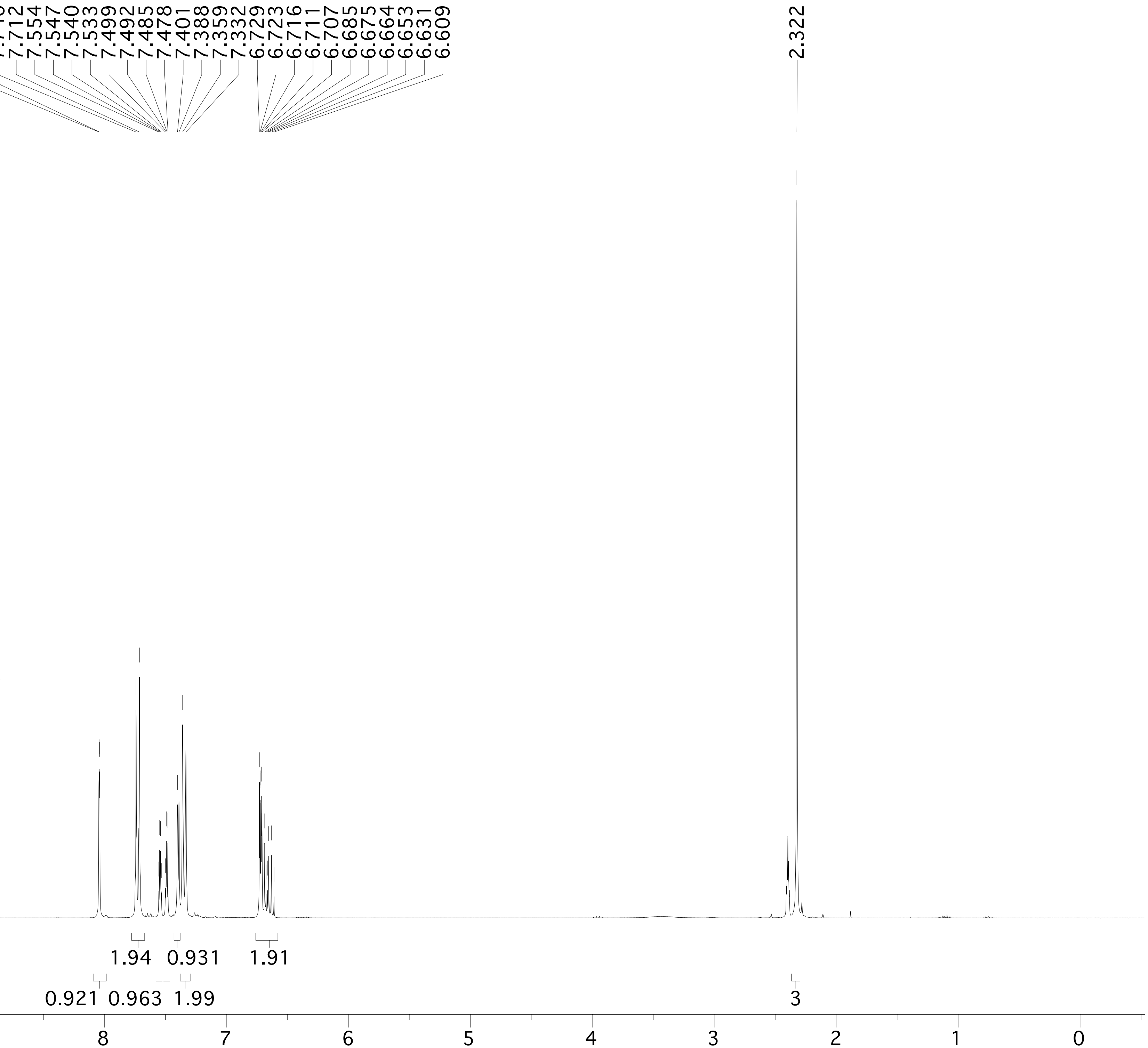

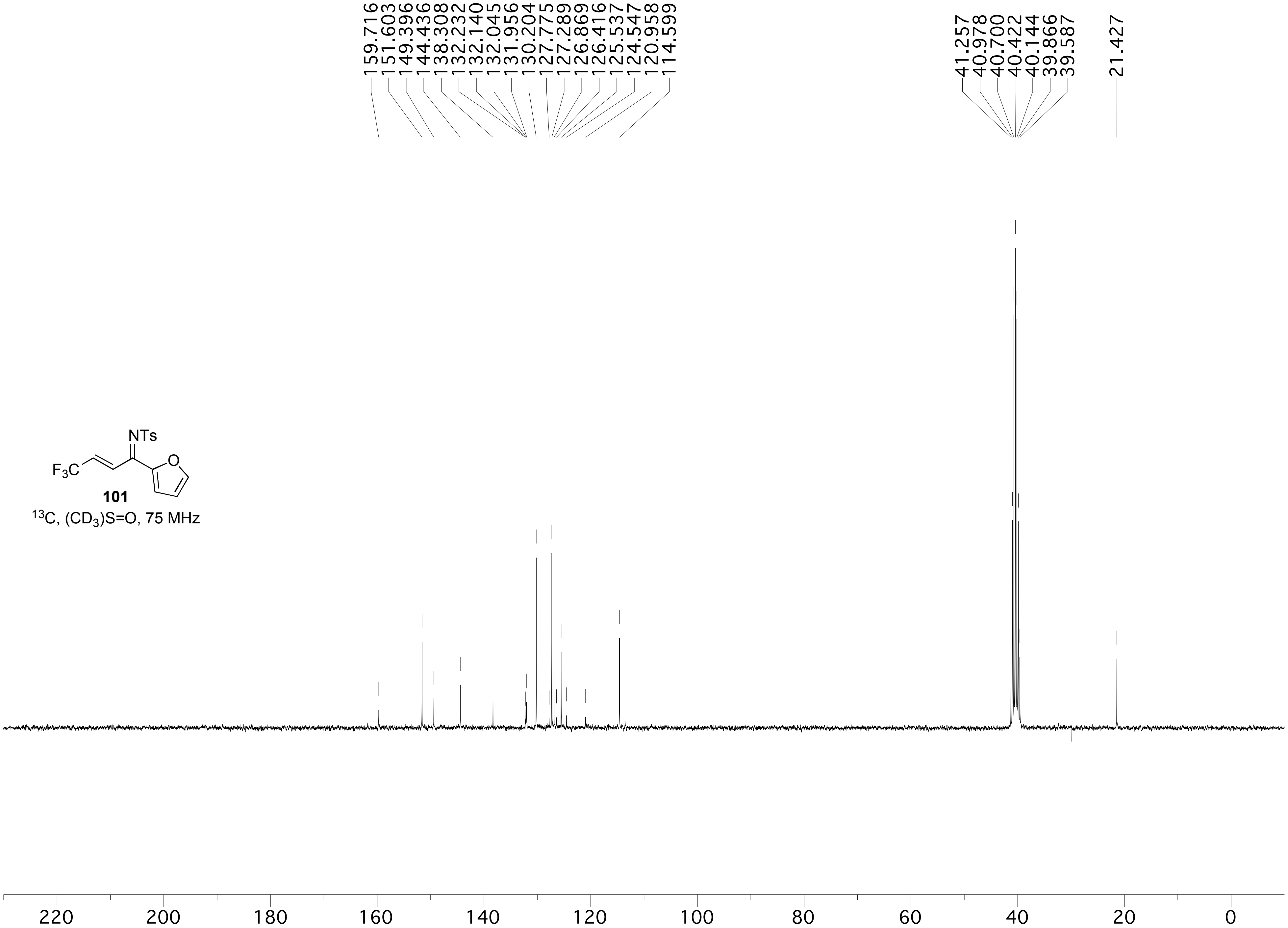
159.716
151.603
149.396
144.436
138.308
132.232
132.140
132.045
131.956
130.204
127.775
127.289
126.869
126.416
125.537
124.547
120.958
114.599
41.257
40.978
40.700
40.422
40.144
39.866
39.587
21.427
NTs
F_3C
O
101
^{13}C, $(CD_3)S{=}O$, 75 MHz
220
200
180
160
140
120
100
80
60
40
20
0

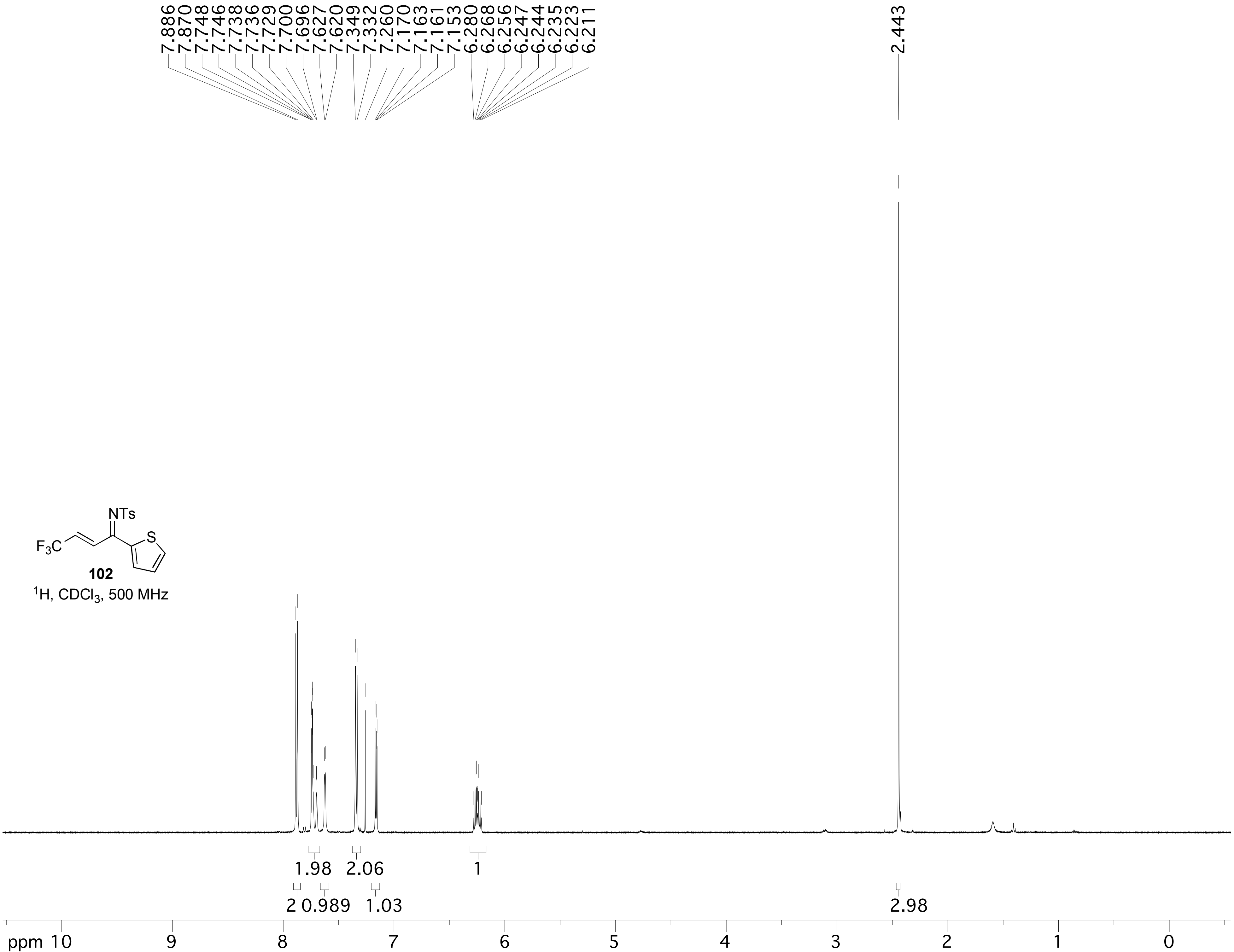

102

^{1}H, $CDCl_3$, 500 MHz

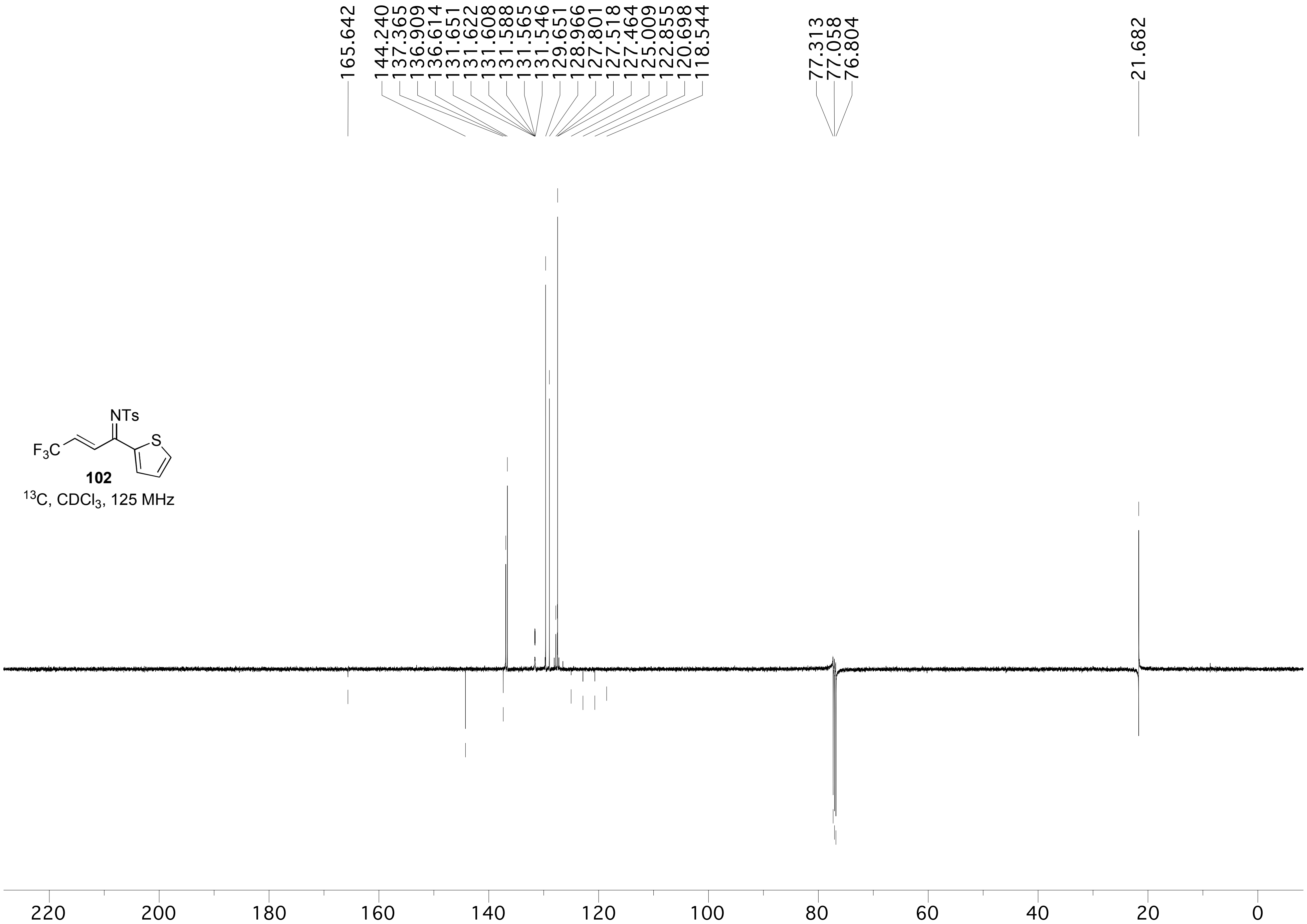

102

^{13}C, $CDCl_3$, 125 MHz

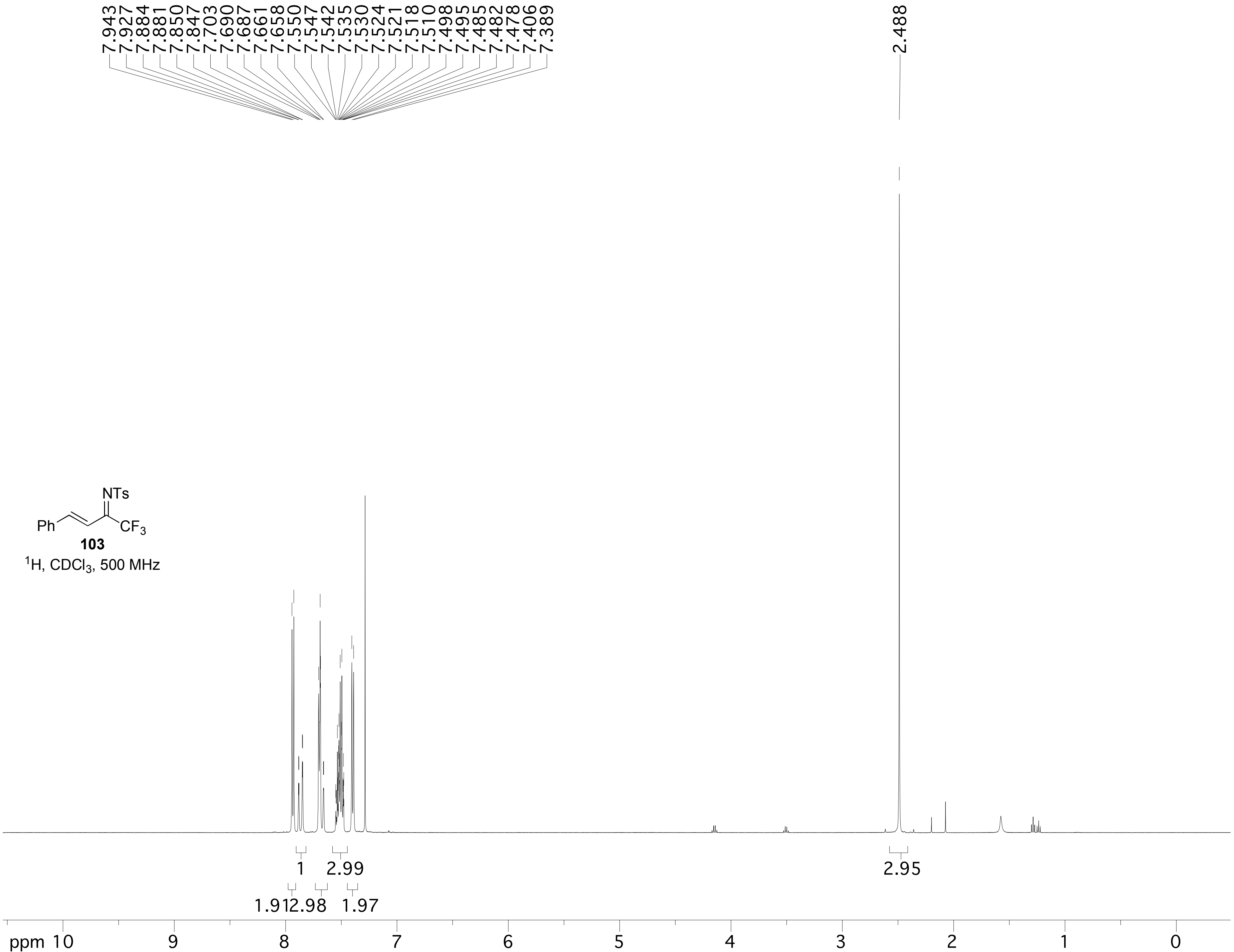
7.943
7.927
7.884
7.881
7.850
7.847
7.703
7.690
7.687
7.661
7.658
7.550
7.547
7.542
7.535
7.530
7.524
7.521
7.518
7.510
7.498
7.495
7.485
7.482
7.478
7.406
7.389
2.488
NTs
Ph
CF3
103
1H, CDCl3, 500 MHz
1
2.99
2.95
1.91
2.98
1.97
ppm 10
9
8
7
6
5
4
3
2
1
0

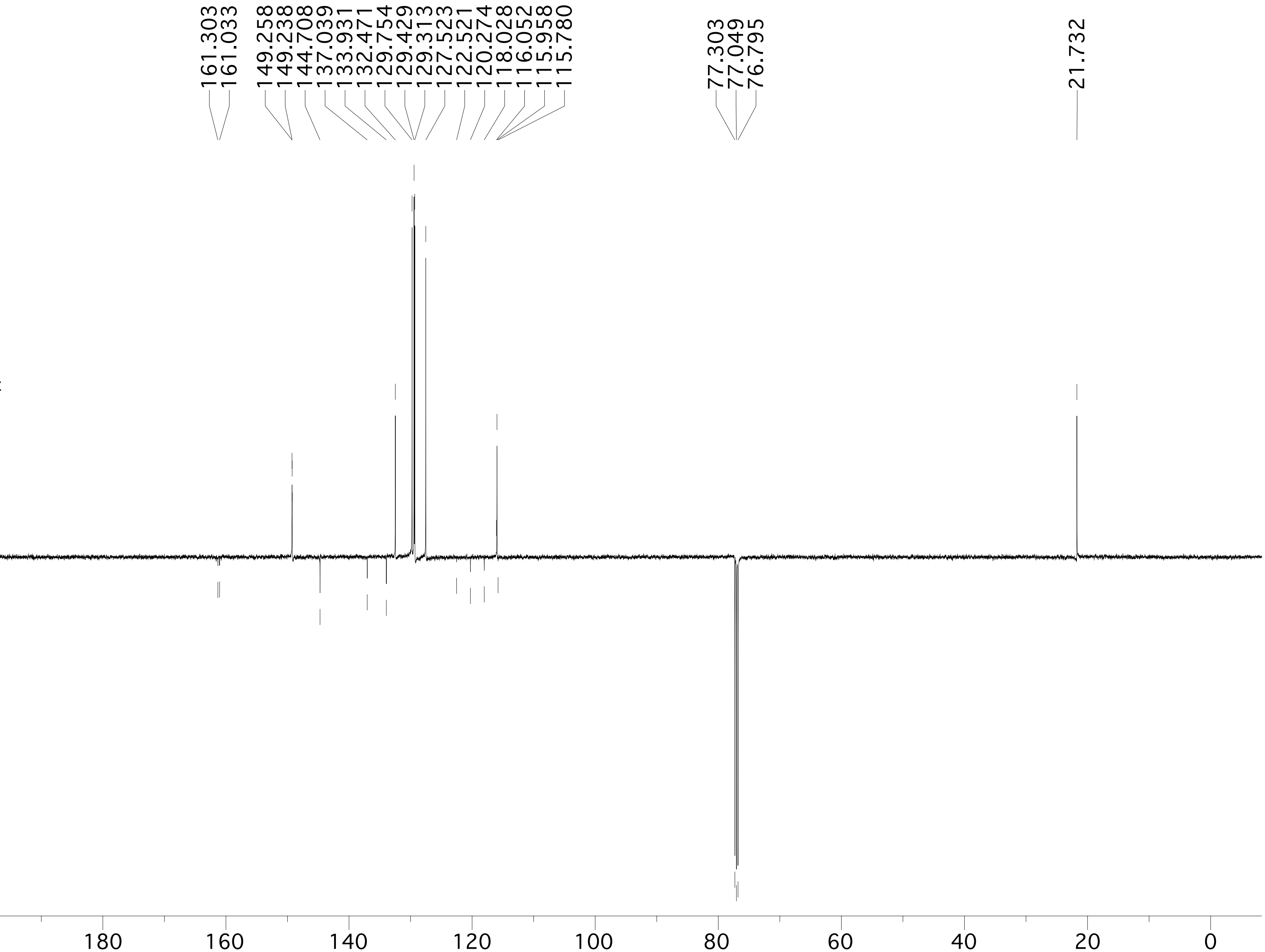

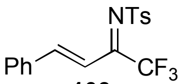

103

^{13}C, $CDCl_3$, 125 MHz

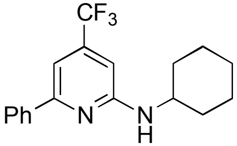

104

^1H, $CDCl_3$, 500 MHz

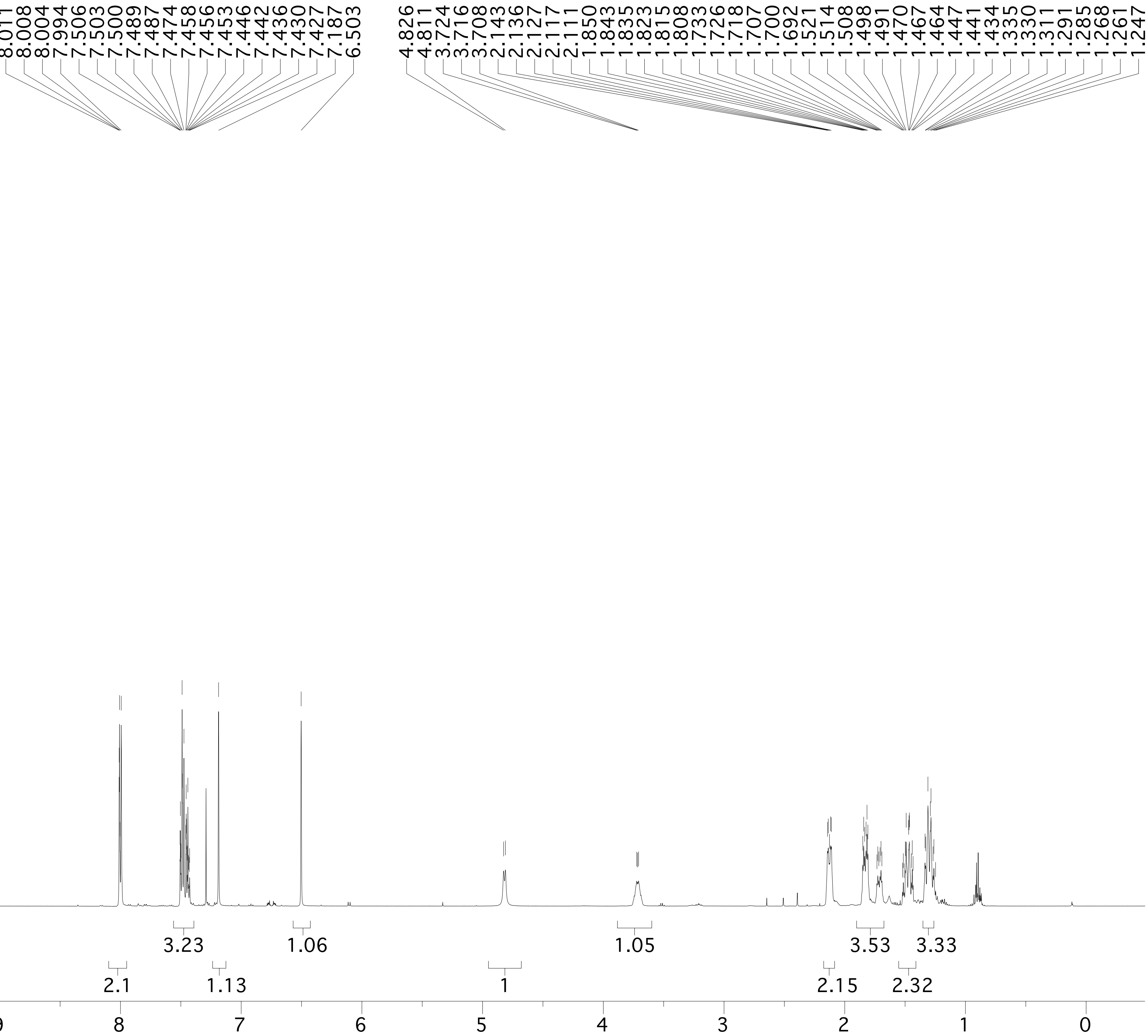

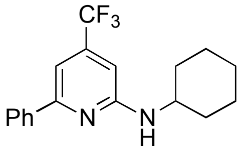

104

^{13}C, $CDCl_3$, 100 MHz

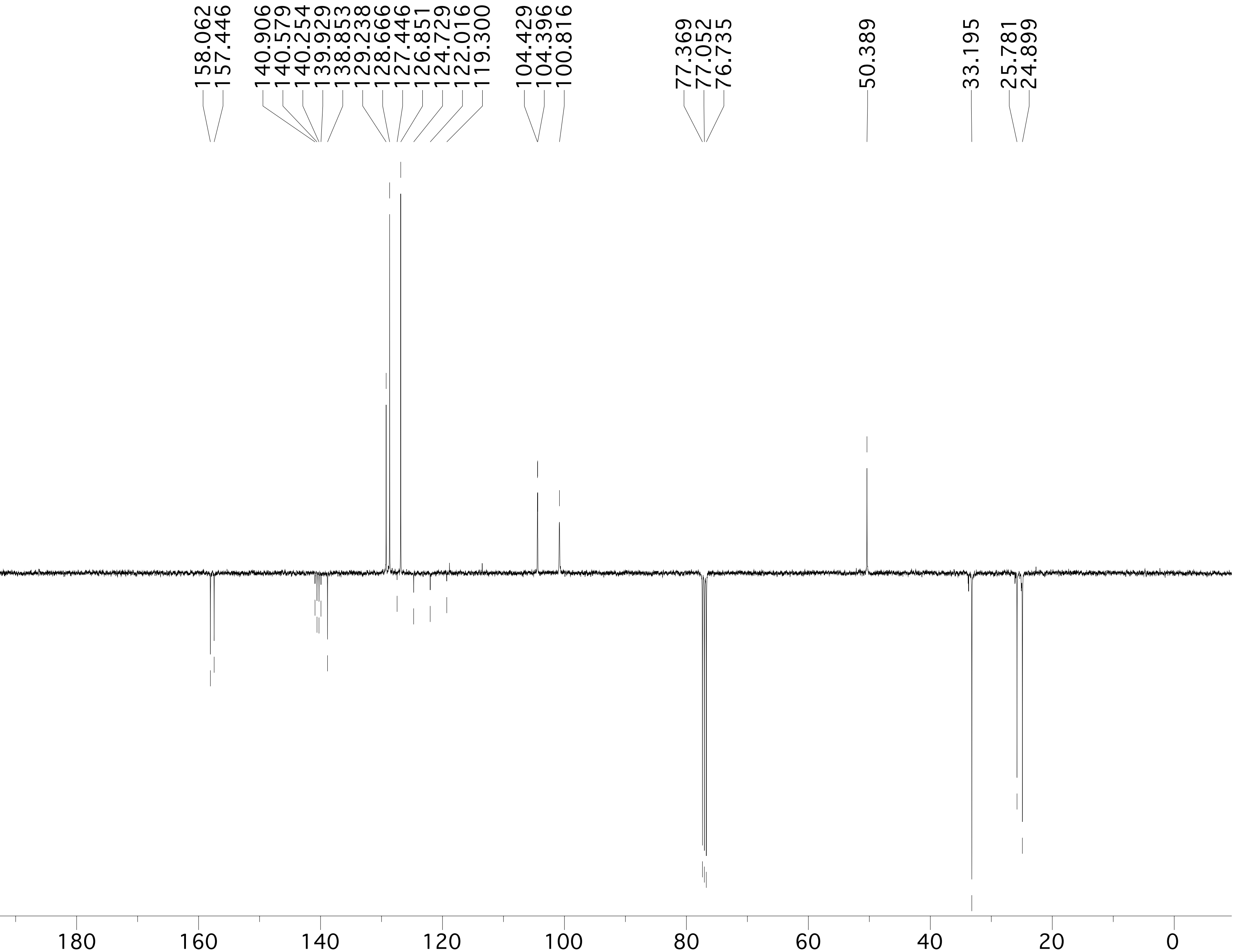

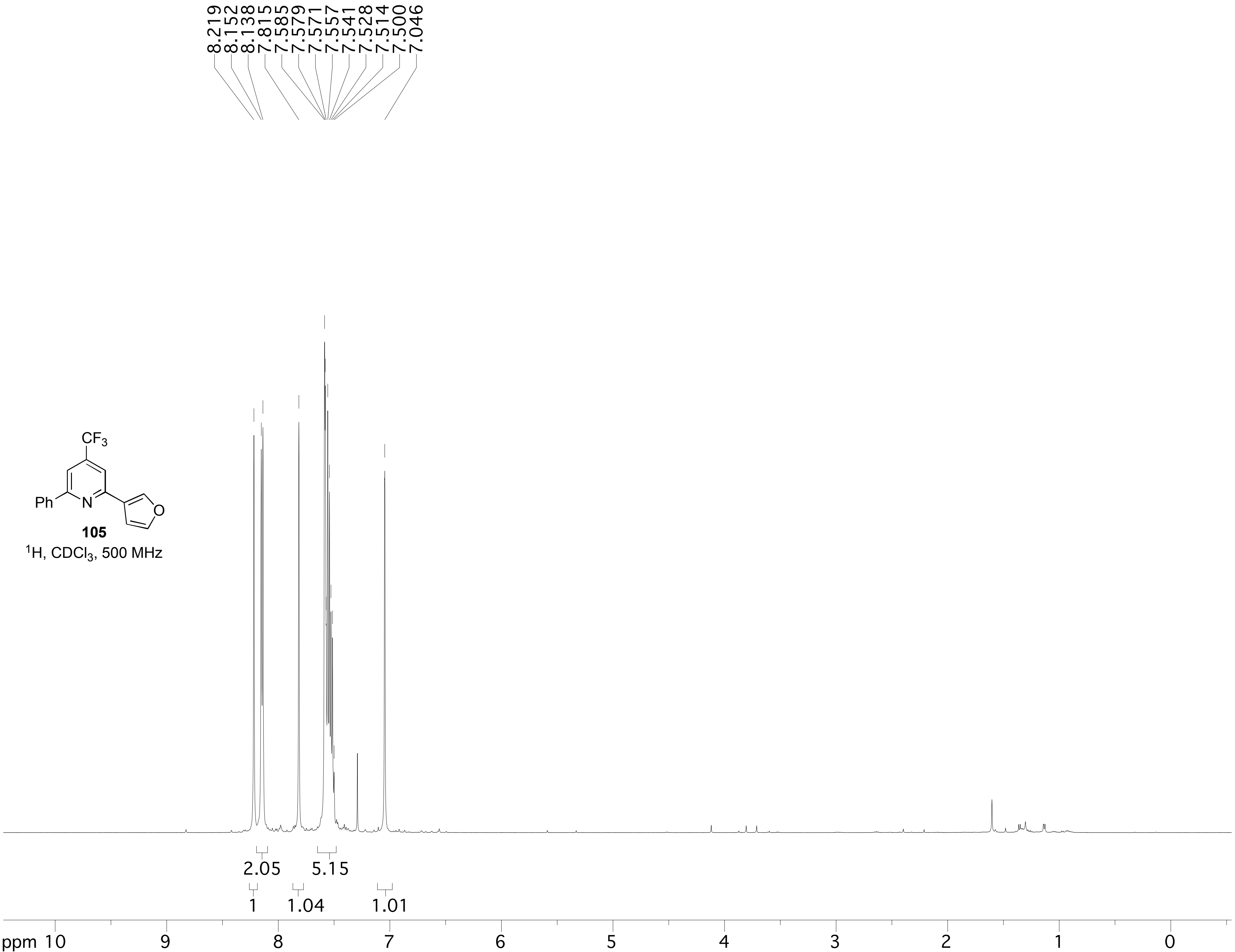

8.219
8.152
8.138
7.815
7.585
7.579
7.571
7.557
7.541
7.528
7.514
7.500
7.046
CF3
Ph
N
O
105
1H, CDCl3, 500 MHz
2.05
5.15
1
1.04
1.01
ppm 10
9
8
7
6
5
4
3
2
1
0

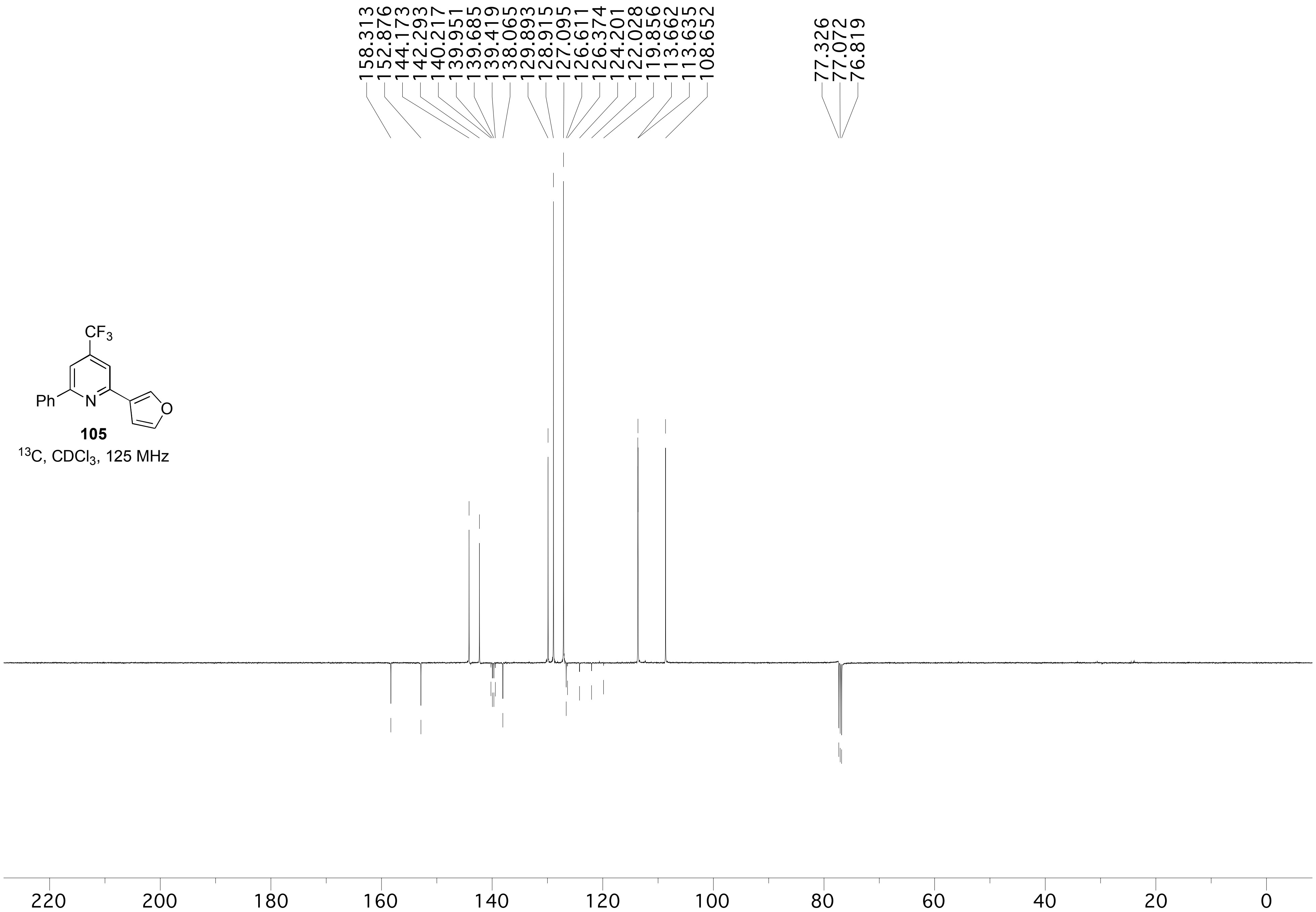

105

^{13}C, $CDCl_3$, 125 MHz

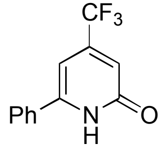

106

^{1}H, $CDCl_3$, 300 MHz

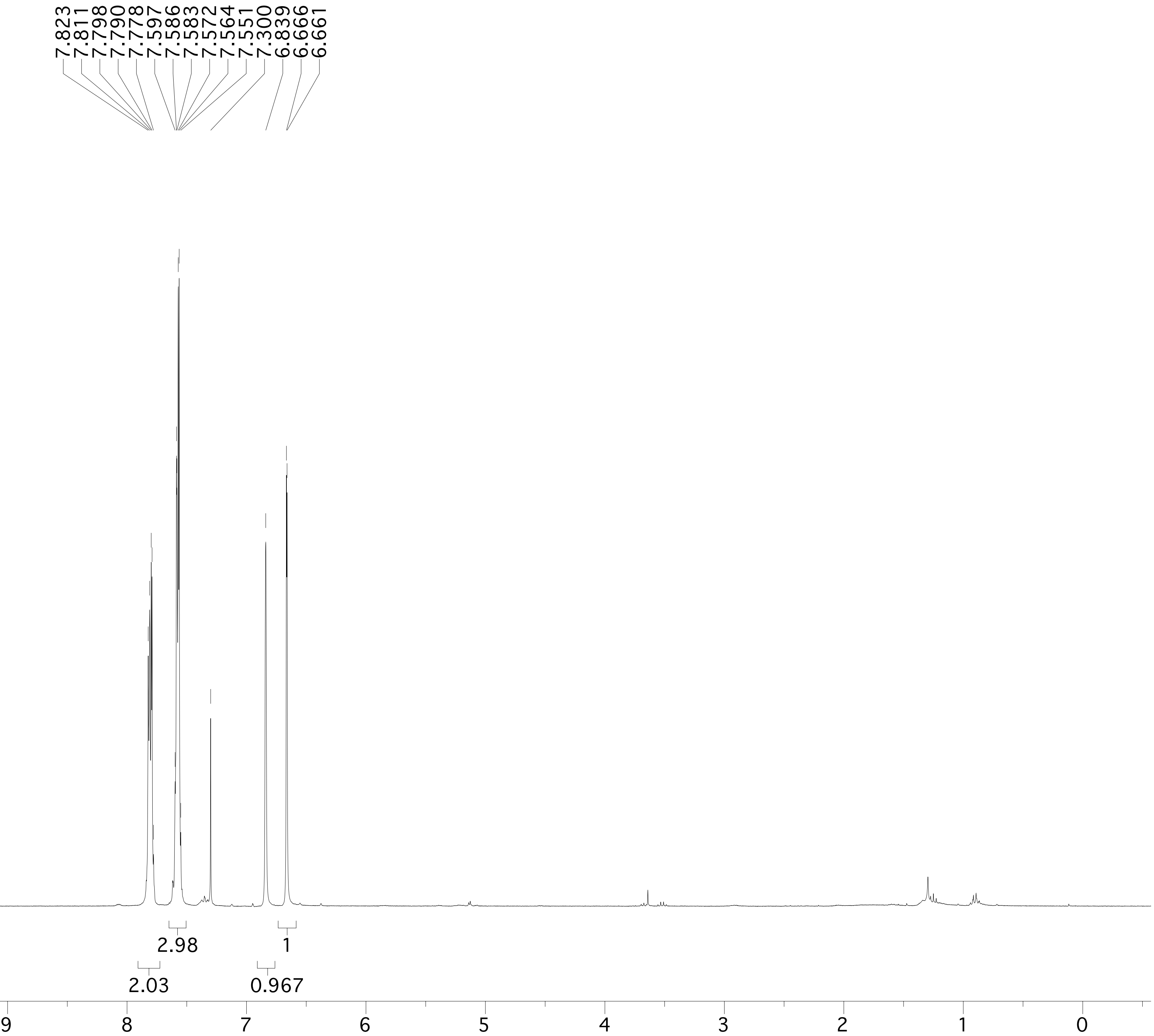

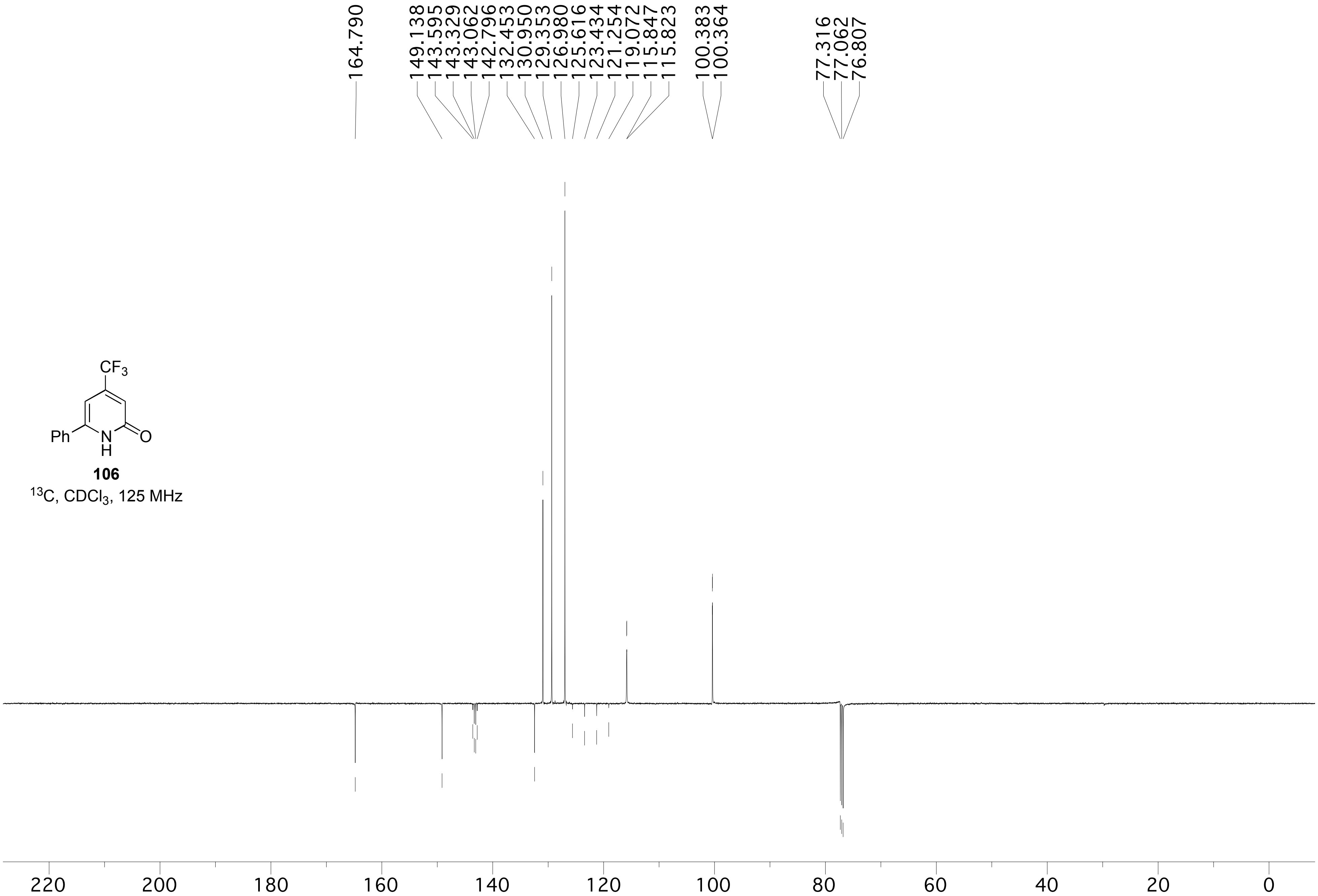

106

^{13}C, $CDCl_3$, 125 MHz

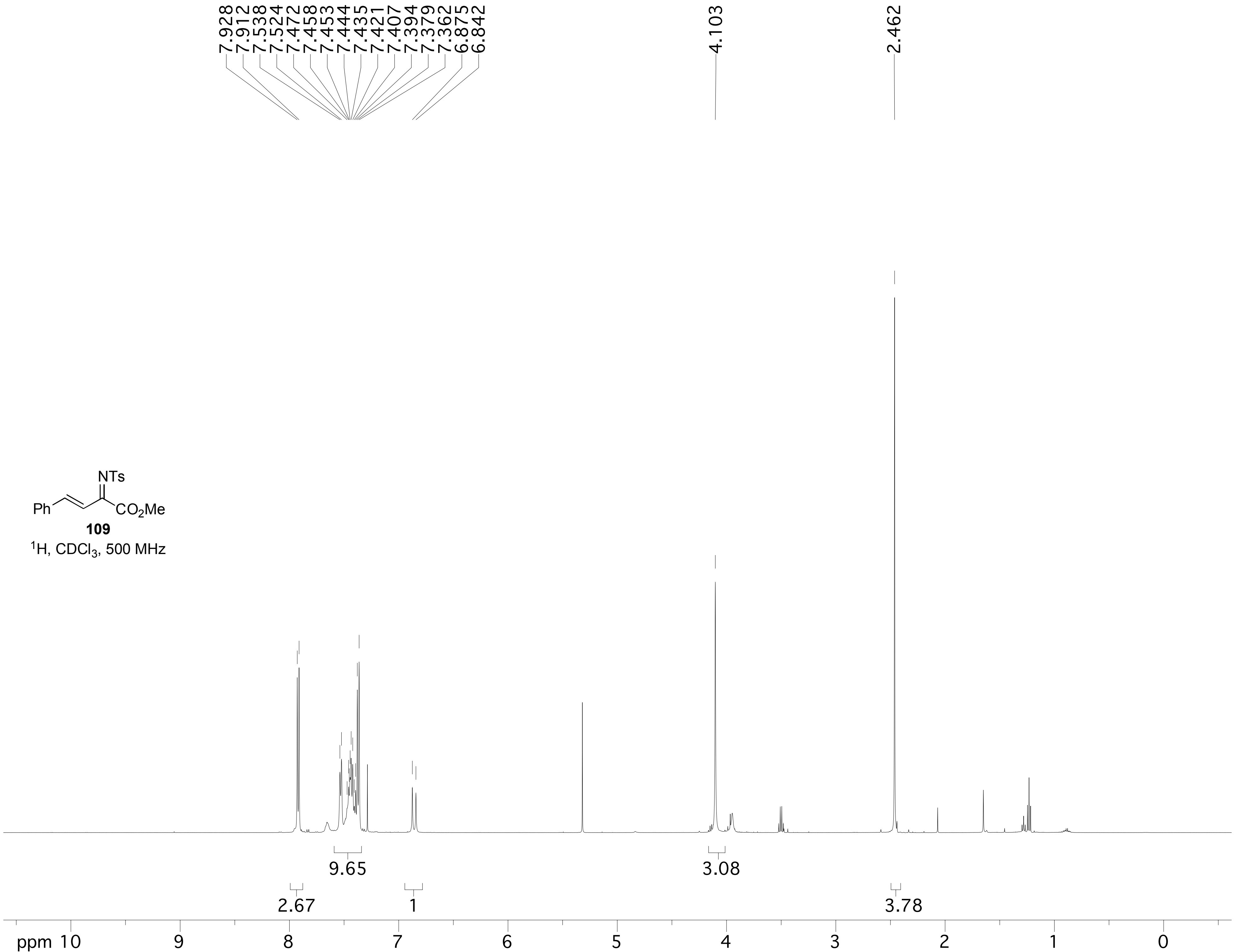

109

^1H, $CDCl_3$, 500 MHz

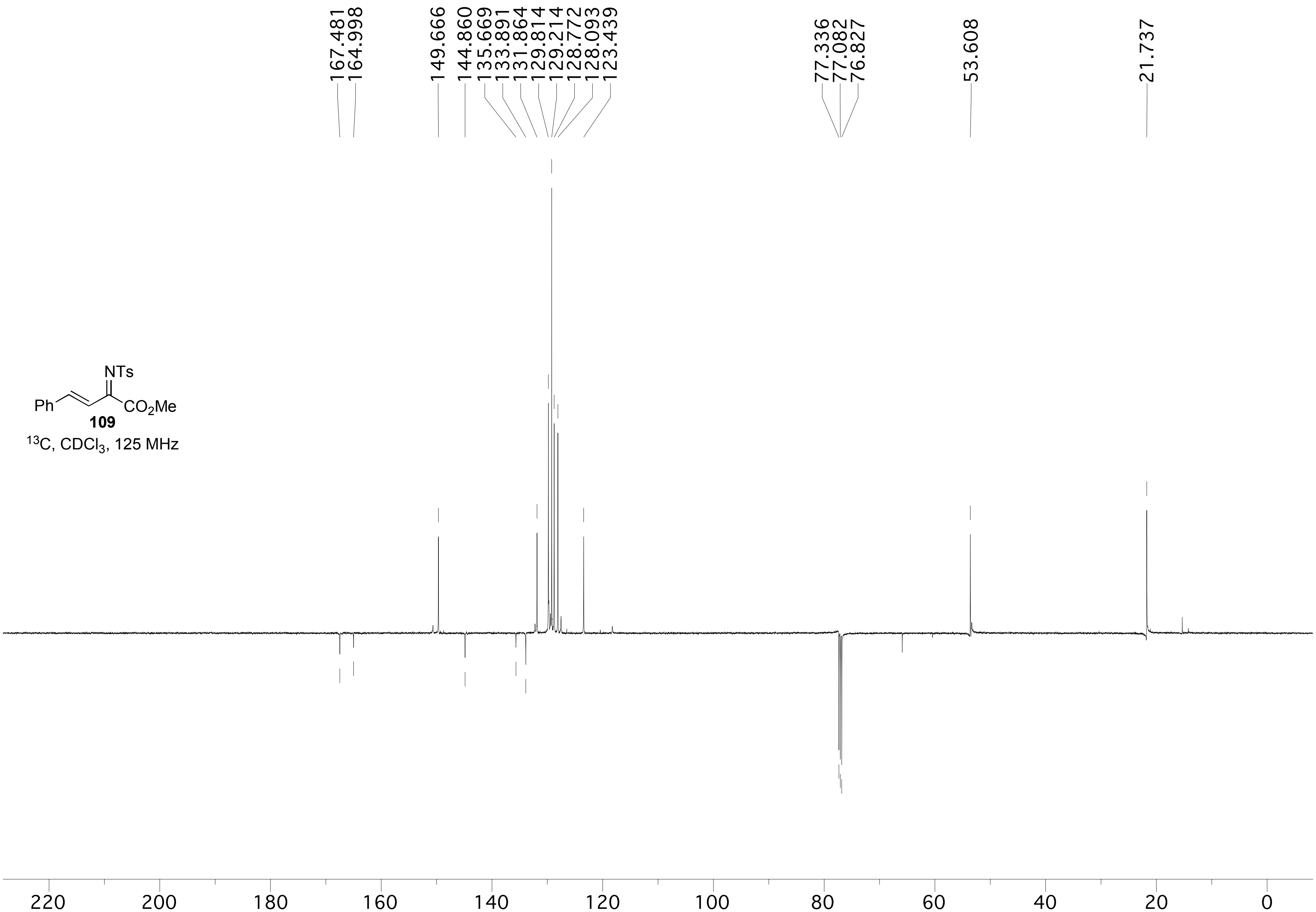
167.481
164.998
149.666
144.860
135.669
133.891
131.864
129.814
129.214
128.772
128.093
123.439
77.336
77.082
76.827
53.608
21.737
NTs
Ph
CO_2Me
109
^{13}C, $CDCl_3$, 125 MHz
220
200
180
160
140
120
100
80
60
40
20
0

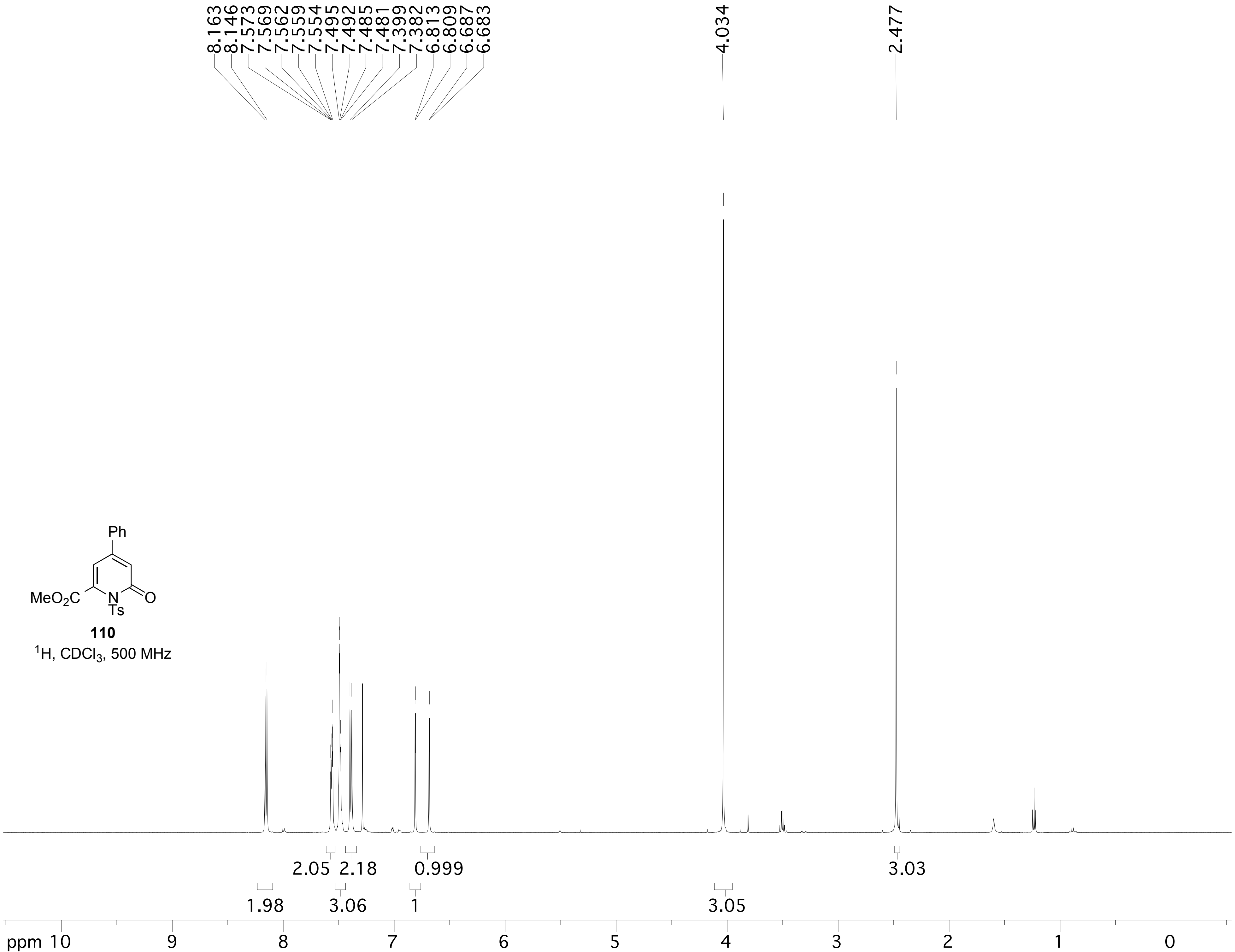
8.163
8.146
7.573
7.569
7.562
7.559
7.554
7.495
7.492
7.485
7.481
7.399
7.382
6.813
6.809
6.687
6.683
4.034
2.477
Ph
MeO_2C
N
O
Ts
110
^1H, $CDCl_3$, 500 MHz
2.05
2.18
0.999
3.03
1.98
3.06
1
3.05
ppm 10
9
8
7
6
5
4
3
2
1
0

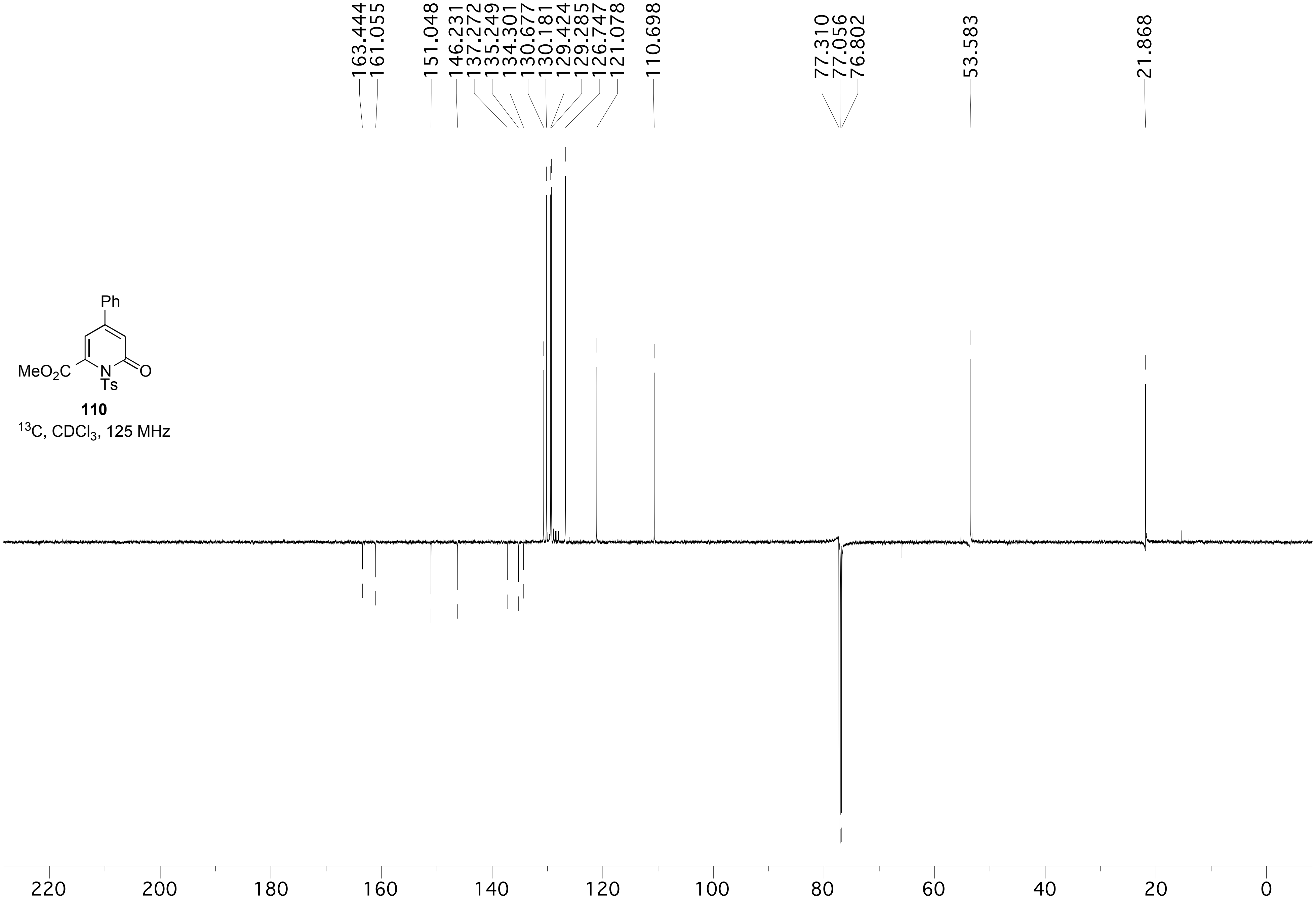

110

^{13}C, $CDCl_3$, 125 MHz

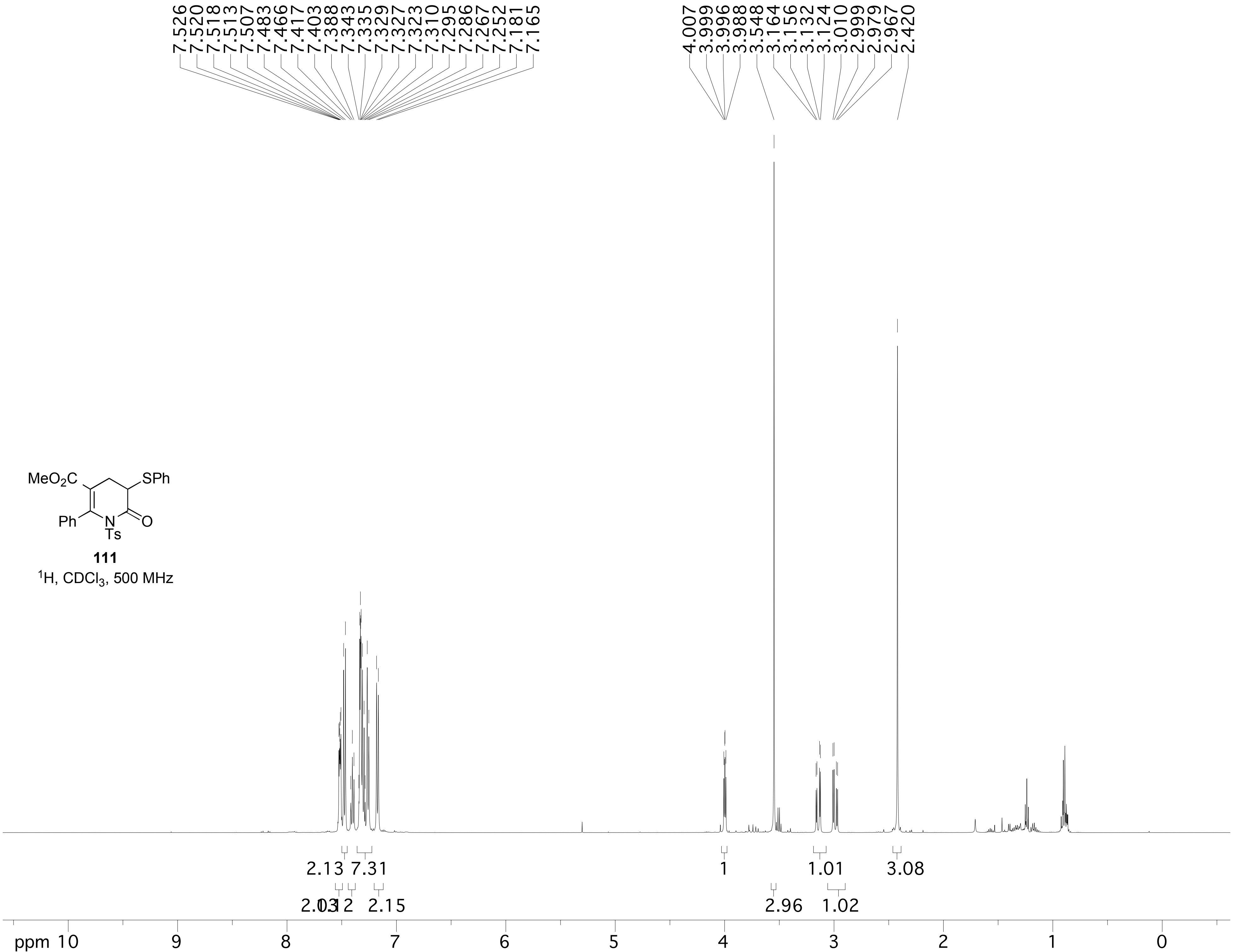

7.526
7.520
7.518
7.513
7.507
7.483
7.466
7.417
7.403
7.388
7.343
7.335
7.329
7.327
7.323
7.310
7.295
7.286
7.267
7.252
7.181
7.165
4.007
3.999
3.996
3.988
3.548
3.164
3.156
3.132
3.124
3.010
2.999
2.979
2.967
2.420
MeO2C
SPh
Ph
N
Ts
O
111
1H, CDCl3, 500 MHz
2.13
7.31
1
1.01
3.08
2.15
2.96
1.02
ppm 10
9
8
7
6
5
4
3
2
1
0

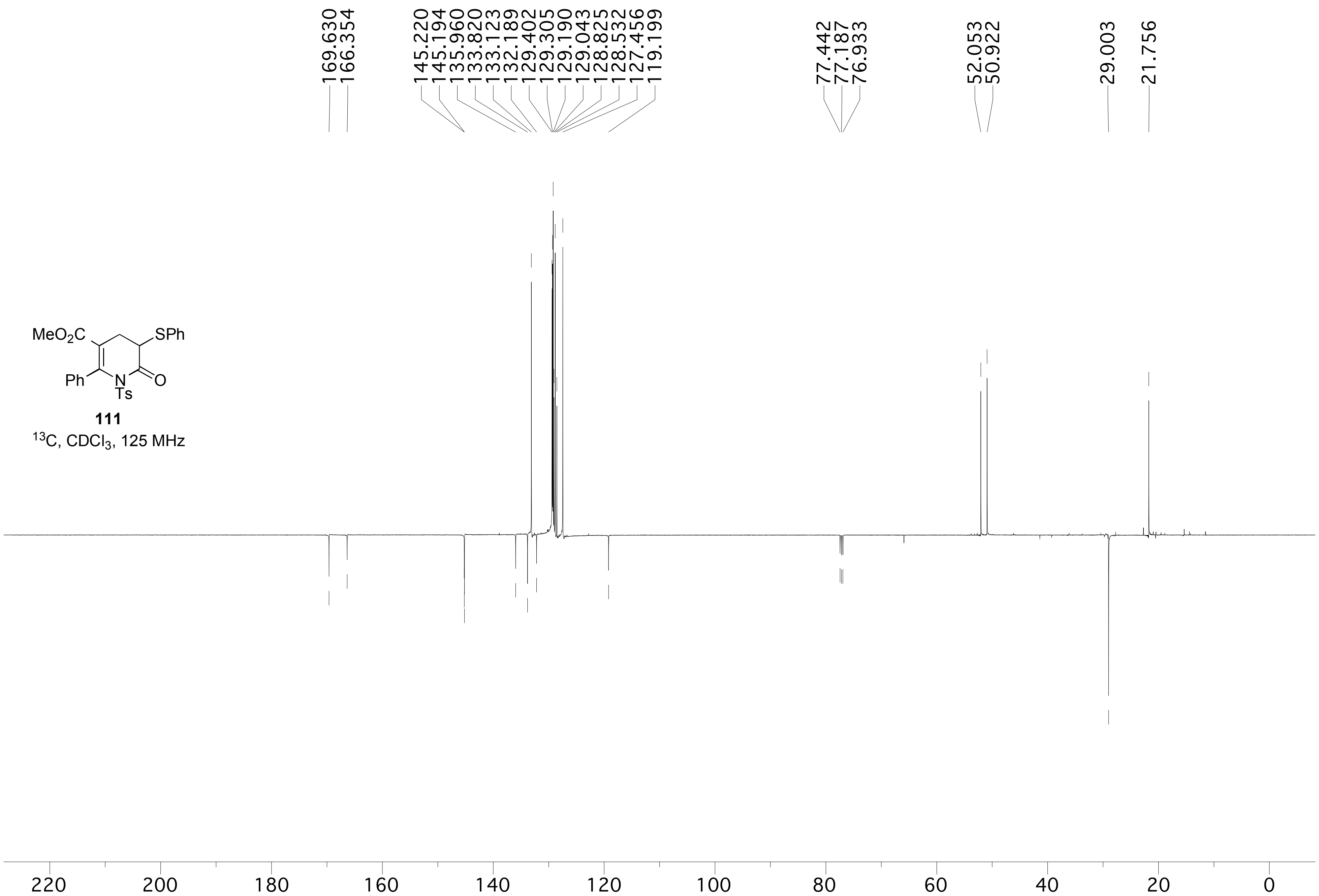
169.630
166.354
145.220
145.194
135.960
133.820
133.123
132.189
129.402
129.305
129.190
129.043
128.825
128.532
127.456
119.199
77.442
77.187
76.933
52.053
50.922
29.003
21.756
MeO$_2$C
SPh
Ph
N
O
Ts
111
^{13}C, CDCl$_3$, 125 MHz
220
200
180
160
140
120
100
80
60
40
20
0
